# Gene socialization: gene order, GC content and gene silencing in *Salmonella*

**DOI:** 10.1186/1471-2164-10-597

**Published:** 2009-12-11

**Authors:** Nikolas Papanikolaou, Kalliopi Trachana, Theodosios Theodosiou, Vasilis J Promponas, Ioannis Iliopoulos

**Affiliations:** 1 Division of Medical Sciences, University of Crete Medical School, Heraklion 71110, Crete, Greece; 2 EMBL, Meyerhofstrasse 1, 69117 Heidelberg, Germany; 3 Department of Informatics, School of Natural Sciences, Aristotle University of Thessaloniki, Thessaloniki, Greece; 4 Bioinformatics Research Laboratory, Department of Biological Sciences, University of Cyprus, CY 1678, Nicosia, Cyprus

## Abstract

**Background:**

Genes of conserved order in bacterial genomes tend to evolve slower than genes whose order is not conserved. In addition, genes with a GC content lower than the GC content of the resident genome are known to be selectively silenced by the histone-like nucleoid structuring protein (H-NS) in *Salmonella*.

**Results:**

In this study, we use a comparative genomics approach to demonstrate that in *Salmonella*, genes whose order is not conserved (or genes without homologs) in closely related bacteria possess a significantly lower average GC content in comparison to genes that preserve their relative position in the genome. Moreover, these genes are more frequently targeted by H-NS than genes that have conserved their genomic neighborhood. We also observed that duplicated genes that do not preserve their genomic neighborhood are, on average, under less selective pressure.

**Conclusions:**

We establish a strong association between gene order, GC content and gene silencing in a model bacterial species. This analysis suggests that genes that are not under strong selective pressure (evolve faster than others) in *Salmonella *tend to accumulate more AT-rich mutations and are eventually silenced by H-NS. Our findings may establish new approaches for a better understanding of bacterial genome evolution and function, using information from functional and comparative genomics.

## Background

The conservation of gene order has been found to play an essential role in genome evolution. More specifically, proteins encoded by genes of conserved order in bacteria tend to evolve more slowly when compared to proteins encoded by genes without a conserved order [1,2] and genes with similar or related functions tend to occur in adjacent chromosomal positions in yeast [3]. Moreover, genes with conserved order were found to evolve at similar rates [4] and, in prokaryotes, proteins encoded by genes with conserved order appear to interact physically [1]. It has also been shown that in eukaryotes essential genes are clustered in regions with low recombination rates [5], whereas in bacteria essential genes are more conserved than non essential genes [6]. In addition, it has been reported that the number of interactions involved in a protein network is directly correlated with the rate of evolution among these proteins [7] and that highly expressed genes evolve slowly [8,9]. A case in point for the role of gene order in evolution can be illustrated by duplicated genes [10]. Following a duplication event one of the two (paralog) genes might keep its original function, whereas the other one might be under less selective pressure. Yet, it is not always readily apparent which duplicated genes evolve faster. There have been reports that have marginally correlated sequence conservation with genome context [11], but there must be other, yet unknown, functional features that determine the fate of duplicate genes.

Recently, two research groups observed independently that the histone-like nucleoid structuring protein (H-NS) plays an important role as a general transcriptional repressor of a large number of genes in *Salmonella enterica *serovar Typhimurium LT2 (*S*. Typhimurium) [12,13]. H-NS is a protein that is believed to play an essential role in the organization and compaction of bacterial chromatin as well as in transcriptional regulation for many bacterial genes [14-16]. H-NS binds to these genes and silences them transcriptionally. An apparent common feature of genes silenced by H-NS is that their GC content is significantly lower than the overall GC content of the *Salmonella *genome [13]. Additionally, a large proportion of these H-NS repressed genes is predicted to have been acquired from a foreign source (horizontally transferred genes, HTGs) [13,15], a fact in agreement with the observation that HTGs are relatively AT-rich [17,18]. It has been suggested that this may be a defensive mechanism against foreign genetic material without loosing the benefit of future usage of this material if necessary [15].

Herein, we aimed to test the correlation between gene order conservation, gene duplication and H-NS dependent silencing in *S*. Typhimurium, using *Escherichia coli *K12 as a reference genome in order to identify the conservation or loss of gene order along the bacterial chromosome. We have also attempted to associate the above mentioned features with GC content and gene essentiality.

## Results

### Evolutionary rate and GC content are related to genomic neighborhood conservation

**Figure 1:**
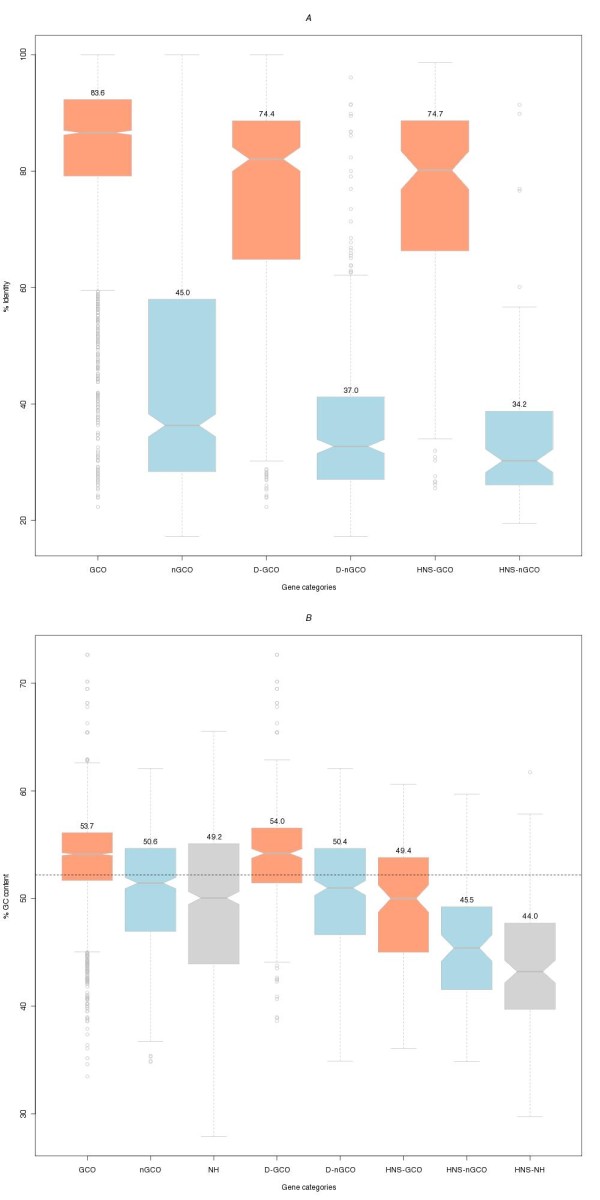
**Correlation of gene order conservation with sequence identity and GC content (*Salmonella *vs *E. coli *K12)**. **(A) **Sequence identity frequency distributions of proteins encoded by GCO/nGCO genes for datasets used in this study. Boxandwhisker plots illustrate the differences between the medians and the dispersion of the respective datasets. Orange and light blue represent GCO versus nGCO datasets. Average values are displayed on top of the box plots. The two leftmost box plots (GCO, nGCO) depict differences between those two gene classes within the overall protein sequence data set. The next two data sets depict differences between duplicated GCO genes (DGCO) and duplicated nGCO genes (DnGCO). The last two datasets represent HNS repressed genes (HNSGCO: HNS repressed GCO genes, HNSnGCO: HNS repressed nGCO genes). **(B) **GC content of GCO genes, nGCO genes and genes with no homolog in *E. coli *K12 NH for datasets used in this study. Additionally to the coloring scheme of Fig. 1A, we use light grey for sequences that had no homolog in *E. coli *K12. The dashed horizontal line corresponds to the overall GC content of *S*. Typhimurium genome (52.2%). (For a more detailed description, including statistical analysis see Additional files 8, 9).

We compared the proteins predicted to be encoded in the completely sequenced genome of *S*. Typhimurium [19] against the proteins encoded in the genome of *E. coli *K12 [20], both obtained from the NCBI Genomes Division ftp://ftp.ncbi.nih.gov/genomes/Bacteria/ using BLASTP [21]. Using the criteria described in Materials and Methods, we identify 3584 homologs (out of 4425 *Salmonella *proteins in total) between these two highly related bacterial species, of which 3024 were found to be encoded by genes of conserved order (GCO) and 560 proteins encoded by genes that have lost their order (nGCO) (Additional file 1). The remaining 841 genes either fell below the E-value threshold or exhibited significant similarities only in short segments, when compared to their full length sequences and thus were not considered to have a homolog in *E. coli *K12. The 3024 homolog pairs with conserved gene order share an average sequence identity of 83.6%, whereas the 560 proteins that were encoded by genes with no order conservation share an average sequence identity of 45.0% (Fig. 1A). The difference between the two groups is statistically significant according to a Wilcoxon rank-sum test (W = 1522518, P-value < 2.2e-16). Subsequent analysis revealed statistically significant differences in the GC content for the three aforementioned classes of *Salmonella *genes (GCO, nGCO, without an *E. coli *K12 homolog), which was 53.7%, 50.6% and 49.2% respectively (Fig. 1B). Taking into account that the GC content of the *Salmonella *genome is 52.2% it is readily apparent that GCO are enriched in Gs and Cs. This sizable decrease in GC content in the last two groups could be explained either by the presence of a large number of horizontally transferred genes in the last two groups, or by an accumulation of AT-rich mutations in genes belonging to these groups.

In order to check the former hypothesis, we cross-checked the three groups for the existence of HTGs in the *Salmonella *genome using HGT-DB, a database of putative horizontally transferred genes in prokaryotes [22]. As expected, the majority of predicted HTGs fall into the last two categories (nGCO, no homolog), but after removing HTGs from the complete gene set, the results were not affected (Additional file 2). However, this observation could be biased because the methodological approach used in HGT-DB to infer lateral gene transfer events, is largely dependent on GC content [22]. The latter hypothesis could be explained by our finding that the last two categories of genes may be under weaker selective pressure than the first one and, consequently, accumulating a larger number of mutations, especially C-to-T mutations which are known to be the most common ones under neutral evolution [23]. The observation that genomes of intracellular pathogens tend to be AT rich for energetic and resources availability reasons [24] could also explain the aforementioned result. After a speciation event occurs, some genes might remain GCO and continue to evolve slowly. This is probably the result of the strong evolutionary pressure to maintain their relative order, assisting the preservation of protein function or interaction with neighboring genes. On the other hand, other genes might lose their relative genomic position and start evolving faster. Genes in *Salmonella *with no homologs in *E. coli *K12 could either be HTGs or genes that have lost their genomic neighborhood and subsequently evolved to such an extent that no significant sequence similarities could be detected to support homology to any *E. coli *K12 gene.

**Figure 2:**
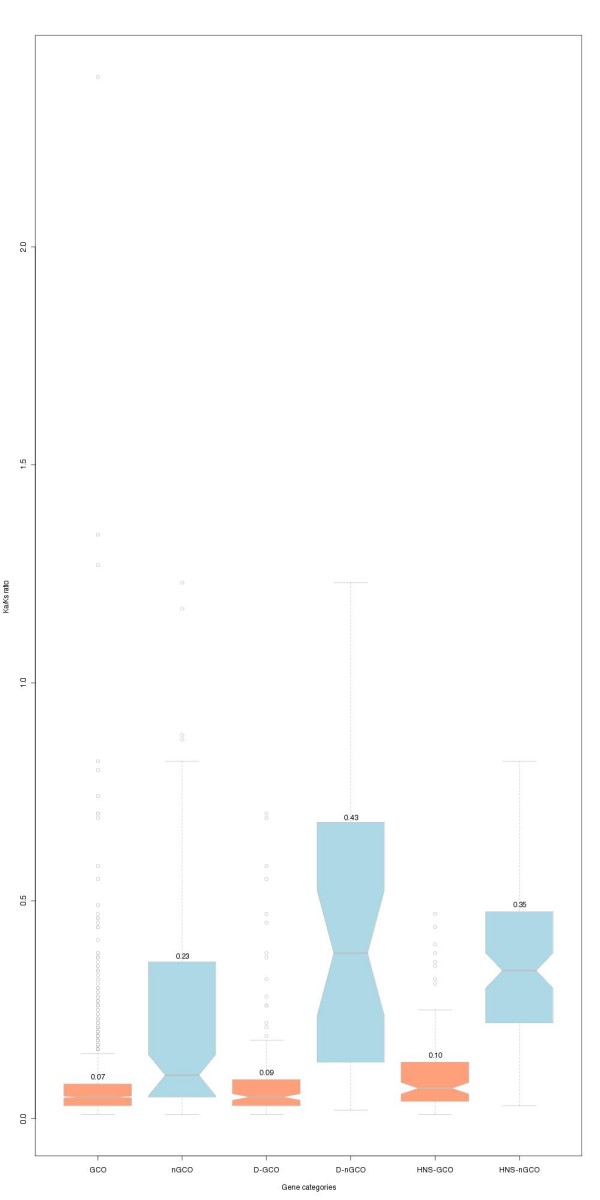
**Boxandwhisker plots of Ka/Ks ratio distributions for GCO and nGCO genes for various datasets used in this study**. Significant differences in Ka/Ks ratios were observed between GCO and nGCO genes both in the overall dataset (Wilcoxon ranksum test: W = 203313, Pvalue = 0, standard deviations 0.09 and 0.18 respectively) and in the subset of duplicated genes (DGCO: Duplicated GCO genes, DnGCO: Duplicated nGCO genes. W = 13595.5, Pvalue = 0, standard deviations 0.13 and 0.17 respectively). The same coloring scheme with Fig. 1A. is used for easy comparison.

In order to obtain a quantitative estimate of the selective constraint for the groups of GCO and nGCO genes we calculated the ratio of non-synonymous (Ka) to synonymous (Ks) nucleotide substitutions by projecting the protein sequence alignments to the respective genomic sequences. The mean Ka/Ks ratio for GCO and nGCO groups was calculated to be 0.07 and 0.23 respectively, using the yn00 method [25] as implemented in the PAML package [26] (Fig. 2). This difference is found to be statistically significant and is indicative of a stronger selective constraint under which GCO genes are evolving in *Salmonella*.

### The fate of duplicated genes is determined by gene order conservation

The most striking evidence that GCO genes evolve, on average, significantly slower than the nGCO genes, is inferred from an analysis of the subset of duplicated genes. We performed an all-against-all self-comparison of proteins encoded in the *Salmonella *genome and detected all groups of genes that could be assigned as homologs in the *Salmonella *genome that share the same best hit in the *E. coli *K12 genome (Additional file 3). We identified 687 such genes (15.5% of the complete *Salmonella *gene set) 343 of which are GCO (49.9%) and 344 are nGCO (51.1%). When compared to the same figures for the complete *Salmonella *gene set (68.3% and 12.6% respectively) the enrichment of the duplicate gene set in nGCO genes is evident. This enrichment probably takes place because, after a duplication event occurs, the selective pressure on one gene to retain its genomic neighborhood becomes less stringent. The proteins of GCO genes in the 343 duplicates share a 74.4% identity (standard deviation sd = 20.2), whereas for the 344 proteins of nGCO genes this figure drops to 37.0% (sd = 14.6; Wilcoxon rank-sum test: W = 106315.5, P-value = 0) (Fig. 1A). This result leads us to two major conclusions: First, as it may be deduced from a comparison of these identity percentages with the result for the complete genome set, the proteins encoded by this subset of duplicated genes evolve faster (in agreement with previous studies [27]). Second, whenever a gene duplication event occurs, the gene that retains its gene order tends to evolve more slowly than the one that travels away from the ancestral gene neighborhood and probably it is the former that keeps the original function. This finding complements an earlier study [11], although different experimental approaches have been employed here. Our results, even though based on the average conservation of putative homologs, corroborate the finding that genomic neighborhood conservation is an important additional criterion that should be taken into account in protein sequence similarity searches. We reached the same conclusions after calculating the Ka/Ks ratio for the two different duplicated gene groups - GCO and nGCO - with respective values 0.09 and 0.43 (Fig. 2). In most cases where all duplicated genes were nGCO, none of the genes exhibited particularly high identity to the common *E. coli *K12 homolog (Additional file 4). An analysis of the average GC content for the duplicated genes (GCO and nGCO) showed a similar pattern with the one of the overall genome (Fig. 1B).

### Essential genes tend to conserve their order

**Figure 3:**
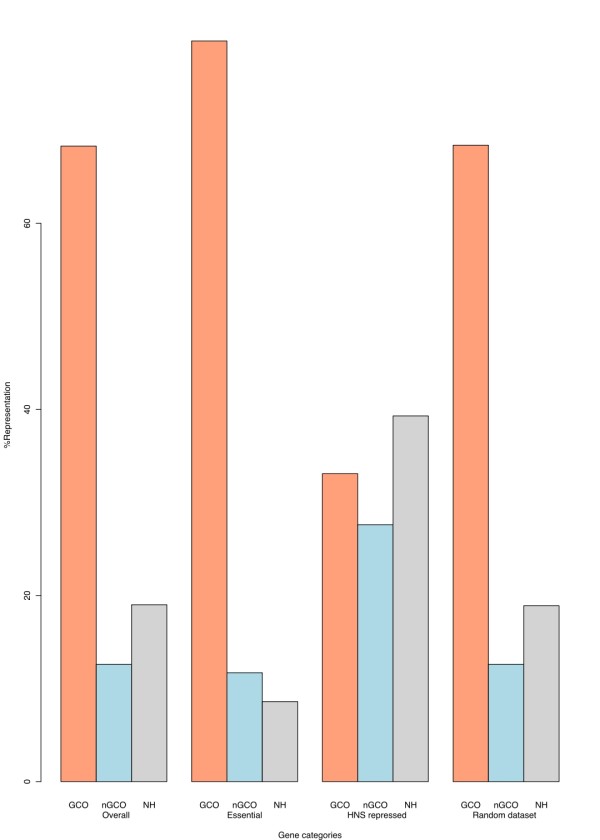
**Distribution of the three categories of genes (GCO, nGCO and genes with no homolog in *E. coli*) in the complete *Salmonella *gene set and in the subsets of HNS repressed genes **[13]**and essential genes **[28]. GCO genes are overrepresented in the essential gene set and underrepresented in HNS repressed genes. In contrary, nGCO genes and genes with no homolog in *E. coli *are overrepresented in HNS repressed genes. As expected, we observed that genes with no homolog in *E. coli *are underrepresented in the essential gene set. In the random dataset we observe the same representation as in the overall *Salmonella *genome. The same coloring scheme with Fig. 1B. is used for easy comparison.

Further examination showed that, of the 231 experimentally verified *Salmonella *genes that were found to be essential in a recent study [28], 184 were GCO (79.6% compared to 68.3% for the overall genome representation), 27 were nGCO (almost equal representation 11.7% compared to 12.6%) and 20 were without an *E. coli *K12 homolog (8.6% compared to 19.0%, see also Fig. 3). To support our finding, we generated 1000 random sets of 231 genes (sampled without replacement from the *Salmonella *genome) and examined the distributions of genes belonging to the three different classes: 68.4%, 12.6%, 18.9% respectively, very close to the values for the complete gene set (Fig. 3). The 211 essential genes with an *E. coli *K12 homolog encode for proteins that exhibit average sequence identity of 82.5% (sd = 19.8). These results show that essential genes in bacteria are not only more evolutionary conserved than non essential ones, as was previously shown [6], but they also tend to be GCO, at least in *Salmonella*.

### H-NS preferentially regulates genes that do not conserve their order

Driven by recent results demonstrating that H-NS silences a significant amount of genes thought to have been acquired by lateral gene transfer, we calculated the number of GCO and nGCO genes in the dataset of 359 genes that were found to be silenced by the action of H-NS in the *Salmonella *chromosome [13]. We ended up with the following numbers: 119 genes were GCO (33.1%), 99 (27.6%) were nGCO and 141 (39.3%) were without any detectable homolog in *E. coli *K12. The difference in the gene order status of genes compared to the overall genome is striking, with the representation being 68.3%, 12.6% and 19% respectively (see also fig. 3). This result demonstrates a preference of H-NS to repress genes that are either nGCO or that have no homologs in closely related species. The 119 GCO genes shared 74.7% identity and the nGCO 34.2% (P-value < 2.2e-16), a finding consistent with the results for both the overall genome set and the set of duplicated genes (Fig. 1A).

We calculated the GC content for the H-NS repressed GCO genes (49.4%), nGCO genes (45.5%) and genes with no detected homolog in *E. coli *K12 (44.0%), which is a significant decrease of the GC content for all three different groups of genes (P-value < 5.498e-12) (Fig. 1B). Using a list of putative HTGs [22] we identified 130 genes out of the 359 H-NS repressed *Salmonella *genes that were predicted to be horizontally transferred. Although there are many genes from the 359 which are predicted to be HTGs - as previously reported [13] - there is still a large number of H-NS repressed genes that were not predicted to be horizontally transferred. An interesting finding was that in the set of 229 H-NS repressed genes (not predicted to be HTGs) presented a higher GC content compared to the 130 H-NS repressed genes predicted to be HTGs (Table 1 and Additional file 1). Someone may argue that these results may be biased due to the method followed in [22]. However, we observed a similar pattern of decrease in GC content compared to the one calculated for the complete *Salmonella *gene set (Table 1).

**Table 1:** Gene order conservation of HNS repressed genes not predicted to be HTGs is associated to their GC content.

	HNSnonHTGs	
***Gene order status***	***Number of Genes (%)***	***Average %GC content (sd)***

GCO	93 (40.6)	51.2 (4.8)

nGCO	54 (23.6)	47.6 (5.3)

No *E. coli *K12 homolog	82 (35.8)	45.4 (6.7)

We illustrate features of the subset of 229 HNS repressed genes predicted not to be HTGs (HNSnonHTGs). More specifically, the distribution of HNSnonHGTs genes in the three categories depending on gene order conservation largely deviates from the overall genome representation. GCO genes are still underrepresented but significantly increased compared to predicted HTGs (see additional file 10). However, we observe the same characteristic decrease in the average GC content between these gene sets with the overall genome (c.f. Fig. 1B) (KruskalWallis chisquared = 37.8234, df = 2, Pvalue < 6.12e09).

In order to clarify whether the observed differences are due to pathogenicity related genes, we also compared *Salmonella *with an enteropathogenic strain of *E. coli *(O127:H6 str. E2348/69) (EPEC) [29]ftp://ftp.ncbi.nih.gov/genomes/Bacteria/. We identified the complement of genes that had no homolog between *Salmonella *and *E. coli *K12 and had a homolog between *Salmonella *and EPEC (Additional file 5). Among the H-NS repressed genes within this dataset (14 genes out of 174 in total), we detected genes of *Salmonella *Pathogenicity Island-2 (SPI-2) such as *ssaL*, *S *[30], *sseB *and *sscA *[31]. We also identified *Salmonella *genes whose order switches from nGCO to GCO when the reference genome is K12 and EPEC, respectively (Additional file 6). In this dataset, the H-NS repressed genes (11 genes out of 55 in total) include genes of SPI-2 such as ssaV, N, R, T, U [30].

Another interesting observation in the 359 H-NS repressed genes was the identification of 98 duplicated genes within this dataset (27.3% compared to 15.5% of duplicated genes in the whole genome) out of which only 29 were GCO. We subsequently analyzed the GC content for the duplicated genes of which only one paralog is targeted by H-NS. Of the 98 duplicated genes, a subset of 74 (out of which 22 were GCO) were identified to have 89 paralogs which are not H-NS repressed. After comparing the GC content of the above subsets of paralogs, we observed a striking difference. More specifically, the GC content of the 74 H-NS repressed genes was 46.5% (sd = 5.5) and for their 89 paralogs the GC content was 51.4% (sd = 4.2) (Wilcoxon rank-sum test: W = 1545, P-value = 5.663e-09). The above results provide a strong indication that, apart from repressing horizontally transferred genes, H-NS may also play a role in the transcriptional regulation of duplicated genes. Interestingly, genes that play an important role in the pathogenicity of *Salmonella *such as ssrB (essential for the expression of SPI-2 genes [32] genes) and ssaN, R, T, U and V (genes of SPI-2 that encode components of the second type III secretion apparatus [30]) were found to be nGCO, with low GC content (43.1 average GC%) whereas their corresponding duplicates were GCO with significantly higher GC content (54.79 average GC%). Moreover, the aforementioned virulence associated genes were identified to be repressed by H-NS [13], whereas their duplicates' expression was not significantly affected by H-NS.

Surprisingly, 20 genes out of those 359 were found to be essential for *Salmonella*. This paradox may be explained either by the growth conditions under which experiments where carried out [28] or by introducing H-NS as a part of a general, yet currently unknown, bacterial regulatory mechanism.

## Discussion

Taken together, this work shows that in *Salmonella*: (a) the proteins encoded by GCO genes tend to be more conserved (Fig. 1A and Fig. 2). Probably this is due to a correlation of conservation of gene order and the number of interacting proteins and/or expression levels of proteins encoded by genes belonging to the conserved clusters. Although it is known that protein expression levels are correlated to protein evolutionary rates [8,9], and it is rather controversial whether there is a correlation between the connectivity of nodes in protein interaction networks and evolutionary rate [2,7,33-35], it still remains to be shown if any of these findings correlate to gene order conservation as well. (b) GCO genes have on average higher GC content when compared to genes that are nGCO and even higher than genes that do not share any homology to *E. coli *K12 genes (Fig. 1B). We speculate that this is a result of a mechanism that leads genes which evolve faster than others (and are not under strong selective pressure) to accumulate AT-rich mutations. (c) Genes that remain GCO have on average significantly lower Ka/Ks ratio than the nGCO ones, indicating that they are under higher selective pressure (Fig. 2). (d) Whenever a duplication event takes place, the duplicate that keeps its original order, is the one that keeps the highest sequence similarity (at least on average) and possibly the original function (in agreement to [11]). On the other hand, when the duplicated gene does not preserve its original order, it is subject to much faster evolution and less selective pressure as it is shown by comparing the respective Ka/Ks ratios (Fig. 2). Overrepresentation of nGCO genes in the duplicated group demonstrates that selective pressure on genes to keep their order is more relaxed after a duplication event. Interestingly, after a duplication event occurs and none of the paralogs retain their ancestral position in the genome, we failed to find any preference for sequence conservation (Additional file 4). (e) Finally, we demonstrated that the silencing mechanism via the H-NS protein in *Salmonella *significantly affects genes that are nGCO or genes that do not have any (detectable) homolog in *E. coli *K12. This could be another indication that H-NS might play a role, not only as a defensive instrument against foreign acquired DNA [12,13,36] but also as an internal control of native genes [15], as it could be of benefit to the organism to have them repressed for a variety of reasons associated with genome structure, for duplicated genes, non-essential genes or genes that did not retain their order.

## Conclusions

In our analysis we tried to correlate gene order conservation with gene duplication, GC content and gene silencing in *Salmonella enterica *serovar Typhimurium LT2 based on two publications concerning H-NS silencing in the aforementioned species. We found that genes of conserved order not only evolve slower, as previously reported, but also tend to have higher GC content than the ones that do not conserve their order. We also found that essential genes tend to retain their genome position. Another finding was that after a duplication event takes place the gene that keeps its original position evolves slower than the one that looses it and probably keeps its primary function. Our study also shows that H-NS protein, a general repressor of a large number of genes in *Salmonella*, tends to avoid binding on genes with a conserved order. There is a clear indication that from the duplicated genes H-NS "prefers" to repress those ones that do not have a conserved gene order.

## Methods

### Definition of genes of conserved order

As a measure of conservation at the amino acid sequence level, we used the percentage of identical residues reported by BLASTP, while disregarding conservative substitutions and gaps. We follow the same criteria of gene order conservation as in [2]. More specifically, given two genomes A and B encoding N_a _and N_b _genes respectively, let us assume these genes are ordered a_1_, a_2_, ..., a_Na _and b_1_, b_2_, ..., b_Nb_, respectively. Then, two genes a_i _and b_j _(1<i<N_a_, 1<j<N_b_) are defined as GCO when the following two conditions are met: (1) a_i_, b_j _exhibit statistically significant sequence similarity; (2) at least one of a_i-1_, a_i+1 _exhibits statistically significant sequence similarity with one of b_j-1_, b_j+1_.

### Genome comparison

We applied a relatively strict E-value cut-off (E ≤ 10^-6^) and an additional requirement of 50% length coverage of both query and target sequences by the BLAST alignment. This approach was chosen in order to avoid spurious matches due to short conserved sequence motifs. The same criteria were applied both in the cross-genome comparison and in the self- comparison as presented in text. Differences from the results reported in [2] originate from the fact that we have used the latest versions of the *E. coli *K12 and *Salmonella *genomes, the difference in the E-value cut-off and the introduction of the alignment coverage criterion in this work.

### Ka/Ks estimation

We used the pairwise alignments reported by BLASTP to guide the construction of alignments of the corresponding coding regions, inserting in-frame gaps where necessary. For the Ka/Ks ratio calculation we used the yn00 program of the PAML package [25,26] with the following parameters:

noisy = 1

icode = 10 * 0:universal code; 1:mammalian mt; 2-10:see below

weighting = 0 * weighting pathways between codons (0/1)?

commonf3 × 4 = 1 * use one set of codon freqs for all pairs (0/1)?

We have removed two genes (NP_462323 and NP_463455) for which the program couldn't provide us with a reliable Ka/Ks ratio, as well as all genes with Ka/Ks = 0 (Additional file 7). In order to avoid probable cases of saturated Ks values, where the estimated Ka/Ks ratio may be unreliable, we have further filtered our dataset to contain only those homologs with Ks<1.5 according to [37]. Nevertheless, since sequences of nGCO genes on average seem to accumulate more non-synonymous substitutions than those of GCO genes, the difference in the Ka/Ks ratio between GCO and nGCO genes would probably be higher than the one reported here (Additional file 8, 9, 10.

### Statistical analysis

All statistical calculations have been performed with R (Version 2.3.1) R Development Core Team. R: A language and environment for statistical computing. R Foundation for Statistical Computing, Vienna, Austria, 2004. URL http://www.R-project.org. 3-900051-07-0.

## Competing interests

The authors declare that they have no competing interests.

## Authors' contributions

II and VJP conceived and designed the experiments. NP, KT and II collected, cleansed and reformatted data. NP, KT, VJP and II performed the experiments. TT performed Statistical Analysis. II and VJP Wrote the paper. All authors read and approved the final manuscript.

## Supplementary Material

Additional file 1 **List of *Salmonella *and *E. coli *homologs**. A table containing information about *Salmonella *and *E*. *coli *homologs. Click here for file

Additional file 2 **GC content analysis of the *Salmonella *gene set after removing predicted HTGs**. Table displaying the content analysis of the *Salmonella *gene set after removing predicted HTGs. Click here for file

Additional file 3 **Duplicates in *Salmonella *that share the same best hit in *E. coli *K12**. Table displaying in *Salmonella *that share the same best hit in *E. coli *K12. Click here for file

Additional file 4 **List of *Salmonella *nGCO duplicated genes**. Table displaying of *Salmonella *nGCO duplicated genes. Click here for file

Additional file 5 **Genes with no homolog between *Salmonella *and *E. coli *k12 that share a homolog between *Salmonella *and EPEC**. Table displaying Genes with no homolog between *Salmonella *and *E. coli *k12 that share a homolog between *Salmonella *and EPEC. Click here for file

Additional file 6 ***Salmonella *genes whose order switches from nGCO to GCO when the reference genome is K12 and EPEC, respectively**. Table displaying *Salmonella *genes whose order switches from nGCO to GCO when the reference genome is K12 and EPEC, respectively. Click here for file

Additional file 7 ***Salmonella *genes that were excluded from Ka/Ks ratio calculations**. Table displaying *Salmonella *genes that were excluded from Ka/Ks ratio calculations. Click here for file

Additional file 8 **Gene order conservation of H-NS repressed genes predicted to be HTGs is not associated to their GC content**. We illustrate features of the subset of 130 H-NS repressed genes predicted to be HTGs (HNS-HTGs). More specifically, the distribution of HNS-HTGs genes in the three categories depending on gene order conservation largely deviates from the overall genome representation, with a striking under-representation of GCO genes. Additionally, the average GC content between these gene sets does not exhibit significant differences among the three gene classes (Kruskal-Wallis chi-squared = 1.1224, df = 2, P-value < 0.7717). This finding was expected since the prediction of the HTGs is mainly based on GC content (Garcia-Vallve S, Guzman E, Montero MA, Romeu A: HGT-DB: a database of putative horizontally transferred genes in prokaryotic complete genomes. *Nucleic Acids Res *2003, 31(1):187-189.) Click here for file

Additional file 9 **Correlation of gene order conservation with poisson and gamma corrected distances for multiple substitutions**. Poisson and gamma corrected distances for multiple substitutions were calculated as described in Yang Z., "Computational Molecular Evolution", Oxford University Press, 2006 (pp. 45 and 46 respectively). Click here for file

Additional file 10 **A detailed legend of Fig**. 1**including statistical analysis**. A Word DOC containing a full legend for figure 1. Click here for file
