# Supplementary material for: Gene socialization: gene order, GC content and gene silencing in Salmonella
Source: BMC Genomics. 2009 Dec 11;10:597. doi: 10.1186/1471-2164-10-597 (PMC2801525; doi:10.1186/1471-2164-10-597)
Supplement: Additional file 1 — List of Salmonella and E. coli homologs. A table containing information about Salmonella and E. coli homologs. [file 1471-2164-10-597-S1.DOC]

| **Salmonela** | **name** | **Salmonela gene description** | **hns** | **k12** | **name** | **K12 gene description** | **GCO** | **Identity** | **GC content** |
| --- | --- | --- | --- | --- | --- | --- | --- | --- | --- |
| NP_459006 | thrL | thr operon leader peptide | - |  |  |  | NO HOMOLOG |  | 54.54% |
| NP_459007 | thrA | bifunctional aspartokinase I/homeserine dehydrogenase I | - | NP_414543 | thrA | bifunctional aspartokinase I/homeserine dehydrogenase I | GCO | 92.80% | 55.94% |
| NP_459008 | thrB | homoserine kinase | - | NP_414544 | thrB | homoserine kinase | GCO | 84.41% | 57.95% |
| NP_459009 | thrC | threonine synthase | - | NP_414545 | thrC | threonine synthase | GCO | 89.95% | 56.79% |
| NP_459010 | yaaA | hypothetical protein | - | NP_414547 | yaaA | hypothetical protein | GCO | 86.38% | 50.64% |
| NP_459011 | yaaJ | putative alanine/glycine transport protein | - | NP_414548 | yaaJ | predicted transporter | GCO | 70.16% | 55.83% |
| NP_459012 | talB | transaldolase | - | NP_414549 | talB | transaldolase B | GCO | 94.63% | 53.98% |
| NP_459013 | mogA | molybdenum cofactor biosynthesis protein | - | NP_414550 | mog | molybdenum cofactor biosynthesis protein | GCO | 91.66% | 56.17% |
| NP_459014 | yaaH | putative regulatory protein | - | NP_414551 | yaaH | conserved inner membrane protein associated with acetate transport | GCO | 90.42% | 54.14% |
| NP_459015 | htgA | positive regulator | - | NP_414552 | yaaW | hypothetical protein | GCO | 78.81% | 54.34% |
| NP_459016 | yaaI | putative periplasmic protein | - | NP_414554 | yaaI | hypothetical protein | GCO | 81.34% | 49.62% |
| NP_459017 | dnaK | molecular chaperone DnaK | - | NP_414555 | dnaK | molecular chaperone DnaK | GCO | 95.17% | 52.58% |
| NP_459018 | dnaJ | heat shock protein | - | NP_414556 | dnaJ | chaperone Hsp40, co-chaperone with DnaK | GCO | 76.25% | 54.03% |
| NP_459019 | - | putative transcriptional regulator | + | NP_415136 | ybdO | predicted DNA-binding transcriptional regulator | nGCO | 24.52% | 40.61% |
| NP_459020 | - | putative bacteriophage protein | - |  |  |  | NO HOMOLOG |  | 40.57% |
| NP_459021 | - | hypothetical protein | - |  |  |  | NO HOMOLOG |  | 51.12% |
| NP_459022 | - | hypothetical protein | + |  |  |  | NO HOMOLOG |  | 38.28% |
| NP_459023 | - | putative exochitinase | - |  |  |  | NO HOMOLOG |  | 51.09% |
| NP_459024 | - | putative hydroxymethyltransferase | - |  |  |  | NO HOMOLOG |  | 54.85% |
| NP_459025 | - | putative cytoplasmic protein | - | NP_414909 | yaiV | predicted DNA-binding transcriptional regulator | nGCO | 33.33% | 46.95% |
| NP_459026 | bcfA | fimbrial subunit | + | NP_415458 | ycbQ | predicted fimbrial-like adhesin protein | nGCO | 35% | 51.74% |
| NP_459027 | bcfB | fimbrial chaparone | - | NP_415064 | sfmC | pilin chaperone, periplasmic | nGCO | 51% | 50.94% |
| NP_459028 | bcfC | fimbrial usher | - | NP_415460 | ycbS | predicted outer membrane usher protein | nGCO | 52.01% | 54.46% |
| NP_459029 | bcfD | fimbrial subunit | - | NP_415066 | sfmH | predicted fimbrial-like adhesin protein | nGCO | 43.96% | 50.09% |
| NP_459030 | bcfE | fimbrial subunit | - | NP_415462 | ycbU | predicted fimbrial-like adhesin protein | GCO | 41.55% | 49.45% |
| NP_459031 | bcfF | fimbrial subunit | - | NP_415463 | ycbV | predicted fimbrial-like adhesin protein | GCO | 41.83% | 55.68% |
| NP_459032 | bcfG | fimbrial chaparone | - | NP_415464 | ycbF | predicted periplasmic pilini chaperone | GCO | 44.23% | 53.96% |
| NP_459033 | bcfH | putative thiol-disulfide isomerase | - |  |  |  | NO HOMOLOG |  | 54.96% |
| NP_945153 | - | hypothetical protein | - |  |  |  | NO HOMOLOG |  | 41.81% |
| NP_459034 | - | putative transcriptional regulator | - |  |  |  | NO HOMOLOG |  | 37.11% |
| NP_459035 | - | putative transcriptional regulator | - | NP_415136 | ybdO | predicted DNA-binding transcriptional regulator | nGCO | 20.77% | 38.80% |
| NP_459036 | - | putative transcriptional regulator | - |  |  |  | NO HOMOLOG |  | 35.82% |
| NP_459037 | - | putative arylsulfatase | - |  |  |  | NO HOMOLOG |  | 46.30% |
| NP_459038 | - | putative 5'-nucleotidase | + | NP_415013 | ushA | UDP-sugar hydrolase | nGCO | 24.48% | 46.81% |
| NP_459039 | - | putative outer membrane/exported protein | - | NP_416903 | yfeN | conserved outer membrane protein | nGCO | 34.71% | 44.22% |
| NP_459040 | - | putative arylsulfatase | + | NP_416015 | ydeN | hypothetical protein | nGCO | 25.13% | 52.40% |
| NP_459041 | - | putative arylsulfatase regulator | - | YP_026259 | aslB | predicted regulator of arylsulfatase activity | nGCO | 46.32% | 49.70% |
| NP_459042 | - | putative cytoplasmic protein | - |  |  |  | NO HOMOLOG |  | 49.92% |
| NP_459043 | - | putative arylsulfatase | - |  |  |  | NO HOMOLOG |  | 49.70% |
| NP_459044 | nhaA | Na+/H antiporter | - | NP_414560 | nhaA | pH-dependent sodium/proton antiporter | GCO | 80.72% | 56.38% |
| NP_459045 | nhaR | transcriptional activator | - | NP_414561 | nhaR | DNA-binding transcriptional activator | GCO | 89.52% | 50.55% |
| NP_459046 | - | putative glycosyl hydrolase | + | NP_418113 | yicI | predicted alpha-glucosidase | nGCO | 25.33% | 54.60% |
| NP_459047 | - | putative sodium galactoside symporter | + | NP_418313 | yihP | predicted transporter | nGCO | 26.74% | 49.05% |
| NP_459048 | rpsT | 30S ribosomal protein S20 | - | NP_414564 | rpsT | 30S ribosomal protein S20 | GCO | 97.70% | 46.59% |
| NP_459049 | yaaY | putative cytoplasmic protein | - | NP_414565 | yaaY | hypothetical protein | GCO | 71.42% | 47.68% |
| NP_459050 | ribF | hypothetical protein | - | NP_414566 | ribF | hypothetical protein | GCO | 89.96% | 56.76% |
| NP_459051 | ileS | isoleucyl-tRNA synthetase | - | NP_414567 | ileS | isoleucyl-tRNA synthetase | GCO | 87.08% | 54.77% |
| NP_459052 | lspA | signal peptidase II | - | NP_414568 | lspA | signal peptidase II | GCO | 84.75% | 52.89% |
| NP_459053 | slpA | FKBP-type peptidyl-prolyl cis-trans isomerase | - | NP_414569 | fkpB | FKBP-type peptidyl-prolyl cis-trans isomerase (rotamase) | GCO | 83.89% | 54.66% |
| NP_459054 | lytB | 4-hydroxy-3-methylbut-2-enyl diphosphate reductase | - | NP_414570 | ispH | 4-hydroxy-3-methylbut-2-enyl diphosphate reductase | GCO | 93.67% | 57.41% |
| NP_459055 | - | putative nitrite reductase | - |  |  |  | NO HOMOLOG |  | 48.21% |
| NP_459056 | rihC | putative purine nucleoside hydrolase | - | NP_414571 | rihC | ribonucleoside hydrolase 3 | nGCO | 77.07% | 56.56% |
| NP_459057 | - | putative transcriptional regulator | - | NP_415153 | citB | DNA-binding response regulator in two-component regulatory system with citA | GCO | 47.29% | 48.61% |
| NP_459058 | - | putative transcriptional regulator | - | NP_415152 | citA | sensory histidine kinase in two-component regulatory system with citB | GCO | 39.39% | 48.02% |
| NP_459059 | - | putative oxalacetate decarboxylase subunit beta | - |  |  |  | NO HOMOLOG |  | 63.97% |
| NP_459060 | - | oxaloacetate decarboxylase | - |  |  |  | NO HOMOLOG |  | 63.68% |
| NP_459061 | - | putative oxaloacetate decarboxylase subunit gamma | - |  |  |  | NO HOMOLOG |  | 57.50% |
| NP_459062 | - | putative citrate-sodium symporter | - |  |  |  | NO HOMOLOG |  | 49.36% |
| NP_459063 | citC2 | putative citrate lyase synthetase | - | NP_415151 | citC | citrate lyase synthetase | GCO | 46.38% | 53.73% |
| NP_459064 | citD2 | putative citrate lyase acyl carrier protein gamma chain | - | NP_415150 | citD | citrate lyase, acyl carrier (gamma) subunit | GCO | 51.35% | 57.14% |
| NP_459065 | citE2 | putative citrate lyase beta chain | - | NP_415149 | citE | citrate lyase, citryl-ACP lyase (beta) subunit | GCO | 60.34% | 55.63% |
| NP_459066 | citF2 | putative citrate lyase alpha chain/citrate-ACP transferase | - | NP_415148 | citF | citrate lyase, citrate-ACP transferase (alpha) subunit | GCO | 72.29% | 55.22% |
| NP_459067 | citX2 | putative cytoplasmic protein | - | NP_415147 | citX | 2'-(5'-triphosphoribosyl)-3'-dephospho-CoA:apo-citrate lyase | GCO | 40.88% | 55.97% |
| NP_459068 | citG2 | triphosphoribosyl-dephospho-CoA synthase | - | NP_415146 | citG | triphosphoribosyl-dephospho-CoA transferase | GCO | 44.75% | 54.78% |
| NP_459069 | dapB | dihydrodipicolinate reductase | - | NP_414572 | dapB | dihydrodipicolinate reductase | GCO | 90.10% | 56.56% |
| NP_459071 | carA | carbamoyl-phosphate synthase small subunit | - | NP_414573 | carA | carbamoyl-phosphate synthase small subunit | GCO | 94.76% | 55.35% |
| NP_459072 | carB | carbamoyl-phosphate synthase large subunit | - | NP_414574 | carB | carbamoyl-phosphate synthase large subunit | GCO | 98.22% | 59.10% |
| NP_459073 | caiF | of cai/fix operon transcriptional regulator | - | NP_414576 | caiF | DNA-binding transcriptional activator | GCO | 76.33% | 46.46% |
| NP_459074 | caiE | carnitine racemase stimulation factor | - | NP_414577 | caiE | predicted acyl transferase | GCO | 85.12% | 55.94% |
| NP_459075 | caiD | carnitinyl-CoA dehydratase | - | NP_414578 | caiD | carnitinyl-CoA dehydratase | GCO | 88.12% | 56.87% |
| NP_459076 | caiC | crotonobetaine/carnitine-CoA ligase | - | NP_414579 | caiC | crotonobetaine/carnitine-CoA ligase | GCO | 84.91% | 50.77% |
| NP_459077 | caiB | crotonobetainyl-CoA:carnitineCoA-transferase | - | NP_414580 | caiB | crotonobetainyl-CoA:carnitineCoA-transferase | GCO | 92.59% | 53.20% |
| NP_459078 | caiA | crotonobetainyl-CoA dehydrogenase | - | NP_414581 | caiA | crotonobetaine reductase subunit II, FAD-binding | GCO | 97.63% | 52.58% |
| NP_459079 | caiT | L-carnitine/gamma-butyrobetaine antiporter | - | NP_414582 | caiT | L-carnitine/gamma-butyrobetaine antiporter | GCO | 93.45% | 54.08% |
| NP_459080 | fixA | putative electron transfer flavoprotein subunit beta | - | NP_414583 | fixA | predicted electron transfer flavoprotein subunit, ETFP adenine nucleotide-binding domain | GCO | 82.42% | 52.01% |
| NP_459081 | fixB | putative electron transfer flavoprotein subunit alpha | - | NP_414584 | fixB | predicted electron transfer flavoprotein, NAD/FAD-binding domain and ETFP adenine nucleotide-binding domain-like | GCO | 76.67% | 58.91% |
| NP_459082 | fixC | flavoprotein | - | NP_414585 | fixC | predicted oxidoreductase with FAD/NAD(P)-binding domain | GCO | 84.34% | 55.47% |
| NP_459083 | fixX | putative ferredoxin | - | NP_414586 | fixX | predicted 4Fe-4S ferredoxin-type protein | GCO | 88.42% | 54.51% |
| NP_459084 | yaaU | putative transport protein | - | NP_414587 | yaaU | predicted transporter | GCO | 82.00% | 54.13% |
| NP_459085 | - | putative outer membrane lipoprotein | - | NP_417289 | ygdI | hypothetical protein | nGCO | 60.71% | 48.48% |
| NP_459086 | - | putative secreted protein | - |  |  |  | NO HOMOLOG |  | 47.00% |
| NP_459087 | - | putative secreted protein | + | NP_417705 | yhcN | hypothetical protein | nGCO | 35.95% | 46.39% |
| NP_459089 | - | putative sulfatase | + | NP_418134 | yidJ | predicted sulfatase/phosphatase | nGCO | 22.55% | 46.19% |
| NP_459090 | yabF | glutathione-regulated potassium-efflux system ancillary protein | - | NP_414588 | kefF | glutathione-regulated potassium-efflux system ancillary protein | GCO | 87.50% | 53.67% |
| NP_459091 | kefC | glutathione-regulated potassium-efflux system protein | - | NP_414589 | kefC | glutathione-regulated potassium-efflux system protein | GCO | 80.93% | 58.18% |
| NP_459092 | folA | dihydrofolate reductase type I | - | NP_414590 | folA | dihydrofolate reductase | GCO | 96.85% | 53.75% |
| NP_459093 | apaH | diadenosinetetraphosphatase | - | NP_414591 | apaH | diadenosinetetraphosphatase | GCO | 93.18% | 56.30% |
| NP_459094 | apaG | hypothetical protein | - | NP_414592 | apaG | hypothetical protein | GCO | 85.60% | 55.02% |
| NP_459095 | ksgA | dimethyladenosine transferase | - | NP_414593 | ksgA | dimethyladenosine transferase | GCO | 94.50% | 52.43% |
| NP_459096 | pdxA | 4-hydroxythreonine-4-phosphate dehydrogenase | - | NP_414594 | pdxA | 4-hydroxythreonine-4-phosphate dehydrogenase | GCO | 89.66% | 58.68% |
| NP_459097 | surA | peptidyl-prolyl cis-trans isomerase | - | NP_414595 | surA | peptidyl-prolyl cis-trans isomerase (PPIase) | GCO | 91.35% | 54.39% |
| NP_459098 | imp | organic solvent tolerance protein precursor | - | NP_414596 | imp | organic solvent tolerance protein precursor | GCO | 91.09% | 52.77% |
| NP_459099 | djlA | Dna-J like membrane chaperone protein | - | NP_414597 | djlA | Dna-J like membrane chaperone protein | GCO | 85.60% | 54.12% |
| NP_459100 | rluA | 23S rRNA pseudouridylate 746 synthase | - | NP_414600 | rluA | pseudouridine synthase for 23S rRNA (position 746) and tRNAphe(position 32) | GCO | 95.43% | 57.12% |
| NP_459101 | hepA | ATP-dependent helicase HepA | - | NP_414601 | hepA | ATP-dependent helicase HepA | GCO | 93.07% | 57.00% |
| NP_459102 | polB | DNA polymerase II | - | NP_414602 | polB | DNA polymerase II | GCO | 89.91% | 56.50% |
| NP_459103 | - | putative secreted protein | - |  |  |  | NO HOMOLOG |  | 49.43% |
| NP_459105 | - | putative cytoplasmic protein | + |  |  |  | NO HOMOLOG |  | 42.61% |
| NP_459106 | araD | L-ribulose-5-phosphate 4-epimerase | - | NP_414603 | araD | L-ribulose-5-phosphate 4-epimerase | GCO | 91.77% | 55.02% |
| NP_459107 | araA | L-arabinose isomerase | - | NP_414604 | araA | L-arabinose isomerase | GCO | 95.60% | 55.82% |
| NP_459108 | araB | ribulokinase | - | NP_414605 | araB | ribulokinase | GCO | 88.02% | 60.87% |
| NP_459109 | araC | arabinose operon transcriptional regulator | - | NP_414606 | araC | DNA-binding transcriptional dual regulator | GCO | 88.25% | 53.90% |
| NP_459110 | yabI | hypothetical protein | - | NP_414607 | yabI | conserved inner membrane protein | GCO | 78.34% | 57.42% |
| NP_459111 | yabJ | putative ABC transporter protein | - | NP_414608 | thiQ | thiamin transporter subunit | GCO | 84.25% | 58.05% |
| NP_459112 | thiP | thiamin ABC transporter membrane component | - | NP_414609 | thiP | thiamin ABC transporter membrane protein | GCO | 84.32% | 59.65% |
| NP_459113 | tbpA | thiamine-binding periplasmic protein | - | NP_414610 | tbpA | thiamin transporter subunit | GCO | 88.59% | 54.97% |
| NP_459114 | yabN | putative periplasmic binding protein | - | NP_414611 | sgrR | DNA-binding transcriptional regulator | GCO | 85.86% | 56.66% |
| NP_459115 | leuD | isopropylmalate isomerase small subunit | - | NP_414613 | leuD | isopropylmalate isomerase small subunit | GCO | 92% | 52.97% |
| NP_459116 | leuC | isopropylmalate isomerase large subunit | - | NP_414614 | leuC | isopropylmalate isomerase large subunit | GCO | 93.13% | 58.95% |
| NP_459117 | leuB | 3-isopropylmalate dehydrogenase | - | NP_414615 | leuB | 3-isopropylmalate dehydrogenase | GCO | 91.46% | 55.95% |
| NP_459118 | leuA | 2-isopropylmalate synthase | - | NP_414616 | leuA | 2-isopropylmalate synthase | GCO | 92.92% | 54.83% |
| NP_459119 | leuL | leu operon leader peptide | - |  |  |  | NO HOMOLOG |  | 47.12% |
| NP_459120 | leuO | leucine transcriptional activator | + | NP_414618 | leuO | leucine transcriptional activator | GCO | 84.39% | 48.04% |
| NP_459121 | ilvI | acetolactate synthase III large subunit | - | YP_025294 | ilvI | acetolactate synthase III large subunit | GCO | 89.51% | 54.69% |
| NP_459122 | ilvH | acetolactate synthase small subunit | - | NP_414620 | ilvH | acetolactate synthase small subunit | GCO | 97.54% | 53.86% |
| NP_459123 | fruR | fru operon transcriptional repressor | - | NP_414622 | fruR | DNA-binding transcriptional dual regulator | GCO | 98.80% | 56.41% |
| NP_459124 | yabB | hypothetical protein | - | NP_414623 | mraZ | hypothetical protein | GCO | 94.07% | 51.85% |
| NP_459125 | mraW | S-adenosyl-methyltransferase | - | NP_414624 | mraW | S-adenosyl-methyltransferase | GCO | 95.84% | 56.26% |
| NP_459126 | ftsL | cell division protein | - | NP_414625 | ftsL | membrane bound cell division protein at septum containing leucine zipper motif | GCO | 83.47% | 51.91% |
| NP_459127 | ftsI | division specific transpeptidase | - | NP_414626 | ftsI | transpeptidase involved in septal peptidoglycan synthesis (penicillin-binding protein 3) | GCO | 96.25% | 54.83% |
| NP_459128 | murE | UDP-N-acetylmuramoylalanyl-D-glutamate--2,6-diaminopimelate ligase | - | NP_414627 | murE | UDP-N-acetylmuramoylalanyl-D-glutamate--2,6-diaminopimelate ligase | GCO | 84.24% | 58.73% |
| NP_459129 | murF | D-alanine-D-alanine ligase | - | NP_414628 | murF | UDP-N-acetylmuramoyl-tripeptide:D-alanyl-D-alanine ligase | GCO | 79.42% | 56.36% |
| NP_459130 | mraY | phospho-N-acetylmuramoyl-pentapeptide-transferase | - | NP_414629 | mraY | phospho-N-acetylmuramoyl-pentapeptide-transferase | GCO | 92.50% | 53.92% |
| NP_459131 | murD | UDP-N-acetylmuramoyl-L-alanyl-D-glutamate synthetase | - | NP_414630 | murD | UDP-N-acetylmuramoyl-L-alanyl-D-glutamate synthetase | GCO | 81.27% | 58.16% |
| NP_459132 | ftsW | essential cell division gene | - | NP_414631 | ftsW | integral membrane protein involved in stabilizing FstZ ring during cell division | GCO | 81.03% | 54.53% |
| NP_459133 | murG | N-acetylglucosaminyl transferase | - | NP_414632 | murG | N-acetylglucosaminyl transferase | GCO | 92.09% | 60.86% |
| NP_459134 | murC | UDP-N-acetylmuramate--L-alanine ligase | - | NP_414633 | murC | UDP-N-acetylmuramate--L-alanine ligase | GCO | 87.55% | 56.43% |
| NP_459135 | ddl | D-alanylalanine synthetase | - | NP_414634 | ddlB | D-alanylalanine synthetase | GCO | 90.52% | 54.18% |
| NP_459136 | ftsQ | cell division protein | - | NP_414635 | ftsQ | membrane anchored protein involved in growth of wall at septum | GCO | 89.31% | 52.58% |
| NP_459137 | ftsA | cell division protein | - | NP_414636 | ftsA | cell division protein | GCO | 95% | 53.60% |
| NP_459138 | ftsZ | cell division protein FtsZ | - | NP_414637 | ftsZ | cell division protein FtsZ | GCO | 86.16% | 56.77% |
| NP_459139 | lpxC | UDP-3-O-[3-hydroxymyristoyl] N-acetylglucosamine deacetylase | - | NP_414638 | lpxC | UDP-3-O-[3-hydroxymyristoyl] N-acetylglucosamine deacetylase | GCO | 98.36% | 50.43% |
| NP_459140 | yacA | SecA regulator SecM | - | NP_414639 | secM | SecA regulator SecM | GCO | 69.38% | 58.04% |
| NP_459141 | secA | translocase | - | NP_414640 | secA | translocase | GCO | 92.22% | 53.88% |
| NP_459142 | mutT | 7,8-dihydro-8-oxoguanine-triphosphatase | - | NP_414641 | mutT | nucleoside triphosphate pyrophosphohydrolase, marked preference for dGTP | GCO | 80.46% | 52.27% |
| NP_459143 | yacG | zinc-binding protein | - | NP_414643 | yacG | zinc-binding protein | GCO | 89.36% | 54.68% |
| NP_459144 | yacF | hypothetical protein | - | NP_414644 | yacF | hypothetical protein | GCO | 89.47% | 52.95% |
| NP_459145 | coaE | dephospho-CoA kinase | - | NP_414645 | coaE | dephospho-CoA kinase | GCO | 84.95% | 54.91% |
| NP_459146 | guaC | guanosine 5'-monophosphate oxidoreductase | - | NP_414646 | guaC | guanosine 5'-monophosphate oxidoreductase | GCO | 91.35% | 53.16% |
| NP_459147 | hofC | putative type IV pilin assembly protein | - | NP_414648 | hofC | assembly protein in type IV pilin biogenesis, transmembrane protein | GCO | 63.50% | 54.44% |
| NP_459148 | hofB | putative type IV fimbrial biosynthetic protein | - | NP_414649 | hofB | conserved protein with nucleoside triphosphate hydrolase domain | GCO | 69.93% | 55.48% |
| NP_459149 | ppdD | putative type IV pilin major component | - | NP_414650 | ppdD | predicted major pilin subunit | GCO | 81.11% | 53.65% |
| NP_459150 | nadC | nicotinate-nucleotide pyrophosphorylase | - | NP_414651 | nadC | nicotinate-nucleotide pyrophosphorylase | GCO | 81.56% | 57.94% |
| NP_459151 | ampD | N-acetyl-anhydromuramyl-L-alanine amidase | - | NP_414652 | ampD | N-acetyl-anhydromuranmyl-L-alanine amidase | GCO | 89.94% | 55.14% |
| NP_459152 | ampE | putative beta lactamase regulator | - | NP_414653 | ampE | predicted inner membrane protein | GCO | 82.39% | 55.67% |
| NP_459153 | - | putative cytoplasmic protein | - |  |  |  | NO HOMOLOG |  | 53.41% |
| NP_459154 | - | Na+/galactoside symporter | - | NP_418114 | yicJ | predicted transporter | nGCO | 43.73% | 50.95% |
| NP_459155 | aroP | aromatic amino acid transporter | - | NP_414654 | aroP | aromatic amino acid transporter | GCO | 84.02% | 55.60% |
| NP_459156 | pdhR | transcriptional regulator of pyruvate dehydrogenase complex | - | NP_414655 | pdhR | transcriptional regulator of pyruvate dehydrogenase complex | GCO | 97.00% | 56.47% |
| NP_459157 | aceE | pyruvate dehydrogenase E1 component | - | NP_414656 | aceE | pyruvate dehydrogenase subunit E1 | GCO | 95.26% | 54.31% |
| NP_459158 | aceF | dihydrolipoamide acetyltransferase | - | NP_414657 | aceF | dihydrolipoamide acetyltransferase | GCO | 78.73% | 55.55% |
| NP_459159 | lpdA | dihydrolipoamide dehydrogenase | - | NP_414658 | lpd | dihydrolipoamide dehydrogenase | GCO | 95.35% | 54.17% |
| NP_459160 | - | putative outer membrane protein | - |  |  |  | NO HOMOLOG |  | 55.78% |
| NP_459161 | - | putative periplasmic protein | - |  |  |  | NO HOMOLOG |  | 56.69% |
| NP_459162 | yacH | putative outer membrane protein | - | NP_414659 | yacH | hypothetical protein | GCO | 50.76% | 57.36% |
| NP_459163 | acnB | aconitate hydratase | - | NP_414660 | acnB | aconitate hydratase | GCO | 92.13% | 57.92% |
| NP_459164 | - | putative restriction endonuclease | - |  |  |  | NO HOMOLOG |  | 31.07% |
| NP_459165 | yacL | hypothetical protein | - | NP_414661 | yacL | hypothetical protein | nGCO | 85.71% | 51.79% |
| NP_459166 | kdgT | 2-keto-3-deoxygluconate permease | - | NP_418345 | kdgT | 2-keto-3-deoxygluconate permease | nGCO | 34.47% | 57.54% |
| NP_459167 | - | putative inner membrane protein | - | NP_417217 | ygbK | hypothetical protein | nGCO | 25.62% | 57.78% |
| NP_459168 | - | 4-hydroxythreonine-4-phosphate dehydrogenase | - | NP_414594 | pdxA | 4-hydroxythreonine-4-phosphate dehydrogenase | nGCO | 37.08% | 55.69% |
| NP_459169 | - | putative transcriptional regulator | - | NP_417215 | ygbI | predicted DNA-binding transcriptional regulator | nGCO | 34.66% | 55.07% |
| NP_459170 | speD | S-adenosylmethionine decarboxylase proenzyme | - | NP_414662 | speD | S-adenosylmethionine decarboxylase proenzyme | GCO | 96.59% | 49.68% |
| NP_459171 | speE | spermidine synthase | - | NP_414663 | speE | spermidine synthase | GCO | 94.75% | 53.42% |
| NP_459172 | yacC | putative periplasmic protein | - | NP_414664 | yacC | hypothetical protein | GCO | 95.65% | 47.12% |
| NP_459173 | cueO | putative multicopper oxidase | - | NP_414665 | cueO | multicopper oxidase (laccase) | GCO | 80.97% | 56.42% |
| NP_459174 | gcd | glucose dehydrogenase | - | NP_414666 | gcd | glucose dehydrogenase | GCO | 84.17% | 57.71% |
| NP_459175 | hpt | hypoxanthine-guanine phosphoribosyltransferase | - | NP_414667 | hpt | hypoxanthine-guanine phosphoribosyltransferase | GCO | 96.06% | 50.27% |
| NP_459176 | yadF | putative carbonic anhydrase | - | NP_414668 | can | carbonic anhydrase | GCO | 85% | 50.07% |
| NP_459177 | yadG | putative ABC-type multidrug transport system ATPase component | - | NP_414669 | yadG | predicted transporter subunit: ATP-binding component of ABC superfamily | GCO | 95.12% | 49.73% |
| NP_459178 | yadH | putative transport protein | - | NP_414670 | yadH | predicted transporter subunit: membrane component of ABC superfamily | GCO | 89.06% | 54.08% |
| NP_459179 | stiH | putative fimbrial protein precurosr | - | NP_415461 | ycbT | predicted fimbrial-like adhesin protein | GCO | 27.05% | 52.68% |
| NP_459180 | stiC | putativie fimbrial usher | - | NP_415460 | ycbS | predicted outer membrane usher protein | GCO | 38.67% | 55.35% |
| NP_459181 | stiB | putative fimbrial chaparone | - | NP_418736 | fimC | chaperone, periplasmic | nGCO | 39.10% | 53.07% |
| NP_459182 | stiA | putative fimbrial subunit | - | NP_418734 | fimA | major type 1 subunit fimbrin (pilin) | nGCO | 34.75% | 52.40% |
| NP_459183 | yadI | putative PTS enzyme | - | NP_414671 | yadI | predicted PTS Enzyme IIA | GCO | 76.71% | 51.24% |
| NP_459184 | yadE | putative xylanase/chitin deacetylase | - | NP_414672 | yadE | predicted polysaccharide deacetylase lipoprotein | GCO | 81.90% | 50.32% |
| NP_459185 | panD | aspartate 1-decarboxylase precursor | - | NP_414673 | panD | aspartate 1-decarboxylase precursor | GCO | 96.03% | 53.54% |
| NP_459186 | panC | pantoate--beta-alanine ligase | - | NP_414675 | panC | pantoate--beta-alanine ligase | GCO | 84.80% | 54.50% |
| NP_459187 | panB | 3-methyl-2-oxobutanoate hydroxymethyltransferase | - | NP_414676 | panB | 3-methyl-2-oxobutanoate hydroxymethyltransferase | GCO | 85.22% | 57.32% |
| NP_459188 | folK | 7,8-dihydro-6-hydroxymethylpterin-pyrophosphokinase | - | NP_414684 | folK | 2-amino-4-hydroxy-6-hydroxymethyldihyropteridine pyrophosphokinase | GCO | 87.83% | 57.50% |
| NP_459189 | pcnB | polyA polymerase I | - | NP_414685 | pcnB | poly(A) polymerase I | GCO | 88.51% | 56.16% |
| NP_459190 | yadB | glutamyl-tRNA synthetase | - | NP_414686 | yadB | glutamyl-Q tRNA(Asp) synthetase | GCO | 79.86% | 57.85% |
| NP_459191 | dksA | dnaK suppressor protein | - | NP_414687 | dksA | DNA-binding transcriptional regulator of rRNA transcription, DnaK suppressor protein | GCO | 88.74% | 53.72% |
| NP_459192 | sfsA | sugar fermentation stimulation protein | - | NP_414688 | sfsA | sugar fermentation stimulation protein A | GCO | 78.63% | 50.35% |
| NP_459193 | ligT | 2'-5' RNA ligase | - | NP_414689 | ligT | 2'-5' RNA ligase | GCO | 77.58% | 62.90% |
| NP_459194 | hrpB | ATP-dependent helicase | - | NP_414690 | hrpB | predicted ATP-dependent helicase | GCO | 78.49% | 59.31% |
| NP_459195 | mrcB | penicillin-binding protein 1b | - | NP_414691 | mrcB | penicillin-binding protein 1b | GCO | 84.17% | 56.75% |
| NP_459196 | fhuA | outer membrane ferrichrome receptor protein precursor | - | NP_414692 | fhuA | ferrichrome outer membrane transporter | GCO | 71.35% | 51.05% |
| NP_459197 | fhuC | hydroxymate-dependent iron transport protein | - | NP_414693 | fhuC | iron-hydroxamate transporter subunit | GCO | 92.07% | 58.89% |
| NP_459198 | fhuD | hydroxamate-dependent iron uptake protein | - | NP_414694 | fhuD | iron-hydroxamate transporter subunit | GCO | 79.07% | 59.48% |
| NP_459199 | fhuB | hydroxamate-dependent iron uptake protein | - | NP_414695 | fhuB | fused iron-hydroxamate transporter subunits of ABC superfamily: membrane components | GCO | 68.13% | 61.07% |
| NP_459200 | stfA | putative fimbrial subunit | - | NP_416841 | yfcV | predicted fimbrial-like adhesin protein | GCO | 37.57% | 52.04% |
| NP_459201 | stfC | putative fimbrial outer membrane usher | - | YP_026167 | yfcU | predicted export usher protein | GCO | 58.40% | 54.96% |
| NP_459202 | stfD | putative periplasmic fimbrial chaperone | - | NP_416839 | yfcS | predicted periplasmic pilus chaperone | GCO | 63.67% | 54.18% |
| NP_459203 | stfE | putative minor fimbrial subunit | - | NP_416838 | yfcR | predicted fimbrial-like adhesin protein | GCO | 44.44% | 55.94% |
| NP_459204 | stfF | putative minor fimbrial subunit | - | NP_416837 | yfcQ | predicted fimbrial-like adhesin protein | GCO | 46.91% | 48.42% |
| NP_459205 | stfG | putative minor fimbrial subunit | - | NP_416836 | yfcP | predicted fimbrial-like adhesin protein | GCO | 37.28% | 54.61% |
| NP_459206 | - | putative outer membrane protein | - | NP_416835 | yfcO | hypothetical protein | GCO | 37.80% | 49.76% |
| NP_459207 | hemL | glutamate-1-semialdehyde aminotransferase | - | NP_414696 | hemL | glutamate-1-semialdehyde aminotransferase | GCO | 97.41% | 59.32% |
| NP_459208 | yadQ | chloride channel protein | - | NP_414697 | clcA | chloride channel protein | GCO | 63.21% | 57.17% |
| NP_459209 | yadR | hypothetical protein | - | NP_414698 | yadR | hypothetical protein | GCO | 99.12% | 50.72% |
| NP_459210 | yadS | putative inner membrane protein | - | NP_414699 | yadS | conserved inner membrane protein | GCO | 83.57% | 56.57% |
| NP_459211 | btuF | vitamin B12-transporter protein BtuF | - | NP_414700 | btuF | vitamin B12-transporter protein BtuF | GCO | 77.44% | 55.68% |
| NP_459212 | pfs | 5'-methylthioadenosine/S-adenosylhomocysteine nucleosidase | - | NP_414701 | - | 5'-methylthioadenosine/S-adenosylhomocysteine nucleosidase | GCO | 96.12% | 56.93% |
| NP_459213 | dgt | deoxyguanosinetriphosphate triphosphohydrolase | - | NP_414702 | dgt | deoxyguanosinetriphosphate triphosphohydrolase | GCO | 88.51% | 49.07% |
| NP_459214 | htrA | high temperature requirement A protein precursor | + | NP_414703 | degP | serine endoprotease (protease Do), membrane-associated | GCO | 88.61% | 54.90% |
| NP_459215 | cdaR | putative inner membrane protein | - | NP_414704 | cdaR | DNA-binding transcriptional activator | GCO | 95.58% | 55.69% |
| NP_459216 | yaeH | putative cytoplasmic protein | - | NP_414705 | yaeH | hypothetical protein | GCO | 85.15% | 46.77% |
| NP_459217 | - | putative inner membrane protein | - | NP_416488 | shiA | shikimate transporter | nGCO | 38.90% | 51.22% |
| NP_459218 | dapD | 2,3,4,5-tetrahydropyridine-2-carboxylate N-succinyltransferase | - | NP_414708 | dapD | 2,3,4,5-tetrahydropyridine-2-carboxylate N-succinyltransferase | GCO | 91.24% | 53.93% |
| NP_459219 | glnD | PII uridylyl-transferase | - | NP_414709 | glnD | PII uridylyl-transferase | GCO | 90.44% | 56.11% |
| NP_459220 | map | methionine aminopeptidase | - | NP_414710 | map | methionine aminopeptidase | GCO | 89.39% | 52.45% |
| NP_459221 | rpsB | 30S ribosomal protein S2 | - | NP_414711 | rpsB | 30S ribosomal protein S2 | GCO | 97.51% | 50.96% |
| NP_459222 | tsf | elongation factor Ts | - | NP_414712 | tsf | elongation factor Ts | GCO | 90.10% | 52.58% |
| NP_459223 | pyrH | uridylate kinase | - | NP_414713 | pyrH | uridylate kinase | GCO | 91.70% | 53.85% |
| NP_459224 | frr | ribosome releasing factor | - | NP_414714 | frr | ribosome releasing factor | GCO | 86.48% | 51.07% |
| NP_459225 | dxr | 1-deoxy-D-xylulose 5-phosphate reductoisomerase | - | NP_414715 | dxr | 1-deoxy-D-xylulose 5-phosphate reductoisomerase | GCO | 88.91% | 55.88% |
| NP_459226 | uppS | undecaprenyl pyrophosphate synthetase | - | NP_414716 | ispU | undecaprenyl pyrophosphate synthase | GCO | 93.65% | 51.25% |
| NP_459227 | cdsA | CDP-diglyceride synthase | - | NP_414717 | geneA | CDP-diglyceride synthase | GCO | 84.91% | 52.79% |
| NP_459228 | yaeL | putative membrane-associated Zn-dependent protease | - | NP_414718 | yaeL | zinc metallopeptidase | GCO | 88.22% | 53.06% |
| NP_459229 | yaeT | putative outer membrane protein precursor | - | NP_414719 | yaeT | hypothetical protein | GCO | 92.97% | 52.46% |
| NP_459230 | hlpA | outer membrane protein H precursor | - | NP_414720 | hlpA | periplasmic chaperone | GCO | 85.09% | 47.32% |
| NP_459231 | lpxD | UDP-3-O-[3-hydroxymyristoyl] glucosamine N-acyltransferase | - | NP_414721 | lpxD | UDP-3-O-[3-hydroxymyristoyl] glucosamine N-acyltransferase | GCO | 92.37% | 51.94% |
| NP_459232 | fabZ | (3R)-hydroxymyristoyl ACP dehydratase | - | NP_414722 | fabZ | (3R)-hydroxymyristoyl ACP dehydratase | GCO | 99.33% | 52.41% |
| NP_459233 | lpxA | UDP-N-acetylglucosamine acyltransferase | - | NP_414723 | lpxA | UDP-N-acetylglucosamine acyltransferase | GCO | 90.07% | 53.48% |
| NP_459234 | lpxB | lipid-A-disaccharide synthase | - | NP_414724 | lpxB | lipid-A-disaccharide synthase | GCO | 94.76% | 55.70% |
| NP_459235 | rnhB | ribonuclease HII | - | NP_414725 | rnhB | ribonuclease HII | GCO | 92.92% | 56.28% |
| NP_459236 | dnaE | DNA polymerase III subunit alpha | - | NP_414726 | dnaE | DNA polymerase III subunit alpha | GCO | 94.22% | 55.90% |
| NP_459237 | accA | acetyl-CoA carboxylase alpha subunit | - | NP_414727 | accA | acetyl-CoA carboxylase subunit alpha | GCO | 92.47% | 54.79% |
| NP_459238 | - | putative endochitinase | - |  |  |  | NO HOMOLOG |  | 55.61% |
| NP_459239 | ldcC | lysine decarboxylase 2 | - | NP_414728 | ldcC | lysine decarboxylase 2, constitutive | GCO | 91.15% | 51.96% |
| NP_459240 | yaeR | putative lactoylglutathione lyase | - | NP_414729 | yaeR | predicted lyase | GCO | 79.06% | 52.30% |
| NP_459241 | mesJ | cell cycle protein | - | NP_414730 | tilS | tRNA(Ile)-lysidine synthetase | GCO | 70.12% | 58.77% |
| NP_459242 | rof | Rho-dependent transcription termination modulator | - | NP_414731 | rof | modulator of Rho-dependent transcription termination | GCO | 80.95% | 43.29% |
| NP_459243 | yaeP | hypothetical protein | - | YP_026160 | yaeP | hypothetical protein | GCO | 92.42% | 50.22% |
| NP_459244 | yaeQ | putative cytoplasmic protein | - | NP_414732 | yaeQ | hypothetical protein | GCO | 88.95% | 55.67% |
| NP_459245 | yaeJ | hypothetical protein | - | NP_414733 | yaeJ | hypothetical protein | GCO | 85.29% | 55.08% |
| NP_459246 | proS | prolyl-tRNA synthetase | - | NP_414736 | proS | prolyl-tRNA synthetase | GCO | 93.35% | 54.74% |
| NP_459247 | yaeB | putative regulatory protein | - | NP_414737 | yaeB | hypothetical protein | GCO | 84.25% | 55.79% |
| NP_459248 | rcsF | colanic acid synthesis regulator | - | NP_414738 | rcsF | predicted outer membrane protein, signal | GCO | 79.85% | 54.07% |
| NP_459249 | yaeC | putative outer membrane lipoprotein | - | NP_414739 | metQ | DL-methionine transporter subunit | GCO | 91.88% | 48.77% |
| NP_459250 | yaeE | putative transport protein | - | NP_414740 | metI | DL-methionine transporter subunit | GCO | 76.03% | 53.36% |
| NP_459251 | abc | putative transport protein | - | NP_414741 | metN | DL-methionine transporter subunit | GCO | 95.33% | 52.42% |
| NP_459252 | yaeD | hypothetical protein | - | NP_414742 | gmhB | hypothetical protein | GCO | 94.68% | 51.32% |
| NP_459253 | yafB | 2,5-diketo-D-gluconate reductase B | - | NP_414743 | dkgB | 2,5-diketo-D-gluconate reductase B | GCO | 92.50% | 51.11% |
| NP_459254 | yafC | putative transcriptional regulator | - | NP_414744 | yafC | predicted DNA-binding transcriptional regulator | GCO | 90.42% | 51.69% |
| NP_459255 | - | putative drug efflux protein | - | NP_416174 | ydhP | predicted transporter | nGCO | 41.53% | 55.78% |
| NP_459256 | yafD | hypothetical protein | - | NP_414745 | yafD | hypothetical protein | GCO | 96.91% | 53.84% |
| NP_459257 | yafE | putative methyltransferase | - | NP_414746 | yafE | predicted S-adenosyl-L-methionine-dependent methyltransferase | GCO | 71.07% | 52.52% |
| NP_459258 | dniR | transcriptional regulator | - | NP_414747 | mltD | predicted membrane-bound lytic murein transglycosylase D | GCO | 91.42% | 52.11% |
| NP_459259 | gloB | hydroxyacylglutathione hydrolase | - | NP_414748 | gloB | predicted hydroxyacylglutathione hydrolase | GCO | 78.48% | 44.17% |
| NP_459260 | yafS | putative SAM-dependent methyltransferase | - | NP_414749 | yafS | predicted S-adenosyl-L-methionine-dependent methyltransferase | GCO | 86.97% | 50.76% |
| NP_459261 | rnhA | ribonuclease H | - | NP_414750 | rnhA | ribonuclease H | GCO | 93.50% | 50.21% |
| NP_459262 | dnaQ | DNA polymerase III subunit epsilon | - | NP_414751 | dnaQ | DNA polymerase III subunit epsilon | GCO | 93.41% | 51.09% |
| NP_459263 | - | putative cytoplasmic protein | - |  |  |  | NO HOMOLOG |  | 61.26% |
| NP_459264 | - | putative cytoplasmic protein | - |  |  |  | NO HOMOLOG |  | 60.04% |
| NP_459265 | - | putative cytoplasmic protein | - |  |  |  | NO HOMOLOG |  | 59.76% |
| NP_459266 | - | putative cytoplasmic protein | - |  |  |  | NO HOMOLOG |  | 56.16% |
| NP_459267 | - | putative cytoplasmic protein | - |  |  |  | NO HOMOLOG |  | 59.63% |
| NP_459268 | - | putative cytoplasmic protein | - |  |  |  | NO HOMOLOG |  | 52.04% |
| NP_459269 | - | putative chaperone ATPase | - | NP_417083 | clpB | protein disaggregation chaperone | nGCO | 37.11% | 59.54% |
| NP_459270 | - | putative cytoplasmic protein | - |  |  |  | NO HOMOLOG |  | 54.88% |
| NP_459271 | - | putative cytoplasmic protein | - |  |  |  | NO HOMOLOG |  | 54.67% |
| NP_459272 | - | invasol SirA | - |  |  |  | NO HOMOLOG |  | 42.34% |
| NP_459273 | - | putative cytoplasmic protein | - |  |  |  | NO HOMOLOG |  | 43.84% |
| NP_459274 | - | putative cytoplasmic protein | - |  |  |  | NO HOMOLOG |  | 53.70% |
| NP_459275 | - | putative cytoplasmic protein | + |  |  |  | NO HOMOLOG |  | 41.15% |
| NP_459276 | - | putative periplasmic protein | + |  |  |  | NO HOMOLOG |  | 37.50% |
| NP_459277 | - | putative cytoplasmic protein | - |  |  |  | NO HOMOLOG |  | 49.79% |
| NP_459278 | - | putative outer membrane lipoprotein | + |  |  |  | NO HOMOLOG |  | 52.88% |
| NP_459279 | - | putative cytoplasmic protein | - |  |  |  | NO HOMOLOG |  | 58.25% |
| NP_459280 | - | hypothetical protein | - |  |  |  | NO HOMOLOG |  | 59.38% |
| NP_459281 | - | putative inner membrane protein | + |  |  |  | NO HOMOLOG |  | 41.73% |
| NP_459282 | - | putative Shiga-like toxin A subunit | + |  |  |  | NO HOMOLOG |  | 34.02% |
| NP_459283 | - | putative inner membrane protein | - |  |  |  | NO HOMOLOG |  | 62.11% |
| NP_459284 | - | putative cytoplasmic protein | - |  |  |  | NO HOMOLOG |  | 65.52% |
| NP_459285 | - | putative periplasmic protein | + |  |  |  | NO HOMOLOG |  | 47.72% |
| NP_459286 | - | putative cytoplasmic protein | + |  |  |  | NO HOMOLOG |  | 47.31% |
| NP_459287 | - | putative cytoplasmic protein | + |  |  |  | NO HOMOLOG |  | 56.84% |
| NP_459288 | - | putative cytoplasmic protein | + |  |  |  | NO HOMOLOG |  | 51.45% |
| NP_459289 | - | putative RHS-like protein | - | NP_415030 | rhsD | rhsD element protein | nGCO | 26.75% | 60.46% |
| NP_459290 | - | putative RHS-like protein | + |  |  |  | NO HOMOLOG |  | 52.49% |
| NP_459291 | - | putative cytoplasmic protein | + |  |  |  | NO HOMOLOG |  | 29.75% |
| NP_459292 | - | putative cytoplasmic protein | + |  |  |  | NO HOMOLOG |  | 31.96% |
| NP_945154 | - | hypothetical protein | - |  |  |  | NO HOMOLOG |  | 52.89% |
| NP_459293 | - | putative cytoplasmic protein | - |  |  |  | NO HOMOLOG |  | 32.03% |
| NP_459295 | - | putative transposase | - |  |  |  | NO HOMOLOG |  | 52.52% |
| NP_459297 | safA | putative outer membrane protein | + |  |  |  | NO HOMOLOG |  | 42.88% |
| NP_459298 | safB | putative fimbrial assembly chaparone | + | NP_417612 | yraI | predicted periplasmic pilin chaperone | nGCO | 35.64% | 48.87% |
| NP_459299 | safC | putative fimbrial usher | + | NP_418737 | fimD | outer membrane usher protein, type 1 fimbrial synthesis | nGCO | 35.17% | 54.67% |
| NP_459300 | safD | putative fimbrial subunit | + |  |  |  | NO HOMOLOG |  | 54.98% |
| NP_459301 | ybeJ | putative xylanase/chitin deacetylase | + | NP_414672 | yadE | predicted polysaccharide deacetylase lipoprotein | nGCO | 26.18% | 51.58% |
| NP_459302 | sinR | transcriptional regulator | + | NP_415420 | ycaN | predicted DNA-binding transcriptional regulator | nGCO | 26.02% | 39.76% |
| NP_459303 | - | putative cytoplasmic protein | + |  |  |  | NO HOMOLOG |  | 49.07% |
| NP_459304 | - | SapA-like protein | + |  |  |  | NO HOMOLOG |  | 42.77% |
| NP_459305 | - | VirG-like protein | - |  |  |  | NO HOMOLOG |  | 47.05% |
| NP_459306 | yafV | putative amidohydrolase | - | NP_414754 | yafV | predicted C-N hydrolase family amidase, NAD(P)-binding | nGCO | 82.35% | 54.16% |
| NP_459307 | fadE | acyl-CoA dehydrogenase | - | NP_414756 | fadE | acyl-CoA dehydrogenase | GCO | 94.10% | 56.03% |
| NP_459308 | ghmA | phosphoheptose isomerase | - | NP_414757 | lpcA | phosphoheptose isomerase | GCO | 97.39% | 47.66% |
| NP_459309 | yafJ | putative glutamine amidotransferase | - | NP_414758 | yafJ | predicted amidotransfease | GCO | 93.33% | 51.69% |
| NP_459310 | yafK | putative periplasmic protein | - | NP_414759 | yafK | hypothetical protein | GCO | 86.99% | 48.58% |
| NP_459311 | dinP | DNA polymerase IV | - | NP_414766 | dinB | DNA polymerase IV | nGCO | 87.74% | 51.42% |
| NP_459312 | prfH | peptide chain release factor 2 | - | NP_414771 | prfH | peptide chain release factor 2 | GCO | 82.71% | 55.12% |
| NP_459313 | pepD | aminoacyl-histidine dipeptidase | - | NP_414772 | pepD | aminoacyl-histidine dipeptidase (peptidase D) | GCO | 92.57% | 53.29% |
| NP_459314 | gpt | xanthine phosphoribosyltransferase | - | NP_414773 | gpt | xanthine phosphoribosyltransferase | GCO | 98.02% | 53.15% |
| NP_459315 | yafA | hypothetical protein | - | NP_414774 | frsA | fermentation/respiration switch protein | GCO | 89.61% | 55.02% |
| NP_459316 | crl | transcriptional regulator | - | NP_414775 | crl | DNA-binding transcriptional regulator | GCO | 83.45% | 50% |
| NP_459317 | phoE | outer membrane pore protein E precursor | - | NP_414776 | phoE | outer membrane phosphoporin protein E | GCO | 89.12% | 47.19% |
| NP_459318 | proB | gamma-glutamyl kinase | - | NP_414777 | proB | gamma-glutamyl kinase | GCO | 96.73% | 55.70% |
| NP_459319 | proA | gamma-glutamyl phosphate reductase | - | NP_414778 | proA | gamma-glutamyl phosphate reductase | GCO | 83.21% | 56.27% |
| NP_459321 | - | putative truncated IS3 transposase | - | NP_416593 | insF-5 | IS3 element protein InsF | nGCO | 86.75% | 54.43% |
| NP_459322 | - | putative cytoplasmic protein | - | NP_416324 | yoaC | hypothetical protein | nGCO | 67.28% | 45.98% |
| NP_459323 | - | putative permease | + | NP_416113 | ynfM | predicted transporter | nGCO | 33.95% | 42.52% |
| NP_459324 | - | isopropylmalate isomerase large subunit | + | NP_414614 | leuC | isopropylmalate isomerase large subunit | GCO | 50.64% | 51.40% |
| NP_459325 | - | putative 3-isopropylmalate isomerase | - | NP_414613 | leuD | isopropylmalate isomerase small subunit | GCO | 38.21% | 52.31% |
| NP_459326 | - | putative fumarylacetoacetate hydrolase | - | NP_415698 | ycgM | predicted isomerase/hydrolase | nGCO | 33.81% | 49.82% |
| NP_459327 | - | putative hydrolase/acyltransferase | + |  |  |  | NO HOMOLOG |  | 51.47% |
| NP_459328 | - | putative transcriptional regulator | - | NP_417532 | ygiP | predicted DNA-binding transcriptional regulator | nGCO | 41.47% | 49.06% |
| NP_459329 | - | putative cytoplasmic protein | - |  |  |  | NO HOMOLOG |  | 51.64% |
| NP_459330 | - | putative outer membrane protein | + |  |  |  | NO HOMOLOG |  | 35.58% |
| NP_459331 | stbE | putative fimbrial chaparone | + | NP_418736 | fimC | chaperone, periplasmic | nGCO | 25.60% | 47.69% |
| NP_459332 | stbD | putative fimbrial usher | + |  |  |  | NO HOMOLOG |  | 52.11% |
| NP_459333 | stbC | putative fimbrial usher | - | NP_418737 | fimD | outer membrane usher protein, type 1 fimbrial synthesis | nGCO | 30.80% | 53.94% |
| NP_459334 | stbB | putative fimbrial chaperone | + | NP_414682 | ecpD | predicted periplasmic pilin chaperone | nGCO | 40.32% | 45.40% |
| NP_459335 | stbA | putative fimbrial major subunit | + | NP_418734 | fimA | major type 1 subunit fimbrin (pilin) | nGCO | 28.93% | 47.11% |
| NP_459336 | - | putative inner membrane protein | + |  |  |  | NO HOMOLOG |  | 36.07% |
| NP_459337 | - | putative periplasmic protein | + |  |  |  | NO HOMOLOG |  | 39.66% |
| NP_459338 | - | hypothetical protein | + | NP_416681 | rtn | hypothetical protein | nGCO | 38.72% | 48.44% |
| NP_459339 | - | putative response regulator | + |  |  |  | NO HOMOLOG |  | 40.86% |
| NP_459340 | - | putative inner membrane protein | + |  |  |  | NO HOMOLOG |  | 39.26% |
| NP_459341 | - | putative outer membrane protein | + | NP_415335 | ompX | outer membrane protein X | nGCO | 39.49% | 41.52% |
| NP_459342 | - | putative response regulator | + |  |  |  | NO HOMOLOG |  | 39.71% |
| NP_459343 | - | putative inner membrane protein | + |  |  |  | NO HOMOLOG |  | 35.11% |
| NP_459344 | - | putative outer membrane lipoprotein | + | NP_418568 | yjeI | hypothetical protein | nGCO | 34.45% | 49.44% |
| NP_459345 | - | outer membrane efflux-like protein | - | NP_415104 | cusC | copper/silver efflux system, outer membrane component | nGCO | 32.89% | 59.19% |
| NP_459346 | - | putative cation efflux system protein | - | NP_417732 | acrF | multidrug efflux system protein | nGCO | 38.04% | 56.69% |
| NP_459347 | - | putative cation efflux pump | - | NP_414996 | acrA | multidrug efflux system | nGCO | 29.64% | 60.77% |
| NP_459348 | - | putative cation transport ATPase | - | NP_415017 | copA | copper transporter | nGCO | 42.04% | 60.02% |
| NP_459349 | - | putative transcriptional regulator | - | NP_415020 | cueR | DNA-binding transcriptional activator of copper-responsive regulon genes | nGCO | 40.47% | 54.40% |
| NP_459350 | - | putative copper chaperone | - |  |  |  | NO HOMOLOG |  | 57.94% |
| NP_459351 | - | putative inner membrane protein | - | NP_418699 | yjhB | KpLE2 phage-like element; predicted transporter | nGCO | 29.01% | 53.90% |
| NP_459352 | mod | DNA methylase | - |  |  |  | NO HOMOLOG |  | 46.75% |
| NP_459353 | res | DNA restriction enzyme | - |  |  |  | NO HOMOLOG |  | 49.15% |
| NP_459354 | - | putative cytoplasmic protein | - |  |  |  | NO HOMOLOG |  | 50% |
| NP_459355 | - | cytochrome BD2 subunit I | - | NP_415497 | appC | cytochrome bd-II oxidase, subunit I | nGCO | 32.81% | 55.41% |
| NP_459356 | - | cytochrome BD2 subunit II | - | NP_415262 | cydB | cytochrome d terminal oxidase, subunit II | nGCO | 28.49% | 55.19% |
| NP_459357 | - | putative cytoplasmic protein | - |  |  |  | NO HOMOLOG |  | 56.88% |
| NP_459358 | - | putative transcriptional regulator | - |  |  |  | NO HOMOLOG |  | 58.00% |
| NP_459359 | foxA | ferrioxamine receptor | - | NP_414692 | fhuA | ferrichrome outer membrane transporter | nGCO | 35.80% | 52.72% |
| NP_459360 | yahN | putative transport protein | - | NP_414862 | yahN | neutral amino-acid efflux system | GCO | 81.90% | 54.18% |
| NP_459361 | yahO | putative periplasmic protein | - | NP_414863 | yahO | hypothetical protein | GCO | 72.52% | 44.20% |
| NP_459362 | prpR | prp operon regulator | - | NP_414864 | prpR | DNA-binding transcriptional activator | GCO | 79.51% | 55.71% |
| NP_459363 | prpB | putative carboxyphosphonoenolpyruvate mutase | + | NP_414865 | prpB | 2-methylisocitrate lyase | GCO | 86.89% | 58.89% |
| NP_459364 | prpC | citrate synthase | + | NP_414867 | prpC | 2-methylcitrate synthase | GCO | 96.39% | 55.72% |
| NP_459365 | prpD | 2-methylcitrate dehydratase | + | NP_414868 | prpD | 2-methylcitrate dehydratase | GCO | 94.40% | 56.68% |
| NP_459366 | prpE | putative acetyl-CoA synthetase | + | NP_414869 | prpE | predicted propionyl-CoA synthetase with ATPase domain | GCO | 89.17% | 58.87% |
| NP_459367 | hemB | delta-aminolevulinic acid dehydratase | - | NP_414903 | hemB | delta-aminolevulinic acid dehydratase | nGCO | 88.88% | 55.38% |
| NP_459368 | yaiU | flagellar protein | + |  |  |  | NO HOMOLOG |  | 46.23% |
| NP_459369 | yaiV | putative inner membrane protein | - | NP_414909 | yaiV | predicted DNA-binding transcriptional regulator | GCO | 71.01% | 42.46% |
| NP_459370 | ampH | penicillin-binding protein | - | NP_414910 | ampH | beta-lactamase/D-alanine carboxypeptidase | GCO | 87.50% | 53.22% |
| NP_459371 | sbmA | putative ABC transporter membrane protein | - | NP_414911 | sbmA | predicted transporter | GCO | 92.58% | 51.59% |
| NP_459372 | yaiW | putative outer membrane lipoprotein | - | NP_414912 | yaiW | predicted DNA-binding transcriptional regulator | GCO | 85.63% | 55.34% |
| NP_459373 | yaiY | putative inner membrane protein | - | NP_414913 | yaiY | predicted inner membrane protein | GCO | 86.27% | 54.04% |
| NP_459374 | yaiZ | putative inner membrane protein | - | NP_414914 | yaiZ | predicted inner membrane protein | GCO | 89.23% | 47.68% |
| NP_459375 | ddl | D-alanylalanine synthetase | - | NP_414915 | ddlA | D-alanylalanine synthetase | GCO | 89.83% | 52.23% |
| NP_459376 | - | putative inner membrane protein | - |  |  |  | NO HOMOLOG |  | 54.34% |
| NP_459377 | - | putative permease | - | NP_415571 | mdtG | predicted drug efflux system | nGCO | 56.81% | 52.39% |
| NP_459378 | yaiB | putative cytoplasmic protein | - | NP_414916 | yaiB | hypothetical protein | nGCO | 68.23% | 46.81% |
| NP_459379 | psiF | phosphate starvation-inducible protein | - | NP_414918 | psiF | hypothetical protein | GCO | 89.53% | 51.40% |
| NP_459380 | yaiC | hypothetical protein | - | NP_414919 | yaiC | predicted diguanylate cyclase | GCO | 75.20% | 55.43% |
| NP_459381 | proC | pyrroline-5-carboxylate reductase | - | NP_414920 | proC | pyrroline-5-carboxylate reductase | GCO | 91.44% | 56.54% |
| NP_459382 | yaiI | hypothetical protein | - | NP_414921 | yaiI | hypothetical protein | GCO | 89.40% | 58.11% |
| NP_459383 | aroL | shikimate kinase II | - | NP_414922 | aroL | shikimate kinase II | GCO | 74.40% | 58.24% |
| NP_459384 | yaiA | putative cytoplasmic protein | - | NP_414923 | yaiA | hypothetical protein | GCO | 93.54% | 52.60% |
| NP_459385 | aroM | aro operon protein | - | NP_414924 | aroM | hypothetical protein | GCO | 73.77% | 50.58% |
| NP_459386 | yaiE | putative cytoplasmic protein | - | NP_414925 | yaiE | hypothetical protein | GCO | 86.17% | 52.98% |
| NP_459387 | rdgC | recombination associated protein | - | NP_414927 | rdgC | recombination associated protein | GCO | 93.06% | 53.94% |
| NP_459388 | yajF | putative sugar kinase/transcriptional regulator | - | NP_414928 | mak | fructokinase | GCO | 88.66% | 58.08% |
| NP_459389 | araJ | arabinose polymer transporter | - | NP_414930 | araJ | predicted transporter | GCO | 75.25% | 55.24% |
| NP_459390 | sbcC | ATP-dependent dsDNA exonuclease | - | NP_414931 | sbcC | exonuclease, dsDNA, ATP-dependent | GCO | 56.87% | 58.26% |
| NP_459391 | sbcD | ATP-dependent dsDNA exonuclease | - | NP_414932 | sbcD | exonuclease, dsDNA, ATP-dependent | GCO | 84% | 56.60% |
| NP_459392 | phoB | response regulator | - | NP_414933 | phoB | DNA-binding response regulator in two-component regulatory system with PhoR (or CreC) | GCO | 95.63% | 54.63% |
| NP_459393 | phoR | sensor kinase | - | NP_414934 | phoR | sensory histidine kinase in two-component regulatory system with PhoB | GCO | 91.18% | 53.78% |
| NP_459394 | brnQ | branched-chain amino acid transporter | - | NP_414935 | brnQ | predicted branched chain amino acid transporter (LIV-II) | GCO | 87.92% | 56.89% |
| NP_459395 | proY | putative proline transporter | - | NP_414936 | proY | predicted cryptic proline transporter | GCO | 86.59% | 54.12% |
| NP_459396 | malZ | maltodextrin glucosidase | - | NP_414937 | malZ | maltodextrin glucosidase | GCO | 81.45% | 57.59% |
| NP_459397 | - | putative thiol-alkyl hydroperoxide reductase | - | NP_415138 | ahpC | alkyl hydroperoxide reductase, C22 subunit | nGCO | 39.02% | 53.23% |
| NP_459398 | yajB | putative cytoplasmic protein | - | NP_414938 | yajB | hypothetical protein | GCO | 80.31% | 54.98% |
| NP_459399 | queA | S-adenosylmethionine:tRNA ribosyltransferase-isomerase | - | NP_414939 | queA | S-adenosylmethionine:tRNA ribosyltransferase-isomerase | GCO | 94.33% | 55.86% |
| NP_459400 | tgt | queuine tRNA-ribosyltransferase | - | NP_414940 | tgt | queuine tRNA-ribosyltransferase | GCO | 97.86% | 52.48% |
| NP_459401 | yajC | preprotein translocase subunit YajC | - | NP_414941 | yajC | preprotein translocase subunit YajC | GCO | 99.09% | 51.95% |
| NP_459402 | secD | protein export protein SecD | - | NP_414942 | secD | protein export protein SecD | GCO | 93.82% | 54.49% |
| NP_459403 | secF | protein export protein SecF | - | NP_414943 | secF | protein export protein SecF | GCO | 95.35% | 54.21% |
| NP_459404 | - | hypothetical protein | - |  |  |  | NO HOMOLOG |  | 50.54% |
| NP_459405 | - | putative regulatory protein | - |  |  |  | NO HOMOLOG |  | 52.81% |
| NP_459406 | yajD | putative cytoplasmic protein | - | NP_414944 | yajD | hypothetical protein | GCO | 96.52% | 49.71% |
| NP_459408 | tsx | nucleoside channel | - | NP_414945 | tsx | nucleoside channel, receptor of phage T6 and colicin K | GCO | 81.61% | 51.27% |
| NP_459409 | yajI | putative outer membrane lipoprotein | - | NP_414946 | yajI | predicted lipoprotein | GCO | 75.30% | 52.77% |
| NP_459410 | ybaD | hypothetical protein | - | NP_414947 | ybaD | hypothetical protein | GCO | 95.97% | 50% |
| NP_459411 | ribD | pyrimidine deaminase/reductase | - | NP_414948 | ribD | fused diaminohydroxyphosphoribosylaminopyrimidine deaminase and 5-amino-6-(5-phosphoribosylamino) uracil reductase | GCO | 86.10% | 60.32% |
| NP_459412 | ribH | riboflavin synthase subunit beta | - | NP_414949 | ribE | riboflavin synthase subunit beta | GCO | 91.02% | 54.35% |
| NP_459413 | nusB | transcription antitermination protein NusB | - | NP_414950 | nusB | transcription antitermination protein NusB | GCO | 97.12% | 52.85% |
| NP_459414 | thiL | thiamine monophosphate kinase | - | NP_414951 | thiL | thiamine monophosphate kinase | GCO | 88% | 56.54% |
| NP_459415 | pgpA | phosphatidylglycerophosphatase A | - | NP_414952 | pgpA | phosphatidylglycerophosphatase A | GCO | 95.32% | 54.06% |
| NP_459416 | yajO | putative oxidoreductase | - | NP_414953 | yajO | predicted oxidoreductase, NAD(P)-binding | GCO | 92.90% | 56.92% |
| NP_459417 | dxs | 1-deoxy-D-xylulose-5-phosphate synthase | - | NP_414954 | dxs | 1-deoxy-D-xylulose-5-phosphate synthase | GCO | 93.54% | 55.55% |
| NP_459418 | ispA | geranyltranstransferase | - | NP_414955 | ispA | geranyltranstransferase | GCO | 86.95% | 57.44% |
| NP_459419 | xseB | exodeoxyribonuclease VII small subunit | - | NP_414956 | xseB | exodeoxyribonuclease VII small subunit | GCO | 90% | 54.32% |
| NP_459420 | thiI | thiamine biosynthesis protein ThiI | - | NP_414957 | thiI | thiamine biosynthesis protein ThiI | GCO | 91.90% | 52.38% |
| NP_459421 | phnV | 2-aminoethylphosphonate transporter | - | NP_415960 | ydcV | predicted spermidine/putrescine transporter subunit | nGCO | 27.71% | 56.64% |
| NP_459422 | phnU | 2-aminoethylphosphonate transporter | - | NP_416919 | cysU | sulfate/thiosulfate transporter subunit | nGCO | 27.84% | 57.25% |
| NP_459423 | phnT | 2-aminoethylphosphonate transporter | - | NP_415376 | potG | putrescine transporter subunit: ATP-binding component of ABC superfamily | nGCO | 35.18% | 58.73% |
| NP_459424 | phnS | 2-aminoethylphosphonate transporter | - |  |  |  | NO HOMOLOG |  | 56.31% |
| NP_459425 | phnR | 2-aminoethylphosphonate transport protein | - | NP_418308 | yihL | predicted DNA-binding transcriptional regulator | nGCO | 33.03% | 59.02% |
| NP_459426 | phnW | 2-aminoethylphosphonate transport | - |  |  |  | NO HOMOLOG |  | 56.70% |
| NP_459427 | phnX | 2-aminoethylphosphonate transport | - |  |  |  | NO HOMOLOG |  | 59.77% |
| NP_459428 | thiJ | 4-methyl-5(beta-hydroxyethyl)-thiazole synthesis | - | NP_414958 | yajL | hypothetical protein | GCO | 91.32% | 56.85% |
| NP_459429 | apbA | 2-dehydropantoate 2-reductase | - | NP_414959 | panE | 2-dehydropantoate 2-reductase | GCO | 87.45% | 52.63% |
| NP_459430 | yajQ | hypothetical protein | - | NP_414960 | yajQ | nucleotide-binding protein | GCO | 90.18% | 50.98% |
| NP_459431 | yajR | putative transport protein | - | NP_414961 | yajR | predicted transporter | GCO | 83.81% | 55.45% |
| NP_459433 | - | putative periplasmic protein | + | NP_415177 | ybeQ | hypothetical protein | nGCO | 25.60% | 43.71% |
| NP_459434 | - | tetratricopeptide repeat protein | + |  |  |  | NO HOMOLOG |  | 39.56% |
| NP_459435 | cyoE | protoheme IX farnesyltransferase | - | NP_414962 | cyoE | protoheme IX farnesyltransferase | GCO | 89.86% | 53.75% |
| NP_459436 | cyoD | cytochrome o ubiquinol oxidase subunit IV | - | NP_414963 | cyoD | cytochrome o ubiquinol oxidase subunit IV | GCO | 93.57% | 51.51% |
| NP_459437 | cyoC | cytochrome o ubiquinol oxidase subunit III | - | NP_414964 | cyoC | cytochrome o ubiquinol oxidase subunit III | GCO | 96.66% | 53.49% |
| NP_459438 | cyoB | cytochrome o ubiquinol oxidase subunit I | - | NP_414965 | cyoB | cytochrome o ubiquinol oxidase subunit I | GCO | 91.85% | 53.76% |
| NP_459439 | cyoA | cytochrome o ubiquinol oxidase subunit II | - | NP_414966 | cyoA | cytochrome o ubiquinol oxidase subunit II | GCO | 92.06% | 50.67% |
| NP_459440 | ampG | muropeptide transporter | - | NP_414967 | ampG | muropeptide transporter | GCO | 82.85% | 56.57% |
| NP_459441 | yajG | putative lipoprotein | - | NP_414968 | yajG | predicted lipoprotein | GCO | 82.29% | 51.02% |
| NP_459442 | bolA | putative regulatory protein | - | NP_414969 | bolA | regulator of penicillin binding proteins and beta lactamase transcription (morphogene) | GCO | 92.38% | 49.05% |
| NP_459443 | tig | trigger factor | - | NP_414970 | tig | trigger factor | GCO | 86.34% | 52.42% |
| NP_459444 | clpP | ATP-dependent Clp protease proteolytic subunit | - | NP_414971 | clpP | ATP-dependent Clp protease proteolytic subunit | GCO | 99.03% | 51.28% |
| NP_459445 | clpX | ATP-dependent protease ATP-binding subunit | - | NP_414972 | clpX | ATP-dependent protease ATP-binding subunit | GCO | 98.34% | 52.83% |
| NP_459446 | lon | ATP-dependent protease Lon | - | NP_414973 | lon | DNA-binding ATP-dependent protease La | GCO | 97.44% | 54.01% |
| NP_459447 | hupB | DNA-binding protein HU-beta | - | NP_414974 | hupB | HU, DNA-binding transcriptional regulator, beta subunit | GCO | 74.44% | 49.45% |
| NP_459448 | cypD | peptidyl-prolyl isomerase | - | NP_414975 | ppiD | peptidyl-prolyl cis-trans isomerase (rotamase D) | GCO | 83.78% | 52.40% |
| NP_459449 | ybaV | putative DNA uptake protein | - | NP_414976 | ybaV | hypothetical protein | GCO | 75.80% | 52.53% |
| NP_459450 | ybaW | putative esterase | - | NP_414977 | ybaW | hypothetical protein | GCO | 85.60% | 47.11% |
| NP_459451 | ybaX | putative aluminum resistance protein | - | NP_414978 | ybaX | predicted aluminum resistance protein | GCO | 89.17% | 53.16% |
| NP_459452 | ybaE | putative ABC transporter periplasmic binding protein | - | NP_414979 | ybaE | predicted transporter subunit: periplasmic-binding component of ABC superfamily | GCO | 78.98% | 56.26% |
| NP_459453 | cof | putative hydrolase | - | NP_414980 | cof | thiamin pyrimidine pyrophosphate hydrolase | GCO | 81.25% | 54.57% |
| NP_459454 | - | putative cysteine synthase/cystathionine beta-synthase | - | NP_416916 | cysM | cysteine synthase B (O-acetylserine sulfhydrolase B) | nGCO | 25.96% | 54.64% |
| NP_459455 | ybaO | putative transcriptional regulator | - | NP_414981 | ybaO | predicted DNA-binding transcriptional regulator | GCO | 95.39% | 52.28% |
| NP_459456 | mdlA | ATP-binding component | - | NP_414982 | mdlA | fused predicted multidrug transporter subunits of ABC superfamily: ATP-binding components | GCO | 80.33% | 55.72% |
| NP_459457 | mdlB | putative transporter | - | NP_414983 | mdlB | fused predicted multidrug transporter subunits of ABC superfamily: ATP-binding components | GCO | 84.14% | 57.80% |
| NP_945155 | - | hypothetical protein | - |  |  |  | NO HOMOLOG |  | 52.08% |
| NP_459458 | glnK | nitrogen regulatory protein P-II 2 | - | NP_414984 | glnK | nitrogen assimilation regulatory protein for GlnL, GlnE, and AmtB | GCO | 97.32% | 53.09% |
| NP_459459 | amtB | putative ammonium transport protein | - | NP_414985 | amtB | ammonium transporter | GCO | 75.93% | 55.71% |
| NP_459460 | tesB | acyl-CoA thioesterase II | - | NP_414986 | tesB | acyl-CoA thioesterase II | GCO | 88.81% | 52.96% |
| NP_459461 | ybaY | hypothetical protein | - | NP_414987 | ybaY | predicted outer membrane lipoprotein | GCO | 72.10% | 54.03% |
| NP_459462 | ybaZ | putative methyltransferase | - | NP_414988 | ybaZ | predicted methyltransferase | GCO | 82.94% | 56.15% |
| NP_459463 | ylaB | hypothetical protein | - | NP_414990 | ylaB | conserved inner membrane protein | nGCO | 63.95% | 52.15% |
| NP_459464 | rpmE2 | 50S ribosomal protein L31 | - | NP_414830 | ykgM | 50S ribosomal protein L31 | GCO | 74.41% | 44.06% |
| NP_459465 | rpmJ2 | 50S ribosomal protein L36 | - | YP_588437 | ykgO | rpmJ (L36) paralog | GCO | 89.13% | 43.26% |
| NP_459466 | ylaC | putative inner membrane protein | - | NP_414991 | ylaC | predicted inner membrane protein | GCO | 75.48% | 48.83% |
| NP_459467 | maa | maltose O-acetyltransferase | - | NP_414992 | maa | maltose O-acetyltransferase | GCO | 72.67% | 48.91% |
| NP_459468 | hha | hemolysin expression-modulating protein | + | NP_414993 | hha | modulator of gene expression, with H-NS | GCO | 98.61% | 40.18% |
| NP_459469 | ybaJ | putative cytoplasmic protein | + | NP_414994 | ybaJ | hypothetical protein | GCO | 95.16% | 42.13% |
| NP_459470 | acrB | acridine efflux pump | - | NP_414995 | acrB | multidrug efflux system protein | GCO | 91.98% | 54.38% |
| NP_459471 | acrA | acridine efflux pump | - | NP_414996 | acrA | multidrug efflux system | GCO | 80.35% | 55.19% |
| NP_459472 | acrR | acrAB operon repressor | - | NP_414997 | acrR | DNA-binding transcriptional repressor | GCO | 87.38% | 46.78% |
| NP_459473 | aefA | putative small-conductance mechanosensitive channel | - | NP_414998 | kefA | fused conserved protein | GCO | 83.25% | 52.63% |
| NP_459474 | - | putative transposase | - | NP_416808 | yfcI | hypothetical protein | nGCO | 49.67% | 53.09% |
| NP_459475 | ybaM | putative inner membrane protein | - | NP_414999 | ybaM | hypothetical protein | GCO | 80.39% | 45.23% |
| NP_459476 | priC | primosomal replication protein N | - | NP_415000 | priC | primosomal replication protein N'' | GCO | 73.09% | 59.68% |
| NP_459477 | ybaN | hypothetical protein | - | NP_415001 | ybaN | conserved inner membrane protein | GCO | 56.45% | 53.43% |
| NP_459478 | apt | adenine phosphoribosyltransferase | - | NP_415002 | apt | adenine phosphoribosyltransferase | GCO | 95.08% | 53.80% |
| NP_459479 | dnaX | DNA polymerase III subunits gamma and tau | - | NP_415003 | dnaX | DNA polymerase III subunits gamma and tau | GCO | 87.09% | 59.04% |
| NP_459480 | ybaB | hypothetical protein | - | NP_415004 | ybaB | hypothetical protein | GCO | 70.64% | 54.54% |
| NP_459481 | recR | recombination protein RecR | - | NP_415005 | recR | recombination protein RecR | GCO | 89.55% | 61.05% |
| NP_459482 | htpG | heat shock protein 90 | - | NP_415006 | htpG | heat shock protein 90 | GCO | 94.71% | 52.90% |
| NP_459483 | adk | adenylate kinase | - | NP_415007 | adk | adenylate kinase | GCO | 96.26% | 54.57% |
| NP_459484 | hemH | ferrochelatase | - | NP_415008 | hemH | ferrochelatase | GCO | 88.75% | 56.80% |
| NP_459485 | aes | acetyl esterase | - | NP_415009 | aes | acetyl esterase | GCO | 69.90% | 56.27% |
| NP_459486 | gsk | inosine-guanosine kinase | - | NP_415010 | gsk | inosine/guanosine kinase | GCO | 91.70% | 53.40% |
| NP_459487 | ybaL | putative transport protein | - | NP_415011 | ybaL | predicted transporter with NAD(P)-binding Rossmann-fold domain | GCO | 88.84% | 57.06% |
| NP_459488 | fsr | putative transport protein | - | NP_415012 | fsr | predicted fosmidomycin efflux system | GCO | 77.03% | 55.85% |
| NP_459489 | ushA | UDP-sugar hydrolase/5'-nucleotidase | - | NP_415013 | ushA | UDP-sugar hydrolase | GCO | 85.97% | 51.72% |
| NP_459490 | ybaK | putative cytoplasmic protein | - | NP_415014 | ybaK | hypothetical protein | GCO | 72.32% | 53.75% |
| NP_459491 | ybaP | putative cytoplasmic protein | - | NP_415015 | ybaP | hypothetical protein | GCO | 66.28% | 57.10% |
| NP_459492 | - | putative periplasmic protein | + |  |  |  | NO HOMOLOG |  | 43.11% |
| NP_459493 | copA | putative copper-transporting ATPase | - | NP_415017 | copA | copper transporter | nGCO | 91.47% | 59.15% |
| NP_459494 | cueR | putative heavy metal transcriptional repressor | - | NP_415020 | cueR | DNA-binding transcriptional activator of copper-responsive regulon genes | GCO | 91.79% | 52.27% |
| NP_459495 | ybbJ | hypothetical protein | - | NP_415021 | ybbJ | conserved inner membrane protein | GCO | 61.90% | 60.70% |
| NP_459496 | ybbK | putative inner membrane protein | - | NP_415022 | ybbK | predicted protease, membrane anchored | GCO | 90.72% | 54.68% |
| NP_459497 | ybbL | putative ABC-type sugar/spermidine/putrescine transport system ATPase component | - | NP_415023 | ybbL | predicted transporter subunit: ATP-binding component of ABC superfamily | GCO | 74.22% | 47.49% |
| NP_459498 | ybbM | putative transport protein | - | NP_415024 | ybbM | predicted inner membrane protein | GCO | 75.58% | 51.41% |
| NP_459499 | ybbN | putative thioredoxin protein | - | NP_415025 | ybbN | predicted thioredoxin domain-containing protein | GCO | 89.78% | 55.55% |
| NP_459500 | ybbO | short chain dehydrogenase | - | NP_415026 | ybbO | short chain dehydrogenase | GCO | 90.98% | 54.99% |
| NP_459501 | tesA | multifunctional acyl-CoA thioesterase I | - | NP_415027 | tesA | multifunctional acyl-CoA thioesterase I and protease I and lysophospholipase L1 | GCO | 89.21% | 53.00% |
| NP_459502 | ybbA | putative transporter | - | NP_415028 | ybbA | predicted transporter subunit: ATP-binding component of ABC superfamily | GCO | 88.15% | 59.09% |
| NP_459503 | ybbP | putative inner membrane protein | - | NP_415029 | ybbP | predicted inner membrane protein | GCO | 69.40% | 58.50% |
| NP_459504 | - | putative outer membrane protein | - |  |  |  | NO HOMOLOG |  | 50.17% |
| NP_459505 | sfbA | putative ABC-type transport system ATPase component | - | NP_414739 | metQ | DL-methionine transporter subunit | nGCO | 37.34% | 52.58% |
| NP_459506 | sfbB | putative ABC-type transport system ATPase component | - | NP_414741 | metN | DL-methionine transporter subunit | GCO | 45.18% | 56.14% |
| NP_459507 | sfbC | putative ABC transporter permease component | - | NP_414740 | metI | DL-methionine transporter subunit | GCO | 44.11% | 54.39% |
| NP_459508 | ybbB | putative ATPase | - | NP_415036 | ybbB | tRNA 2-selenouridine synthase, selenophosphate-dependent | GCO | 78.02% | 55.98% |
| NP_459509 | ybbS | putative transcriptional regulator | - | NP_415037 | ybbS | DNA-binding transcriptional activator of the allD operon | GCO | 86.64% | 53.50% |
| NP_459510 | allA | ureidoglycolate hydrolase | - | NP_415038 | allA | ureidoglycolate hydrolase | GCO | 85% | 50.31% |
| NP_459511 | allR | putative regulatory protein | - | NP_415039 | allR | DNA-binding transcriptional repressor | GCO | 86.94% | 54.94% |
| NP_459512 | gcl | glyoxylate carboligase | - | NP_415040 | gcl | glyoxylate carboligase | GCO | 94.26% | 54.32% |
| NP_459513 | gip | glyoxylate-induced protein | - | NP_415041 | hyi | hydroxypyruvate isomerase | GCO | 84.10% | 45.68% |
| NP_459514 | glxR | tartronic semialdehyde reductase | - | NP_415042 | glxR | tartronate semialdehyde reductase, NADH-dependent | GCO | 91.78% | 50.85% |
| NP_459515 | - | putative permease | - | NP_418776 | yjiZ | predicted transporter | nGCO | 20.68% | 46.07% |
| NP_459516 | ybbV | putative cytoplasmic protein | - | NP_415043 | ybbV | hypothetical protein | GCO | 57.14% | 40.78% |
| NP_459517 | allP | putative allantoin transport protein | + | NP_415044 | ybbW | predicted allantoin transporter | GCO | 85.58% | 45.96% |
| NP_459518 | allB | allantoinase | + | NP_415045 | allB | allantoinase | GCO | 93.37% | 51.68% |
| NP_459519 | ybbY | putative transport protein | - | NP_415046 | ybbY | predicted uracil/xanthine transporter | GCO | 73.36% | 51.03% |
| NP_459520 | glxK | glycerate kinase II | + | NP_415047 | glxK | glycerate kinase II | GCO | 77.57% | 57.61% |
| NP_459521 | ylbA | putative glyoxylate utilization protein | + | NP_415048 | ylbA | hypothetical protein | GCO | 87.35% | 49.61% |
| NP_459522 | allC | N-carbamoyl-L-amino acid amidohydrolase | + | NP_415049 | allC | N-carbamoyl-L-amino acid amidohydrolase | GCO | 86.13% | 53.88% |
| NP_459523 | allD | ureidoglycolate dehydrogenase | + | NP_415050 | allD | ureidoglycolate dehydrogenase | GCO | 86.81% | 50% |
| NP_459524 | fdrA | succinyl-CoA synthetase alpha subunit | - | NP_415051 | fdrA | membrane protein FdrA | GCO | 76.03% | 57.29% |
| NP_459525 | ylbE | putative cytoplasmic protein | - | NP_414855 | yahG | hypothetical protein | nGCO | 43.47% | 57.14% |
| NP_459526 | ylbF | putative cytoplasmic protein | - | NP_415053 | ylbF | hypothetical protein | GCO | 65.42% | 57.40% |
| NP_459527 | arcC | carbamate kinase | - | NP_415054 | ybcF | predicted carbamate kinase | GCO | 75.08% | 58.16% |
| NP_459528 | purK | phosphoribosylaminoimidazole carboxylase | - | NP_415055 | purK | phosphoribosylaminoimidazole carboxylase | GCO | 86.40% | 59.26% |
| NP_459529 | purE | phosphoribosylaminoimidazole carboxylase catalytic subunit | - | NP_415056 | purE | phosphoribosylaminoimidazole carboxylase catalytic subunit | GCO | 84.43% | 60.19% |
| NP_459530 | lpxH | UDP-2,3-diacylglucosamine hydrolase | - | NP_415057 | lpxH | UDP-2,3-diacylglucosamine hydrolase | GCO | 80.75% | 55.46% |
| NP_459531 | ppiB | peptidyl-prolyl cis-trans isomerase B | - | NP_415058 | ppiB | peptidyl-prolyl cis-trans isomerase B (rotamase B) | GCO | 97.56% | 49.69% |
| NP_459532 | cysS | cysteinyl-tRNA synthetase | - | NP_415059 | cysS | cysteinyl-tRNA synthetase | GCO | 90.02% | 54.90% |
| NP_459533 | - | putative outer membrane protein | - |  |  |  | NO HOMOLOG |  | 51.63% |
| NP_459534 | - | putative inner membrane protein | - |  |  |  | NO HOMOLOG |  | 49.08% |
| NP_459535 | ybcI | putative membrane-bound metal-dependent hydrolase | - | NP_415060 | ybcI | conserved inner membrane protein | GCO | 86.98% | 52.93% |
| NP_459536 | ybcJ | putative cytoplasmic protein | - | NP_415061 | ybcJ | predicted RNA-binding protein | GCO | 92.85% | 57.27% |
| NP_459537 | folD | 5,10-methylene-tetrahydrofolate dehydrogenase/5,10-methylene-tetrahydrofolate cyclohydrolase | - | NP_415062 | folD | bifunctional 5,10-methylene-tetrahydrofolate dehydrogenase/ 5,10-methylene-tetrahydrofolate cyclohydrolase | GCO | 95.80% | 54.44% |
| NP_459538 | fimA | fimbrin | + | NP_415063 | sfmA | predicted fimbrial-like adhesin protein | GCO | 65% | 55.24% |
| NP_459539 | fimI | fimbrial protein | + | NP_418735 | fimI | fimbrial protein involved in type 1 pilus biosynthesis | nGCO | 35.09% | 54.11% |
| NP_459540 | fimC | periplasmic chaperone | + | NP_415064 | sfmC | pilin chaperone, periplasmic | GCO | 61.57% | 50.36% |
| NP_459541 | fimD | outer membrane usher protein precursor | + | NP_415065 | sfmD | predicted outer membrane export usher protein | GCO | 69.29% | 55.68% |
| NP_459542 | fimH | minor fimbrial subunit | + | NP_415066 | sfmH | predicted fimbrial-like adhesin protein | GCO | 72.20% | 51.68% |
| NP_459543 | fimF | putative fimbrial protein | + | NP_415067 | sfmF | predicted fimbrial-like adhesin protein | GCO | 49.70% | 55.10% |
| NP_459544 | fimZ | fimbrial protein Z | + | NP_415068 | fimZ | predicted DNA-binding transcriptional regulator | GCO | 71.90% | 41.07% |
| NP_459545 | fimY | putative regulatory protein | - |  |  |  | NO HOMOLOG |  | 46.47% |
| NP_459546 | - | hypothetical protein | + |  |  |  | NO HOMOLOG |  | 42.62% |
| NP_459547 | fimW | putative fimbrial protein | + |  |  |  | NO HOMOLOG |  | 42.04% |
| NP_459548 | - | integrase | - |  |  |  | NO HOMOLOG |  | 44.44% |
| NP_459550 | - | putative inner membrane protein | - |  |  |  | NO HOMOLOG |  | 36.78% |
| NP_459551 | yfdH | putative glycosyltransferase | - | NP_416852 | yfdH | CPS-53 (KpLE1) prophage; bactoprenol glucosyl transferase | GCO | 80.71% | 42.28% |
| NP_459552 | rfbI | putative glycosyl translocase | - | NP_416851 | yfdG | CPS-53 (KpLE1) prophage; bactoprenol-linked glucose translocase (flippase) | GCO | 81.66% | 46.55% |
| NP_459553 | - | sensor kinase | - |  |  |  | NO HOMOLOG |  | 49.39% |
| NP_459555 | - | putative transcriptional regulator | - | NP_414839 | ykgD | predicted DNA-binding transcriptional regulator | GCO | 71.63% | 58.24% |
| NP_459556 | - | pyridine nucleotide-disulfide oxidoreductase | - | NP_414838 | ykgC | pyridine nucleotide-disulfide oxidoreductase | GCO | 69.38% | 52.41% |
| NP_459557 | - | putative periplasmic protein | - | NP_414837 | ykgI | hypothetical protein | GCO | 62.74% | 45.99% |
| NP_459558 | - | putative inner membrane protein | - | NP_414835 | ykgB | conserved inner membrane protein | GCO | 84.32% | 51.33% |
| NP_459559 | - | putative DNA repair ATPase | - |  |  |  | NO HOMOLOG |  | 53.74% |
| NP_459560 | pheP | phenylalanine transporter | - | NP_415108 | pheP | phenylalanine transporter | GCO | 83.33% | 53.18% |
| NP_459561 | ybdG | hypothetical protein | - | NP_415109 | ybdG | predicted mechanosensitive channel | GCO | 85.99% | 49.51% |
| NP_459562 | apeE | outer membrane esterase | - |  |  |  | NO HOMOLOG |  | 55.70% |
| NP_459563 | - | putative inner membrane protein | - |  |  |  | NO HOMOLOG |  | 51.61% |
| NP_459564 | - | putative phosphosugar isomerase | - | NP_417830 | frlB | fructoselysine-6-P-deglycase | nGCO | 26.25% | 52.58% |
| NP_459565 | - | putative inner membrane protein | - | NP_418185 | glmS | D-fructose-6-phosphate amidotransferase | nGCO | 20.70% | 58.14% |
| NP_459566 | - | putative PTS system mannose-specific enzyme IID | - | NP_416333 | manZ | mannose-specific enzyme IID component of PTS | GCO | 34.70% | 56.95% |
| NP_459567 | - | putative inner membrane protein | - | NP_416332 | manY | mannose-specific enzyme IIC component of PTS | GCO | 34.71% | 57.25% |
| NP_459568 | - | putative PTS system mannose-specific enzyme IIAB | - | NP_417607 | agaB | N-acetylgalactosamine-specific enzyme IIB component of PTS | nGCO | 24.67% | 55.62% |
| NP_459569 | - | putative PTS system mannose-specific enzyme IIAB | - |  |  |  | NO HOMOLOG |  | 52.22% |
| NP_459570 | nfnB | dihydropteridine reductase/oxygen-insensitive NAD(P)H nitroreductase | - | NP_415110 | nfnB | dihydropteridine reductase, NAD(P)H-dependent, oxygen-insensitive | GCO | 88.47% | 54.43% |
| NP_459571 | ybdF | putative cytoplasmic protein | - | NP_415111 | ybdF | hypothetical protein | GCO | 75.20% | 50.94% |
| NP_459572 | - | putative regulatory protein | - |  |  |  | NO HOMOLOG |  | 52.74% |
| NP_459573 | - | putative regulatory protein | - | NP_416048 | marA | DNA-binding transcriptional dual activator of multiple antibiotic resistance | nGCO | 39.60% | 50% |
| NP_459574 | ybdJ | putative inner membrane protein | - | NP_415112 | ybdJ | predicted inner membrane protein | GCO | 41.46% | 51.00% |
| NP_459575 | ybdK | putative cytoplasmic protein | - | NP_415113 | ybdK | gamma-glutamyl:cysteine ligase | GCO | 81.94% | 54.15% |
| NP_459576 | entD | enterochelin synthetase component D | - | NP_415115 | entD | phosphopantetheinyltransferase component of enterobactin synthase multienzyme complex | GCO | 51.91% | 55.88% |
| NP_459577 | fepA | outer membrane ferric enterobactin receptor precursor | - | NP_415116 | fepA | iron-enterobactin outer membrane transporter | GCO | 78.54% | 53.98% |
| NP_459578 | fes | enterochelin esterase | - | NP_415117 | fes | enterobactin/ferric enterobactin esterase | GCO | 73.92% | 60.08% |
| NP_459579 | ybdZ | putative cytoplasmic protein | - | YP_588441 | ybdZ | hypothetical protein | GCO | 66.66% | 54.79% |
| NP_459580 | entF | enterobactin synthetase component F | - | NP_415118 | entF | enterobactin synthase multienzyme complex component, ATP-dependent | GCO | 76.50% | 59.38% |
| NP_459581 | fepE | ferric enterobactin/enterochelin transporter | - | NP_415119 | fepE | regulator of length of O-antigen component of lipopolysaccharide chains | GCO | 67.56% | 48.90% |
| NP_459582 | fepC | enterobactin transporter | - | NP_415120 | fepC | iron-enterobactin transporter subunit | GCO | 88.21% | 56.85% |
| NP_459583 | fepG | ferric enterobactin transporter | - | NP_415121 | fepG | iron-enterobactin transporter subunit | GCO | 77.20% | 60.10% |
| NP_459584 | fepD | ferric enterobactin/enterochelin transporter | - | NP_415122 | fepD | iron-enterobactin transporter subunit | GCO | 72.37% | 61.21% |
| NP_459585 | ybdA | putative transport protein | - | NP_415123 | ybdA | predicted transporter | GCO | 66.99% | 58.87% |
| NP_459586 | fepB | ferric enterobactin transporter | - | NP_415124 | fepB | iron-enterobactin transporter subunit | GCO | 75.47% | 57.99% |
| NP_459587 | entC | isochorismate synthase | - | NP_415125 | entC | isochorismate synthase | GCO | 84.65% | 58.24% |
| NP_459588 | entE | 2,3-dihydroxybenzoate-AMP ligase | - | NP_415126 | entE | 2,3-dihydroxybenzoate-AMP ligase component of enterobactin synthase multienzyme complex | GCO | 86.32% | 57.41% |
| NP_459589 | entB | 2,3-dihydro-2,3-dihydroxybenzoate synthetase | - | NP_415127 | entB | isochorismatase | GCO | 84.56% | 56.87% |
| NP_459590 | entA | 2,3-dihydroxybenzoate-2,3-dehydrogenase | - | NP_415128 | entA | 2,3-dihydroxybenzoate-2,3-dehydrogenase | GCO | 80.56% | 58.59% |
| NP_459591 | ybdB | hypothetical protein | - | NP_415129 | ybdB | hypothetical protein | GCO | 82.48% | 60.14% |
| NP_459592 | cstA | carbon starvation protein | - | NP_415130 | cstA | carbon starvation protein | GCO | 88.58% | 56.93% |
| NP_459593 | ybdD | putative cytoplasmic protein | - | YP_588442 | ybdD | hypothetical protein | GCO | 90.76% | 52.52% |
| NP_459594 | ybdH | putative glycerol dehydrogenase | - | NP_415132 | ybdH | predicted oxidoreductase | GCO | 79.50% | 57.85% |
| NP_459595 | ybdL | putative aminotransferase | - | NP_415133 | ybdL | putative aminotransferase | GCO | 85.49% | 54.26% |
| NP_459596 | ybdM | putative transcriptional regulator | + | NP_415134 | ybdM | hypothetical protein | GCO | 77.07% | 51.45% |
| NP_459597 | ybdN | putative 3'-phosphoadenosine 5'-phosphosulfate sulfotransferase | + | NP_415135 | ybdN | hypothetical protein | GCO | 82.06% | 48.94% |
| NP_459598 | ybdO | putative transcriptional regulator | + | NP_415136 | ybdO | predicted DNA-binding transcriptional regulator | GCO | 54.36% | 38.98% |
| NP_459599 | dsbG | periplasmic disulfide isomerase/thiol-disulphide oxidase | - | NP_415137 | dsbG | periplasmic disulfide isomerase/thiol-disulphide oxidase | GCO | 75.60% | 50.73% |
| NP_459600 | ahpC | alkyl hydroperoxide reductase C22 subunit | - | NP_415138 | ahpC | alkyl hydroperoxide reductase, C22 subunit | GCO | 98.39% | 50.88% |
| NP_459601 | ahpF | alkyl hydroperoxide reductase F52a subunit | - | NP_415139 | ahpF | alkyl hydroperoxide reductase, F52a subunit, FAD/NAD(P)-binding | GCO | 90.21% | 54.91% |
| NP_459602 | - | putative anaerobic dehydrogenase component | - | NP_415518 | torD | chaperone protein TorD | nGCO | 28.80% | 43.31% |
| NP_459603 | - | putative oxidoreductase protein | - | NP_416104 | ynfE | oxidoreductase subunit | nGCO | 28.78% | 54.73% |
| NP_459604 | - | putative hydrogenase protein | - | NP_415415 | dmsB | dimethyl sulfoxide reductase, anaerobic, subunit B | nGCO | 41.33% | 53.76% |
| NP_459605 | - | putative hydrogenase protein | - | NP_416107 | ynfH | oxidoreductase, membrane subunit | nGCO | 28.50% | 55.07% |
| NP_459606 | ybdQ | putative universal stress protein | - | NP_415140 | uspG | universal stress protein UP12 | GCO | 89.43% | 51.98% |
| NP_459607 | ybdR | putative dehydrogenase | - | NP_415141 | ybdR | predicted oxidoreductase, Zn-dependent and NAD(P)-binding | GCO | 87.37% | 54.31% |
| NP_459608 | rnk | nucleoside diphosphate kinase regulator | - | NP_415143 | rnk | nucleoside diphosphate kinase regulator | GCO | 91.85% | 58.39% |
| NP_459609 | rna | RNase I | - | NP_415144 | rna | ribonuclease I | GCO | 73.13% | 54.77% |
| NP_459610 | citT | citrate/succinate transport antiport protein | - | NP_415145 | citT | citrate:succinate antiporter | GCO | 91.78% | 53.68% |
| NP_459611 | citG | triphosphoribosyl-dephospho-CoA synthase | - | NP_415146 | citG | triphosphoribosyl-dephospho-CoA transferase | GCO | 71.23% | 60.64% |
| NP_459612 | citX | 2'-(5'-triphosphoribosyl)-3'-dephospho-CoA:apo-citrate lyase | - | NP_415147 | citX | 2'-(5'-triphosphoribosyl)-3'-dephospho-CoA:apo-citrate lyase | GCO | 75.41% | 61.95% |
| NP_459613 | citF | citrate lyase alpha chain/citrate-ACP transferase | - | NP_415148 | citF | citrate lyase, citrate-ACP transferase (alpha) subunit | GCO | 88.62% | 58.36% |
| NP_459614 | citE | citrate lyase beta chain | - | NP_415149 | citE | citrate lyase, citryl-ACP lyase (beta) subunit | GCO | 88.41% | 59.51% |
| NP_459615 | citD | citrate lyase acyl carrier protein gamma chain | - | NP_415150 | citD | citrate lyase, acyl carrier (gamma) subunit | GCO | 79.59% | 56.90% |
| NP_459616 | citC | citrate lyase synthetase | - | NP_415151 | citC | citrate lyase synthetase | GCO | 86.89% | 51.62% |
| NP_459617 | dpiB | sensory histidine kinase | - | NP_415152 | citA | sensory histidine kinase in two-component regulatory system with citB | GCO | 83.88% | 51.86% |
| NP_459618 | dpiA | response regulator | + | NP_415153 | citB | DNA-binding response regulator in two-component regulatory system with citA | GCO | 84.07% | 53.74% |
| NP_459619 | dcuC | dicarboxylate transporter | - | NP_415154 | dcuC | anaerobic C4-dicarboxylate transport | GCO | 74.94% | 53.75% |
| NP_459620 | pagP | PhoPQ-activated gene | - | NP_415155 | crcA | palmitoyl transferase for Lipid A | GCO | 75.26% | 49.38% |
| NP_459621 | cspE | cold shock protein E | - | NP_415156 | cspE | cold shock protein E | GCO | 98.55% | 43.80% |
| NP_459622 | ccrB | camphor resistance protein CrcB | - | NP_415157 | crcB | camphor resistance protein CrcB | GCO | 92.10% | 52.60% |
| NP_459623 | ybeM | putative hydrolase | - | NP_414754 | yafV | predicted C-N hydrolase family amidase, NAD(P)-binding | nGCO | 26.87% | 56.40% |
| NP_459624 | tatE | twin argininte translocase protein A | - | NP_415160 | tatE | twin arginine translocase protein E | GCO | 70.14% | 49.50% |
| NP_459625 | lipA | lipoyl synthase | - | NP_415161 | lipA | lipoyl synthase | GCO | 97.19% | 54.86% |
| NP_459626 | ybeF | putative transcriptional regulator | + | NP_415162 | ybeF | predicted DNA-binding transcriptional regulator | GCO | 68.76% | 50.94% |
| NP_459627 | lipB | lipoyltransferase | - | NP_415163 | lipB | lipoyltransferase | GCO | 84.03% | 49.84% |
| NP_459628 | ybeD | hypothetical protein | - | NP_415164 | ybeD | hypothetical protein | GCO | 97.70% | 46.21% |
| NP_459629 | dacA | D-alanyl-D-alanine carboxypeptidase | - | NP_415165 | dacA | D-alanyl-D-alanine carboxypeptidase (penicillin-binding protein 5) | GCO | 95.03% | 52.47% |
| NP_459630 | rlpA | minor lipoprotein | - | NP_415166 | rlpA | minor lipoprotein | GCO | 70.34% | 60.55% |
| NP_459631 | mrdB | rod shape-determining membrane protein | - | NP_415167 | mrdB | cell wall shape-determining protein | GCO | 78.91% | 53.00% |
| NP_459632 | mrdA | cell elongation-specific transpeptidase | - | NP_415168 | mrdA | transpeptidase involved in peptidoglycan synthesis (penicillin-binding protein 2) | GCO | 96.20% | 53.83% |
| NP_459633 | ybeA | hypothetical protein | - | NP_415169 | ybeA | hypothetical protein | GCO | 98.70% | 57.47% |
| NP_459634 | ybeB | hypothetical protein | - | NP_415170 | ybeB | hypothetical protein | GCO | 97.14% | 50.62% |
| NP_459635 | cobC | alpha ribazole-5'-P phosphatase | - | NP_415171 | cobC | predicted alpha-ribazole-5'-P phosphatase | GCO | 74.24% | 57.79% |
| NP_459636 | cobD | threonine-phosphate decarboxylase | - | NP_416525 | hisC | histidinol-phosphate aminotransferase | nGCO | 25.66% | 56.62% |
| NP_459637 | nadD | nicotinic acid mononucleotide adenyltransferase | - | NP_415172 | nadD | nicotinic acid mononucleotide adenyltransferase | GCO | 85.44% | 54.82% |
| NP_459638 | holA | DNA polymerase III subunit delta | - | NP_415173 | holA | DNA polymerase III subunit delta | GCO | 81.63% | 57.65% |
| NP_459639 | rlpB | minor lipoprotein | - | NP_415174 | rlpB | minor lipoprotein | GCO | 67.34% | 55.16% |
| NP_459640 | leuS | leucyl-tRNA synthetase | - | NP_415175 | leuS | leucyl-tRNA synthetase | GCO | 94.06% | 55.78% |
| NP_459641 | - | putative hydrolase | - |  |  |  | NO HOMOLOG |  | 49.48% |
| NP_459642 | - | putative hydrolas | - | NP_417597 | garD | (D)-galactarate dehydrogenase | nGCO | 33.91% | 52.60% |
| NP_459643 | - | 2-keto-3-deoxygluconate permease | - | NP_418345 | kdgT | 2-keto-3-deoxygluconate permease | nGCO | 31.44% | 52.30% |
| NP_459644 | - | putative sigma-54 dependent transcriptional regulator | - | NP_415839 | tyrR | DNA-binding transcriptional dual regulator, tyrosine-binding | nGCO | 32.35% | 50.07% |
| NP_459645 | ybeL | putative cytoplasmic protein | - | NP_415176 | ybeL | hypothetical protein | GCO | 91.25% | 55.69% |
| NP_459646 | ybeQ | tetratricopeptide repeat protein | + | NP_415177 | ybeQ | hypothetical protein | GCO | 49.84% | 47.48% |
| NP_459647 | ybeR | putative cytoplasmic protein | + | NP_415178 | ybeR | hypothetical protein | GCO | 62.97% | 52.96% |
| NP_459648 | ybeS | putative molecular chaperone | - | NP_415182 | djlC | Hsc56 co-chaperone of HscC | GCO | 47.29% | 54.88% |
| NP_459649 | ybeU | putative cytoplasmic protein | - | NP_415181 | ybeU | predicted tRNA ligase | GCO | 61.70% | 51.14% |
| NP_459650 | ybeV | putative molecular chaperone | - | NP_415182 | djlC | Hsc56 co-chaperone of HscC | GCO | 57.14% | 54.10% |
| NP_459651 | hscC | putative heatshock protein | - | NP_415183 | hscC | Hsp70 family chaperone Hsc62, binds to RpoD and inhibits transcription | GCO | 83.09% | 52.55% |
| NP_459652 | - | putative cytoplasmic protein | - |  |  |  | NO HOMOLOG |  | 54.07% |
| NP_459653 | ybeK | putative purine nucleoside hydrolase | - | NP_415184 | rihA | ribonucleoside hydrolase 1 | GCO | 92.60% | 58.54% |
| NP_459654 | gltL | glutamate/aspartate transporter | - | NP_415185 | gltL | glutamate and aspartate transporter subunit | GCO | 90.45% | 51.92% |
| NP_459655 | gltK | glutamate/aspartate transporter | - | NP_415186 | gltK | glutamate and aspartate transporter subunit | GCO | 95.51% | 53.77% |
| NP_459656 | gltJ | glutamate/aspartate transporter | - | NP_415187 | gltJ | glutamate and aspartate transporter subunit | GCO | 94.71% | 54.52% |
| NP_459657 | gltI | glutamate/aspartate transporter | - | NP_415188 | gltI | glutamate and aspartate transporter subunit | GCO | 93.37% | 51.13% |
| NP_459658 | lnt | apolipoprotein N-acyltransferase | - | NP_415190 | lnt | apolipoprotein N-acyltransferase | GCO | 84.17% | 54.90% |
| NP_459659 | ybeX | putative transport protein | - | NP_415191 | ybeX | predicteed ion transport | GCO | 92.80% | 51.87% |
| NP_459660 | ybeY | hypothetical protein | - | NP_415192 | ybeY | hypothetical protein | GCO | 85.71% | 54.21% |
| NP_459661 | phoL | putative phosphate starvation-inducible protein | - | NP_415193 | ybeZ | predicted protein with nucleoside triphosphate hydrolase domain | GCO | 96.72% | 53.77% |
| NP_459662 | miaB | rRNA modification protein | - | NP_415194 | miaB | isopentenyl-adenosine A37 tRNA methylthiolase | GCO | 98.31% | 53.89% |
| NP_459663 | ubiF | 2-octaprenyl-3-methyl-6-methoxy-1,4-benzoquinol hydroxylase | - | NP_415195 | ubiF | 2-octaprenyl-3-methyl-6-methoxy-1,4-benzoquinol hydroxylase | GCO | 79.53% | 59.94% |
| NP_459664 | - | putative inner membrane protein | - |  |  |  | NO HOMOLOG |  | 42.88% |
| NP_459665 | asnB | asparagine synthetase B | - | NP_415200 | asnB | asparagine synthetase B | GCO | 94.40% | 54.47% |
| NP_459666 | nagD | putative phosphatase | - | NP_415201 | nagD | UMP phosphatase | GCO | 96.80% | 54.31% |
| NP_459667 | nagC | N-acetylglucosamine operon transcriptional repressor | - | NP_415202 | nagC | DNA-binding transcriptional dual regulator, repressor of N-acetylglucosamine | GCO | 94.33% | 53.39% |
| NP_459668 | nagA | N-acetylglucosamine-6-phosphate deacetylase | - | NP_415203 | nagA | N-acetylglucosamine-6-phosphate deacetylase | GCO | 92.63% | 52.90% |
| NP_459669 | nagB | glucosamine-6-phosphate deaminase | - | NP_415204 | nagB | glucosamine-6-phosphate deaminase | GCO | 95.48% | 50.43% |
| NP_459670 | nagE | N-acetylglucosamine-specific enzyme IIABC | - | NP_415205 | nagE | fused N-acetyl glucosamine specific PTS enzyme: IIC, IIB , and IIA components | GCO | 83.17% | 56.16% |
| NP_459671 | glnS | glutaminyl-tRNA synthetase | - | NP_415206 | glnS | glutaminyl-tRNA synthetase | GCO | 96.03% | 53.41% |
| NP_459672 | ybfM | putative outer membrane protein | - | NP_415207 | ybfM | predicted outer membrane porin | GCO | 91.23% | 51.38% |
| NP_459673 | ybfN | putative lipoprotein | - | NP_415208 | ybfN | predicted lipoprotein | GCO | 84.11% | 52.85% |
| NP_459674 | citA | citrate-proton symporter | - | NP_417082 | kgtP | alpha-ketoglutarate transporter | nGCO | 30.58% | 54.02% |
| NP_459675 | citB | citrate utilization protein b | - |  |  |  | NO HOMOLOG |  | 57.54% |
| NP_459676 | - | hypothetical protein | - |  |  |  | NO HOMOLOG |  | 58.40% |
| NP_459677 | - | putative transcriptional regulator | - | NP_416493 | nac | DNA-binding transcriptional dual regulator of nitrogen assimilation | nGCO | 31.73% | 56.20% |
| NP_459678 | fur | ferric uptake regulator | - | NP_415209 | fur | ferric uptake regulator | GCO | 99.31% | 47.24% |
| NP_459679 | fldA | flavodoxin | - | NP_415210 | fldA | flavodoxin 1 | GCO | 97.72% | 50.65% |
| NP_459680 | ybfE | putative SOS response protein | - | NP_415211 | ybfE | LexA regulated protein | GCO | 90.72% | 52.72% |
| NP_459681 | ybfF | putative enzyme | - | NP_415212 | ybfF | hypothetical protein | GCO | 89.76% | 56.16% |
| NP_459682 | seqA | sequestration protein A | - | NP_415213 | seqA | regulatory protein for replication initiation | GCO | 87.84% | 51.19% |
| NP_459683 | pgm | phosphoglucomutase | - | NP_415214 | pgm | phosphoglucomutase | GCO | 94.87% | 56.18% |
| NP_459684 | - | putative cytoplasmic protein | - |  |  |  | NO HOMOLOG |  | 50.82% |
| NP_459685 | potE | putrescine/ornithine antiporter | - | NP_415219 | potE | putrescine/proton symporter: putrescine/ornithine antiporter | GCO | 94.07% | 54.09% |
| NP_459686 | speF | ornithine decarboxylase isozyme | - | NP_415220 | speF | ornithine decarboxylase isozyme, inducible | GCO | 91.66% | 53.88% |
| NP_459687 | kdpE | response regulator | - | NP_415222 | kdpE | DNA-binding response regulator in two-component regulatory system with KdpD | GCO | 79.01% | 56.34% |
| NP_459688 | kdpD | sensor kinase | - | NP_415223 | kdpD | fused sensory histidine kinase in two-component regulatory system with KdpE: signal sensing protein | GCO | 83.78% | 58.88% |
| NP_459689 | kdpC | potassium-transporting ATPase subunit C | - | NP_415224 | kdpC | potassium-transporting ATPase subunit C | GCO | 69.14% | 60% |
| NP_459690 | kdpB | potassium-transporting ATPase subunit B | - | NP_415225 | kdpB | potassium-transporting ATPase subunit B | GCO | 79.44% | 58.80% |
| NP_459691 | kdpA | potassium-transporting ATPase subunit A | - | NP_415226 | kdpA | potassium-transporting ATPase subunit A | GCO | 73.24% | 57.08% |
| NP_459692 | - | putative outer membrane protein | - |  |  |  | NO HOMOLOG |  | 48.88% |
| NP_459693 | ybfA | putative periplasmic protein | - | NP_415228 | ybfA | hypothetical protein | nGCO | 82.35% | 50.72% |
| NP_459694 | phrB | deoxyribodipyrimidine photolyase | - | NP_415236 | phr | deoxyribodipyrimidine photolyase, FAD-binding | GCO | 76.27% | 56.96% |
| NP_459695 | ybgH | putative POT family transport protein | - | NP_415237 | ybgH | predicted transporter | GCO | 84.58% | 54.79% |
| NP_459696 | ybgI | putative cytoplasmic protein | - | NP_415238 | ybgI | conserved metal-binding protein | GCO | 92.30% | 55.51% |
| NP_459697 | ybgJ | putative carboxylase | - | NP_415239 | ybgJ | predicted enzyme subunit | GCO | 86.69% | 60.73% |
| NP_459698 | ybgK | putative carboxylase | - | NP_415240 | ybgK | predicted enzyme subunit | GCO | 84.83% | 58.30% |
| NP_459699 | ybgL | hypothetical protein | - | NP_415241 | ybgL | hypothetical protein | GCO | 82.06% | 59.31% |
| NP_459700 | - | putative cytoplasmic protein | - |  |  |  | NO HOMOLOG |  | 39.39% |
| NP_459701 | - | putative phage integrase | - | NP_418732 | fimB | tyrosine recombinase/inversion of on/off regulator of fimA | nGCO | 60% | 50.61% |
| NP_459702 | - | putative inner membrane protein | - |  |  |  | NO HOMOLOG |  | 55.35% |
| NP_459703 | - | putative cytoplasmic protein | - |  |  |  | NO HOMOLOG |  | 56.54% |
| NP_459704 | - | putative UDP-galactopyranose mutase | - | NP_416540 | glf | UDP-galactopyranose mutase, FAD/NAD(P)-binding | nGCO | 39.27% | 38.80% |
| NP_459705 | - | putative glycosyl transferase | - |  |  |  | NO HOMOLOG |  | 37.12% |
| NP_459706 | - | putative glycosyl transferase | - |  |  |  | NO HOMOLOG |  | 35.89% |
| NP_459707 | - | putative ABC transporter permease protein | - |  |  |  | NO HOMOLOG |  | 36.05% |
| NP_459708 | - | putative ABC-type polysaccharide/polyol phosphate transport system ATPase component | + | NP_415376 | potG | putrescine transporter subunit: ATP-binding component of ABC superfamily | nGCO | 26.63% | 39.66% |
| NP_459709 | - | putative glycosyltransferase | - |  |  |  | NO HOMOLOG |  | 32.49% |
| NP_459710 | - | putative glycosyltransferase | - |  |  |  | NO HOMOLOG |  | 31.65% |
| NP_459711 | - | putative glycosyl transferase | - |  |  |  | NO HOMOLOG |  | 35.81% |
| NP_459712 | - | putative cytoplasmic protein | - |  |  |  | NO HOMOLOG |  | 50% |
| NP_459713 | nei | endonuclease VIII | - | NP_415242 | nei | endonuclease VIII/ 5-formyluracil/5-hydroxymethyluracil DNA glycosylase | GCO | 88.54% | 52.90% |
| NP_459714 | abrB | putative transport protein | - | NP_415243 | abrB | predicted regulator | GCO | 71.96% | 56.16% |
| NP_459715 | gltA | citrate synthase | - | NP_415248 | gltA | citrate synthase | nGCO | 96.25% | 52.72% |
| NP_459716 | - | putative inner membrane protein | - |  |  |  | NO HOMOLOG |  | 44.44% |
| NP_459717 | sdhC | succinate dehydrogenase cytochrome b556 large membrane subunit | - | NP_415249 | sdhC | succinate dehydrogenase cytochrome b556 large membrane subunit | GCO | 92.24% | 48.46% |
| NP_459718 | sdhD | succinate dehydrogenase cytochrome b556 small membrane subunit | - | NP_415250 | sdhD | succinate dehydrogenase cytochrome b556 small membrane subunit | GCO | 95.34% | 50.57% |
| NP_459719 | sdhA | succinate dehydrogenase catalytic subunit | - | NP_415251 | sdhA | succinate dehydrogenase flavoprotein subunit | GCO | 94.38% | 58.85% |
| NP_459720 | sdhB | succinate dehydrogenase catalytic subunit | - | NP_415252 | sdhB | succinate dehydrogenase, FeS subunit | GCO | 96.63% | 51.11% |
| NP_459721 | sucA | 2-oxoglutarate dehydrogenase | - | NP_415254 | sucA | alpha-ketoglutarate decarboxylase | GCO | 94.74% | 55.85% |
| NP_459722 | sucB | dihydrolipoamide acetyltransferase | - | NP_415255 | sucB | dihydrolipoamide acetyltransferase | GCO | 96.04% | 56.32% |
| NP_459723 | sucC | succinyl-CoA synthetase subunit beta | - | NP_415256 | sucC | succinyl-CoA synthetase subunit beta | GCO | 90.72% | 56.55% |
| NP_459724 | sucD | succinyl-CoA synthetase alpha subunit | - | NP_415257 | sucD | succinyl-CoA synthetase subunit alpha | GCO | 80.62% | 56.66% |
| NP_459725 | cydA | cytochrome d terminal oxidase polypeptide subunit I | - | NP_415261 | cydA | cytochrome d terminal oxidase, subunit I | GCO | 96.16% | 53.72% |
| NP_459726 | cydB | cytochrome d terminal oxidase polypeptide subunit II | - | NP_415262 | cydB | cytochrome d terminal oxidase, subunit II | GCO | 86.01% | 54.91% |
| NP_459727 | ybgT | putative outer membrane lipoprotein | - | YP_588444 | ybgT | hypothetical protein | GCO | 86.11% | 50.87% |
| NP_459728 | ybgE | putative inner membrane lipoprotein | - | NP_415263 | ybgE | conserved inner membrane protein | GCO | 89.88% | 52.12% |
| NP_459729 | ybgC | putative esterase | - | NP_415264 | ybgC | predicted acyl-CoA thioesterase | GCO | 92.53% | 50.37% |
| NP_459730 | tolQ | Tol import system inner membrane protein | - | NP_415265 | tolQ | membrane spanning protein in TolA-TolQ-TolR complex | GCO | 98.26% | 52.38% |
| NP_459731 | tolR | Tol import sytem inner membrane protein | - | NP_415266 | tolR | membrane spanning protein in TolA-TolQ-TolR complex | GCO | 76.76% | 51.04% |
| NP_459732 | tolA | cell envelope integrity inner membrane protein TolA | - |  |  |  | NO HOMOLOG |  | 58.82% |
| NP_459733 | tolB | translocation protein TolB precursor | - | NP_415268 | tolB | translocation protein TolB precursor | GCO | 95.58% | 55.68% |
| NP_459734 | pal | peptidoglycan-associated lipoprotein precursor | - | NP_415269 | pal | peptidoglycan-associated outer membrane lipoprotein | GCO | 89.71% | 53.71% |
| NP_459735 | ybgF | putative periplasmic protein | - | NP_415270 | ybgF | hypothetical protein | GCO | 73.76% | 52.34% |
| NP_459736 | nadA | quinolinate synthetase | - | NP_415271 | nadA | quinolinate synthetase | GCO | 89.01% | 55.26% |
| NP_459737 | pnuC | nucleoside/purine/pyrimidine transporter | - | NP_415272 | pnuC | predicted nicotinamide mononucleotide transporter | GCO | 82.35% | 50.69% |
| NP_459738 | ybgR | zinc transporter ZitB | - | NP_415273 | zitB | zinc transporter ZitB | GCO | 74.35% | 54.95% |
| NP_459739 | ybgS | hypothetical protein | - | NP_415274 | ybgS | hypothetical protein | GCO | 65.47% | 49.35% |
| NP_459740 | aroG | 3-deoxy-7-phosphoheptulonate synthase | - | NP_415275 | aroG | 3-deoxy-D-arabino-heptulosonate-7-phosphate synthase, phenylalanine repressible | GCO | 91.71% | 53.56% |
| NP_459741 | - | fumarate hydratase | + | NP_417534 | ttdB | L(+)-tartrate dehydratase | GCO | 30.27% | 42.49% |
| NP_459742 | - | fumarate hydratase | + | NP_417533 | ttdA | tartrate dehydratase | GCO | 27.58% | 40.89% |
| NP_459743 | - | transcriptional regulator | + | NP_414872 | cynR | DNA-binding transcriptional dual regulator | nGCO | 25.61% | 34.90% |
| NP_459744 | - | transcriptional regulator | + | NP_418747 | yjiE | predicted DNA-binding transcriptional regulator | nGCO | 29.60% | 39.53% |
| NP_459745 | - | putative cation transporter | - | NP_417959 | arsB | arsenite/antimonite transporter | nGCO | 20.96% | 38.06% |
| NP_459746 | dcoC | putative oxaloacetate decarboxylase subunit gamma | - |  |  |  | NO HOMOLOG |  | 61.78% |
| NP_459747 | dcoB | oxaloacetate decarboxylase beta chain | - |  |  |  | NO HOMOLOG |  | 64.13% |
| NP_459748 | - | putative cytoplasmic protein | - | NP_415146 | citG | triphosphoribosyl-dephospho-CoA transferase | nGCO | 30.23% | 59.65% |
| NP_459749 | - | putative ABC transport protein | - | NP_418708 | fecD | KpLE2 phage-like element; iron-dicitrate transporter subunit | nGCO | 27.47% | 55.09% |
| NP_459750 | - | putative ABC-type cobalamin/Fe3+-siderophore transport component | - | NP_415120 | fepC | iron-enterobactin transporter subunit | nGCO | 31.30% | 56.37% |
| NP_459751 | gpmA | phosphoglyceromutase | - | NP_415276 | gpmA | phosphoglyceromutase | GCO | 98.27% | 51.79% |
| NP_459752 | galM | galactose-1-epimerase | - | NP_415277 | galM | galactose-1-epimerase (mutarotase) | GCO | 88.37% | 56.58% |
| NP_459753 | galK | galactokinase | - | NP_415278 | galK | galactokinase | GCO | 86.91% | 57.00% |
| NP_459754 | galT | galactose-1-phosphate uridylyltransferase | - | NP_415279 | galT | galactose-1-phosphate uridylyltransferase | GCO | 93.39% | 58.45% |
| NP_459755 | galE | UDP-galactose 4-epimerase | - | NP_415280 | galE | UDP-galactose-4-epimerase | GCO | 95.85% | 55.85% |
| NP_459756 | - | putative inner membrane protein | + |  |  |  | NO HOMOLOG |  | 46.86% |
| NP_459757 | modF | putative molybdenum transporter | - | NP_415281 | modF | fused molybdate transporter subunits of ABC superfamily: ATP-binding components | GCO | 86.80% | 55.42% |
| NP_459758 | modE | molybdate uptake operon transcriptional repressor | - | NP_415282 | modE | DNA-binding transcriptional dual regulator | GCO | 79.77% | 57.28% |
| NP_459759 | - | hypothetical protein | - | NP_415283 | ybhT | hypothetical protein | GCO | 62.50% | 46.66% |
| NP_459760 | modA | molybdate transporter | - | NP_415284 | modA | molybdate transporter subunit | GCO | 86.77% | 55.03% |
| NP_459761 | modB | molybdate transport permease protein | - | NP_415285 | modB | molybdate ABC transporter permease protein | GCO | 68.12% | 59.42% |
| NP_459762 | modC | molybdate transporter | - | NP_415286 | modC | molybdate transporter subunit | GCO | 86.64% | 55.90% |
| NP_459763 | ybhA | putative hydrolase | - | NP_415287 | ybhA | predicted hydrolase | GCO | 84.19% | 53.23% |
| NP_459764 | ybhE | putative 3-carboxymuconate cyclase | - | NP_415288 | ybhE | 6-phosphogluconolactonase | GCO | 87.31% | 55.72% |
| NP_459765 | ybhC | putative pectinesterase | - | NP_415293 | ybhC | predicted pectinesterase | nGCO | 87.11% | 57.24% |
| NP_459766 | hutI | imidazolonepropionase | - |  |  |  | NO HOMOLOG |  | 58.33% |
| NP_459767 | hutG | formimidoylglutamase | - |  |  |  | NO HOMOLOG |  | 61.67% |
| NP_459768 | hutC | histidine utilization repressor | - | NP_415258 | mngR | DNA-binding transcriptional dual regulator, fatty-acyl-binding | nGCO | 24.77% | 56.88% |
| NP_459769 | hutH | histidine ammonia-lyase | - |  |  |  | NO HOMOLOG |  | 60.35% |
| NP_459770 | ybhB | putative phospholipid-binding protein | - | NP_415294 | ybhB | predicted kinase inhibitor | GCO | 86.70% | 58.49% |
| NP_459771 | bioA | adenosylmethionine--8-amino-7-oxononanoate transaminase | - | NP_415295 | bioA | adenosylmethionine--8-amino-7-oxononanoate transaminase | GCO | 86.88% | 58.68% |
| NP_459772 | bioB | biotin synthetase | - | NP_415296 | bioB | biotin synthase | GCO | 91.04% | 55.13% |
| NP_459773 | bioF | 8-amino-7-oxononanoate synthase | - | NP_415297 | bioF | 8-amino-7-oxononanoate synthase | GCO | 73.95% | 60.44% |
| NP_459774 | bioC | biotin biosynthetic protein | - | NP_415298 | bioC | predicted methltransferase, enzyme of biotin synthesis | GCO | 70.51% | 59.78% |
| NP_459775 | bioD | dithiobiotin synthetase | - | NP_415299 | bioD | dethiobiotin synthetase | GCO | 75.11% | 59.09% |
| NP_459776 | uvrB | excinuclease ABC subunit B | - | NP_415300 | uvrB | excinuclease ABC subunit B | GCO | 93.01% | 55.34% |
| NP_459778 | slrP | leucine-rich repeat protein | - |  |  |  | NO HOMOLOG |  | 45.99% |
| NP_459779 | ybhK | putative cytoplasmic protein | - | NP_415301 | ybhK | predicted transferase with NAD(P)-binding Rossmann-fold domain | GCO | 83.44% | 54.34% |
| NP_459780 | moaA | molybdenum cofactor biosynthesis protein A | - | NP_415302 | moaA | molybdenum cofactor biosynthesis protein A | GCO | 92.40% | 54.14% |
| NP_459781 | moaB | molybdopterin biosynthetic protein B | - | NP_415303 | moaB | molybdopterin biosynthesis protein B | GCO | 95.88% | 55.55% |
| NP_459782 | moaC | molybdenum cofactor biosynthesis protein C | - | NP_415304 | moaC | molybdenum cofactor biosynthesis protein C | GCO | 94.40% | 55.55% |
| NP_459783 | moaD | molybdopterin biosynthetic protein | - | NP_415305 | moaD | molybdopterin synthase, small subunit | GCO | 86.41% | 57.53% |
| NP_459784 | moaE | molybdopterin converting factor subunit 2 | - | NP_415306 | moaE | molybdopterin synthase, large subunit | GCO | 91.33% | 60.92% |
| NP_459785 | ybhL | putative permease | - | NP_415307 | ybhL | predicted inner membrane protein | GCO | 87.17% | 49.78% |
| NP_459786 | ybhM | putative integral membrane protein | - | NP_415308 | ybhM | conserved inner membrane protein | GCO | 29.06% | 43.92% |
| NP_459787 | - | putative inner membrane protein | + |  |  |  | NO HOMOLOG |  | 41.05% |
| NP_459788 | - | putative inner membrane protein | + |  |  |  | NO HOMOLOG |  | 45.45% |
| NP_459789 | ybhN | hypothetical protein | - | NP_415309 | ybhN | conserved inner membrane protein | GCO | 79.87% | 54.82% |
| NP_459790 | ybhO | cardiolipin synthase | - | NP_415310 | ybhO | cardiolipin synthase 2 | GCO | 83.29% | 54.34% |
| NP_459791 | ybhP | putative cytoplasmic protein | - | NP_415311 | ybhP | predicted DNase | GCO | 86.56% | 56.52% |
| NP_459792 | ybhQ | putative inner membrane protein | - | NP_415312 | ybhQ | predicted inner membrane protein | GCO | 75% | 56.44% |
| NP_459793 | ybhR | putative transport protein | - | NP_415313 | ybhR | predicted transporter subunit: membrane component of ABC superfamily | GCO | 92.11% | 52.84% |
| NP_459794 | ybhS | putative transport protein | - | NP_415314 | ybhS | predicted transporter subunit: membrane component of ABC superfamily | GCO | 88.17% | 54.46% |
| NP_459795 | ybhF | putative ABC-type multidrug transport system ATPase component | - | NP_415315 | ybhF | fused predicted transporter subunits of ABC superfamily: ATP-binding components | GCO | 91.34% | 55.84% |
| NP_459796 | - | hypothetical protein | - | NP_415316 | ybhG | hypothetical protein | GCO | 72.80% | 57.83% |
| NP_459797 | ybiH | putative transcriptional repressor | - | NP_415317 | ybiH | predicted DNA-binding transcriptional regulator | GCO | 85.20% | 53.18% |
| NP_459798 | rhlE | putative ATP-dependent RNA helicase | - | NP_415318 | rhlE | RNA helicase | GCO | 84.97% | 58.31% |
| NP_459799 | dinG | SOS repair enzyme | - | NP_415320 | dinG | ATP-dependent DNA helicase | GCO | 92.30% | 54.91% |
| NP_459800 | ybiB | hypothetical protein | - | NP_415321 | ybiB | hypothetical protein | GCO | 84.78% | 57.02% |
| NP_459801 | ybiJ | putative periplasmic protein | - | NP_415323 | ybiJ | hypothetical protein | GCO | 63.95% | 51.72% |
| NP_459802 | ybiI | hypothetical protein | - | NP_415324 | ybiI | hypothetical protein | GCO | 80.68% | 52.05% |
| NP_459803 | ybiN | putative SAM-dependent methyltransferase | - | NP_415328 | ybiN | predicted SAM-dependent methyltransferase | GCO | 81.49% | 54.69% |
| NP_459804 | ybiO | putative transport protein | - | NP_415329 | ybiO | predicted mechanosensitive channel | GCO | 81.40% | 54.38% |
| NP_459805 | glnQ | glutamine ABC transporter ATP-binding component | - | NP_415330 | glnQ | glutamine ABC transporter ATP-binding protein | GCO | 91.25% | 52.28% |
| NP_459806 | glnP | glutamine ABC transporter permease component | - | NP_415331 | glnP | glutamine ABC transporter permease protein | GCO | 84.01% | 53.18% |
| NP_459807 | glnH | glutamine ABC transporter periplasmic-binding protein | - | NP_415332 | glnH | glutamine ABC transporter periplasmic protein | GCO | 96.77% | 48.86% |
| NP_459808 | dps | DNA protection during starvation conditions | - | NP_415333 | dps | DNA protection during starvation conditions | GCO | 95.20% | 48.21% |
| NP_459809 | ybiF | putative permease | - | NP_415334 | rhtA | threonine and homoserine efflux system | GCO | 79.93% | 56.19% |
| NP_459810 | ompX | outer membrane protein X | - | NP_415335 | ompX | outer membrane protein X | GCO | 77.77% | 51.74% |
| NP_459811 | ybiP | putative Integral membrane protein | - | NP_415336 | ybiP | predicted hydrolase, inner membrane | GCO | 83.65% | 51.80% |
| NP_459812 | - | putative Mn-dependent transcriptional regulator | - | NP_415338 | mntR | DNA-binding transcriptional regulator of mntH | GCO | 92.15% | 52.95% |
| NP_459813 | ybiR | putative transporter | - | NP_415339 | ybiR | predicted transporter | GCO | 76.98% | 54.71% |
| NP_459814 | ybiS | putative periplasmic protein | - | NP_415340 | ybiS | hypothetical protein | GCO | 90.52% | 55.37% |
| NP_459815 | ybiT | putative ABC transporter ATPase component | - | NP_415341 | ybiT | fused predicted transporter subunits of ABC superfamily: ATP-binding components | GCO | 96.98% | 53.29% |
| NP_459816 | - | putative inner membrane protein | + |  |  |  | NO HOMOLOG |  | 43.54% |
| NP_459817 | ybiV(2) | putative hydrolase | - | NP_415343 | ybiV | predicted hydrolase | GCO | 93.67% | 46.80% |
| NP_459818 | ybiU | putative cytoplasmic protein | - | NP_415342 | ybiU | hypothetical protein | GCO | 81.71% | 58.53% |
| NP_459819 | ybiV(1) | putative hydrolase | - | NP_415343 | ybiV | predicted hydrolase | GCO | 88.84% | 49.38% |
| NP_459820 | pflF | putative pyruvate formate lyase | - | NP_415344 | ybiW | predicted pyruvate formate lyase | GCO | 91.60% | 55.65% |
| NP_459821 | pflE | putative pyruvate formate lyase activating enzyme | - | NP_415345 | ybiY | predicted pyruvate formate lyase activating enzyme | GCO | 87.95% | 55.55% |
| NP_459822 | moeB | molybdopterin biosynthesis protein MoeB | - | NP_415347 | moeB | molybdopterin biosynthesis protein MoeB | GCO | 85.14% | 57.46% |
| NP_459823 | moeA | molybdopterin biosynthesis protein | - | NP_415348 | moeA | molybdopterin biosynthesis protein | GCO | 84.91% | 57.97% |
| NP_459824 | ybiK | putative asparaginase | - | NP_415349 | iaaA | L-asparaginase | GCO | 88.13% | 60.19% |
| NP_459825 | yliA | putative ABC transporter ATPase components | - | NP_415350 | yliA | fused predicted peptide transport subunits of ABC superfamily: ATP-binding components | GCO | 84.28% | 57.53% |
| NP_459826 | yliB | putative ABC transporter periplasmic binding protein | - | NP_415351 | yliB | predicted peptide transporter subunit: periplasmic-binding component of ABC superfamily | GCO | 89.89% | 53.28% |
| NP_459827 | yliC | putative ABC transporter periplasmic binding protein | - | NP_415352 | yliC | predicted peptide transporter subunit: membrane component of ABC superfamily | GCO | 83.33% | 54.61% |
| NP_459828 | yliD | putative ABC transporter inner membrane component | - | NP_415353 | yliD | predicted peptide transporter subunit: membrane component of ABC superfamily | GCO | 91.41% | 55.48% |
| NP_459829 | yliG | putative FeS oxidoreductase | - | NP_415356 | yliG | predicted SAM-dependent methyltransferase | GCO | 95.01% | 54.14% |
| NP_459830 | yliH | putative cytoplasmic protein | - | NP_415357 | yliH | hypothetical protein | GCO | 70.96% | 50.26% |
| NP_459831 | - | putative cytoplasmic protein | + |  |  |  | NO HOMOLOG |  | 38.95% |
| NP_459832 | - | putative electron transfer protein beta subunit | + | NP_414583 | fixA | predicted electron transfer flavoprotein subunit, ETFP adenine nucleotide-binding domain | nGCO | 27.42% | 37.77% |
| NP_459833 | - | putative electron transfer protein alpha subunit | + | NP_416213 | ydiR | predicted electron transfer flavoprotein, FAD-binding | nGCO | 29.43% | 41.45% |
| NP_459834 | - | putative acyl-CoA dehydrogenase | + | NP_416210 | ydiO | predicted acyl-CoA dehydrogenase | nGCO | 28.06% | 43.12% |
| NP_459835 | - | putative dehydrogenase | + | NP_417246 | ygcN | predicted oxidoreductase with FAD/NAD(P)-binding domain | nGCO | 27.87% | 43.23% |
| NP_459836 | - | putative transcriptional regulator | + | NP_415289 | ybhD | predicted DNA-binding transcriptional regulator | nGCO | 23.86% | 40.17% |
| NP_459837 | - | putative inner membrane protein | - |  |  |  | NO HOMOLOG |  | 43.89% |
| NP_459839 | yliJ | putative glutathione S-transferase | - | NP_415359 | yliJ | predicted glutathione S-transferase | GCO | 78.36% | 54.38% |
| NP_459840 | dacC | D-alanyl-D-alanine carboxypeptidase | - | NP_415360 | dacC | D-alanyl-D-alanine carboxypeptidase (penicillin-binding protein 6a) | GCO | 92.25% | 53.94% |
| NP_459841 | deoR | deoxyribose operon transcriptional repressor | - | NP_415361 | deoR | DNA-binding transcriptional repressor | GCO | 83.33% | 51.91% |
| NP_459842 | ybjG | putative permease | - | NP_415362 | ybjG | undecaprenyl pyrophosphate phosphatase | GCO | 78.21% | 55.17% |
| NP_459843 | mdfA | multidrug translocase | - | NP_415363 | cmr | multidrug efflux system protein | GCO | 87.93% | 55.31% |
| NP_459844 | - | putative hydrolase | - | NP_415365 | ybjI | hypothetical protein | GCO | 75.18% | 51.34% |
| NP_459845 | - | putative transport protein/regulator | - | NP_415366 | ybjJ | predicted transporter | GCO | 81.84% | 57.50% |
| NP_459846 | - | putative regulatory protein | - | NP_415367 | ybjK | predicted DNA-binding transcriptional regulator | GCO | 78.65% | 51.69% |
| NP_459847 | - | hypothetical protein | - | NP_415368 | ybjL | hypothetical protein | GCO | 90.37% | 53.91% |
| NP_459848 | ybjM | putative inner membrane protein | - | NP_415369 | ybjM | predicted inner membrane protein | GCO | 64% | 51.58% |
| NP_459849 | grxA | glutaredoxin 1 | - | NP_415370 | grxA | glutaredoxin 1, redox coenzyme for ribonucleotide reductase (RNR1a) | GCO | 78.57% | 43.18% |
| NP_459850 | ybjC | putative inner membrane protein | - | NP_415371 | ybjC | predicted inner membrane protein | GCO | 43.75% | 53.26% |
| NP_459851 | mdaA | oxygen-insensitive NADPH nitroreductase | - | NP_415372 | nfsA | nitroreductase A, NADPH-dependent, FMN-dependent | GCO | 75.41% | 54.08% |
| NP_459852 | rimK | ribosomal protein S6 modification protein | - | NP_415373 | rimK | ribosomal protein S6 modification protein | GCO | 90.33% | 56.36% |
| NP_459853 | ybjN | putative cytoplasmic protein | - | NP_415374 | ybjN | predicted oxidoreductase | GCO | 77.21% | 50.10% |
| NP_459854 | potF | putrescine transporter | - | NP_415375 | potF | putrescine transporter subunit: periplasmic-binding component of ABC superfamily | GCO | 94.05% | 52.20% |
| NP_459855 | potG | putrescine transporter | + | NP_415376 | potG | putrescine transporter subunit: ATP-binding component of ABC superfamily | GCO | 93.63% | 54.76% |
| NP_459856 | potH | putrescine transporter | - | NP_415377 | potH | putrescine transporter subunit: membrane component of ABC superfamily | GCO | 82.64% | 52.30% |
| NP_459857 | potI | putrescine transporter | - | NP_415378 | potI | putrescine transporter subunit: membrane component of ABC superfamily | GCO | 88.25% | 57.56% |
| NP_459858 | ybjO | putative inner membrane protein | - | NP_415379 | ybjO | predicted inner membrane protein | GCO | 64.96% | 50.42% |
| NP_459859 | ybjF | 23S rRNA (uracil-5-)-methyltransferase | - | NP_415380 | rumB | 23S rRNA m(5)U747-methyltransferase | GCO | 87.90% | 55.79% |
| NP_459860 | ulaA | ascorbate-specific PTS system enzyme IIC | - | NP_418614 | ulaA | ascorbate-specific PTS system enzyme IIC | GCO | 37.81% | 45.61% |
| NP_459861 | - | putative inner membrane protein | + | NP_418615 | ulaB | L-ascorbate-specific enzyme IIB component of PTS | GCO | 25.51% | 38.62% |
| NP_459862 | - | putative sulfatase | + | NP_418134 | yidJ | predicted sulfatase/phosphatase | nGCO | 24.40% | 51.88% |
| NP_459863 | artJ | arginine transport system component | - | NP_415381 | artJ | arginine transporter subunit | GCO | 84.36% | 48.63% |
| NP_459864 | artM | arginine transport system component | - | NP_415382 | artM | arginine transporter subunit | GCO | 85.58% | 52.01% |
| NP_459865 | artQ | arginine transport system component | - | NP_415383 | artQ | arginine transporter subunit | GCO | 89.87% | 54.95% |
| NP_459866 | artI | arginine transport system | - | NP_415384 | artI | arginine transporter subunit | GCO | 95.06% | 51.77% |
| NP_459867 | artP | arginine transport system | - | NP_415385 | artP | arginine transporter subunit | GCO | 88.84% | 53.63% |
| NP_459868 | ybjP | putative lipoprotein | - | NP_415386 | ybjP | predicted lipoprotein | GCO | 77.19% | 56.78% |
| NP_459869 | - | putative integrase | - | NP_416096 | intQ | Qin prophage; predicted defective integrase | nGCO | 55.78% | 47.36% |
| NP_459870 | - | putative excisionase | - |  |  |  | NO HOMOLOG |  | 46.91% |
| NP_951048 | - | hypothetical protein | - |  |  |  | NO HOMOLOG |  | 51.70% |
| NP_459871 | - | hypothetical protein | - |  |  |  | NO HOMOLOG |  | 48.54% |
| NP_459872 | - | hypothetical protein | - |  |  |  | NO HOMOLOG |  | 48.60% |
| NP_459873 | - | hypothetical protein | - |  |  |  | NO HOMOLOG |  | 41.48% |
| NP_459874 | - | putative transcriptional regulator | - |  |  |  | NO HOMOLOG |  | 42.52% |
| NP_459875 | - | hypothetical protein | - |  |  |  | NO HOMOLOG |  | 48.23% |
| NP_459876 | - | hypothetical protein | - |  |  |  | NO HOMOLOG |  | 47.15% |
| NP_459877 | - | putative helicase | - | NP_416689 | yejH | predicted ATP-dependet helicase | nGCO | 28.03% | 52.90% |
| NP_459878 | - | putative phage DNA primase | - |  |  |  | NO HOMOLOG |  | 52.01% |
| NP_459879 | - | hypothetical protein | - |  |  |  | NO HOMOLOG |  | 50.05% |
| NP_459880 | - | putative chaparone | - | NP_416077 | ydfT | Qin prophage; predicted antitermination protein Q | nGCO | 33.33% | 54.65% |
| NP_459881 | - | hypothetical protein | - |  |  |  | NO HOMOLOG |  | 36.89% |
| NP_459882 | - | hypothetical protein | - | NP_415695 | ycgJ | hypothetical protein | nGCO | 51.96% | 41.35% |
| NP_459883 | - | hypothetical protein | - |  |  |  | NO HOMOLOG |  | 49.56% |
| NP_459884 | - | putative chitinase | - |  |  |  | NO HOMOLOG |  | 53.49% |
| NP_459885 | - | hypothetical protein | - | NP_415088 | rzpD | DLP12 prophage; predicted murein endopeptidase | nGCO | 66.43% | 53.90% |
| NP_459886 | - | hypothetical protein | - |  |  |  | NO HOMOLOG |  | 51.42% |
| NP_459887 | - | hypothetical protein | - |  |  |  | NO HOMOLOG |  | 56.70% |
| NP_459888 | - | hypothetical protein | - |  |  |  | NO HOMOLOG |  | 60.48% |
| NP_459889 | - | ATP-dependent protease | - |  |  |  | NO HOMOLOG |  | 57.65% |
| NP_459890 | - | hypothetical protein | - |  |  |  | NO HOMOLOG |  | 59.97% |
| NP_945156 | - | hypothetical protein | - |  |  |  | NO HOMOLOG |  | 60.91% |
| NP_945157 | - | hypothetical protein | - |  |  |  | NO HOMOLOG |  | 60.50% |
| NP_459891 | - | putative phage tail component | - |  |  |  | NO HOMOLOG |  | 58.63% |
| NP_459892 | - | hypothetical protein | - |  |  |  | NO HOMOLOG |  | 54.97% |
| NP_459893 | - | putative major tail protein | - |  |  |  | NO HOMOLOG |  | 56.85% |
| NP_945158 | - | hypothetical protein | - |  |  |  | NO HOMOLOG |  | 56.29% |
| NP_459894 | - | putative minor tail protein | - |  |  |  | NO HOMOLOG |  | 58.17% |
| NP_459895 | - | putative minor tail protein | - |  |  |  | NO HOMOLOG |  | 57.17% |
| NP_459896 | - | putative minor tail protein | - |  |  |  | NO HOMOLOG |  | 55.75% |
| NP_459897 | - | Ail/OmpX-like protein | - | NP_415335 | ompX | outer membrane protein X | nGCO | 34.91% | 45.66% |
| NP_459898 | - | putative minor tail protein | - |  |  |  | NO HOMOLOG |  | 58.04% |
| NP_459899 | - | putative phage tail assembly protein | - |  |  |  | NO HOMOLOG |  | 55.78% |
| NP_459900 | - | putative phage tail assembly protein | - |  |  |  | NO HOMOLOG |  | 58.99% |
| NP_459901 | - | putative CuZn superoxide dismutase | - | NP_416163 | sodC | superoxide dismutase, Cu, Zn | nGCO | 61.74% | 48.57% |
| NP_459902 | - | putative host-specificity protein | - |  |  |  | NO HOMOLOG |  | 57.21% |
| NP_459903 | - | putative minor tail protein | - |  |  |  | NO HOMOLOG |  | 58.23% |
| NP_459904 | - | putative phage tail assembly protein | - | NP_415891 | tfaR | Rac prophage; predicted tail fiber assembly protein | nGCO | 61.13% | 47.22% |
| NP_459905 | nanH | neuraminidase | - |  |  |  | NO HOMOLOG |  | 40.43% |
| NP_459906 | - | putative inner membrane protein | - |  |  |  | NO HOMOLOG |  | 35.48% |
| NP_459907 | orfB | hypothetical protein | - | NP_415387 | ybjQ | hypothetical protein | GCO | 94.39% | 53.70% |
| NP_459908 | ybjR | putative aminidase | - | NP_415388 | ybjR | predicted amidase and lipoprotein | GCO | 82.75% | 57.03% |
| NP_459909 | - | putative nucleoside-diphosphate-sugar epimerase | - | NP_415389 | ybjS | predicted NAD(P)H-binding oxidoreductase with NAD(P)-binding Rossmann-fold domain | GCO | 93.17% | 54.83% |
| NP_459910 | ybjT | putative nucleoside-diphosphate-sugar epimerase | - | NP_415390 | ybjT | conserved protein with NAD(P)-binding Rossmann-fold domain | GCO | 80.67% | 58.01% |
| NP_459911 | ltaA | L-allo-threonine aldolase | - | NP_415391 | ltaE | L-allo-threonine aldolase, PLP-dependent | GCO | 85.58% | 59.78% |
| NP_459912 | poxB | pyruvate dehydrogenase | - | NP_415392 | poxB | pyruvate dehydrogenase | GCO | 90.20% | 57.06% |
| NP_459913 | hcr | NADH oxidoreductase | - | NP_415393 | hcr | HCP oxidoreductase, NADH-dependent | GCO | 86.33% | 56.37% |
| NP_459914 | hcp | hydroxylamine reductase | - | NP_415394 | hcp | hydroxylamine reductase | GCO | 94% | 56.86% |
| NP_459915 | ybjE | putative inner membrane protein | - | NP_415395 | ybjE | predicted transporter | GCO | 83.87% | 51% |
| NP_459916 | ybjD | hypothetical protein | - | NP_415397 | ybjD | conserved protein with nucleoside triphosphate hydrolase domain | GCO | 86.77% | 55.45% |
| NP_459917 | ybjX | VirK-like protein | - | NP_415398 | ybjX | hypothetical protein | GCO | 57.14% | 46.85% |
| NP_459918 | ybjY | hypothetical protein | - | NP_415399 | macA | macrolide transporter subunit, membrane fusion protein (MFP) component | GCO | 83.10% | 53.35% |
| NP_459919 | ybjZ | putative transport protein | - | NP_415400 | macB | fused macrolide transporter subunits of ABC superfamily: ATP-binding component/membrane component | GCO | 77.00% | 54.44% |
| NP_459920 | cspD | stress response protein | - | NP_415401 | cspD | cold shock protein homolog | GCO | 94.36% | 51.80% |
| NP_459921 | clpS | ATP-dependent Clp protease adaptor protein ClpS | - | NP_415402 | clpS | ATP-dependent Clp protease adaptor protein ClpS | GCO | 97.16% | 46.41% |
| NP_459922 | clpA | ATP-binding subunit of serine protease | - | NP_415403 | clpA | ATPase and specificity subunit of ClpA-ClpP ATP-dependent serine protease, chaperone activity | GCO | 97.22% | 54.19% |
| NP_459923 | tnpA_1 | transposase | - |  |  |  | NO HOMOLOG |  | 45.96% |
| NP_459924 | - | putative integrase | - | NP_418015 | insK | IS150 conserved protein InsB | nGCO | 29.88% | 50.43% |
| NP_459925 | - | putative cytoplasmic protein | - |  |  |  | NO HOMOLOG |  | 50.24% |
| NP_459926 | - | SlsA | - | NP_415417 | ycaC | predicted hydrolase | nGCO | 46.66% | 53.83% |
| NP_459927 | - | putative cytoplasmic protein | - | NP_417896 | yhhW | hypothetical protein | nGCO | 28.64% | 56.21% |
| NP_459928 | - | putative transcriptional regulator | - | NP_414744 | yafC | predicted DNA-binding transcriptional regulator | nGCO | 28.47% | 57.89% |
| NP_459929 | infA | translation initiation factor IF-1 | - | NP_415404 | infA | translation initiation factor IF-1 | nGCO | 100% | 48.85% |
| NP_459930 | - | putative inner membrane protein | - |  |  |  | NO HOMOLOG |  | 43.48% |
| NP_459931 | aat | leucyl/phenylalanyl-tRNA--protein transferase | - | NP_415405 | aat | leucyl/phenylalanyl-tRNA--protein transferase | GCO | 82.47% | 56.73% |
| NP_459932 | cydC | cytochrome-related transporter | - | NP_415406 | cydC | fused cysteine transporter subunits of ABC superfamily: membrane component/ATP-binding component | GCO | 77.13% | 57.02% |
| NP_459933 | cydD | cytochrome-related transporter | - | NP_415407 | cydD | fused cysteine transporter subunits of ABC superfamily: membrane component/ATP-binding component | GCO | 86.90% | 56.59% |
| NP_459934 | trxB | thioredoxin reductase | - | NP_415408 | trxB | thioredoxin reductase, FAD/NAD(P)-binding | GCO | 92.81% | 55.00% |
| NP_459935 | lrp | leucine-responsive regulatory protein | - | NP_415409 | lrp | DNA-binding transcriptional dual regulator, leucine-binding | GCO | 99.39% | 49.09% |
| NP_459936 | ftsK | cell division protein | - | NP_415410 | ftsK | DNA-binding membrane protein required for chromosome resolution and partitioning | GCO | 55.28% | 58.55% |
| NP_459937 | lolA | outer-membrane lipoprotein carrier protein precursor | - | NP_415411 | lolA | outer-membrane lipoprotein carrier protein precursor | GCO | 85.29% | 50.40% |
| NP_459938 | ycaJ | hypothetical protein | - | NP_415412 | ycaJ | recombination protein | GCO | 97.31% | 57.06% |
| NP_459939 | serS | seryl-tRNA synthetase | - | NP_415413 | serS | seryl-tRNA synthetase | GCO | 97.67% | 54.06% |
| NP_459940 | dmsA | anaerobic dimethyl sulfoxide reductase subunit A | - | NP_415414 | dmsA | dimethyl sulfoxide reductase, anaerobic, subunit A | GCO | 92.25% | 53.53% |
| NP_459941 | dmsB | anaerobic dimethyl sulfoxide reductase subunit B | - | NP_415415 | dmsB | dimethyl sulfoxide reductase, anaerobic, subunit B | GCO | 96.58% | 57.11% |
| NP_459942 | dmsC | anaerobic dimethyl sulfoxide reductase subunit C | - | NP_415416 | dmsC | dimethyl sulfoxide reductase, anaerobic, subunit C | GCO | 81.88% | 58.21% |
| NP_459943 | ycaD | putative MFS family transporter protein | - | NP_415418 | ycaD | putative MFS family transporter protein | GCO | 79.84% | 51.78% |
| NP_459944 | ycaM | putative amino-acid transporter | - | NP_415419 | ycaM | predicted transporter | GCO | 93.17% | 47.25% |
| NP_459945 | pflA | pyruvate formate lyase-activating enzyme 1 | - | NP_415422 | pflA | pyruvate formate lyase activating enzyme 1 | nGCO | 92.21% | 48.12% |
| NP_459946 | - | putative cytoplasmic protein | - |  |  |  | NO HOMOLOG |  | 44.95% |
| NP_459947 | - | SopD-like protein | + |  |  |  | NO HOMOLOG |  | 33.33% |
| NP_459948 | pflB | pyruvate formate lyase I | - | NP_415423 | pflB | pyruvate formate lyase I | GCO | 93.02% | 51.64% |
| NP_459949 | focA | putative formate transporter | - | NP_415424 | focA | formate transporter | GCO | 91.22% | 48.36% |
| NP_459950 | ycaO | putative cytoplasmic protein | - | NP_415425 | ycaO | hypothetical protein | GCO | 90.61% | 52.75% |
| NP_459951 | ycaP | putative inner membrane protein | - | NP_415426 | ycaP | conserved inner membrane protein | GCO | 86.46% | 51.51% |
| NP_459952 | serC | phosphoserine aminotransferase | - | NP_415427 | serC | phosphoserine aminotransferase | GCO | 89.50% | 53.62% |
| NP_459953 | aroA | 3-phosphoshikimate 1-carboxyvinyltransferase | - | NP_415428 | aroA | 3-phosphoshikimate 1-carboxyvinyltransferase | GCO | 86.65% | 53.89% |
| NP_459954 | ycaL | putative Zn-dependent protease | - | NP_415429 | ycaL | predicted peptidase with chaperone function | GCO | 69.68% | 51.04% |
| NP_459955 | cmk | cytidylate kinase | - | NP_415430 | cmk | cytidylate kinase | GCO | 86.78% | 55.70% |
| NP_459956 | rpsA | 30S ribosomal protein S1 | - | NP_415431 | rpsA | 30S ribosomal protein S1 | GCO | 87.25% | 51.01% |
| NP_459957 | himD | integration host factor beta subunit | - | NP_415432 | ihfB | integration host factor subunit beta | GCO | 98.93% | 50.17% |
| NP_459958 | ycaI | putative recombination protein | - | NP_415433 | ycaI | conserved inner membrane protein | GCO | 64.85% | 55.32% |
| NP_459959 | msbA | transport protein | - | NP_415434 | msbA | fused lipid transporter subunits of ABC superfamily: membrane component/ATP-binding component | GCO | 91.75% | 51.68% |
| NP_459960 | lpxK | tetraacyldisaccharide 4'-kinase | - | NP_415435 | lpxK | tetraacyldisaccharide 4'-kinase | GCO | 65.43% | 59.91% |
| NP_459961 | ycaQ | putative cytoplasmic protein | - | NP_415436 | ycaQ | hypothetical protein | GCO | 77.45% | 55.39% |
| NP_459962 | ycaR | putative inner membrane protein | - | NP_415437 | ycaR | hypothetical protein | GCO | 93.33% | 46.44% |
| NP_459963 | kdsB | 3-deoxy-manno-octulosonate cytidylyltransferase | - | NP_415438 | kdsB | 3-deoxy-manno-octulosonate cytidylyltransferase | GCO | 89.11% | 57.42% |
| NP_459964 | - | hypothetical protein | - | NP_415439 | ycbJ | hypothetical protein | GCO | 90.90% | 55.36% |
| NP_459965 | ycbC | putative KicA-like protein | - | NP_415440 | ycbC | conserved inner membrane protein | GCO | 77.73% | 56.49% |
| NP_459966 | smtA | S-adenosylmethionine-dependent methyltransferase | - | NP_415441 | smtA | predicted S-adenosyl-L-methionine-dependent methyltransferase | GCO | 88.58% | 55.59% |
| NP_459967 | mukF | condesin subunit F | - | NP_415442 | mukF | condesin subunit F | GCO | 95.90% | 53.81% |
| NP_459968 | mukE | condesin subunit E | - | NP_415443 | mukE | condesin subunit E | GCO | 99.55% | 53.47% |
| NP_459969 | mukB | condesin subunit B | - | NP_415444 | mukB | cell division protein MukB | GCO | 87.36% | 56.70% |
| NP_459970 | ycbB | putative periplasmic protein | - | NP_415445 | ycbB | predicted carboxypeptidase | GCO | 79.22% | 56.94% |
| NP_459971 | ycbK | putative outer membrane protein | - | NP_415446 | ycbK | hypothetical protein | GCO | 83.51% | 51.91% |
| NP_459972 | ycbL | putative metallo-beta-lactamase | - | NP_415447 | ycbL | predicted metal-binding enzyme | GCO | 91.62% | 55.09% |
| NP_459973 | aspC | aspartate aminotransferase | - | NP_415448 | aspC | aspartate aminotransferase, PLP-dependent | GCO | 95.95% | 55.41% |
| NP_459974 | ompF | outer membrane protein F precursor | - | NP_415449 | ompF | outer membrane porin 1a (Ia;b;F) | GCO | 57.26% | 51.09% |
| NP_459975 | asnS | asparaginyl-tRNA synthetase | - | NP_415450 | asnS | asparaginyl-tRNA synthetase | GCO | 94.42% | 55.10% |
| NP_459976 | - | putative leucine response regulator | - | NP_415409 | lrp | DNA-binding transcriptional dual regulator, leucine-binding | nGCO | 31.12% | 48.91% |
| NP_459977 | - | diaminopropionate ammonia-lyase | + | NP_417347 | ygeX | diaminopropionate ammonia-lyase | nGCO | 50.51% | 45.59% |
| NP_459978 | - | putative transcriptional regulator | + | NP_414548 | yaaJ | predicted transporter | nGCO | 26.94% | 43.07% |
| NP_459979 | pncB | nicotinate phosphoribosyltransferase | - | NP_415451 | pncB | nicotinate phosphoribosyltransferase | nGCO | 91% | 54.44% |
| NP_459980 | - | integrase | - | NP_416096 | intQ | Qin prophage; predicted defective integrase | nGCO | 54.19% | 48.80% |
| NP_459981 | - | excisionase | - |  |  |  | NO HOMOLOG |  | 49.79% |
| NP_459982 | - | hypothetical protein | - |  |  |  | NO HOMOLOG |  | 52.08% |
| NP_459983 | - | hypothetical protein | - |  |  |  | NO HOMOLOG |  | 51.89% |
| NP_459984 | - | exodeoxyribonuclease | - |  |  |  | NO HOMOLOG |  | 53.08% |
| NP_459985 | - | hypothetical protein | + |  |  |  | NO HOMOLOG |  | 38% |
| NP_945159 | - | hypothetical protein | - | YP_588451 | ydaE | Rac prophage; conserved protein | nGCO | 40% | 44.65% |
| NP_459986 | - | hypothetical protein | - |  |  |  | NO HOMOLOG |  | 48.65% |
| NP_459987 | - | probable regulatory protein | - | NP_416088 | dicA | Qin prophage; predicted regulator for DicB | nGCO | 32.98% | 46.95% |
| NP_459988 | - | probable regulatory protein | - |  |  |  | NO HOMOLOG |  | 52.80% |
| NP_459989 | - | probable regulatory protein | - |  |  |  | NO HOMOLOG |  | 46.74% |
| NP_459990 | - | DNA replication protein DnaC | - | NP_415878 | ydaV | Rac prophage; predicted DNA replication protein | nGCO | 49.39% | 52.53% |
| NP_459991 | - | hypothetical protein | - |  |  |  | NO HOMOLOG |  | 54.31% |
| NP_459992 | - | hypothetical protein | - |  |  |  | NO HOMOLOG |  | 54.48% |
| NP_459993 | - | hypothetical protein | - |  |  |  | NO HOMOLOG |  | 35.39% |
| NP_459994 | - | hypothetical protein | - | NP_415579 | dinI | DNA damage-inducible protein I | nGCO | 47.43% | 46.15% |
| NP_459995 | - | hypothetical protein | - |  |  |  | NO HOMOLOG |  | 52.23% |
| NP_459996 | - | hypothetical protein | - |  |  |  | NO HOMOLOG |  | 58.66% |
| NP_951049 | - | hypothetical protein | - |  |  |  | NO HOMOLOG |  | 46.25% |
| NP_459997 | - | putative molecular chaperone | - | NP_416077 | ydfT | Qin prophage; predicted antitermination protein Q | nGCO | 34.31% | 46.36% |
| NP_459998 | - | hypothetical protein | - |  |  |  | NO HOMOLOG |  | 46.54% |
| NP_459999 | - | hypothetical protein | - |  |  |  | NO HOMOLOG |  | 42.85% |
| NP_460000 | - | hypothetical protein | - |  |  |  | NO HOMOLOG |  | 53.77% |
| NP_460001 | - | hypothetical protein | - |  |  |  | NO HOMOLOG |  | 43.46% |
| NP_460002 | - | hypothetical protein | - |  |  |  | NO HOMOLOG |  | 52.20% |
| NP_460003 | - | lysozyme | - | NP_415087 | ybcS | DLP12 prophage; predicted lysozyme | GCO | 36.75% | 53.20% |
| NP_460004 | - | hypothetical protein | - | NP_415088 | rzpD | DLP12 prophage; predicted murein endopeptidase | GCO | 65.30% | 54.37% |
| NP_460005 | - | hypothetical protein | - |  |  |  | NO HOMOLOG |  | 51.49% |
| NP_460006 | - | hypothetical protein | - |  |  |  | NO HOMOLOG |  | 54.93% |
| NP_460007 | - | hypothetical protein | - |  |  |  | NO HOMOLOG |  | 56.84% |
| NP_460008 | - | Clp protease-like protein | - |  |  |  | NO HOMOLOG |  | 52.49% |
| NP_460009 | - | putative RecA/RadA recombinase | - |  |  |  | NO HOMOLOG |  | 52.77% |
| NP_460010 | - | ATP-binding sugar transporter-like protein | - |  |  |  | NO HOMOLOG |  | 52.33% |
| NP_460011 | - | probable minor tail protein | - |  |  |  | NO HOMOLOG |  | 51.32% |
| NP_460012 | - | probable minor tail protein | - |  |  |  | NO HOMOLOG |  | 55.47% |
| NP_460013 | - | probable major tail protein | - |  |  |  | NO HOMOLOG |  | 50.80% |
| NP_460014 | - | probable minor tail protein | - |  |  |  | NO HOMOLOG |  | 50.12% |
| NP_460015 | - | probable minor tail protein | - |  |  |  | NO HOMOLOG |  | 53.63% |
| NP_460016 | - | probable minor tail protein | - |  |  |  | NO HOMOLOG |  | 56.29% |
| NP_460017 | - | probable minor tail protein | - |  |  |  | NO HOMOLOG |  | 51.35% |
| NP_460018 | - | attachment/invasion protein | - | NP_415335 | ompX | outer membrane protein X | nGCO | 33.33% | 44.37% |
| NP_460019 | sodC | superoxide dismutase precursor | - | NP_416163 | sodC | superoxide dismutase, Cu, Zn | nGCO | 55.88% | 48.68% |
| NP_460020 | - | probable minor tail protein | - |  |  |  | NO HOMOLOG |  | 53.73% |
| NP_460021 | - | probable tail assembly protein | - |  |  |  | NO HOMOLOG |  | 54.60% |
| NP_460022 | - | probable tail assembly protein | - |  |  |  | NO HOMOLOG |  | 56.87% |
| NP_460023 | - | host specificity protein J | - |  |  |  | NO HOMOLOG |  | 56.61% |
| NP_945160 | - | hypothetical protein | - |  |  |  | NO HOMOLOG |  | 53.42% |
| NP_460024 | - | probable tail fiber protein | - |  |  |  | NO HOMOLOG |  | 57.60% |
| NP_460025 | - | tail fiber assembly like-protein | - | NP_415891 | tfaR | Rac prophage; predicted tail fiber assembly protein | nGCO | 62.69% | 48.62% |
| NP_460026 | sseI | secreted effector protein | - |  |  |  | NO HOMOLOG |  | 41.07% |
| NP_460027 | - | hypothetical protein | - | YP_025310 | yedK | hypothetical protein | nGCO | 85.39% | 52.47% |
| NP_460028 | - | hypothetical protein | + |  |  |  | NO HOMOLOG |  | 42.33% |
| NP_460029 | - | hypothetical protein | + |  |  |  | NO HOMOLOG |  | 34.49% |
| NP_460030 | - | MsgA-like protein | - |  |  |  | NO HOMOLOG |  | 47.91% |
| NP_460031 | pepN | aminopeptidase N | - | NP_415452 | pepN | aminopeptidase N | nGCO | 91.49% | 53.73% |
| NP_460032 | pyrD | dihydroorotate dehydrogenase | - | NP_415465 | pyrD | dihydroorotate dehydrogenase | GCO | 94.64% | 48.07% |
| NP_460033 | ycbW | putative cytoplasmic protein | - | NP_415466 | ycbW | hypothetical protein | GCO | 90% | 51.74% |
| NP_460034 | - | putative iron-sulfur protein | - | NP_415467 | ycbX | predicted 2Fe-2S cluster-containing protein | GCO | 80.38% | 55.40% |
| NP_460035 | ycbY | putative N6-adenine-specific DNA methylase | - | NP_415468 | ycbY | predicted methyltransferase | GCO | 87.17% | 55.04% |
| NP_460036 | uup | putative ABC transporter ATPase component | - | NP_415469 | uup | fused predicted transporter subunits of ABC superfamily: ATP-binding components | GCO | 89.90% | 52.83% |
| NP_460037 | pqiA | paraquat-inducible protein A | - | NP_415470 | pqiA | paraquat-inducible membrane protein A | GCO | 84.41% | 52.23% |
| NP_460038 | pqiB | paraquat-inducible protein B | - | NP_415471 | pqiB | paraquat-inducible protein B | GCO | 86.99% | 51.31% |
| NP_460039 | ymbA | putative outer membrane protein | - | NP_415472 | ymbA | hypothetical protein | GCO | 78.57% | 52.30% |
| NP_460040 | rmf | ribosome modulation factor | - | NP_415473 | rmf | ribosome modulation factor | GCO | 92.72% | 53.57% |
| NP_460041 | fabA | 3-hydroxydecanoyl-ACP dehydratase | - | NP_415474 | fabA | 3-hydroxydecanoyl-ACP dehydratase | GCO | 98.83% | 53.56% |
| NP_460042 | lonH | putative protease | - | NP_415475 | ycbZ | predicted peptidase | GCO | 84.12% | 53.43% |
| NP_460043 | ycbG | hypothetical protein | - | NP_415476 | ycbG | hypothetical protein | GCO | 81.33% | 46.79% |
| NP_460044 | ompA | putative hydrogenase membrane component precurosr | - | NP_415477 | ompA | outer membrane protein A (3a;II*;G;d) | GCO | 88.41% | 54.13% |
| NP_460045 | sulA | cell division inhibitor | - | NP_415478 | sulA | SOS cell division inhibitor | GCO | 66.86% | 50.58% |
| NP_460046 | yccR | putative DNA transformation protein | - | NP_415479 | yccR | hypothetical protein | GCO | 75.87% | 50.16% |
| NP_460047 | yccS | putative transporter | - | NP_415480 | yccS | predicted inner membrane protein | GCO | 82.74% | 54.31% |
| NP_460048 | yccF | putative inner membrane protein | - | NP_415481 | yccF | conserved inner membrane protein | GCO | 74.62% | 51.90% |
| NP_460049 | helD | DNA helicase IV | - | NP_415482 | helD | DNA helicase IV | GCO | 83.91% | 55.57% |
| NP_460050 | mgsA | methylglyoxal synthase | - | NP_415483 | mgsA | methylglyoxal synthase | GCO | 94.73% | 53.81% |
| NP_460051 | yccT | hypothetical protein | - | NP_415484 | yccT | hypothetical protein | GCO | 84.54% | 52.94% |
| NP_460052 | - | putative cytoplasmic protein | - | NP_415485 | yccU | predicted CoA-binding protein with NAD(P)-binding Rossmann-fold domain | GCO | 91.97% | 55.63% |
| NP_460053 | yccV | putative inner membrane protein | - | NP_415486 | yccV | DNA-binding protein, hemimethylated | GCO | 92.38% | 55.66% |
| NP_460054 | yccW | putative SAM-dependent methyltransferase | - | NP_415487 | yccW | predicted methyltransferase | GCO | 90.73% | 54.20% |
| NP_460055 | - | putative outer membrane protein | - | NP_415077 | ybcL | DLP12 prophage; predicted kinase inhibitor | GCO | 64.05% | 54.64% |
| NP_460056 | - | bacterial regulatory protein | - | NP_415078 | ybcM | DLP12 prophage; predicted DNA-binding transcriptional regulator | GCO | 31.62% | 53.84% |
| NP_460057 | yccX | putative phosphohydrolase | - | NP_415488 | yccX | predicted acylphosphatase | GCO | 78.26% | 56.02% |
| NP_460058 | yccK | putative sulfite reductase gamma subunit | - | NP_415489 | yccK | predicted sulfite reductase subunit | GCO | 90.82% | 50.30% |
| NP_460059 | yccA | putative transport protein | - | NP_415490 | yccA | inner membrane protein | GCO | 88.61% | 52.57% |
| NP_460060 | pipA | pathogenicity island-encoded protein A | + |  |  |  | NO HOMOLOG |  | 44.05% |
| NP_460061 | pipB | secreted effector protein | + |  |  |  | NO HOMOLOG |  | 39.38% |
| NP_460062 | - | putative inner membrane protein | - |  |  |  | NO HOMOLOG |  | 35.51% |
| NP_460063 | pipC | pathogenicity island-encoded protein C | - |  |  |  | NO HOMOLOG |  | 43.85% |
| NP_460064 | sopB | secreted effector protein | - |  |  |  | NO HOMOLOG |  | 47.03% |
| NP_460065 | orfX | putative cytoplasmic protein | - | YP_588454 | ynfO | Qin prophage; predicted protein | nGCO | 54.90% | 43.85% |
| NP_460066 | - | putative cytoplasmic protein | - |  |  |  | NO HOMOLOG |  | 44.14% |
| NP_460067 | pipD | pathogenicity island-encoded protein D | - |  |  |  | NO HOMOLOG |  | 48.63% |
| NP_460068 | copS | copper resistance protein | - | NP_416477 | yedV | predicted sensory kinase in two-component regulatory system with YedW | GCO | 54.44% | 51.50% |
| NP_460069 | copR | copper resistance protein | - | NP_416478 | yedW | predicted DNA-binding response regulator in two-component system with YedV | GCO | 75.45% | 50.06% |
| NP_460070 | - | hypothetical protein | - | NP_416479 | yedX | hypothetical protein | GCO | 66.17% | 48.17% |
| NP_460071 | hpaC | 4-hydroxyphenylacetate catabolism | - | NP_415527 | ycdH | predicted oxidoreductase, flavin:NADH component | nGCO | 46.20% | 54.58% |
| NP_460072 | hpaB | 4-hydroxyphenylacetate catabolism | - |  |  |  | NO HOMOLOG |  | 52.59% |
| NP_460073 | hpaR | 4-hydroxyphenylacetate catabolism | - |  |  |  | NO HOMOLOG |  | 52.15% |
| NP_460074 | hpaG | 4-hydroxyphenylacetate catabolism | - |  |  |  | NO HOMOLOG |  | 56.35% |
| NP_460075 | hpaE | 4-hydroxyphenylacetate catabolism | - | NP_414846 | betB | betaine aldehyde dehydrogenase, NAD-dependent | nGCO | 39.30% | 59.71% |
| NP_460076 | hpaD | 4-hydroxyphenylacetate catabolism | - |  |  |  | NO HOMOLOG |  | 53.87% |
| NP_460077 | hpaF | 4-hydroxyphenylacetate catabolism | - |  |  |  | NO HOMOLOG |  | 53.80% |
| NP_460078 | hpaH | 4-hydroxyphenylacetate catabolism | - | NP_414884 | mhpD | 2-keto-4-pentenoate hydratase | nGCO | 32.83% | 59.07% |
| NP_460079 | hpaI | 4-hydroxyphenylacetate catabolism | - | NP_416748 | yfaU | predicted 2,4-dihydroxyhept-2-ene-1,7-dioic acid aldolase | GCO | 58.89% | 60.73% |
| NP_460080 | hpaX | 4-hydroxyphenylacetate catabolism | - | NP_416749 | yfaV | predicted transporter | GCO | 30.42% | 53.08% |
| NP_460081 | hpaA | 4-hydroxyphenylacetate catabolism | - |  |  |  | NO HOMOLOG |  | 52.06% |
| NP_460082 | - | putative periplasmic protein | - | NP_416771 | elaC | ribonuclease Z | nGCO | 23.79% | 52.60% |
| NP_460083 | - | putative cytoplasmic protein | + | NP_415678 | elbA | hypothetical protein | nGCO | 40.36% | 38.01% |
| NP_460084 | yccD | putative cytoplasmic protein | - | NP_415519 | cbpM | modulator of CbpA co-chaperone | GCO | 53.46% | 55.22% |
| NP_460085 | cbpA | DNA-binding protein | - | NP_415520 | cbpA | curved DNA-binding protein, DnaJ homologue that functions as a co-chaperone of DnaK | GCO | 85.62% | 53.42% |
| NP_460086 | scsA | suppression of copper sensitivity protein A | - |  |  |  | NO HOMOLOG |  | 59.50% |
| NP_460087 | scsB | suppression of copper sensitivity protein | - | NP_418559 | dipZ | thiol:disulfide interchange protein precursor | nGCO | 24.52% | 58.08% |
| NP_460088 | scsC | suppression of copper sensitivity protein | - |  |  |  | NO HOMOLOG |  | 52.24% |
| NP_460089 | scsD | suppression of copper sensitivity protein | - |  |  |  | NO HOMOLOG |  | 61.93% |
| NP_460090 | agp | glucose-1-phosphatase | - | NP_415522 | agp | glucose-1-phosphatase/inositol phosphatase | GCO | 84.47% | 54.99% |
| NP_460091 | yccJ | putative cytoplasmic protein | - | NP_415523 | yccJ | hypothetical protein | GCO | 92% | 47.80% |
| NP_460092 | wraB | TrpR binding protein WrbA | - | NP_415524 | wrbA | TrpR binding protein WrbA | GCO | 86.36% | 56.78% |
| NP_460093 | ymdF | putative cytoplasmic protein | - | NP_415525 | ymdF | hypothetical protein | GCO | 92.72% | 52.38% |
| NP_460094 | ycdC | putative transcriptional repressor | - | NP_415533 | ycdC | predicted DNA-binding transcriptional regulator | nGCO | 83.01% | 54.46% |
| NP_460095 | - | putative periplasmic protein | - |  |  |  | NO HOMOLOG |  | 58.33% |
| NP_460097 | putP | major sodium/proline symporter | - | NP_415535 | putP | proline:sodium symporter | nGCO | 88.80% | 54.07% |
| NP_460099 | - | putative transcriptional regulator | - | NP_416922 | yfeT | predicted DNA-binding transcriptional regulator | nGCO | 29.60% | 49.20% |
| NP_460100 | - | putative sodium/glucose cotransporter | + | NP_418135 | yidK | predicted transporter | nGCO | 19.48% | 45.35% |
| NP_460101 | - | putative inner membrane protein | - | NP_417690 | nanE | predicted N-acetylmannosamine-6-P epimerase | nGCO | 65.48% | 51.54% |
| NP_460102 | - | putative inner membrane protein | + | NP_418730 | yjhT | hypothetical protein | GCO | 40.37% | 40.56% |
| NP_460103 | - | putative outer membrane protein | + | NP_418731 | yjhA | N-acetylnuraminic acid outer membrane channel protein | GCO | 26.14% | 36.07% |
| NP_460104 | - | putative sialic acid transporter | + | NP_418699 | yjhB | KpLE2 phage-like element; predicted transporter | GCO | 56.40% | 45.27% |
| NP_460105 | - | putative dehydrogenase | + | NP_418700 | yjhC | KpLE2 phage-like element; predicted oxidoreductase | GCO | 59.56% | 46.52% |
| NP_460106 | ycdW | putative oxidoreductase | - | NP_415551 | ycdW | 2-ketoacid reductase | GCO | 77.88% | 55.05% |
| NP_460107 | ycdX | hypothetical protein | - | NP_415552 | ycdX | hypothetical protein | GCO | 79.18% | 51.62% |
| NP_460108 | ycdY | putative oxidoreductase component | - | NP_415553 | ycdY | hypothetical protein | GCO | 86.41% | 53.15% |
| NP_460109 | ycdZ | putative inner membrane protein | - | NP_415554 | ycdZ | predicted inner membrane protein | GCO | 86.50% | 52.54% |
| NP_460110 | csgG | putative curli operon transcriptional regulator | + | NP_415555 | csgG | outer membrane lipoprotein | GCO | 97.34% | 49.04% |
| NP_460111 | csgF | curli production assembly/transport component | + | NP_415556 | csgF | predicted transport protein | GCO | 78.26% | 44.60% |
| NP_460112 | csgE | curli production assembly/transport component | + | NP_415557 | csgE | predicted transport protein | GCO | 90.07% | 44.44% |
| NP_460113 | csgD | putative transcriptional regulator | + | NP_415558 | csgD | DNA-binding transcriptional activator in two-component regulatory system | GCO | 87.03% | 40.70% |
| NP_460114 | csgB | minor curlin subunit precursor | + | NP_415559 | csgB | curlin nucleator protein, minor subunit in curli complex | GCO | 82.11% | 43.42% |
| NP_460115 | csgA | major curlin subunit precursor | + | NP_415560 | csgA | cryptic curlin major subunit | GCO | 66.88% | 51.53% |
| NP_460116 | csgC | putative curli production protein precursor | - | NP_415561 | csgC | predicted curli production protein | GCO | 73.14% | 44.95% |
| NP_460117 | ymdA | putative periplasmic protein | - | NP_415562 | ymdA | hypothetical protein | GCO | 47.19% | 47.66% |
| NP_460118 | - | hypothetical protein | - | NP_415563 | ymdB | hypothetical protein | GCO | 76.27% | 55.18% |
| NP_460119 | ymdC | putative phospholipase | - | NP_415564 | ymdC | predicted hydrolase | GCO | 79.83% | 53.56% |
| NP_460120 | mdoC | glucans biosynthesis protein | - | NP_415565 | mdoC | glucans biosynthesis protein | GCO | 81.00% | 46.40% |
| NP_460121 | mdoG | periplasmic glucans biosynthesis protein | - | NP_415566 | mdoG | glucan biosynthesis protein, periplasmic | GCO | 93.73% | 51.04% |
| NP_460122 | mdoH | glucosyltransferase MdoH | - | NP_415567 | mdoH | glucosyltransferase MdoH | GCO | 90.71% | 56.25% |
| NP_460123 | yceK | putative outer membrane lipoprotein | - | NP_415568 | yceK | predicted lipoprotein | GCO | 88% | 52.63% |
| NP_460124 | msyB | acidic protein | - | NP_415569 | msyB | hypothetical protein | GCO | 92.74% | 52.26% |
| NP_460125 | yceE | putative transport protein | - | NP_415571 | mdtG | predicted drug efflux system | GCO | 74.93% | 54.89% |
| NP_460126 | htrB | lipid A biosynthesis lauroyl acyltransferase | - | NP_415572 | lpxL | lipid A biosynthesis lauroyl acyltransferase | GCO | 83.66% | 53.74% |
| NP_460127 | yceA | hypothetical protein | - | NP_415573 | yceA | hypothetical protein | GCO | 85.71% | 51.09% |
| NP_460128 | yceI | hypothetical protein | - | NP_415574 | yceI | hypothetical protein | GCO | 84.29% | 50.34% |
| NP_460129 | - | putative inner membrane protein | - | NP_415575 | yceJ | predicted cytochrome b561 | GCO | 70.05% | 52.53% |
| NP_460130 | yceO | putative inner membrane protein | - | NP_415576 | yceO | hypothetical protein | GCO | 64.86% | 38.59% |
| NP_460131 | solA | putative sarcosine oxidase | - | NP_415577 | solA | N-methyltryptophan oxidase, FAD-binding | GCO | 81.13% | 55.76% |
| NP_460132 | yceP | putative cytoplasmic protein | - | NP_415578 | yceP | hypothetical protein | GCO | 94.04% | 46.51% |
| NP_460133 | dinI | DNA damage-inducible protein I | - | NP_415579 | dinI | DNA damage-inducible protein I | GCO | 85.18% | 51.62% |
| NP_460134 | pyrC | dihydroorotase | - | NP_415580 | pyrC | dihydroorotase | GCO | 88.50% | 53.96% |
| NP_460135 | yceB | putative outer membrane lipoprotein | - | NP_415581 | yceB | predicted lipoprotein | GCO | 84.40% | 45.45% |
| NP_460136 | grxB | glutaredoxin 2 | - | NP_415582 | grxB | glutaredoxin 2 (Grx2) | GCO | 86.04% | 46.45% |
| NP_460137 | yceL | putative transport protein | - | NP_415583 | mdtH | predicted drug efflux system | GCO | 81.84% | 56.32% |
| NP_460138 | rimJ | acetylatase | - | NP_415584 | rimJ | ribosomal-protein-S5-alanine N-acetyltransferase | GCO | 95.36% | 51.11% |
| NP_460139 | yceH | putative cytoplasmic protein | - | NP_415585 | yceH | hypothetical protein | GCO | 80% | 56.01% |
| NP_460140 | mviM | putative virulence protein | - | NP_415586 | mviM | predicted oxidoreductase with NAD(P)-binding Rossmann-fold domain | GCO | 81.75% | 57.68% |
| NP_460141 | mviN | putative virulence protein | - | NP_415587 | mviN | predicted inner membrane protein | GCO | 84.90% | 54.22% |
| NP_460142 | flgN | putative FlgK/FlgL export chaperone | - | NP_415588 | flgN | export chaperone for FlgK and FlgL | GCO | 80% | 53.66% |
| NP_460143 | flgM | anti-FliA factor | - | NP_415589 | flgM | anti-sigma factor for FliA (sigma 28) | GCO | 73.95% | 51.70% |
| NP_460144 | flgA | flagellar basal body P-ring biosynthesis protein | - | NP_415590 | flgA | flagellar basal body P-ring biosynthesis protein A | GCO | 64.38% | 58.03% |
| NP_460145 | flgB | flagellar basal body rod protein | - | NP_415591 | flgB | flagellar basal-body rod protein B | GCO | 81.15% | 52.27% |
| NP_460146 | flgC | flagellar basal body rod protein | - | NP_415592 | flgC | flagellar basal-body rod protein C | GCO | 94.02% | 54.81% |
| NP_460147 | flgD | flagellar basal body rod modification protein | - | NP_415593 | flgD | flagellar basal body rod modification protein D | GCO | 73.70% | 54.50% |
| NP_460148 | flgE | flagellar hook protein | - | NP_415594 | flgE | flagellar hook protein E | GCO | 78.46% | 55.36% |
| NP_460149 | flgF | cell-proximal portion of basal-body rod | - | NP_415595 | flgF | flagellar component of cell-proximal portion of basal-body rod | GCO | 86.85% | 60.71% |
| NP_460150 | flgG | flagellar basal-body rod protein | - | NP_415596 | flgG | flagellar component of cell-distal portion of basal-body rod | GCO | 94.61% | 56.44% |
| NP_460151 | flgH | flagellar L-ring protein precursor | - | NP_415597 | flgH | flagellar L-ring protein precursor H | GCO | 86.20% | 54.36% |
| NP_460152 | flgI | flagellar P-ring protein precursor | - | NP_415598 | flgI | flagellar P-ring protein precursor I | GCO | 84.10% | 57.65% |
| NP_460153 | flgJ | flagellar biosynthesis protein | - | NP_415599 | flgJ | flagellar biosynthesis protein FlgJ | GCO | 80.06% | 56.25% |
| NP_460154 | flgK | flagellar hook-associated protein | - | NP_415600 | flgK | flagellar hook-associated protein K | GCO | 70.39% | 51.80% |
| NP_460155 | flgL | flagellar hook-associated protein | - | NP_415601 | flgL | flagellar hook-associated protein L | GCO | 76.97% | 53.66% |
| NP_460156 | rne | RNase E | - | NP_415602 | rne | fused ribonucleaseE: endoribonuclease/RNA-binding protein/RNA degradosome binding protein | GCO | 91.09% | 58.42% |
| NP_460157 | rluC | 23S rRNA pseudouridylate synthase | - | NP_415604 | rluC | 23S rRNA pseudouridylate synthase | nGCO | 92.16% | 55.31% |
| NP_460158 | - | putative inner membrane lipoprotein | - | NP_418010 | yiaF | hypothetical protein | nGCO | 28.12% | 46.23% |
| NP_460159 | maf | Maf-like protein | - | NP_415605 | yceF | Maf-like protein | GCO | 88.02% | 55.38% |
| NP_460160 | yceD | putative metal-binding protein | - | NP_415606 | yceD | hypothetical protein | GCO | 87.28% | 51.72% |
| NP_460161 | rpmF | 50S ribosomal protein L32 | - | NP_415607 | rpmF | 50S ribosomal protein L32 | GCO | 100% | 52.87% |
| NP_460162 | plsX | fatty acid/phospholipid synthesis protein | - | NP_415608 | plsX | fatty acid/phospholipid synthesis protein | GCO | 74.43% | 53.42% |
| NP_460163 | fabH | 3-oxoacyl-(acyl carrier protein) synthase | - | NP_415609 | fabH | 3-oxoacyl-(acyl carrier protein) synthase | GCO | 94.63% | 54.50% |
| NP_460164 | fabD | acyl carrier protein S-malonyltransferase | - | NP_415610 | fabD | acyl carrier protein S-malonyltransferase | GCO | 87.94% | 56.66% |
| NP_460165 | fabG | 3-ketoacyl-(acyl-carrier-protein) reductase | - | NP_415611 | fabG | 3-oxoacyl-[acyl-carrier-protein] reductase | GCO | 90.57% | 50.34% |
| NP_460166 | acpP | acyl carrier protein | - | NP_415612 | acpP | acyl carrier protein | GCO | 82.05% | 47.67% |
| NP_460167 | fabF | 3-oxoacyl-(acyl carrier protein) synthase | - | NP_415613 | fabF | 3-oxoacyl-(acyl carrier protein) synthase | GCO | 90.79% | 55.23% |
| NP_460168 | pabC | 4-amino-4-deoxychorismate lyase | - | NP_415614 | pabC | 4-amino-4-deoxychorismate lyase | GCO | 66.54% | 51.48% |
| NP_460169 | yceG | putative periplasmic solute-binding protein | - | NP_415615 | yceG | predicted aminodeoxychorismate lyase | GCO | 86.47% | 52.59% |
| NP_460170 | tmk | thymidylate kinase | - | NP_415616 | tmk | thymidylate kinase | GCO | 90.95% | 57.78% |
| NP_460171 | holB | DNA polymerase III subunit delta | - | NP_415617 | holB | DNA polymerase III subunit delta' | GCO | 72.15% | 59.30% |
| NP_460172 | ycfH | putative metal-dependent hydrolase | - | NP_415618 | ycfH | predicted metallodependent hydrolase | GCO | 87.16% | 52.75% |
| NP_460173 | ptsG | glucose-specific IIBC component | - | NP_415619 | ptsG | fused glucose-specific PTS enzymes: IIB component/IIC component | GCO | 93.06% | 54.74% |
| NP_460174 | fhuE | outer membrane receptor protein precursor | - | NP_415620 | fhuE | ferric-rhodotorulic acid outer membrane transporter | GCO | 80.05% | 52.68% |
| NP_460175 | ycfF | putative protein kinase C inhibitor | - | NP_415621 | hinT | purine nucleoside phosphoramidase | GCO | 98.31% | 50.83% |
| NP_460176 | ycfL | putative outer membrane lipoprotein | - | NP_415622 | ycfL | hypothetical protein | GCO | 80.80% | 57.60% |
| NP_460177 | ycfM | putative outer membrane lipoprotein | - | NP_415623 | ycfM | predicted outer membrane lipoprotein | GCO | 75.11% | 54.14% |
| NP_460178 | ycfN | putative cytoplasmic protein | - | NP_415624 | ycfN | thiamin kinase | GCO | 61.90% | 56.60% |
| NP_460179 | nagZ | beta-hexosaminidase | - | NP_415625 | nagZ | beta-hexosaminidase | GCO | 91.78% | 56.72% |
| NP_460180 | ycfP | hypothetical protein | - | NP_415626 | ycfP | hypothetical protein | GCO | 94.44% | 47.69% |
| NP_460181 | ndh | respiratory NADH dehydrogenase 2 | - | NP_415627 | ndh | respiratory NADH dehydrogenase 2/cupric reductase | GCO | 94.23% | 54.55% |
| NP_460182 | ycfJ | putative outer membrane lipoprotein | - | NP_415628 | ycfJ | hypothetical protein | GCO | 78.08% | 53.33% |
| NP_460183 | ycfQ | putative transcriptional repressor | - | NP_415629 | ycfQ | predicted DNA-binding transcriptional regulator | GCO | 80% | 53.45% |
| NP_460184 | ycfR | putative outer membrane protein | - | NP_415630 | ycfR | hypothetical protein | GCO | 75.29% | 54.26% |
| NP_460185 | ycfS | putative periplasmic protein | - | NP_415631 | ycfS | hypothetical protein | GCO | 82.55% | 56.31% |
| NP_460186 | mfd | transcription-repair coupling factor | - | NP_415632 | mfd | transcription-repair coupling factor | GCO | 93.98% | 57.06% |
| NP_460187 | ycfU | integral membrane protein | - | NP_415634 | lolC | outer membrane-specific lipoprotein transporter subunit | GCO | 83.70% | 55.41% |
| NP_460188 | ycfV | ATP-binding protein | - | NP_415635 | lolD | outer membrane-specific lipoprotein transporter subunit | GCO | 95.27% | 55.55% |
| NP_460189 | ycfW | integral membrane protein | - | NP_415636 | lolE | outer membrane-specific lipoprotein transporter subunit | GCO | 83.80% | 53.81% |
| NP_460190 | ycfX | putative regulatory protein | - | NP_415637 | nagK | N-acetyl-D-glucosamine kinase | GCO | 87.12% | 56.35% |
| NP_460191 | cobB | NAD-dependent deacetylase | - | NP_415638 | cobB | NAD-dependent deacetylase | GCO | 79.85% | 52.91% |
| NP_460192 | potD | spermidine/putrescine ABC transporter periplasmic substrate-binding component | + | NP_415641 | potD | spermidine/putrescine ABC transporter periplasmic substrate-binding protein | GCO | 89.08% | 51.48% |
| NP_460193 | potC | spermidine/putrescine ABC transporter membrane component | + | NP_415642 | potC | spermidine/putrescine ABC transporter membrane protein | GCO | 84.88% | 51.53% |
| NP_460194 | sifA | secreted effector protein | + |  |  |  | NO HOMOLOG |  | 41.14% |
| NP_460195 | potB | spermidine/putrescine ABC transporter membrane component | - | NP_415643 | potB | spermidine/putrescine ABC transporter membrane protein | GCO | 97.05% | 49.18% |
| NP_460196 | potA | putrescine/spermidine ABC transporter ATPase | - | NP_415644 | potA | putrescine/spermidine ABC transporter ATPase protein | GCO | 91.00% | 49.51% |
| NP_460197 | pepT | peptidase T | - | NP_415645 | pepT | peptidase T | GCO | 91.40% | 52.19% |
| NP_460198 | - | putative periplasmic protein | - |  |  |  | NO HOMOLOG |  | 47.64% |
| NP_460199 | ycfD | putative cytoplasmic protein | - | NP_415646 | ycfD | hypothetical protein | GCO | 90.08% | 55.79% |
| NP_460200 | phoQ | sensor kinase protein | - | NP_415647 | phoQ | sensory histidine kinase in two-compoent regulatory system with PhoP | GCO | 81.93% | 51.50% |
| NP_460201 | phoP | response regulator | - | NP_415648 | phoP | DNA-binding response regulator in two-component regulatory system with PhoQ | GCO | 89.68% | 51.70% |
| NP_460202 | purB | adenylosuccinate lyase | - | NP_415649 | purB | adenylosuccinate lyase | GCO | 94.95% | 51.49% |
| NP_460203 | ycfC | hypothetical protein | - | NP_415650 | hflD | hypothetical protein | GCO | 86.85% | 55.86% |
| NP_460204 | trmU | tRNA (5-methylaminomethyl-2-thiouridylate)-methyltransferase | - | NP_415651 | trmU | tRNA (5-methylaminomethyl-2-thiouridylate)-methyltransferase | GCO | 88.58% | 54.29% |
| NP_460205 | ymfB | putative MutT-like protein | - | NP_415652 | ymfB | bifunctional thiamin pyrimidine pyrophosphate hydrolase/ thiamin pyrophosphate hydrolase | GCO | 84.21% | 51.94% |
| NP_460206 | - | putative periplasmic protein | - |  |  |  | NO HOMOLOG |  | 50.90% |
| NP_460207 | ymfC | putative ribosomal large subunit pseudouridine synthase | - | NP_415653 | ymfC | 23S rRNA pseudouridine synthase | GCO | 83.25% | 52.85% |
| NP_460208 | icdA | isocitrate dehydrogenase | - | NP_415654 | icd | isocitrate dehydrogenase | GCO | 93.75% | 51.71% |
| NP_460209 | - | putative cytoplasmic protein | - |  |  |  | NO HOMOLOG |  | 38.57% |
| NP_460210 | envF | putative envelope lipoprotein | - |  |  |  | NO HOMOLOG |  | 39.67% |
| NP_460211 | msgA | macrophage survival protein | - | NP_415579 | dinI | DNA damage-inducible protein I | nGCO | 37.87% | 46.25% |
| NP_460212 | envE | putative envelope protein | + |  |  |  | NO HOMOLOG |  | 45.40% |
| NP_460213 | - | cold shock-like protein | - | NP_415509 | cspH | stress protein, member of the CspA-family | nGCO | 81.42% | 47.41% |
| NP_460214 | pagD | virulence protein PAGD precursor | - |  |  |  | NO HOMOLOG |  | 41.66% |
| NP_460215 | pagC | virulence membrane protein PAGC precursor | - | NP_415335 | ompX | outer membrane protein X | nGCO | 38.78% | 43.18% |
| NP_460216 | - | putative periplasmic protein | - |  |  |  | NO HOMOLOG |  | 42.02% |
| NP_460217 | - | putative cytoplasmic protein | - |  |  |  | NO HOMOLOG |  | 44.32% |
| NP_460218 | - | putative molecular chaperone | - | NP_418141 | ibpB | heat shock chaperone | nGCO | 31.45% | 45.51% |
| NP_460219 | - | putative cytoplasmic protein | - |  |  |  | NO HOMOLOG |  | 50.67% |
| NP_460220 | - | putative inner membrane protein | + | NP_416483 | yodB | predicted cytochrome | nGCO | 56.64% | 47.26% |
| NP_460221 | - | putative outer membrane lipoprotein | - |  |  |  | NO HOMOLOG |  | 41.58% |
| NP_460222 | - | putative ABC transporter periplasmic binding protein | - | NP_417933 | nikA | nickel transporter subunit | nGCO | 25.77% | 54.00% |
| NP_460223 | - | putative ABC transporter protein | - | NP_418000 | dppB | dipeptide transporter | nGCO | 27.24% | 52.92% |
| NP_460224 | - | putative ABC transporter protein | - | NP_417935 | nikC | nickel transporter subunit | GCO | 31.19% | 56.45% |
| NP_460225 | - | putative ABC-type transport system ATPase component | - | NP_417936 | nikD | nickel transporter subunit | GCO | 29.23% | 55.38% |
| NP_460226 | - | putative ABC-type transport system ATPase component | - | NP_415806 | sapF | predicted antimicrobial peptide transporter subunit | nGCO | 27.46% | 57.36% |
| NP_460227 | - | putative inner membrane protein | - | NP_415846 | ynaI | conserved inner membrane protein | nGCO | 23.94% | 48.32% |
| NP_460228 | - | putative cytoplasmic protein | - |  |  |  | NO HOMOLOG |  | 52.29% |
| NP_460229 | - | putative periplasmic protein | - | NP_416482 | yodA | conserved metal-binding protein | nGCO | 70% | 46.45% |
| NP_460230 | aadA | aminoglycoside adenyltransferase | - |  |  |  | NO HOMOLOG |  | 54.49% |
| NP_460231 | - | putative response regulator | - |  |  |  | NO HOMOLOG |  | 36.02% |
| NP_460232 | - | putative transcriptional regulator | - | NP_415680 | ycgE | predicted DNA-binding transcriptional regulator | nGCO | 31.51% | 46.28% |
| NP_460233 | - | putative cytoplasmic protein | + | NP_415684 | ymgB | hypothetical protein | nGCO | 41.79% | 38.15% |
| NP_460234 | - | putative cytoplasmic protein | - |  |  |  | NO HOMOLOG |  | 30.66% |
| NP_460235 | - | chorismate mutase | + |  |  |  | NO HOMOLOG |  | 47.06% |
| NP_460236 | yeaS | putative transport protein | - | NP_416312 | yeaS | neutral amino-acid efflux system | GCO | 82.07% | 46.79% |
| NP_460237 | yeaR | putative cytoplasmic protein | - | NP_416311 | yeaR | hypothetical protein | GCO | 83.19% | 49.86% |
| NP_460238 | yoaG | putative cytoplasmic protein | - | NP_416310 | yoaG | hypothetical protein | GCO | 71.66% | 48.08% |
| NP_460239 | - | putative nitric oxide reductase | - |  |  |  | NO HOMOLOG |  | 50.77% |
| NP_951050 | - | hypothetical protein | - |  |  |  | NO HOMOLOG |  | 37.09% |
| NP_460240 | yeaQ | putative inner membrane protein | - | NP_416309 | yeaQ | conserved inner membrane protein | nGCO | 65.85% | 46.18% |
| NP_460241 | yaoF | putative hemolysin | - | NP_416307 | yoaF | conserved outer membrane protein | nGCO | 63.09% | 55.55% |
| NP_460242 | - | putative periplasmic protein | - |  |  |  | NO HOMOLOG |  | 56.08% |
| NP_460243 | yeaO | putative cytoplasmic protein | - | NP_416306 | yeaO | hypothetical protein | GCO | 73.91% | 51.43% |
| NP_460244 | yeaN | putative amino acid/amine transport protein | - | NP_416305 | yeaN | predicted transporter | GCO | 70.15% | 58.31% |
| NP_460245 | yeaM | putative regulatory protein | - | NP_416304 | yeaM | predicted DNA-binding transcriptional regulator | GCO | 68.65% | 55.72% |
| NP_460246 | yeaL | putative inner membrane protein | - | NP_416303 | yeaL | conserved inner membrane protein | GCO | 66.15% | 57.03% |
| NP_460247 | - | putative inner membrane protein | - |  |  |  | NO HOMOLOG |  | 46.29% |
| NP_460248 | yeaK | putative cytoplasmic protein | + | NP_416301 | yeaK | hypothetical protein | GCO | 77.57% | 54.91% |
| NP_460249 | yeaJ | putative methyl-accepting chemotaxis protein | - | NP_416300 | yeaJ | predicted diguanylate cyclase | GCO | 67.33% | 44.84% |
| NP_460250 | yeaH | hypothetical protein | - | NP_416298 | yeaH | hypothetical protein | GCO | 86.08% | 51.82% |
| NP_460251 | yeaG | putative serine protein kinase | - | NP_416297 | yeaG | conserved protein with nucleoside triphosphate hydrolase domain | GCO | 98.60% | 50.69% |
| NP_460252 | mipA | MltA-interacting protein A | - | NP_416296 | mipA | scaffolding protein for murein synthesizing machinery | GCO | 84.27% | 51.13% |
| NP_460253 | - | arylsulfatase regulator | - | YP_026259 | aslB | predicted regulator of arylsulfatase activity | nGCO | 46.05% | 49.95% |
| NP_460254 | - | putative aldehyde reductase | - | NP_416295 | yeaE | predicted oxidoreductase | GCO | 74.37% | 55.12% |
| NP_460255 | yeaD | aldose 1-epimerase | - | NP_416294 | yeaD | hypothetical protein | GCO | 82.65% | 52.42% |
| NP_460256 | gapA | glyceraldehyde-3-phosphate dehydrogenase | - | NP_416293 | gapA | glyceraldehyde-3-phosphate dehydrogenase | GCO | 94.86% | 50.90% |
| NP_460257 | yeaA | methionine sulfoxide reductase B | - | NP_416292 | yeaA | methionine sulfoxide reductase B | GCO | 85.40% | 51.12% |
| NP_460258 | yeaC | putative cytoplasmic protein | - | NP_416291 | yeaC | hypothetical protein | GCO | 87.77% | 49.46% |
| NP_460259 | pncA | nicotinamidase/pyrazinamidase | - | NP_416282 | pncA | nicotinamidase/pyrazinamidase | GCO | 80.28% | 57.53% |
| NP_460260 | ansA | cytoplasmic asparaginase I | - | NP_416281 | ansA | cytoplasmic asparaginase I | GCO | 94.67% | 52.80% |
| NP_460261 | sppA | protease IV | - | NP_416280 | sppA | protease IV (signal peptide peptidase) | GCO | 80.58% | 55.78% |
| NP_460262 | ydjA | putative oxidoreductase | - | NP_416279 | ydjA | predicted oxidoreductase | GCO | 82.51% | 56.34% |
| NP_460263 | selD | selenophosphate synthetase | - | NP_416278 | selD | selenophosphate synthetase | GCO | 92.79% | 57.56% |
| NP_460264 | topB | DNA topoisomerase III | - | NP_416277 | topB | DNA topoisomerase III | GCO | 94.59% | 56.35% |
| NP_460265 | gdhA | glutamate dehydrogenase | - | NP_416275 | gdhA | glutamate dehydrogenase | GCO | 92.84% | 57.96% |
| NP_460266 | - | putative periplasmic protein | - | NP_416274 | ynjH | hypothetical protein | GCO | 73.33% | 54.71% |
| NP_460267 | - | putative mutator protein | - | NP_416273 | nudG | pyrimidine (deoxy)nucleoside triphosphate pyrophosphohydrolase | GCO | 77.03% | 57.31% |
| NP_460268 | xthA | exonuclease III | - | NP_416263 | xthA | exonuclease III | GCO | 92.50% | 52.66% |
| NP_460269 | argD | bifunctional N-succinyldiaminopimelate-aminotransferase/acetylornithine transaminase protein | - | NP_416262 | astC | succinylornithine transaminase, PLP-dependent | GCO | 84.40% | 56.23% |
| NP_460270 | astA | arginine succinyltransferase | - | NP_416261 | astA | arginine succinyltransferase | GCO | 91.83% | 55.07% |
| NP_460271 | astD | aldehyde dehyrogenase | - | NP_416260 | astD | succinylglutamic semialdehyde dehydrogenase | GCO | 77.61% | 59.97% |
| NP_460272 | astB | succinylarginine dihydrolase | - | NP_416259 | astB | succinylarginine dihydrolase | GCO | 78.29% | 56.10% |
| NP_460273 | astE | succinylglutamate desuccinylase | - | NP_416258 | astE | succinylglutamate desuccinylase | GCO | 67.28% | 58.10% |
| NP_460274 | spy | putative stress response protein | + | NP_416257 | spy | envelope stress induced periplasmic protein | GCO | 77.63% | 50% |
| NP_460275 | - | putative excinuclease subunit | - | NP_416255 | ydjQ | endonuclease of nucleotide excision repair | GCO | 80.76% | 54.89% |
| NP_460276 | nadE | NAD(+) synthetase | - | NP_416254 | nadE | NAD synthetase | GCO | 89.45% | 53.01% |
| NP_460277 | osmE | transcriptional activator | - | NP_416253 | osmE | DNA-binding transcriptional activator | GCO | 96.42% | 50.58% |
| NP_460278 | celA | sugar-specific enzyme IIB | - | NP_416252 | chbB | N,N'-diacetylchitobiose-specific enzyme IIB component of PTS | GCO | 95.69% | 46.10% |
| NP_460279 | celB | sugar-specific enzyme II | - | NP_416251 | chbC | N,N'-diacetylchitobiose-specific enzyme IIC component of PTS | GCO | 88.93% | 50.77% |
| NP_460280 | celC | sugar-specific enzyme III | - | NP_416250 | chbA | N,N'-diacetylchitobiose-specific enzyme IIA component of PTS | GCO | 86.73% | 49.42% |
| NP_460281 | celD | transcriptional repressor of cel operon | + | NP_416249 | chbR | DNA-binding transcriptional dual regulator | GCO | 85.71% | 43.06% |
| NP_460282 | celF | phospho-beta-glucosidase/cellobiose-6-phosphate hydrolase | + | NP_416248 | chbF | cryptic phospho-beta-glucosidase, NAD(P)-binding | GCO | 90.44% | 48.08% |
| NP_460283 | celG | hypothetical protein | + | NP_416247 | chbG | hypothetical protein | GCO | 76.19% | 54.41% |
| NP_460284 | katE | hydroperoxidase HPII | - | YP_025308 | katE | hydroperoxidase HPII(III) (catalase) | GCO | 85.73% | 54.06% |
| NP_460285 | cedA | cell division modulator protein | - | NP_416245 | cedA | cell division modulator | GCO | 82.50% | 51.02% |
| NP_460286 | ydjN | kinase/transporter-like protein | - | NP_416243 | ydjN | predicted transporter | GCO | 81.16% | 54.81% |
| NP_460287 | ydjM | putative SOS response protein | - | NP_416242 | ydjM | predicted inner membrane protein regulated by LexA | GCO | 86.66% | 53.16% |
| NP_460288 | yniC | putative enzyme | - | NP_416241 | yniC | predicted hydrolase | GCO | 90.09% | 55.00% |
| NP_460289 | yniB | putative regulatory protein | - | NP_416240 | yniB | predicted inner membrane protein | GCO | 79.21% | 45.99% |
| NP_460290 | - | putative cytoplasmic protein | - | NP_416239 | yniA | predicted phosphotransferase/kinase | GCO | 92.98% | 54.35% |
| NP_460291 | ydiZ | putative cytoplasmic protein | - | NP_416238 | ydiZ | hypothetical protein | GCO | 69.14% | 48.79% |
| NP_460292 | pfkB | 6-phosphofructokinase II | - | NP_416237 | pfkB | 6-phosphofructokinase II | GCO | 90.25% | 57.12% |
| NP_460293 | ydiY | putative outer membrane protein | - | NP_416236 | ydiY | hypothetical protein | GCO | 88.49% | 50.32% |
| NP_460294 | - | putative outer membrane protein | - |  |  |  | NO HOMOLOG |  | 47.60% |
| NP_460295 | - | putative inner membrane protein | - |  |  |  | NO HOMOLOG |  | 30.79% |
| NP_460296 | - | putative DNA/RNA non-specific endonuclease | - |  |  |  | NO HOMOLOG |  | 51.34% |
| NP_460298 | rfc | O-antigen polymerase | - |  |  |  | NO HOMOLOG |  | 33.49% |
| NP_460299 | thrS | threonyl-tRNA synthetase | - | NP_416234 | thrS | threonyl-tRNA synthetase | GCO | 96.72% | 50.49% |
| NP_460300 | infC | translation initiation factor IF-3 | - | NP_416233 | infC | translation initiation factor IF-3 | GCO | 87.22% | 47.51% |
| NP_460301 | rpmI | 50S ribosomal protein L35 | - | NP_416232 | rpmI | 50S ribosomal protein L35 | GCO | 70.76% | 48.98% |
| NP_460302 | rplT | 50S ribosomal protein L20 | - | NP_416231 | rplT | 50S ribosomal protein L20 | GCO | 89.83% | 49.85% |
| NP_460303 | pheS | phenylalanyl-tRNA synthetase alpha subunit | - | NP_416229 | pheS | phenylalanyl-tRNA synthetase alpha subunit | GCO | 98.35% | 53.86% |
| NP_460304 | pheT | phenylalanyl-tRNA synthetase beta subunit | - | NP_416228 | pheT | phenylalanyl-tRNA synthetase beta subunit | GCO | 91.06% | 53.68% |
| NP_460305 | himA | integration host factor alpha subunit | - | NP_416227 | ihfA | integration host factor subunit alpha | GCO | 98.98% | 48.33% |
| NP_460306 | btuC | vtamin B12-transporter permease | - | NP_416226 | btuC | vtamin B12-transporter permease | GCO | 70.24% | 59.32% |
| NP_460307 | btuE | vitamin B12 transport protein | - | NP_416225 | btuE | predicted glutathione peroxidase | GCO | 85.79% | 49.45% |
| NP_460308 | btuD | vitamin B12-transporter ATPase | - | NP_416224 | btuD | vitamin B12-transporter ATPase | GCO | 80.97% | 56.53% |
| NP_460309 | nlpC | lipoprotein | - | NP_416223 | nlpC | predicted lipoprotein | GCO | 86.36% | 48.60% |
| NP_460310 | ydiV | hypothetical protein | - | NP_416222 | ydiV | hypothetical protein | GCO | 51.50% | 46.07% |
| NP_460311 | ydiU | hypothetical protein | - | NP_416221 | ydiU | hypothetical protein | GCO | 82.08% | 54.33% |
| NP_460312 | ydiE | putative cytoplasmic protein | - | NP_416220 | ydiE | hypothetical protein | GCO | 60.31% | 51.56% |
| NP_460313 | aroH | 3-deoxy-7-phosphoheptulonate synthase | - | NP_416219 | aroH | 3-deoxy-D-arabino-heptulosonate-7-phosphate synthase, tryptophan repressible | GCO | 90.80% | 52.72% |
| NP_460314 | ydiA | hypothetical protein | - | NP_416218 | ydiA | hypothetical protein | GCO | 92.77% | 51.79% |
| NP_460315 | pps | phosphoenolpyruvate synthase | - | NP_416217 | pps | phosphoenolpyruvate synthase | GCO | 96.96% | 55.44% |
| NP_460316 | ydiD | hypothetical protein | - | NP_416216 | ydiD | hypothetical protein | GCO | 82.84% | 53.32% |
| NP_460317 | ydiT | putative ferredoxin | - | NP_416215 | ydiT | predicted 4Fe-4S ferredoxin-type protein | GCO | 80.41% | 48.29% |
| NP_460318 | ydiS | flavoprotein | - | NP_416214 | ydiS | predicted oxidoreductase with FAD/NAD(P)-binding domain | GCO | 73.65% | 55.24% |
| NP_460319 | ydiR | putative electron transfer flavoprotein subunit alpha | - | NP_416213 | ydiR | predicted electron transfer flavoprotein, FAD-binding | GCO | 72.75% | 53.84% |
| NP_460320 | ydiQ | putative electron transfer flavoprotein subunit beta | - | NP_416212 | ydiQ | hypothetical protein | GCO | 79.52% | 53.98% |
| NP_460321 | ydiP | putative transcriptional regulator | + | NP_416211 | ydiP | predicted DNA-binding transcriptional regulator | GCO | 80.82% | 42.64% |
| NP_460322 | ydiO | crotonobetainyl-CoA dehydrogenase | + | NP_416210 | ydiO | predicted acyl-CoA dehydrogenase | GCO | 95.30% | 49.91% |
| NP_460323 | ydiF | putative acetyl-CoA/acetoacetyl-CoA transferase beta subunit | + | NP_416209 | ydiF | fused predicted acetyl-CoA:acetoacetyl-CoA transferase: alpha subunit/beta subunit | GCO | 93.03% | 49.24% |
| NP_460324 | aroD | 3-dehydroquinate dehydratase | + | NP_416208 | aroD | 3-dehydroquinate dehydratase | GCO | 80.15% | 51.51% |
| NP_460325 | aroE | shikimate 5-dehydrogenase | + | NP_416207 | ydiB | quinate/shikimate 5-dehydrogenase, NAD(P)-binding | GCO | 85.76% | 48.21% |
| NP_460326 | ydiN | putative transport protein | + | NP_416206 | ydiN | predicted transporter | GCO | 85.60% | 46.44% |
| NP_460327 | ydiM | putative transport protein | - | NP_416205 | ydiM | predicted transporter | GCO | 79.29% | 46.86% |
| NP_460328 | ydiL | putative cytoplasmic protein | - | NP_416204 | ydiL | hypothetical protein | GCO | 59.32% | 42.22% |
| NP_460329 | ydiK | putative permease | - | NP_416203 | ydiK | predicted inner membrane protein | GCO | 71.83% | 56.03% |
| NP_460330 | ydiJ | putative oxidase | - | NP_416202 | ydiJ | predicted FAD-linked oxidoreductase | GCO | 87.90% | 54.36% |
| NP_460331 | - | hypothetical protein | - | NP_416201 | ydiI | hypothetical protein | GCO | 89.70% | 56.69% |
| NP_460332 | ydiH | putative cytoplasmic protein | - | NP_416200 | ydiH | hypothetical protein | GCO | 89.39% | 46.05% |
| NP_460333 | - | putative Na+-dicarboxylate symporter | - | NP_416243 | ydjN | predicted transporter | nGCO | 39.86% | 48.03% |
| NP_460334 | sufA | iron-sulfur cluster assembly scaffold protein | - | NP_416199 | sufA | iron-sulfur cluster assembly scaffold protein | GCO | 84.42% | 52.03% |
| NP_460335 | sufB | putative ABC transporter protein | - | NP_416198 | sufB | component of SufBCD complex | GCO | 96.16% | 52.62% |
| NP_460336 | sufC | putative transport protein | - | NP_416197 | sufC | cysteine desulfurase ATPase component | GCO | 92.33% | 52.61% |
| NP_460337 | sufD | cysteine desulfurase modulator | - | NP_416196 | sufD | component of SufBCD complex | GCO | 79.09% | 53.77% |
| NP_460338 | sufS | selenocysteine lyase | - | NP_416195 | sufS | selenocysteine lyase | GCO | 87.19% | 57.08% |
| NP_460339 | ynhA | cysteine desufuration protein SufE | - | NP_416194 | sufE | cysteine desufuration protein SufE | GCO | 87.68% | 50.83% |
| NP_460340 | ynhG | hypothetical protein | - | NP_416193 | ynhG | hypothetical protein | GCO | 85.32% | 56.98% |
| NP_460341 | lppB | putative methyl-accepting chemotaxis protein | - | NP_416192 | lpp | murein lipoprotein | GCO | 88.57% | 47.08% |
| NP_460342 | lpp | murein lipoprotein | - | NP_416192 | lpp | murein lipoprotein | GCO | 96.15% | 51.47% |
| NP_460343 | pykF | pyruvate kinase | - | NP_416191 | pykF | pyruvate kinase | GCO | 95.74% | 52.22% |
| NP_460344 | orf48 | putative amino acid permease | + | NP_415812 | puuP | putrescine importer | nGCO | 30.93% | 50.81% |
| NP_460345 | orf32 | putative proline iminopeptidase | + | NP_415529 | rarA | predicted hydrolase | nGCO | 23.17% | 51.00% |
| NP_460346 | orf245 | putative cytoplasmic protein | + |  |  |  | NO HOMOLOG |  | 42.14% |
| NP_460347 | orf408 | putative regulatory protein | + | NP_418208 | rbsK | ribokinase | nGCO | 27.83% | 45.80% |
| NP_460348 | ttrA | tetrathionate reductase complex subunit A | - |  |  |  | NO HOMOLOG |  | 57.59% |
| NP_460349 | ttrC | tetrathionate reductase complex subunit C | - |  |  |  | NO HOMOLOG |  | 55.52% |
| NP_460350 | ttrB | tetrathionate reductase complex subunit B | - | NP_418496 | nrfC | formate-dependent nitrite reductase, 4Fe4S subunit | nGCO | 38.91% | 56.30% |
| NP_460351 | ttrS | sensory histidine kinase | - | NP_418536 | basS | sensory histidine kinase in two-component regulatory system with BasR | nGCO | 25.07% | 56.77% |
| NP_460352 | ttrR | response regulator | - | NP_418125 | uhpA | DNA-binding response regulator in two-component regulatory system wtih UhpB | nGCO | 32.65% | 55.08% |
| NP_460353 | orf70 | putative cytoplasmic protein | - | NP_416190 | ydhZ | hypothetical protein | nGCO | 86.95% | 52.58% |
| NP_460354 | orf319 | putative inner membrane protein | - |  |  |  | NO HOMOLOG |  | 55.72% |
| NP_460355 | orf242 | putative regulatory protein | + | NP_416631 | mlrA | DNA-binding transcriptional regulator | nGCO | 44.10% | 53.90% |
| NP_460356 | ssrB | transcriptional activator | + | NP_416424 | uvrY | response regulator | nGCO | 30.54% | 39.59% |
| NP_460357 | ssrA | sensor kinase | + | NP_415513 | torS | hybrid sensory histidine kinase in two-component regulatory system with TorR | nGCO | 25.67% | 42.49% |
| NP_460358 | ssaB | secreted effector protein | + |  |  |  | NO HOMOLOG |  | 41.29% |
| NP_460359 | ssaC | outer membrane secretin precursor | + | NP_417850 | hofQ | predicted fimbrial transporter | nGCO | 24.71% | 42.30% |
| NP_460360 | ssaD | virulence protein | + |  |  |  | NO HOMOLOG |  | 43.64% |
| NP_460361 | ssaE | secretion system effector | + |  |  |  | NO HOMOLOG |  | 42.38% |
| NP_460362 | sseA | secretion system chaperone protein | + |  |  |  | NO HOMOLOG |  | 40.67% |
| NP_460363 | sseB | translocation machinery component | + |  |  |  | NO HOMOLOG |  | 43.31% |
| NP_460364 | sscA | secretion system chaparone | + |  |  |  | NO HOMOLOG |  | 50.63% |
| NP_460365 | sseC | translocation machinery component | + |  |  |  | NO HOMOLOG |  | 46.04% |
| NP_460366 | sseD | translocation machinery component | + |  |  |  | NO HOMOLOG |  | 49.14% |
| NP_460367 | sseE | secreted effector protein | + |  |  |  | NO HOMOLOG |  | 45.08% |
| NP_460368 | sscB | secretion system chaparone | + |  |  |  | NO HOMOLOG |  | 47.58% |
| NP_460369 | sseF | secreted effector protein | + |  |  |  | NO HOMOLOG |  | 49.04% |
| NP_460370 | sseG | secreted effector protein | + |  |  |  | NO HOMOLOG |  | 47.68% |
| NP_460371 | ssaG | type III secretion system apparatus protein | + |  |  |  | NO HOMOLOG |  | 38.42% |
| NP_460372 | ssaH | type III secretion system apparatus protein | + |  |  |  | NO HOMOLOG |  | 43.85% |
| NP_460373 | ssaI | type III secretion system apparatus protein | + |  |  |  | NO HOMOLOG |  | 43.37% |
| NP_460374 | ssaJ | needle complex inner membrane lipoprotein | + |  |  |  | NO HOMOLOG |  | 42% |
| NP_460375 | - | putative cytoplasmic protein | + |  |  |  | NO HOMOLOG |  | 46.81% |
| NP_460376 | ssaK | type III secretion system apparatus protein | + |  |  |  | NO HOMOLOG |  | 42.66% |
| NP_460377 | ssaL | type III secretion system apparatus protein | + |  |  |  | NO HOMOLOG |  | 45.62% |
| NP_460378 | ssaM | type III secretion system apparatus protein | + |  |  |  | NO HOMOLOG |  | 42.27% |
| NP_460379 | ssaV | type III secretion system apparatus protein | + | NP_416393 | flhA | flagellar biosynthesis protein A | nGCO | 26.51% | 46.52% |
| NP_460380 | ssaN | type III secretion system ATPase | + | NP_416451 | fliI | flagellum-specific ATP synthase | nGCO | 42.01% | 51.22% |
| NP_460381 | ssaO | type III secretion system apparatus protein | + |  |  |  | NO HOMOLOG |  | 43.65% |
| NP_460382 | ssaP | type III secretion system apparatus protein | + |  |  |  | NO HOMOLOG |  | 44.53% |
| NP_460383 | ssaQ | type III secretion protein | + |  |  |  | NO HOMOLOG |  | 46.64% |
| NP_460384 | ssaR | needle complex export protein | + | NP_416458 | fliP | flagellar biosynthesis protein P | nGCO | 40.20% | 42.90% |
| NP_460385 | ssaS | type III secretion system apparatus protein | + |  |  |  | NO HOMOLOG |  | 41.94% |
| NP_460386 | ssaT | type III secretion system apparatus protein | + | NP_416460 | fliR | flagellar biosynthesis protein R | nGCO | 25.13% | 38.97% |
| NP_460387 | ssaU | type III secretion system apparatus protein | + | NP_416394 | flhB | flagellar biosynthesis protein B | nGCO | 29.71% | 39.66% |
| NP_460388 | ydhE | multidrug efflux protein NorA | - | YP_025307 | mdtK | multidrug efflux protein NorM | GCO | 89.93% | 53.63% |
| NP_460389 | ribE | riboflavin synthase subunit alpha | - | NP_416179 | ribC | riboflavin synthase subunit alpha | GCO | 91.26% | 51.55% |
| NP_460390 | cfa | cyclopropane fatty acyl phospholipid synthase | - | NP_416178 | cfa | cyclopropane fatty acyl phospholipid synthase (unsaturated-phospholipid methyltransferase) | GCO | 90.31% | 48.73% |
| NP_460391 | ydhC | putative transport protein | - | YP_025306 | ydhC | predicted transporter | GCO | 75.93% | 55.05% |
| NP_460392 | ydhB | putative transcriptional regulator | - | NP_416176 | ydhB | predicted DNA-binding transcriptional regulator | GCO | 90.96% | 57.44% |
| NP_460393 | purR | pur regulon transcriptional repressor | - | NP_416175 | purR | DNA-binding transcriptional repressor, hypoxanthine-binding | GCO | 95.89% | 54.58% |
| NP_460394 | sodB | superoxide dismutase | - | NP_416173 | sodB | superoxide dismutase, Fe | GCO | 87.04% | 51.20% |
| NP_460395 | ydhO | putative cell wall-associated hydrolase | - | NP_416172 | ydhO | predicted lipoprotein | GCO | 65.20% | 51.22% |
| NP_460396 | ydhD | putative glutaredoxin protein | - | NP_416171 | ydhD | hypothetical protein | GCO | 97.39% | 50% |
| NP_460397 | rnt | ribonuclease T | - | NP_416169 | rnt | ribonuclease T | GCO | 90.90% | 53.54% |
| NP_460398 | gloA | glyoxalase I | - | NP_416168 | gloA | glyoxalase I, Ni-dependent | GCO | 91.85% | 52.20% |
| NP_460399 | nemA | N-ethylmaleimide reductase | - | NP_416167 | nemA | N-ethylmaleimide reductase, FMN-linked | GCO | 92.05% | 57.92% |
| NP_460400 | ydhM | putative transcriptional repressor | - | NP_416166 | ydhM | predicted DNA-binding transcriptional regulator | GCO | 81.65% | 55.33% |
| NP_460401 | ydhL | putative oxidoreductase | - | NP_416165 | ydhL | hypothetical protein | GCO | 83.54% | 50.41% |
| NP_460402 | ydhF | putative aldo/keto reductase | - | YP_025305 | ydhF | predicted oxidoreductase | GCO | 84.56% | 52.28% |
| NP_460403 | sodC | copper/zinc superoxide dismutase | - | NP_416163 | sodC | superoxide dismutase, Cu, Zn | GCO | 82.08% | 56.32% |
| NP_460404 | - | putative inner membrane protein | - | NP_416162 | ydhK | conserved inner membrane protein | GCO | 72.23% | 55.88% |
| NP_460405 | ydhJ | putative multidrug resistance efflux pump | - | NP_416161 | ydhJ | undecaprenyl pyrophosphate phosphatase | GCO | 80% | 50.05% |
| NP_460406 | ydhI | putative inner membrane protein | - | NP_416160 | ydhI | predicted inner membrane protein | GCO | 78.20% | 46.83% |
| NP_460407 | slyA | transcriptional regulator SlyA | - | NP_416159 | slyA | transcriptional regulator SlyA | GCO | 76.76% | 48.52% |
| NP_460408 | slyB | putative outer membrane lipoprotein | - | YP_025304 | slyB | outer membrane lipoprotein | GCO | 70.32% | 54.91% |
| NP_460409 | ydhH | putative cytoplasmic protein | - | NP_416157 | ydhH | anhydro-N-acetylmuramic acid kinase | GCO | 84.82% | 57.75% |
| NP_460410 | ydhA | putative outer membrane lipoprotein | - | NP_416156 | ydhA | predicted lipoprotein | GCO | 83.48% | 46.06% |
| NP_460411 | pdxH | pyridoxamine 5'-phosphate oxidase | - | NP_416155 | pdxH | pyridoxamine 5'-phosphate oxidase | GCO | 91.28% | 55.09% |
| NP_460412 | tyrS | tyrosyl-tRNA synthetase | - | NP_416154 | tyrS | tyrosyl-tRNA synthetase | GCO | 93.63% | 52.86% |
| NP_460413 | pdxY | pyridoxine kinase | - | NP_416153 | pdxY | pyridoxine kinase | GCO | 90.90% | 54.81% |
| NP_460414 | gst | glutathionine S-transferase | - | NP_416152 | gst | glutathionine S-transferase | GCO | 78% | 51.65% |
| NP_460415 | ydgR | putative peptide transport protein | - | NP_416151 | tppB | putative tripeptide transporter permease | GCO | 86.77% | 53.05% |
| NP_460416 | nth | endonuclease III | - | NP_416150 | nth | DNA glycosylase and apyrimidinic (AP) lyase (endonuclease III) | GCO | 90.04% | 52.20% |
| NP_460417 | ydgQ | NADH-ubiquinone oxidoreductase | - | NP_416149 | rsxE | NADH-ubiquinone oxidoreductase | GCO | 80.51% | 54.11% |
| NP_460418 | ydgP | electron transport complex protein RnfG | - | NP_416148 | rsxG | electron transport complex protein RnfG | GCO | 84.95% | 52.01% |
| NP_460419 | rnfD | electron transport complex protein RnfD | - | NP_416147 | rsxD | electron transport complex protein RnfD | GCO | 87.50% | 56.46% |
| NP_460420 | - | electron transport complex protein RnfC | - | NP_416146 | rsxC | electron transport complex protein RnfC | GCO | 84.38% | 59.87% |
| NP_460421 | ydgM | electron transport complex protein RnfB | - | NP_416145 | rsxB | electron transport complex protein RnfB | GCO | 89.06% | 55.26% |
| NP_460422 | - | Na(+)-translocating NADH-quinone reductase subunit E | - | NP_416144 | rsxA | Na(+)-translocating NADH-quinone reductase subunit E | GCO | 91.19% | 49.82% |
| NP_460423 | ydgK | putative inner membrane protein | - | NP_416143 | ydgK | conserved inner membrane protein | GCO | 66.43% | 51.24% |
| NP_460424 | ydgT | putative cytoplasmic protein | - | NP_416142 | ydgT | predicted regulator | GCO | 88.73% | 44.15% |
| NP_460425 | ydgJ | putative oxidoreductase | - | NP_416141 | ydgJ | predicted oxidoreductase | GCO | 92.19% | 55.13% |
| NP_460426 | add | adenosine deaminase | - | NP_416140 | add | adenosine deaminase | GCO | 90.03% | 55.08% |
| NP_460427 | ydgA | putative periplasmic protein | - | NP_416131 | ydgA | hypothetical protein | GCO | 78.96% | 52.48% |
| NP_460428 | manA | mannose-6-phosphate isomerase | - | NP_416130 | manA | mannose-6-phosphate isomerase | GCO | 86.44% | 54.33% |
| NP_460429 | fumA | fumarase A | - | NP_416129 | fumA | fumarate hydratase (fumarase A), aerobic Class I | GCO | 95.94% | 54.67% |
| NP_460430 | fumC | fumarate hydratase | - | NP_416128 | fumC | fumarate hydratase | GCO | 83.94% | 55.48% |
| NP_460431 | tus | DNA replication terminus site-binding protein | - | NP_416127 | tus | DNA replication terminus site-binding protein | GCO | 79.93% | 53.22% |
| NP_460432 | rstB | sensory histidine kinase | - | NP_416126 | rstB | sensory histidine kinase in two-component regulatory system with RstA | GCO | 82.21% | 54.14% |
| NP_460433 | - | putative periplasmic protein | - |  |  |  | NO HOMOLOG |  | 56.88% |
| NP_460434 | ompN | outer membrane protein N precursor | + | NP_415895 | ompN | outer membrane pore protein N, non-specific | nGCO | 76.96% | 47.61% |
| NP_460435 | rstA | response regulator | - | NP_416125 | rstA | DNA-binding response regulator in two-component regulatory system with RstB | GCO | 80.75% | 53.14% |
| NP_460436 | ydgC | putative inner membrane protein | - | NP_416124 | ydgC | conserved inner membrane protein associated with alginate biosynthesis | GCO | 79.12% | 51.78% |
| NP_460437 | ydgI | putative amino acid transporter | - | NP_416122 | ydgI | predicted arginine/ornithine antiporter transporter | GCO | 87.82% | 54.37% |
| NP_460438 | ydgH | putative periplasmic protein | - | NP_416121 | ydgH | hypothetical protein | GCO | 78.66% | 53.43% |
| NP_460439 | pntA | NAD(P) transhydrogenase subunit alpha | - | NP_416120 | pntA | NAD(P) transhydrogenase subunit alpha | GCO | 86.47% | 54.96% |
| NP_460440 | pntB | pyridine nucleotide transhydrogenase | - | NP_416119 | pntB | pyridine nucleotide transhydrogenase | GCO | 85.28% | 54.93% |
| NP_460441 | - | putative transport protein | - | NP_416118 | ydgG | predicted inner membrane protein | GCO | 80.75% | 48.79% |
| NP_460442 | ydgF | putative cationic transporter | - | NP_416117 | mdtJ | multidrug efflux system transporter | GCO | 83.89% | 44.62% |
| NP_460443 | ydgE | putative cationic transporter | - | NP_416116 | mdtI | multidrug efflux system transporter | GCO | 68.80% | 55.45% |
| NP_460444 | - | putative protease | - | NP_416115 | ydgD | predicted peptidase | GCO | 73.52% | 56.69% |
| NP_460445 | - | acid shock protein precursor | - |  |  |  | NO HOMOLOG |  | 49.82% |
| NP_460446 | ynfM | putative transport protein | - | NP_416113 | ynfM | predicted transporter | GCO | 80.33% | 55.18% |
| NP_460447 | ynfL | putative transcriptional regulator | - | NP_416112 | ynfL | predicted DNA-binding transcriptional regulator | GCO | 78.45% | 57.11% |
| NP_460448 | mlc | pts operon transcriptional repressor | - | NP_416111 | dgsA | DNA-binding transcriptional repressor | GCO | 89.40% | 51.92% |
| NP_460449 | bioD | dithiobiotin synthetase | - | NP_416110 | ynfK | predicted dethiobiotin synthetase | GCO | 93.44% | 51.86% |
| NP_460450 | - | putative voltage-gated ClC-type chloride channel ClcB | - | NP_416109 | clcB | putative voltage-gated ClC-type chloride channel ClcB | GCO | 53.28% | 58.29% |
| NP_460451 | - | proline/glycine betaine transport systems | - | NP_416633 | yehX | predicted transporter subunit: ATP-binding component of ABC superfamily | GCO | 41.91% | 53.08% |
| NP_460452 | - | putative ABC transporter permease component | - | NP_416632 | yehW | predicted transporter subunit: membrane component of ABC superfamily | GCO | 28.64% | 54.93% |
| NP_460453 | - | putative ABC transporter periplasmic component | - | NP_416635 | yehZ | predicted transporter subunit: periplasmic-binding component of ABC superfamily | nGCO | 27.71% | 52.38% |
| NP_460454 | - | putative transport system permease component | - | NP_416632 | yehW | predicted transporter subunit: membrane component of ABC superfamily | nGCO | 39% | 54.71% |
| NP_460455 | ynfI | putative anaerobic dehydrogenase component | - | NP_416108 | dmsD | twin-argninine leader-binding protein for DmsA and TorA | GCO | 76.96% | 54.47% |
| NP_460456 | - | putative dimethylsulfoxide reductase | - | NP_416107 | ynfH | oxidoreductase, membrane subunit | GCO | 74.73% | 56.87% |
| NP_460457 | - | putative dimethyl sulphoxide reductase | - | NP_415415 | dmsB | dimethyl sulfoxide reductase, anaerobic, subunit B | nGCO | 96.09% | 56.79% |
| NP_460458 | - | putative dimethyl sulphoxide reductase | - | NP_416105 | ynfF | oxidoreductase subunit | GCO | 88.76% | 55.33% |
| NP_460459 | - | putative dimethyl sulphoxide reductase chain A1 | - | NP_416104 | ynfE | oxidoreductase subunit | GCO | 84.97% | 54.21% |
| NP_460460 | ynfD | putative outer membrane protein | - | NP_416103 | ynfD | hypothetical protein | GCO | 77.21% | 54.69% |
| NP_460461 | ynfC | hypothetical protein | - | NP_416102 | ynfC | hypothetical protein | GCO | 72.03% | 50.69% |
| NP_460462 | speG | spermidine N1-acetyltransferase | - | NP_416101 | speG | spermidine N1-acetyltransferase | GCO | 91.39% | 48.12% |
| NP_460463 | ynfB | putative periplasmic protein | - | NP_416100 | ynfB | hypothetical protein | GCO | 81.41% | 52.04% |
| NP_460464 | ynfA | hypothetical protein | - | NP_416099 | ynfA | hypothetical protein | GCO | 81.48% | 56.57% |
| NP_460465 | rspA | putative dehydratase | - | NP_416098 | rspA | predicted dehydratase | GCO | 94.80% | 54.65% |
| NP_460466 | rspB | putative dehydrogenase | - | NP_416097 | rspB | predicted oxidoreductase, Zn-dependent and NAD(P)-binding | GCO | 75.22% | 51.56% |
| NP_460468 | ydfI | putative mannitol dehydrogenase | - | NP_416060 | ydfI | predicted mannonate dehydrogenase | GCO | 81.40% | 56.03% |
| NP_460469 | ydfZ | putative cytoplasmic protein | - | NP_416059 | ydfZ | hypothetical protein | GCO | 79.10% | 50.98% |
| NP_460470 | ydfH | putative regulatory protein | - | NP_416058 | ydfH | predicted DNA-binding transcriptional regulator | GCO | 83.33% | 50.65% |
| NP_460471 | ydfG | putative oxidoreductase | - | NP_416057 | ydfG | L-allo-threonine dehydrogenase, NAD(P)-binding | GCO | 88.25% | 53.54% |
| NP_460472 | dcp | dipeptidyl carboxypeptidase II | - | NP_416056 | dcp | dipeptidyl carboxypeptidase II | GCO | 77.51% | 53.74% |
| NP_460473 | - | putative cytoplasmic protein | + | NP_415775 | yciG | hypothetical protein | nGCO | 89.83% | 49.72% |
| NP_460474 | ydeJ | competence damage-inducible protein A | + | NP_416055 | ydeJ | competence damage-inducible protein A | GCO | 52.04% | 48.67% |
| NP_460475 | ydeI | putative periplasmic protein | + | NP_416054 | ydeI | hypothetical protein | GCO | 79.23% | 47.83% |
| NP_460476 | ydeE | putative transport protein | - | NP_416051 | ydeE | predicted transporter | GCO | 70.40% | 52.18% |
| NP_460477 | ydeD | putative permease | - | NP_416050 | eamA | cysteine and O-acetyl-L-serine efflux system | GCO | 68.35% | 53.59% |
| NP_460478 | marB | multiple antibiotic resistance protein | - | NP_416049 | marB | hypothetical protein | GCO | 46.47% | 50.46% |
| NP_460479 | marA | transcriptional activator | - | NP_416048 | marA | DNA-binding transcriptional dual activator of multiple antibiotic resistance | GCO | 95.23% | 46.66% |
| NP_460480 | marR | marRAB operon repressor | - | NP_416047 | marR | DNA-binding transcriptional repressor of multiple antibiotic resistance | GCO | 92.36% | 48.27% |
| NP_460481 | marC | multiple antibiotic resistance transporter | - | NP_416046 | marC | predicted transporter | GCO | 91.85% | 53.45% |
| NP_460482 | ydeA | sugar efflux transporter | - | NP_416045 | ydeA | sugar efflux transporter | GCO | 71.57% | 53.31% |
| NP_460483 | yneJ | putative transcriptional regulator | - | NP_416043 | yneJ | predicted DNA-binding transcriptional regulator | GCO | 84.45% | 56.93% |
| NP_460484 | yneI | putative succinate-semialdehyde dehydrogenase | - | NP_416042 | yneI | predicted aldehyde dehydrogenase | GCO | 75.75% | 56.94% |
| NP_460485 | yneH | glutaminase | - | NP_416041 | yneH | predicted glutaminase | GCO | 91.88% | 54.90% |
| NP_460486 | yneG | putative cytoplasmic protein | - | NP_416040 | yneG | hypothetical protein | GCO | 80.50% | 55% |
| NP_460487 | - | putative inner membrane protein | - | NP_416037 | yneE | conserved inner membrane protein | nGCO | 84.86% | 45.99% |
| NP_460488 | - | putative outer membrane protein | + |  |  |  | NO HOMOLOG |  | 48.98% |
| NP_460490 | - | putative outer membrane protein | - | NP_415895 | ompN | outer membrane pore protein N, non-specific | nGCO | 62.15% | 46.38% |
| NP_460491 | - | putative hydrogenase | - | NP_417465 | hybF | protein involved with the maturation of hydrogenases 1 and 2 | nGCO | 38.05% | 50.58% |
| NP_460492 | - | putative dehydrogenase protein | - |  |  |  | NO HOMOLOG |  | 54.27% |
| NP_460493 | - | putative hydrogenase | - | NP_415496 | hyaF | protein involved in nickel incorporation into hydrogenase-1 proteins | GCO | 30.87% | 50.37% |
| NP_460494 | - | putative hydrogenase | - | NP_415495 | hyaE | protein involved in processing of HyaA and HyaB proteins | GCO | 36.36% | 53.04% |
| NP_460495 | - | putative hydrogenase protein | - |  |  |  | NO HOMOLOG |  | 56% |
| NP_460496 | - | putative hydrogenase maturation protease | - | NP_415494 | hyaD | protein involved in processing of HyaA and HyaB proteins | GCO | 53.07% | 53.20% |
| NP_460497 | - | putative Ni/Fe hydrogenase 1 b-type cytochrome subunit | - | NP_415493 | hyaC | hydrogenase 1, b-type cytochrome subunit | GCO | 54.42% | 51.07% |
| NP_460498 | - | putative hydrogenase-1 large subunit | - | NP_415492 | hyaB | hydrogenase 1, large subunit | GCO | 66.33% | 52.63% |
| NP_460499 | - | putative hydrogenase-1 small subunit | + | NP_415491 | hyaA | hydrogenase 1, small subunit | GCO | 72.92% | 51.90% |
| NP_460500 | - | putative hydrolase | + |  |  |  | NO HOMOLOG |  | 47.71% |
| NP_460501 | - | putative regulatory protein | - | NP_418744 | uxuR | DNA-binding transcriptional repressor | nGCO | 31.83% | 47.09% |
| NP_460502 | - | putative zinc-binding dehydrogenase | + | NP_416097 | rspB | predicted oxidoreductase, Zn-dependent and NAD(P)-binding | nGCO | 31.17% | 47.66% |
| NP_460503 | - | putative transport protein | - | NP_418776 | yjiZ | predicted transporter | nGCO | 27.10% | 46.62% |
| NP_460504 | pqaA | PhoPQ-regulated protein | - |  |  |  | NO HOMOLOG |  | 41.81% |
| NP_460505 | - | putative multidrug efflux protein | - |  |  |  | NO HOMOLOG |  | 49.35% |
| NP_460506 | - | hypothetical protein | - | NP_414881 | mhpA | 3-(3-hydroxyphenyl)propionate hydroxylase | nGCO | 28.93% | 52.88% |
| NP_460507 | - | putative transcriptional regulator | - |  |  |  | NO HOMOLOG |  | 48.67% |
| NP_460508 | - | putative S-adenosylmethionine/tRNA-ribosyltransferase-isomerase | + | NP_414939 | queA | S-adenosylmethionine:tRNA ribosyltransferase-isomerase | nGCO | 28.52% | 38.51% |
| NP_460509 | - | putative translation initiation inhibitor | - | NP_418669 | yjgH | predicted mRNA endoribonuclease | nGCO | 82.94% | 49.74% |
| NP_460510 | - | putative cytoplasmic protein | - | NP_416081 | relE | Qin prophage; toxin of the RelE-RelB toxin-antitoxin system | nGCO | 62.06% | 42.10% |
| NP_460511 | - | putative cytoplasmic protein | - |  |  |  | NO HOMOLOG |  | 44.17% |
| NP_945161 | - | hypothetical protein | - |  |  |  | NO HOMOLOG |  | 40.84% |
| NP_460512 | - | putative cytoplasmic protein | - |  |  |  | NO HOMOLOG |  | 42.46% |
| NP_460513 | - | putative coiled-coil protein | + |  |  |  | NO HOMOLOG |  | 36.05% |
| NP_460514 | - | putative transcriptional regulator | - | NP_417314 | galR | DNA-binding transcriptional repressor | nGCO | 42.12% | 53.96% |
| NP_460515 | - | putative Na+/H+ antiporter | - |  |  |  | NO HOMOLOG |  | 51.30% |
| NP_460516 | - | putative aminotransferase | - | NP_416139 | malY | bifunctional beta-cystathionase, PLP-dependent/ regulator of maltose regulon | nGCO | 33.16% | 51.53% |
| NP_460517 | - | putative glycosyl hydrolase | - | NP_417889 | glgX | glycogen debranching enzyme | nGCO | 46.39% | 52.11% |
| NP_460518 | - | putative glycosyl hydrolase | - |  |  |  | NO HOMOLOG |  | 54.48% |
| NP_460519 | - | putative alpha amylase | - | NP_417889 | glgX | glycogen debranching enzyme | nGCO | 27.25% | 54.34% |
| NP_460520 | - | putative lipoprotein | - |  |  |  | NO HOMOLOG |  | 53.68% |
| NP_460521 | - | putative periplasmic transport protein | - | NP_417966 | hdeB | acid-resistance protein | nGCO | 43.92% | 44.84% |
| NP_460522 | osmC | putative envelope protein | - | NP_415999 | osmC | osmotically inducible, stress-inducible membrane protein | GCO | 92.30% | 51.62% |
| NP_460523 | yddX | putative cytoplasmic protein | + | NP_415998 | bdm | biofilm-dependent modulation protein | GCO | 88.73% | 48.61% |
| NP_460524 | rpsV | 30S ribosomal subunit protein S22 | - | NP_415997 | sra | 30S ribosomal subunit protein S22 | GCO | 86.36% | 50.69% |
| NP_460525 | sfcA | NAD-linked malate dehydrogenase | - | NP_415996 | sfcA | malate dehydrogenase, (decarboxylating, NAD-requiring) (malic enzyme) | GCO | 89.20% | 53.06% |
| NP_460526 | adhP | alcohol dehydrogenase | - | NP_415995 | adhP | alcohol dehydrogenase | GCO | 83.28% | 52.81% |
| NP_460527 | fdnI | formate dehydrogenase-N gamma subunit | - | NP_415993 | fdnI | formate dehydrogenase-N, cytochrome B556 (gamma) subunit, nitrate-inducible | GCO | 98.01% | 48.24% |
| NP_460528 | fdnH | formate dehydrogenase-N beta subunit | - | NP_415992 | fdnH | formate dehydrogenase-N, Fe-S (beta) subunit, nitrate-inducible | GCO | 93.28% | 53.55% |
| NP_460529 | fdnG | formate dehydorgenase-N alpha subunit | - | NP_415991 | fdnG | formate dehydrogenase-N, alpha subunit, nitrate-inducible | GCO | 89.45% | 54.46% |
| NP_460530 | yddG | putative permease | - | NP_415990 | yddG | predicted methyl viologen efflux pump | GCO | 81.31% | 50.78% |
| NP_460531 | nmpC | putative outer membrane porin precursor | - | NP_415085 | nmpC | DLP12 prophage; truncated outer membrane porin (pseudogene) | nGCO | 81.81% | 50.32% |
| NP_460532 | - | putative cytoplasmic protein | - |  |  |  | NO HOMOLOG |  | 54.87% |
| NP_460533 | smvA | methyl viologen resistance | - | NP_416868 | emrY | predicted multidrug efflux system | nGCO | 22.19% | 57.79% |
| NP_460534 | - | putative transcriptional regulator | - |  |  |  | NO HOMOLOG |  | 57.16% |
| NP_460535 | narU | nitrate extrusion protein | - | NP_415986 | narU | nitrate/nitrite transporter | GCO | 75.32% | 52.62% |
| NP_460536 | narZ | nitrate reductase 2 alpha subunit | - | NP_415985 | narZ | nitrate reductase 2 (NRZ), alpha subunit | GCO | 90.69% | 55.43% |
| NP_460537 | narY | nitrate reductase 2 beta subunit | - | NP_415984 | narY | nitrate reductase 2 (NRZ), beta subunit | GCO | 94.16% | 54.11% |
| NP_460538 | narW | nitrate reductase 2 delta subunit | - | NP_415983 | narW | nitrate reductase 2 (NRZ), delta subunit (assembly subunit) | GCO | 77.05% | 55.89% |
| NP_460539 | narV | nitrate reductase 2 gamma subunit | - | NP_415982 | narV | nitrate reductase 2 (NRZ), gamma subunit | GCO | 89.38% | 53.59% |
| NP_460540 | yddE | putative phenazine biosynthetic protein | - | NP_415981 | yddE | hypothetical protein | GCO | 81.69% | 53.46% |
| NP_460541 | nhoA | putative arylamine N-acetyltransferase | - | NP_415980 | nhoA | N-hydroxyarylamine O-acetyltransferase | GCO | 71.53% | 52.24% |
| NP_460542 | - | putative cytoplasmic protein | - |  |  |  | NO HOMOLOG |  | 42.81% |
| NP_460543 | ansP | L-asparagine transport protein | - | NP_415970 | ansP | L-asparagine transporter | nGCO | 88.04% | 53.48% |
| NP_460544 | - | putative outer membrane lipoprotein | - | NP_417310 | ygdR | hypothetical protein | nGCO | 45.07% | 43.69% |
| NP_460545 | - | putative periplasmic protein | - | NP_415969 | yncE | hypothetical protein | GCO | 83.85% | 51.69% |
| NP_460546 | yncD | putative outer membrane receptor | - | NP_415968 | yncD | predicted iron outer membrane transporter | GCO | 82.86% | 52.99% |
| NP_460547 | yncC | putative regulatory protein | + | NP_415967 | yncC | predicted DNA-binding transcriptional regulator | GCO | 52.51% | 46.99% |
| NP_460548 | yncB | putative NADP-dependent oxidoreductase | - | NP_415966 | yncB | predicted oxidoreductase, Zn-dependent and NAD(P)-binding | GCO | 81.10% | 53.31% |
| NP_460549 | yncA | putative acyltransferase | - | NP_415965 | yncA | predicted acyltransferase with acyl-CoA N-acyltransferase domain | GCO | 80% | 54.65% |
| NP_460550 | ydcZ | putative inner membrane protein | - | NP_415964 | ydcZ | predicted inner membrane protein | GCO | 86.57% | 55.77% |
| NP_460551 | ydcY | putative cytoplasmic protein | - | NP_415963 | ydcY | hypothetical protein | GCO | 85.71% | 57.26% |
| NP_460552 | srfA | putative virulence protein | - |  |  |  | NO HOMOLOG |  | 55.45% |
| NP_460553 | srfB | putative virulence protein | - |  |  |  | NO HOMOLOG |  | 55.16% |
| NP_460554 | srfC | putative virulence protein | - |  |  |  | NO HOMOLOG |  | 57.01% |
| NP_460555 | ydcX | putative inner membrane protein | - | NP_415962 | ydcX | predicted inner membrane protein | GCO | 82.45% | 44.31% |
| NP_460556 | ydcW | putative aldehyde dehydrogenase | - | NP_415961 | ydcW | medium chain aldehyde dehydrogenase | GCO | 77.84% | 55.39% |
| NP_460557 | ydcR | putative regulatory protein | - | NP_415956 | ydcR | fused predicted DNA-binding transcriptional regulator/predicted amino transferase | nGCO | 86.11% | 53.19% |
| NP_460558 | pdgL | periplasmic dipeptidase precursor | - | NP_416005 | ddpX | D-ala-D-ala dipeptidase, Zn-dependent | nGCO | 28.01% | 47.47% |
| NP_460560 | ugtL | hypothetical protein | + |  |  |  | NO HOMOLOG |  | 40.35% |
| NP_460561 | sifB | secreted effector protein | + |  |  |  | NO HOMOLOG |  | 37.85% |
| NP_460562 | yncJ | putative periplasmic protein | + | NP_415953 | yncJ | hypothetical protein | GCO | 76.71% | 51.51% |
| NP_460563 | ydcP | putative collagenase | - | NP_415952 | ydcP | predicted peptidase | GCO | 90.50% | 52.56% |
| NP_460564 | ydcN | putative repressor | - | NP_415951 | ydcN | predicted DNA-binding transcriptional regulator | GCO | 72.88% | 52.88% |
| NP_460565 | - | putative benzoate membrane transport protein | - | NP_415950 | ydcO | predicted benzoate transporter | GCO | 69.37% | 57.84% |
| NP_460566 | - | putative outer membrane lipoprotein | - | NP_415948 | ydcL | predicted lipoprotein | GCO | 84.68% | 49.32% |
| NP_460567 | tehB | putative methyltransferase | - | NP_415947 | tehB | predicted S-adenosyl-L-methionine-dependent methyltransferase | GCO | 84.18% | 49.91% |
| NP_460568 | tehA | K+-tellurite ethidium and proflavin transport protein | - | NP_415946 | tehA | potassium-tellurite ethidium and proflavin transporter | GCO | 78.35% | 54.93% |
| NP_460569 | ydcK | putative nucleoside-diphosphate-sugar pyrophosphorylase | - | NP_415945 | ydcK | predicted enzyme | GCO | 66.25% | 51.47% |
| NP_460570 | rimL | acetyl transferase | - | NP_415944 | rimL | ribosomal-protein-L7/L12-serine acetyltransferase | GCO | 67.97% | 50.37% |
| NP_460571 | - | putative cellulase protein | - | NP_418725 | sgcX | KpLE2 phage-like element; predicted endoglucanase with Zn-dependent exopeptidase domain | GCO | 84.67% | 55.58% |
| NP_460572 | - | putative PTS system enzymeIIB component | - | YP_588476 | sgcB | predicted enzyme IIB component of PTS | GCO | 93.47% | 50% |
| NP_460573 | - | putative PTS system enzyme IIC component | - | NP_418724 | sgcC | KpLE2 phage-like element; predicted phosphotransferase enzyme IIC component | GCO | 88.78% | 52.13% |
| NP_460574 | - | putative nucleoside triphosphatase | - | NP_418723 | sgcQ | KpLE2 phage-like element; predicted nucleoside triphosphatase | GCO | 92.91% | 53.53% |
| NP_460575 | - | putative sugar-specific PTS enzyme II | - | NP_418722 | sgcA | KpLE2 phage-like element; predicted phosphotransferase enzyme IIA component | GCO | 73.38% | 57.07% |
| NP_460576 | - | ribulose-phosphate 3-epimerase | - | NP_418721 | sgcE | ribulose-phosphate 3-epimerase | GCO | 61.42% | 56.55% |
| NP_460577 | - | putative transcriptional repressor of sgc operon | - | NP_418720 | sgcR | KpLE2 phage-like element; predicted DNA-binding transcriptional regulator | GCO | 89.80% | 51.89% |
| NP_460578 | - | cryptic aminoglycoside resistance gene | - |  |  |  | NO HOMOLOG |  | 51.82% |
| NP_460579 | - | putative oxidase | - | NP_418062 | lldD | L-lactate dehydrogenase, FMN-linked | nGCO | 31.96% | 48.29% |
| NP_460580 | - | putative periplasmic protein | + | NP_415574 | yceI | hypothetical protein | nGCO | 31.76% | 42.36% |
| NP_460581 | ydcG | putative MDO biosynthetic protein | - | NP_415941 | mdoD | glucan biosynthesis protein, periplasmic | nGCO | 89.25% | 54.77% |
| NP_460582 | - | putative carboxylesterase | - |  |  |  | NO HOMOLOG |  | 57.19% |
| NP_460583 | - | putative cytoplasmic protein | - | NP_415940 | ydcJ | hypothetical protein | GCO | 87.47% | 54.38% |
| NP_460584 | ydcI | putative transcriptional regulator | - | NP_415939 | ydcI | predicted DNA-binding transcriptional regulator | GCO | 83.33% | 54.65% |
| NP_460585 | trg | methyl-accepting chemotaxis protein III | - | NP_415938 | trg | methyl-accepting chemotaxis protein III, ribose and galactose sensor receptor | GCO | 61.61% | 54.12% |
| NP_460586 | - | alcohol dehydrogenase class III | - | NP_414890 | frmA | alcohol dehydrogenase class III/glutathione-dependent formaldehyde dehydrogenase | GCO | 77.50% | 53.35% |
| NP_460587 | - | putative cytoplasmic protein | + | NP_414891 | frmR | regulator protein that represses frmRAB operon | GCO | 52.74% | 55.07% |
| NP_460589 | - | putative inner membrane protein | + |  |  |  | NO HOMOLOG |  | 36.54% |
| NP_460590 | sseJ | secreted effector protein | + |  |  |  | NO HOMOLOG |  | 39.20% |
| NP_460591 | - | putative inner membrane protein | - |  |  |  | NO HOMOLOG |  | 46.95% |
| NP_460592 | - | putative periplasmic binding protein | + | NP_416430 | fliY | cystine transporter subunit | nGCO | 23.66% | 39.63% |
| NP_460593 | - | putative ABC transporter permease component | + | NP_416428 | yecS | predicted transporter subunit: membrane component of ABC superfamily | nGCO | 31.34% | 41.97% |
| NP_460594 | - | putative ABC-type polar amino acid transport system ATPase component | + | NP_415185 | gltL | glutamate and aspartate transporter subunit | nGCO | 39.71% | 43.44% |
| NP_460595 | - | putative ABC-type transport system membrane component | + | NP_416428 | yecS | predicted transporter subunit: membrane component of ABC superfamily | nGCO | 32.85% | 43.46% |
| NP_460596 | - | putative inner membrane protein | + |  |  |  | NO HOMOLOG |  | 44.68% |
| NP_460597 | - | putative SAM-dependent methyltransferase | - | NP_415094 | ybcY | DLP12 prophage; predicted SAM-dependent methyltransferase | nGCO | 55.71% | 48.40% |
| NP_460598 | cybB | cytochrome b561 | - | NP_415935 | cybB | cytochrome b561 | nGCO | 84% | 48.02% |
| NP_460599 | ydcF | putative inner membrane protein | - | NP_415932 | ydcF | hypothetical protein | GCO | 68.79% | 54.68% |
| NP_460600 | hrpA | ATP-dependent helicase | - | NP_415931 | hrpA | ATP-dependent helicase | GCO | 95.07% | 53.72% |
| NP_460601 | acpD | acyl carrier protein phosphodiesterase | - | NP_415930 | azoR | acyl carrier protein phosphodiesterase | GCO | 82.08% | 53.63% |
| NP_460602 | - | putative inner membrane protein | - |  |  |  | NO HOMOLOG |  | 50% |
| NP_460603 | ydbL | putative periplasmic protein | - | NP_415901 | ydbL | hypothetical protein | GCO | 78.16% | 52.16% |
| NP_460604 | ynbE | putative outer membrane lipoprotein | - | NP_415900 | ynbE | predicted lipoprotein | GCO | 73.77% | 45.83% |
| NP_460605 | ydbH | putative periplasmic protein | - | NP_415899 | ydbH | hypothetical protein | GCO | 75.05% | 55.40% |
| NP_460606 | ldhA | D-lactate dehydrogenase | - | NP_415898 | ldhA | D-lactate dehydrogenase | GCO | 90.54% | 51.71% |
| NP_460607 | hslJ | heat shock protein hslJ | - | NP_415897 | hslJ | heat-inducible protein | GCO | 69.56% | 52.31% |
| NP_460608 | - | putative cytoplasmic protein | - | YP_588453 | ydbJ | hypothetical protein | GCO | 92.15% | 57.05% |
| NP_460609 | - | hypothetical protein | - |  |  |  | NO HOMOLOG |  | 48.66% |
| NP_460610 | nifJ | putative pyruvate-flavodoxin oxidoreductase | - | NP_415896 | ydbK | fused predicted pyruvate-flavodoxin oxidoreductase: conserved protein/conserved protein/FeS binding protein | nGCO | 91.99% | 55.17% |
| NP_460611 | ynaF | putative universal stress protein | - | NP_415894 | uspF | stress-induced protein, ATP-binding protein | nGCO | 90.97% | 47.81% |
| NP_460612 | - | putative membrane transporter of cations | - | NP_415075 | emrE | multidrug efflux protein | nGCO | 46.22% | 41.88% |
| NP_460613 | ydaO | putative ATPase | - | NP_415860 | ydaO | predicted C32 tRNA thiolase | GCO | 94.53% | 50.53% |
| NP_460614 | dbpA | ATP-dependent RNA helicase | - | NP_415859 | dbpA | ATP-dependent RNA helicase, specific for 23S rRNA | GCO | 86.65% | 55.31% |
| NP_460615 | - | putative Zn transport protein | - | NP_415858 | ydaN | zinc transporter | GCO | 86.54% | 53.25% |
| NP_460616 | - | putative methyl-accepting chemotaxis protein | - | NP_418775 | tsr | methyl-accepting chemotaxis protein I, serine sensor receptor | nGCO | 36.30% | 48.91% |
| NP_460617 | ydaL | hypothetical protein | - | NP_415856 | ydaL | hypothetical protein | nGCO | 86.09% | 52.48% |
| NP_460618 | ogt | O-6-alkylguanine-DNA/cysteine-protein methyltransferase | - | NP_415851 | ogt | O-6-alkylguanine-DNA:cysteine-protein methyltransferase | GCO | 78.94% | 55.03% |
| NP_460619 | fnr | transcriptional regulator | - | NP_415850 | fnr | DNA-binding transcriptional dual regulator, global regulator of anaerobic growth | GCO | 99.20% | 50.73% |
| NP_460620 | ydaA | putative universal stress protein | - | NP_415849 | uspE | stress-induced protein | GCO | 95.87% | 51.89% |
| NP_460621 | ynaJ | putative inner membrane protein | - | NP_415848 | ynaJ | predicted inner membrane protein | GCO | 88.09% | 47.84% |
| NP_460622 | ynaI | putative integral membrane protein | - | NP_415846 | ynaI | conserved inner membrane protein | nGCO | 71.34% | 44.86% |
| NP_460623 | - | putative transcriptional regulator | - | NP_415951 | ydcN | predicted DNA-binding transcriptional regulator | nGCO | 28.65% | 51.74% |
| NP_460624 | - | putative cytoplasmic protein | - |  |  |  | NO HOMOLOG |  | 54.14% |
| NP_460625 | - | putative thiol peroxidase | + | NP_415840 | tpx | thiol peroxidase | nGCO | 30.25% | 44.44% |
| NP_460626 | - | hypothetical protein | + |  |  |  | NO HOMOLOG |  | 40.55% |
| NP_460627 | - | invasin-like protein | - |  |  |  | NO HOMOLOG |  | 51.53% |
| NP_460628 | - | putative lipoprotein | - |  |  |  | NO HOMOLOG |  | 48.22% |
| NP_460629 | - | putative regulatory protein | + | NP_414834 | ykgA | predicted DNA-binding transcriptional regulator | nGCO | 35.17% | 38.34% |
| NP_460631 | - | putative outer membrane lipoprotein | - |  |  |  | NO HOMOLOG |  | 47.05% |
| NP_460632 | - | putative regulatory protein | - | NP_418672 | yjgJ | predicted transcriptional regulator | GCO | 54.59% | 57.36% |
| NP_460633 | - | oxidoreductase | - | NP_418670 | yjgI | predicted oxidoreductase with NAD(P)-binding Rossmann-fold domain | GCO | 80.16% | 57.42% |
| NP_460634 | - | putative aldo/keto reductase | + | NP_417485 | dkgA | 2,5-diketo-D-gluconate reductase A | nGCO | 40.07% | 46.66% |
| NP_460635 | - | putative transcriptional regulator | - | NP_417978 | yhjC | predicted DNA-binding transcriptional regulator | nGCO | 35.51% | 54.74% |
| NP_460636 | - | putative 2'-hydroxyisoflavone reductase | - |  |  |  | NO HOMOLOG |  | 53.22% |
| NP_460637 | mppA | periplasmic murein tripeptide transport protein | - | NP_415845 | mppA | murein tripeptide (L-ala-gamma-D-glutamyl-meso-DAP) transporter subunit | nGCO | 91.06% | 48.82% |
| NP_460638 | ycjI | putative carboxypeptidase | - | NP_415842 | mpaA | murein peptide amidase A | GCO | 90.90% | 58.02% |
| NP_460639 | ycjG | putative chloromuconate cycloisomerase | - | NP_415841 | ycjG | L-Ala-D/L-Glu epimerase | GCO | 77.57% | 57.66% |
| NP_460640 | tpx | thiol peroxidase | - | NP_415840 | tpx | thiol peroxidase | GCO | 88.09% | 50.09% |
| NP_460641 | tyrR | transcriptional regulator | - | NP_415839 | tyrR | DNA-binding transcriptional dual regulator, tyrosine-binding | GCO | 85.18% | 53.56% |
| NP_460642 | ycjF | hypothetical protein | - | NP_415838 | ycjF | hypothetical protein | GCO | 80.16% | 56.68% |
| NP_460643 | ycjX | putative ATPase | - | NP_415837 | ycjX | conserved protein with nucleoside triphosphate hydrolase domain | GCO | 93.76% | 54.20% |
| NP_460644 | pspE | phage shock protein | - | NP_415824 | pspE | thiosulfate:cyanide sulfurtransferase (rhodanese) | GCO | 66.99% | 43.17% |
| NP_460645 | pspD | phage shock protein | - | NP_415823 | pspD | peripheral inner membrane phage-shock protein | GCO | 87.50% | 56.62% |
| NP_460646 | pspC | phage shock protein | - | NP_415822 | pspC | DNA-binding transcriptional activator | GCO | 84.03% | 52.22% |
| NP_460647 | pspB | phage shock protein B | - | NP_415821 | pspB | phage shock protein B | GCO | 72.97% | 53.77% |
| NP_460648 | pspA | phage shock protein | - | NP_415820 | pspA | regulatory protein for phage-shock-protein operon | GCO | 91.44% | 51.56% |
| NP_460649 | pspF | transcription activator | - | NP_415819 | pspF | DNA-binding transcriptional activator | GCO | 85.58% | 54.33% |
| NP_460650 | sapA | peptide transport protein | - | NP_415810 | sapA | predicted antimicrobial peptide transporter subunit | GCO | 84.15% | 54.12% |
| NP_460651 | sapB | peptide transport protein | - | NP_415809 | sapB | predicted antimicrobial peptide transporter subunit | GCO | 86.66% | 53.10% |
| NP_460652 | sapC | peptide transport protein | - | NP_415808 | sapC | predicted antimicrobial peptide transporter subunit | GCO | 85.81% | 57.91% |
| NP_460653 | sapD | peptide transport protein | - | NP_415807 | sapD | predicted antimicrobial peptide transporter subunit | GCO | 93.33% | 51.86% |
| NP_460654 | sapF | peptide transport protein | - | NP_415806 | sapF | predicted antimicrobial peptide transporter subunit | GCO | 97.76% | 54.27% |
| NP_460655 | - | hypothetical protein | - | NP_416222 | ydiV | hypothetical protein | nGCO | 29.88% | 48.24% |
| NP_460656 | - | putative inner membrane protein | + |  |  |  | NO HOMOLOG |  | 39.95% |
| NP_460657 | - | hypothetical protein | - |  |  |  | NO HOMOLOG |  | 30% |
| NP_460658 | ycjE | putative cytoplasmic protein | + |  |  |  | NO HOMOLOG |  | 49.33% |
| NP_460659 | fabI | enoyl-(acyl carrier protein) reductase | - | NP_415804 | fabI | enoyl-(acyl carrier protein) reductase | GCO | 97.70% | 54.24% |
| NP_460660 | yciW | putative cytoplasmic protein | - | NP_415803 | yciW | predicted oxidoreductase | GCO | 49.69% | 55.86% |
| NP_460661 | rnb | exoribonuclease II | - | NP_415802 | rnb | exoribonuclease II | GCO | 87.11% | 55.60% |
| NP_460662 | yciR | putative diguanylate cyclase/phosphodiesterase | - | NP_415801 | gmr | modulator of Rnase II stability | GCO | 76.71% | 50.52% |
| NP_460663 | yciT | putative regulatory protein | - | NP_415800 | yciT | predicted DNA-binding transcriptional regulator | GCO | 82.59% | 50.33% |
| NP_460664 | osmB | lipoprotein B | - |  |  |  | NO HOMOLOG |  | 58.90% |
| NP_460665 | yciH | hypothetical protein | - | NP_415798 | yciH | translation intiation factor Sui1 | GCO | 77.77% | 46.48% |
| NP_460666 | pyrF | orotidine 5'-phosphate decarboxylase | - | NP_415797 | pyrF | orotidine 5'-phosphate decarboxylase | GCO | 80% | 56.50% |
| NP_460667 | yciM | putative N-acetylglucosaminyl transferase | - | NP_415796 | yciM | hypothetical protein | GCO | 93.40% | 51.70% |
| NP_460668 | yciS | putative inner membrane protein | - | NP_415795 | yciS | conserved inner membrane protein | GCO | 80.39% | 51.45% |
| NP_460669 | pgpB | phosphatidylglycerophosphate phosphatase B | - | NP_415794 | pgpB | phosphatidylglycerophosphatase B | GCO | 80.59% | 56.47% |
| NP_460670 | ribA | GTP cyclohydrolase II protein | - | NP_415793 | ribA | GTP cyclohydrolase II protein | GCO | 95.40% | 50.08% |
| NP_460671 | acnA | aconitate hydratase | - | NP_415792 | acnA | aconitate hydratase | GCO | 91.68% | 55.75% |
| NP_460672 | cysB | transcriptional regulator for cysteine regulon | - | NP_415791 | cysB | DNA-binding transcriptional dual regulator, O-acetyl-L-serine-binding | GCO | 95.06% | 51.89% |
| NP_460673 | topA | DNA topoisomerase I | - | NP_415790 | topA | DNA topoisomerase I | GCO | 92.60% | 54.04% |
| NP_460674 | yciN | putative cytoplasmic protein | - | NP_415789 | yciN | hypothetical protein | GCO | 92.77% | 47.61% |
| NP_460675 | sohB | putative peptidase | - | NP_415788 | sohB | predicted inner membrane peptidase | GCO | 76.50% | 54.05% |
| NP_460676 | yciK | short chain dehydrogenase | - | NP_415787 | yciK | short chain dehydrogenase | GCO | 88.93% | 56.82% |
| NP_460677 | btuR | cob(I)yrinic acid a,c-diamide adenosyltransferase | - | NP_415786 | btuR | cob(I)yrinic acid a,c-diamide adenosyltransferase | GCO | 81.12% | 53.97% |
| NP_460678 | yciL | putative ribosomal large subunit pseudouridine synthase | - | NP_415785 | rluB | 23S rRNA pseudouridylate synthase | GCO | 97.49% | 56.84% |
| NP_460679 | yciO | putative dsRNA-binding protein | - | NP_415783 | yciO | hypothetical protein | GCO | 93.20% | 52.49% |
| NP_460680 | trpH | hypothetical protein | - | NP_415782 | yciV | hypothetical protein | GCO | 91.60% | 56.00% |
| NP_460681 | trpL | trp operon leader peptide | - |  |  |  | NO HOMOLOG |  | 53.33% |
| NP_460682 | trpE | anthranilate synthase component I | - | NP_415780 | trpE | anthranilate synthase component I | GCO | 84.03% | 57.96% |
| NP_460683 | trpD | anthranilate phosphoribosyltransferase | - | NP_415779 | trpD | bifunctional indole-3-glycerol-phosphate synthase/anthranilate phosphoribosyltransferase | GCO | 90.77% | 57.33% |
| NP_460684 | trpC | bifunctional indole-3-glycerol phosphate synthase/phosphoribosylanthranilate isomerase | - | NP_415778 | trpC | bifunctional indole-3-glycerol phosphate synthase/phosphoribosylanthranilate isomerase | GCO | 85.61% | 55.18% |
| NP_460685 | trpB | tryptophan synthase subunit beta | - | NP_415777 | trpB | tryptophan synthase subunit beta | GCO | 90.93% | 56.44% |
| NP_460686 | trpA | tryptophan synthase subunit alpha | - | NP_415776 | trpA | tryptophan synthase subunit alpha | GCO | 78.35% | 57.12% |
| NP_460687 | yciG | putative cytoplasmic protein | + | NP_415775 | yciG | hypothetical protein | GCO | 88.33% | 50.81% |
| NP_460688 | yciF | putative cytoplasmic protein | + | NP_415774 | yciF | hypothetical protein | GCO | 79.51% | 42.85% |
| NP_460689 | yciE | putative cytoplasmic protein | + | NP_415773 | yciE | hypothetical protein | GCO | 85.71% | 42.60% |
| NP_460690 | - | putative catalase | + |  |  |  | NO HOMOLOG |  | 53.35% |
| NP_460691 | ompW | outer membrane protein W precursor | - | NP_415772 | ompW | outer membrane protein W | nGCO | 80.66% | 53.20% |
| NP_460692 | - | putative ferredoxin | - | NP_414822 | ykgJ | predicted ferredoxin | nGCO | 74.31% | 51.11% |
| NP_460693 | yciC | hypothetical protein | - | NP_415771 | yciC | hypothetical protein | GCO | 72.46% | 51.07% |
| NP_460694 | yciB | putative intracellular septation protein | - | NP_415770 | yciB | intracellular septation protein A | GCO | 93.29% | 47.03% |
| NP_460695 | yciA | putative Acyl-CoA hydrolase | - | NP_415769 | yciA | predicted hydrolase | GCO | 90.90% | 54.22% |
| NP_460696 | tonB | periplasmic protein | - |  |  |  | NO HOMOLOG |  | 57.75% |
| NP_460697 | yciI | putative cytoplasmic protein | - | NP_415767 | yciI | predicted enzyme | nGCO | 91.83% | 54.54% |
| NP_460698 | cls | cardiolipin synthetase | - | NP_415765 | cls | cardiolipin synthetase | nGCO | 94.44% | 53.66% |
| NP_460700 | - | putative voltage-gated potassium channel | - |  |  |  | NO HOMOLOG |  | 48.38% |
| NP_460701 | oppF | oligopeptide transport protein | - | NP_415763 | oppF | oligopeptide transporter subunit | GCO | 93.41% | 54.12% |
| NP_460702 | oppD | oligopeptide transporter ATP-binding component | - | YP_025300 | oppD | oligopeptide transporter ATP-binding component | GCO | 92.28% | 52.48% |
| NP_460703 | oppC | oligopeptide transport protein | - | NP_415761 | oppC | oligopeptide transporter subunit | GCO | 84.76% | 52.69% |
| NP_460704 | oppB | oligopeptide permease ABC transporter membrane component | - | NP_415760 | oppB | oligopeptide permease ABC transporter membrane protein | GCO | 96.07% | 50.81% |
| NP_460705 | oppA | oligopeptide transport protein | - | NP_415759 | oppA | oligopeptide transporter subunit | GCO | 82.32% | 48.28% |
| NP_460707 | ychE | putative integral membrane protein | - | NP_415758 | ychE | predicted inner membrane protein | GCO | 93.95% | 50.61% |
| NP_460708 | adhE | iron-dependent alcohol dehydrogenase | - | NP_415757 | adhE | fused acetaldehyde-CoA dehydrogenase/iron-dependent alcohol dehydrogenase/pyruvate-formate lyase deactivase | GCO | 88.51% | 53.19% |
| NP_460709 | tdk | thymidine kinase | - | NP_415754 | tdk | thymidine kinase | GCO | 92.64% | 48.86% |
| NP_460710 | hns | DNA-binding protein HLP-II | - | NP_415753 | hns | global DNA-binding transcriptional dual regulator H-NS | GCO | 67.88% | 45.41% |
| NP_460711 | galU | glucose-1-phosphate uridylyltransferase | - | NP_415752 | galU | glucose-1-phosphate uridylyltransferase | GCO | 97.01% | 49.61% |
| NP_460712 | hnr | response regulator | - | NP_415751 | rssB | response regulator of RpoS | GCO | 81.89% | 51.08% |
| NP_460713 | ychK | putative phosphoesterase | - | NP_415750 | rssA | hypothetical protein | GCO | 83.72% | 50.88% |
| NP_460714 | ychJ | hypothetical protein | - | NP_415749 | ychJ | hypothetical protein | GCO | 77.37% | 48.80% |
| NP_460715 | purU | formyltetrahydrofolate deformylase | - | NP_415748 | purU | formyltetrahydrofolate deformylase | GCO | 92.50% | 50.41% |
| NP_460716 | - | tetratricopeptide repeat protein | - | NP_415177 | ybeQ | hypothetical protein | nGCO | 31.80% | 49.47% |
| NP_460717 | narI | nitrate reductase 1 gamma subunit | - | NP_415745 | narI | nitrate reductase 1, gamma (cytochrome b(NR)) subunit | GCO | 88.88% | 55.16% |
| NP_460718 | narJ | nitrate reductase 1 delta subunit | - | NP_415744 | narJ | molybdenum-cofactor-assembly chaperone subunit (delta subunit) of nitrate reductase 1 | GCO | 88.55% | 55.27% |
| NP_460719 | narH | nitrate reductase 1 beta subunit | - | NP_415743 | narH | nitrate reductase 1, beta (Fe-S) subunit | GCO | 92.95% | 55.72% |
| NP_460720 | narG | nitrate reductase 1 alpha subunit | - | NP_415742 | narG | nitrate reductase 1, alpha subunit | GCO | 95.34% | 56.25% |
| NP_460721 | narK | nitrite extrusion protein | - | NP_415741 | narK | nitrate/nitrite transporter | GCO | 81.16% | 54.29% |
| NP_460722 | narX | sensory histidine kinase | - | NP_415740 | narX | sensory histidine kinase in two-component regulatory system with NarL | GCO | 86.12% | 54.03% |
| NP_460723 | narL | response regulator | - | NP_415739 | narL | DNA-binding response regulator in two-component regulatory system with NarX (or NarQ) | GCO | 89.81% | 53.76% |
| NP_460724 | ychP | putative invasin | - | NP_415738 | ychP | predicted invasin | GCO | 77.69% | 53.47% |
| NP_460725 | ychN | putative sulfur reduction protein | - | NP_415737 | ychN | hypothetical protein | GCO | 92.30% | 51.97% |
| NP_460726 | chaB | cation transport regulator | - | NP_415735 | chaB | cation transport regulator | GCO | 72% | 45.45% |
| NP_460727 | chaA | sodium-calcium/proton antiporter | - | NP_415734 | chaA | calcium/sodium:proton antiporter | GCO | 82.24% | 50.68% |
| NP_460728 | kdsA | 2-dehydro-3-deoxyphosphooctonate aldolase | - | NP_415733 | kdsA | 2-dehydro-3-deoxyphosphooctonate aldolase | GCO | 93.61% | 55.43% |
| NP_460729 | ychA | putative transcriptional regulator | - | NP_415732 | ychA | predicted transcriptional regulator | GCO | 85.87% | 49.01% |
| NP_460730 | sirC | transcriptional regulator | - | NP_415731 | ychQ | predicted transcriptional regulator | GCO | 73.43% | 51.28% |
| NP_460731 | hemK | N5-glutamine S-adenosyl-L-methionine-dependent methyltransferase | - | NP_415730 | prmC | N5-glutamine S-adenosyl-L-methionine-dependent methyltransferase | GCO | 69.45% | 58.75% |
| NP_460732 | prfA | peptide chain release factor 1 | - | NP_415729 | prfA | peptide chain release factor 1 | GCO | 93.61% | 57.52% |
| NP_460733 | hemA | glutamyl-tRNA reductase | - | NP_415728 | hemA | glutamyl-tRNA reductase | GCO | 94.49% | 56.72% |
| NP_460734 | lolB | outer membrane lipoprotein LolB precursor | - | NP_415727 | lolB | outer membrane lipoprotein LolB precursor | GCO | 91.78% | 55.28% |
| NP_460735 | ipk | 4-diphosphocytidyl-2-C-methyl-D-erythritol kinase | - | NP_415726 | ispE | 4-diphosphocytidyl-2-C-methyl-D-erythritol kinase | GCO | 87.63% | 54.46% |
| NP_460736 | prsA | ribose-phosphate pyrophosphokinase | - | NP_415725 | prsA | ribose-phosphate pyrophosphokinase | GCO | 99.36% | 54.53% |
| NP_460737 | ychM | putative transport protein | - | NP_415724 | ychM | predicted transporter | GCO | 74.59% | 57.09% |
| NP_460738 | ychH | putative inner membrane protein | - | NP_415723 | ychH | predicted inner membrane protein | GCO | 75% | 50.53% |
| NP_460739 | pth | peptidyl-tRNA hydrolase | - | NP_415722 | pth | peptidyl-tRNA hydrolase | GCO | 80.92% | 51.79% |
| NP_460740 | ychF | putative GTP-binding protein | - | NP_415721 | ychF | translation-associated GTPase | GCO | 90.08% | 52.83% |
| NP_460741 | - | putative cytoplasmic protein | + |  |  |  | NO HOMOLOG |  | 38.70% |
| NP_460742 | - | hydrogenase-1 small subunit | - | NP_415491 | hyaA | hydrogenase 1, small subunit | GCO | 84.67% | 56.92% |
| NP_460743 | - | hydrogenase-1 large subunit | - | NP_415492 | hyaB | hydrogenase 1, large subunit | GCO | 91.12% | 55.18% |
| NP_460744 | - | putative Ni/Fe hydrogenase 1 b-type cytochrome subunit | - | NP_415493 | hyaC | hydrogenase 1, b-type cytochrome subunit | GCO | 83.04% | 55.32% |
| NP_460745 | - | putative hydrogenase maturation protease | - | NP_415494 | hyaD | protein involved in processing of HyaA and HyaB proteins | GCO | 79.16% | 57.95% |
| NP_460746 | - | putative chaperone | - | NP_415495 | hyaE | protein involved in processing of HyaA and HyaB proteins | GCO | 73.60% | 57.53% |
| NP_460747 | - | putative hydrogenase-1 protein | - | NP_415496 | hyaF | protein involved in nickel incorporation into hydrogenase-1 proteins | GCO | 70.96% | 59.59% |
| NP_460748 | - | putative cytochrome oxidase subunit I | - | NP_415497 | appC | cytochrome bd-II oxidase, subunit I | GCO | 86.90% | 55.53% |
| NP_460749 | - | putative cytochrome oxidase subunit II | - | NP_415498 | appB | cytochrome bd-II oxidase, subunit II | GCO | 67.19% | 55.32% |
| NP_460750 | - | putative periplasmic protein | - |  |  |  | NO HOMOLOG |  | 48.88% |
| NP_460751 | - | putative glutamic dehyrogenase-like protein | - | NP_416275 | gdhA | glutamate dehydrogenase | nGCO | 32.32% | 55.50% |
| NP_460752 | treA | trehalase | - | NP_415715 | treA | periplasmic trehalase | nGCO | 77.46% | 55.34% |
| NP_460753 | ymgE | putative transglycosylase-associated protein | - | NP_415713 | ymgE | predicted inner membrane protein | GCO | 75.90% | 56.47% |
| NP_460754 | ycgR | putative inner membrane protein | - | NP_415712 | ycgR | protein involved in flagellar function | GCO | 72.54% | 52.65% |
| NP_460755 | emtA | membrane-bound lytic murein transglycosylase E | - | NP_415711 | emtA | lytic murein endotransglycosylase E | GCO | 90.14% | 53.26% |
| NP_460756 | ycgQ | putative microcin C7 resistance protein | - | NP_415710 | ldcA | L,D-carboxypeptidase A | GCO | 79.93% | 56.28% |
| NP_460757 | ycgO | cell volume regulation protein CvrA | - | NP_415709 | cvrA | potassium/proton antiporter | GCO | 82.66% | 54.72% |
| NP_460758 | alr | alanine racemase | - | NP_415708 | dadX | alanine racemase | GCO | 85.91% | 57.42% |
| NP_460759 | dadA | D-amino acid dehydrogenase small subunit | - | NP_415707 | dadA | D-amino acid dehydrogenase small subunit | GCO | 87.26% | 56.58% |
| NP_460760 | ycgB | putative cytoplasmic protein | - | NP_415706 | ycgB | hypothetical protein | GCO | 96.84% | 49.83% |
| NP_460761 | fadR | fatty acid metabolism regulator | - | NP_415705 | fadR | fatty acid metabolism regulator | GCO | 97.07% | 52.50% |
| NP_460762 | nhaB | Na+/H+ antiporter | - | NP_415704 | nhaB | sodium/proton antiporter | GCO | 78.75% | 52.94% |
| NP_460763 | dsbB | disulfide bond formation protein B | - | NP_415703 | dsbB | disulfide bond formation protein B | GCO | 78.97% | 54.80% |
| NP_460764 | - | putative cytoplasmic protein | - | NP_416311 | yeaR | hypothetical protein | nGCO | 42.85% | 51.75% |
| NP_460765 | - | putative cytoplasmic protein | - | YP_588446 | gnsA | predicted regulator of phosphatidylethanolamine synthesis | nGCO | 61.40% | 45.97% |
| NP_460766 | - | putative cytoplasmic protein | - |  |  |  | NO HOMOLOG |  | 49.27% |
| NP_460767 | ycgN | hypothetical protein | + | NP_415699 | ycgN | hypothetical protein | GCO | 93.24% | 49.13% |
| NP_460768 | ycgM | putative fumarylacetoacetate hydrolase | - | NP_415698 | ycgM | predicted isomerase/hydrolase | GCO | 84.93% | 53.18% |
| NP_460769 | ycgL | putative cytoplasmic protein | - | NP_415697 | ycgL | hypothetical protein | GCO | 84.46% | 45.04% |
| NP_460770 | minC | septum formation inhibitor | - | NP_415694 | minC | septum formation inhibitor | GCO | 84.25% | 51.12% |
| NP_460771 | minD | cell division inhibitor protein | - | NP_415693 | minD | membrane ATPase of the MinC-MinD-MinE system | GCO | 91.85% | 51.78% |
| NP_460772 | minE | cell division topological specificity factor MinE | - | NP_415692 | minE | cell division topological specificity factor MinE | GCO | 97.72% | 43.44% |
| NP_460773 | rnd | RNase D | - | NP_416318 | rnd | ribonuclease D | GCO | 79.67% | 55.05% |
| NP_460774 | fadD | acyl-CoA synthase | - | NP_416319 | fadD | acyl-CoA synthase | GCO | 93.76% | 52.31% |
| NP_460775 | slp | putative outer membrane protein | - | NP_416320 | yeaY | predicted lipoprotein | GCO | 80.31% | 56.18% |
| NP_460776 | yeaZ | putative molecular chaperone | - | NP_416321 | yeaZ | predicted peptidase | GCO | 79.22% | 60.20% |
| NP_460777 | yoaA | putative DNA helicase | - | NP_416322 | yoaA | conserved protein with nucleoside triphosphate hydrolase domain | GCO | 95.27% | 56.41% |
| NP_460778 | yoaB | putative translation initiation inhibitor | - | NP_416323 | yoaB | hypothetical protein | GCO | 92.98% | 51.01% |
| NP_460779 | yoaH | hypothetical protein | - | NP_416325 | yoaH | hypothetical protein | GCO | 89.83% | 50.55% |
| NP_460780 | pabB | para-aminobenzoate synthase component I | - | NP_416326 | pabB | para-aminobenzoate synthase component I | GCO | 75.66% | 55.23% |
| NP_460781 | yeaB | putative NTP pyrophosphohydrolase | - | NP_416327 | yeaB | predicted NUDIX hydrolase | GCO | 87.50% | 58.89% |
| NP_460782 | sdaA | L-serine deaminase I/L-threonine deaminase I | - | NP_416328 | sdaA | L-serine deaminase I | GCO | 92.29% | 55.53% |
| NP_460783 | - | putative diguanylate cyclase/phosphodiesterase | - | NP_416329 | yoaD | predicted phosphodiesterase | GCO | 74.76% | 53.24% |
| NP_460784 | yoaE | putative inner membrane protein | - | NP_416330 | yoaE | fused predicted membrane protein/conserved protein | GCO | 81.31% | 55.74% |
| NP_460785 | - | putative cytoplasmic protein | - |  |  |  | NO HOMOLOG |  | 41.96% |
| NP_460786 | manX | mannose-specific enzyme IIAB | - | NP_416331 | manX | fused mannose-specific PTS enzymes: IIA component/IIB component | GCO | 87.30% | 52.83% |
| NP_460787 | manY | mannose-specific enzyme IIC | - | NP_416332 | manY | mannose-specific enzyme IIC component of PTS | GCO | 81.95% | 56.30% |
| NP_460788 | manZ | mannose-specific enzyme IID | - | NP_416333 | manZ | mannose-specific enzyme IID component of PTS | GCO | 89.47% | 54.58% |
| NP_460789 | - | hypothetical protein | - | NP_416334 | yobD | hypothetical protein | GCO | 78.94% | 45.01% |
| NP_460790 | yebN | putative transport protein | - | NP_416335 | yebN | conserved inner membrane protein | GCO | 78.19% | 52.49% |
| NP_460791 | rrmA | 23S rRNA m1G745 methyltransferase | - | NP_416336 | rrmA | 23S rRNA m1G745 methyltransferase | GCO | 80.22% | 56.17% |
| NP_460792 | - | putative penicillin-binding protein 3 | - | NP_414626 | ftsI | transpeptidase involved in septal peptidoglycan synthesis (penicillin-binding protein 3) | nGCO | 59.33% | 52.74% |
| NP_460793 | cspC | cold shock protein | - | NP_416337 | cspC | stress protein, member of the CspA-family | GCO | 100% | 47.14% |
| NP_460794 | yobF | putative cytoplasmic protein | - | NP_416338 | yobF | hypothetical protein | GCO | 74.46% | 40.27% |
| NP_460795 | - | hypothetical protein | - | NP_416339 | yebO | hypothetical protein | GCO | 65.26% | 49.30% |
| NP_460796 | yobG | putative inner membrane protein | - | NP_416340 | yobG | hypothetical protein | GCO | 68.08% | 43.75% |
| NP_460797 | - | hypothetical protein | - | YP_588456 | yobH | hypothetical protein | GCO | 73.41% | 52.50% |
| NP_460798 | kdgR | putative transcriptional repressor | - | NP_416341 | kdgR | predicted DNA-binding transcriptional regulator | GCO | 96.19% | 49.49% |
| NP_460799 | - | putative transport protein | - | NP_416342 | yebQ | predicted transporter | GCO | 82.27% | 55.02% |
| NP_460800 | htpX | heat shock protein HtpX | - | NP_416343 | htpX | heat shock protein HtpX | GCO | 96.24% | 52.94% |
| NP_460801 | prc | carboxy-terminal protease | - | NP_416344 | prc | carboxy-terminal protease for penicillin-binding protein 3 | GCO | 86.95% | 51.73% |
| NP_460802 | proQ | putative solute/DNA competence effector | - | YP_026161 | proQ | putative solute/DNA competence effector | GCO | 73.70% | 53.71% |
| NP_460803 | yebR | putative nucleotide-binding protein | - | NP_416346 | yebR | hypothetical protein | GCO | 83.81% | 51.11% |
| NP_460804 | yebS | putative inner membrane protein | - | NP_416347 | yebS | conserved inner membrane protein | GCO | 76.34% | 52.33% |
| NP_460805 | - | putative inner membrane protein | - | NP_416348 | yebT | hypothetical protein | GCO | 88.71% | 54.65% |
| NP_460806 | yebU | putative rRNA methyltransferase | - | NP_416349 | yebU | predicted methyltransferase | GCO | 85.38% | 55.13% |
| NP_460807 | - | putative cytoplasmic protein | - | NP_416350 | yebV | hypothetical protein | GCO | 88.46% | 50.83% |
| NP_460808 | yebW | putative inner membrane lipoprotein | - | NP_416351 | yebW | hypothetical protein | GCO | 87.30% | 47.91% |
| NP_460809 | prpA | serine/threonine protein phosphatase | - | NP_416352 | pphA | serine/threonine-specific protein phosphatase 1 | GCO | 63.30% | 52.53% |
| NP_460810 | - | putative inner membrane protein | - |  |  |  | NO HOMOLOG |  | 49.09% |
| NP_460811 | sopE2 | type III-secreted effector protein | + |  |  |  | NO HOMOLOG |  | 41.77% |
| NP_460812 | - | putative cytoplasmic protein | + | NP_415679 | ycgX | hypothetical protein | nGCO | 40.94% | 34.84% |
| NP_945162 | - | hypothetical protein | - |  |  |  | NO HOMOLOG |  | 50.73% |
| NP_460813 | - | putative acetyltransferase | - |  |  |  | NO HOMOLOG |  | 42.85% |
| NP_460814 | - | putative cytoplasmic protein | - |  |  |  | NO HOMOLOG |  | 44.44% |
| NP_460815 | - | putative cytoplasmic protein | - |  |  |  | NO HOMOLOG |  | 42.52% |
| NP_460818 | pagO | integral membrane protein | + | NP_416468 | yedA | predicted inner membrane protein | nGCO | 23.92% | 41.20% |
| NP_460819 | - | putative inner membrane protein | - |  |  |  | NO HOMOLOG |  | 40.25% |
| NP_460820 | - | putative inner membrane protein | + |  |  |  | NO HOMOLOG |  | 43.25% |
| NP_460822 | pagK | PagK | - |  |  |  | NO HOMOLOG |  | 37.31% |
| NP_460823 | mig-3 | phage-tail assembly-like protein | - | NP_415891 | tfaR | Rac prophage; predicted tail fiber assembly protein | nGCO | 50.88% | 50.68% |
| NP_945163 | - | hypothetical protein | - |  |  |  | NO HOMOLOG |  | 50.37% |
| NP_460824 | - | lytic enzyme | - |  |  |  | NO HOMOLOG |  | 37.50% |
| NP_460825 | - | phage-tail assembly-like protein | - | NP_416071 | ydfP | Qin prophage; conserved protein | nGCO | 59.61% | 52.24% |
| NP_460826 | - | hypothetical protein | - |  |  |  | NO HOMOLOG |  | 53.12% |
| NP_460827 | - | RecE-like protein | - |  |  |  | NO HOMOLOG |  | 52.32% |
| NP_460829 | - | putative cytoplasmic protein | - |  |  |  | NO HOMOLOG |  | 43.33% |
| NP_460830 | - | hypothetical protein | - | NP_416353 | yebY | hypothetical protein | GCO | 82.14% | 53.38% |
| NP_460831 | - | putative inner membrane protein | - | NP_416354 | yebZ | predicted inner membrane protein | GCO | 58.62% | 52.73% |
| NP_460832 | yobA | putative copper resistance protein | - | NP_416355 | yobA | hypothetical protein | GCO | 81% | 53.86% |
| NP_460833 | holE | DNA polymerase III theta subunit | - | NP_416356 | holE | DNA polymerase III, theta subunit | GCO | 88.15% | 52.81% |
| NP_460834 | - | putative amidohydrolase | - | NP_416357 | yobB | hypothetical protein | GCO | 65.59% | 52.35% |
| NP_460835 | exoX | exodeoxyribonuclease X | - | NP_416358 | exoX | exodeoxyribonuclease X | GCO | 90.86% | 51.93% |
| NP_460836 | ptrB | protease II | - | NP_416359 | ptrB | protease II | GCO | 85.58% | 51.21% |
| NP_460837 | yebE | putative inner membrane protein | - | NP_416360 | yebE | hypothetical protein | GCO | 72.35% | 53.18% |
| NP_460838 | yebF | putative periplasmic protein | - | NP_416361 | yebF | hypothetical protein | GCO | 81.52% | 53.38% |
| NP_460839 | yebG | DNA damage-inducible protein | - | NP_416362 | yebG | conserved protein regulated by LexA | GCO | 79.16% | 51.89% |
| NP_460840 | purT | 5'-phosphoribosylglycinamide transformylase | - | NP_416363 | purT | phosphoribosylglycinamide formyltransferase 2 | GCO | 84.94% | 56.82% |
| NP_460841 | eda | keto-hydroxyglutarate-aldolase/keto-deoxy-phosphogluconate aldolase | - | NP_416364 | eda | keto-hydroxyglutarate-aldolase/keto-deoxy-phosphogluconate aldolase | GCO | 97.16% | 57.63% |
| NP_460842 | edd | phosphogluconate dehydratase | - | NP_416365 | edd | phosphogluconate dehydratase | GCO | 91.37% | 55.84% |
| NP_460843 | zwf | glucose-6-phosphate 1-dehydrogenase | - | NP_416366 | zwf | glucose-6-phosphate 1-dehydrogenase | GCO | 97.35% | 53.86% |
| NP_460844 | yebK | putative transcriptional regulator | - | NP_416367 | yebK | predicted DNA-binding transcriptional regulator | GCO | 92.38% | 51.37% |
| NP_460845 | pykA | pyruvate kinase | - | NP_416368 | pykA | pyruvate kinase | GCO | 95.41% | 55.23% |
| NP_460846 | msbB | lipid A biosynthesis lauroyl acyltransferase | - | NP_416369 | lpxM | lipid A biosynthesis (KDO)2-(lauroyl)-lipid IVA acyltransferase | GCO | 88.85% | 53.08% |
| NP_460847 | yebA | putative peptidase | - | NP_416370 | yebA | predicted peptidase | GCO | 92.27% | 54.69% |
| NP_460848 | znuA | high-affinity Zn transport protein | - | NP_416371 | znuA | high-affinity zinc transporter periplasmic component | GCO | 83.43% | 49.90% |
| NP_460849 | znuC | high-affinity Zn transport protein | - | NP_416372 | znuC | high-affinity zinc transporter ATPase | GCO | 95.21% | 53.30% |
| NP_460850 | znuB | high-affinity Zn transport protein | - | NP_416373 | znuB | high-affinity zinc transporter membrane component | GCO | 76.24% | 55.47% |
| NP_460851 | ruvB | Holliday junction DNA helicase RuvB | - | NP_416374 | ruvB | Holliday junction DNA helicase B | GCO | 96.42% | 57.17% |
| NP_460852 | ruvA | Holliday junction DNA helicase motor protein | - | NP_416375 | ruvA | Holliday junction DNA helicase motor protein | GCO | 89.16% | 54.73% |
| NP_460853 | - | putative cytoplasmic protein | - |  |  |  | NO HOMOLOG |  | 44.59% |
| NP_460854 | yebB | putative periplasmic protein | + | NP_416376 | yebB | hypothetical protein | GCO | 79.48% | 48.83% |
| NP_460855 | ruvC | Holliday junction resolvase | - | NP_416377 | ruvC | Holliday junction resolvase | GCO | 88.43% | 55.93% |
| NP_460856 | yebC | hypothetical protein | - | NP_416378 | yebC | hypothetical protein | GCO | 98.37% | 53.98% |
| NP_460857 | ntpA | dATP pyrophosphohydrolase | - | NP_416379 | nudB | dATP pyrophosphohydrolase | GCO | 89.33% | 52.98% |
| NP_460858 | aspS | aspartyl-tRNA synthetase | - | NP_416380 | aspS | aspartyl-tRNA synthetase | GCO | 94.40% | 56.62% |
| NP_460859 | yecD | putative isochorismatase | - | NP_416381 | yecD | predicted hydrolase | GCO | 80.85% | 55.90% |
| NP_460860 | yecE | putative cytoplasmic protein | - | NP_416382 | yecE | hypothetical protein | GCO | 82.72% | 56.16% |
| NP_460861 | yecN | putative inner membrane protein | - | NP_416383 | yecN | predicted inner membrane protein | GCO | 93.84% | 52.77% |
| NP_460862 | yecO | putative SAM-dependent methyltransferase | - | NP_416384 | yecO | predicted methyltransferase | GCO | 93.92% | 53.36% |
| NP_460863 | yecP | putative enzyme | - | NP_416385 | yecP | predicted S-adenosyl-L-methionine-dependent methyltransferase | GCO | 92.87% | 55.24% |
| NP_460864 | cutC | copper homeostasis protein | - | YP_025309 | cutC | copper homeostasis protein | GCO | 78.22% | 54.35% |
| NP_460865 | yecM | putative cytoplasmic protein | - | NP_416389 | yecM | predicted metal-binding enzyme | GCO | 84.57% | 53.50% |
| NP_460866 | argS | arginyl-tRNA synthetase | - | NP_416390 | argS | arginyl-tRNA synthetase | GCO | 89.60% | 55.19% |
| NP_460867 | - | putative penicillin-binding protein | - | NP_415168 | mrdA | transpeptidase involved in peptidoglycan synthesis (penicillin-binding protein 2) | nGCO | 57.94% | 51.92% |
| NP_460868 | - | putative cytoplasmic protein | - |  |  |  | NO HOMOLOG |  | 52.63% |
| NP_460869 | flhE | flagellar protein | - | NP_416392 | flhE | hypothetical protein | GCO | 75.38% | 58.77% |
| NP_460870 | flhA | flagellar biosynthesis protein | - | NP_416393 | flhA | flagellar biosynthesis protein A | GCO | 75.28% | 55.98% |
| NP_460871 | flhB | flagellar biosynthesis protein | - | NP_416394 | flhB | flagellar biosynthesis protein B | GCO | 80.05% | 55.90% |
| NP_460872 | cheZ | chemotactic response protein | - | NP_416395 | cheZ | chemotaxis regulator, protein phosphatase for CheY | GCO | 84.77% | 57.36% |
| NP_460873 | cheY | chemotaxis regulator | - | NP_416396 | cheY | chemotaxis regulator transmitting signal to flagellar motor component | GCO | 86.82% | 51.53% |
| NP_460874 | cheB | chemotaxis-specific methylesterase | - | NP_416397 | cheB | chemotaxis-specific methylesterase | GCO | 95.12% | 55.80% |
| NP_460875 | cheR | glutamate methyltransferase | - | NP_416398 | cheR | chemotaxis regulator, protein-glutamate methyltransferase | GCO | 87.41% | 52.71% |
| NP_460876 | cheM | methyl accepting chemotaxis protein II | - | NP_416400 | tar | methyl-accepting chemotaxis protein II | GCO | 68.59% | 55.53% |
| NP_460877 | cheW | chemotaxis docking protein | - | NP_416401 | cheW | purine-binding chemotaxis protein | GCO | 83.23% | 53.17% |
| NP_460878 | cheA | chemotaxis sensory histidine protein kinase | - | NP_416402 | cheA | fused chemotactic sensory histidine kinase in two-component regulatory system with CheB and CheY: sensory histidine kinase/signal sensing protein | GCO | 84.12% | 55.30% |
| NP_460879 | motB | flagellar motor protein | - | NP_416403 | motB | flagellar motor protein MotB | GCO | 86.73% | 54.51% |
| NP_460880 | motA | flagellar motor protein | - | NP_416404 | motA | flagellar motor protein MotA | GCO | 88.47% | 51.23% |
| NP_460881 | flhC | flagellar transcriptional activator | - | NP_416405 | flhC | DNA-binding transcriptional dual regulator with FlhD | GCO | 93.75% | 50.60% |
| NP_460882 | flhD | transcriptional activator FlhD | - | NP_416406 | flhD | transcriptional activator FlhD | GCO | 89.07% | 48.43% |
| NP_460884 | yecG | putative universal stress protein | - | NP_416409 | yecG | universal stress protein | GCO | 67.60% | 52.21% |
| NP_460885 | otsA | trehalose-6-phosphate synthase | - | NP_416410 | otsA | trehalose-6-phosphate synthase | GCO | 85.02% | 52.53% |
| NP_460886 | otsB | trehalose-6-phosphate phophatase | - | NP_416411 | otsB | trehalose-6-phosphate phosphatase, biosynthetic | GCO | 71.53% | 54.10% |
| NP_460887 | araH | putative intracellular protease/amidase | - | NP_414958 | yajL | hypothetical protein | nGCO | 30.55% | 54.65% |
| NP_460888 | ftnB | ferritin-like protein | + | NP_416415 | yecI | predicted ferritin-like protein | nGCO | 76.64% | 49.40% |
| NP_460889 | - | hypothetical protein | - |  |  |  | NO HOMOLOG |  | 48.82% |
| NP_460890 | - | putative outer membrane lipoprotein | - | NP_416417 | yecR | hypothetical protein | GCO | 61.53% | 52.97% |
| NP_460891 | ftn | cytoplasmic ferritin | - | NP_416418 | ftn | ferritin iron storage protein (cytoplasmic) | GCO | 92.12% | 45.98% |
| NP_460892 | yecH | putative cytoplasmic protein | - | NP_416419 | yecH | hypothetical protein | GCO | 87.34% | 51.66% |
| NP_460893 | tyrP | tyrosine-specific transport protein | - | NP_416420 | tyrP | tyrosine transporter | GCO | 77.41% | 55.44% |
| NP_460894 | yecA | putative metal-binding protein | - | NP_416421 | yecA | conserved metal-binding protein | GCO | 81.90% | 53.45% |
| NP_460895 | - | putative glucose-6-phosphate dehydrogenase | - |  |  |  | NO HOMOLOG |  | 41.89% |
| NP_460896 | - | putative cell wall-associated hydrolase | - |  |  |  | NO HOMOLOG |  | 48.47% |
| NP_460897 | - | putative inner membrane protein | + |  |  |  | NO HOMOLOG |  | 36.93% |
| NP_460898 | pgsA | phosphatidylglycerophosphate synthetase | - | NP_416422 | pgsA | phosphatidylglycerophosphate synthetase | GCO | 94.50% | 54.64% |
| NP_460899 | uvrC | excinuclease ABC subunit C | - | NP_416423 | uvrC | excinuclease ABC subunit C | GCO | 95.40% | 53.35% |
| NP_460900 | uvrY | response regulator | - | NP_416424 | uvrY | response regulator | GCO | 96.78% | 51.44% |
| NP_460902 | yecF | putative cytoplasmic protein | - | NP_416425 | yecF | hypothetical protein | GCO | 87.83% | 48.88% |
| NP_460903 | sdiA | transcriptional regulator | - | NP_416426 | sdiA | DNA-binding transcriptional activator | GCO | 62.08% | 49.10% |
| NP_460904 | yecC | putative ABC-type polar amino acid transport system ATPase component | - | NP_416427 | yecC | predicted transporter subunit: ATP-binding component of ABC superfamily | GCO | 88.40% | 54.84% |
| NP_460905 | yecS | putative ABC-type amino acid transporter permease component | - | NP_416428 | yecS | predicted transporter subunit: membrane component of ABC superfamily | GCO | 86.03% | 54.55% |
| NP_460906 | yedO | D-cysteine desulfhydrase | - | NP_416429 | yedO | D-cysteine desulfhydrase | GCO | 82.01% | 57.64% |
| NP_460907 | fliY | putative periplasmic binding transport protein | - | NP_416430 | fliY | cystine transporter subunit | GCO | 86.84% | 51.68% |
| NP_460908 | fliZ | putative FliA-regulator | - | NP_416431 | fliZ | predicted regulator of FliA activity | GCO | 79.78% | 49.45% |
| NP_460909 | fliA | flagellar biosynthesis sigma factor FliA | - | NP_416432 | fliA | flagellar biosynthesis sigma factor | GCO | 93.72% | 54.72% |
| NP_460910 | tnpA_2 | transposase for IS200 | - |  |  |  | NO HOMOLOG |  | 46.07% |
| NP_460911 | fliB | lysine-N-methylase | - |  |  |  | NO HOMOLOG |  | 49.25% |
| NP_460912 | fliC | flagellar biosynthesis protein | - | NP_416433 | fliC | flagellin | GCO | 50.39% | 49.19% |
| NP_460913 | fliD | flagellar hook-associated protein | - | NP_416434 | fliD | flagellar capping protein | GCO | 49.35% | 49.35% |
| NP_460914 | fliS | flagellar protein FliS | - | NP_416435 | fliS | flagellar protein FliS | GCO | 62.90% | 50% |
| NP_460915 | fliT | possible FliD export chaperone | - | NP_416436 | fliT | predicted chaperone | GCO | 31.13% | 50.67% |
| NP_460916 | amyA | cytoplasmic alpha-amylase | - | NP_416437 | amyA | cytoplasmic alpha-amylase | GCO | 88.66% | 51.38% |
| NP_460917 | yedD | putative outer membrane lipoprotein | - | NP_416438 | yedD | hypothetical protein | GCO | 83.72% | 58.92% |
| NP_460918 | yedE | hypothetical protein | - | NP_416439 | yedE | predicted inner membrane protein | GCO | 79.44% | 56.87% |
| NP_460919 | yedF | putative transcriptional regulator | - | NP_416440 | yedF | hypothetical protein | GCO | 100% | 49.14% |
| NP_460921 | fliE | flagellar basal body protein | - | NP_416447 | fliE | flagellar hook-basal body protein FliE | GCO | 86.53% | 56.82% |
| NP_460922 | fliF | flagellar M-ring protein | - | NP_416448 | fliF | flagellar M-ring protein | GCO | 74.59% | 57.15% |
| NP_460923 | fliG | flagellar motor protein | - | NP_416449 | fliG | flagellar motor switch protein G | GCO | 93.35% | 55.32% |
| NP_460924 | fliH | flagellar assembly protein | - | NP_416450 | fliH | flagellar assembly protein H | GCO | 64.68% | 60.45% |
| NP_460925 | fliI | flagellum-specific ATP synthase | - | NP_416451 | fliI | flagellum-specific ATP synthase | GCO | 91.00% | 60.24% |
| NP_460926 | fliJ | flagellar protein | - | NP_416452 | fliJ | flagellar biosynthesis chaperone | GCO | 88.43% | 51.35% |
| NP_460927 | fliK | flagellar hook-length control protein | - | NP_416453 | fliK | flagellar hook-length control protein | GCO | 48.57% | 61.49% |
| NP_460928 | fliL | flagellar biosynthesis protein | - | NP_416454 | fliL | flagellar basal body-associated protein FliL | GCO | 72.25% | 54.70% |
| NP_460929 | fliM | flagellar motor switch protein | - | NP_416455 | fliM | flagellar motor switch protein M | GCO | 95.50% | 53.43% |
| NP_460930 | fliN | flagellar motor switch protein | - | NP_416456 | fliN | flagellar motor switch protein | GCO | 89.78% | 54.83% |
| NP_460931 | fliO | flagellar biosynthetic protein | - | NP_416457 | fliO | flagellar biosynthesis protein | GCO | 55.64% | 60.58% |
| NP_460932 | fliP | flagellar biosynthesis protein | - | NP_416458 | fliP | flagellar biosynthesis protein P | GCO | 89.38% | 53.65% |
| NP_460933 | fliQ | flagellar biosynthesis protein | - | NP_416459 | fliQ | flagellar biosynthesis protein Q | GCO | 69.66% | 51.85% |
| NP_460934 | fliR | flagellar biosynthesis protein | - | NP_416460 | fliR | flagellar biosynthesis protein R | GCO | 81.15% | 51.57% |
| NP_460935 | rcsA | colanic acid capsular biosynthesis activation protein A | + | NP_416461 | rcsA | DNA-binding transcriptional activator, co-regulator with RcsB | GCO | 87.92% | 38.78% |
| NP_951051 | dsrB | hypothetical protein | - | NP_416462 | dsrB | hypothetical protein | GCO | 98.38% | 53.33% |
| NP_460936 | yodD | putative cytoplasmic protein | - | NP_416463 | yodD | hypothetical protein | GCO | 92% | 47.80% |
| NP_460937 | yedP | hypothetical protein | - | NP_416464 | yedP | hypothetical protein | GCO | 71.37% | 55.51% |
| NP_460938 | - | putative inner membrane protein | - | NP_416465 | yedQ | predicted diguanylate cyclase | GCO | 64.94% | 53.35% |
| NP_460939 | - | putative cytoplasmic protein | - | NP_416466 | yodC | hypothetical protein | GCO | 83.05% | 54.33% |
| NP_460940 | yedI | putative inner membrane protein | - | NP_416467 | yedI | conserved inner membrane protein | GCO | 80.52% | 53.72% |
| NP_460941 | yedA | putative permease | - | NP_416468 | yedA | predicted inner membrane protein | GCO | 73.15% | 56.02% |
| NP_460942 | vsr | DNA mismatch endonuclease | - | NP_416469 | vsr | DNA mismatch endonuclease of very short patch repair | GCO | 86.53% | 56.05% |
| NP_460943 | dcm | DNA cytosine methylase | - | NP_416470 | dcm | DNA cytosine methylase | GCO | 83.22% | 53.73% |
| NP_460944 | yedJ | putative hydrolase | - | NP_416471 | yedJ | predicted phosphohydrolase | GCO | 73.36% | 54.88% |
| NP_460945 | - | putative inner membrane protein | - | NP_416472 | yedR | predicted inner membrane protein | GCO | 56.81% | 55.66% |
| NP_460946 | ompS | putative porin | - | NP_415895 | ompN | outer membrane pore protein N, non-specific | nGCO | 66.84% | 50.37% |
| NP_460947 | cspB | putative cold-shock protein | - | NP_416070 | cspI | Qin prophage; cold shock protein | nGCO | 84.28% | 41.78% |
| NP_460948 | umuC | DNA polymerase IV | - | NP_415702 | umuC | DNA polymerase V subunit UmuC | GCO | 83.64% | 54.84% |
| NP_460949 | umuD | SOS-response transcriptional repressors | - | NP_415701 | umuD | DNA polymerase V, subunit D | GCO | 73.18% | 50.23% |
| NP_460950 | - | putative cytoplasmic protein | - |  |  |  | NO HOMOLOG |  | 51.23% |
| NP_460951 | yeeI | putative inner membrane protein | - | NP_416484 | yeeI | hypothetical protein | nGCO | 85.20% | 51.12% |
| NP_460952 | - | putative endoprotease | - |  |  |  | NO HOMOLOG |  | 58.01% |
| NP_460954 | - | tetratricopeptide repeat protein | + |  |  |  | NO HOMOLOG |  | 43.53% |
| NP_460955 | - | putative periplasmic protein | - |  |  |  | NO HOMOLOG |  | 46.44% |
| NP_460956 | amn | AMP nucleosidase | - | NP_416489 | amn | AMP nucleosidase | nGCO | 88.42% | 54.15% |
| NP_460958 | - | putative cytoplasmic protein | - |  |  |  | NO HOMOLOG |  | 40.97% |
| NP_951052 | - | hypothetical protein | - |  |  |  | NO HOMOLOG |  | 45.83% |
| NP_460959 | yeeO | putative transport protein | - | NP_416491 | yeeO | predicted multidrug efflux system | nGCO | 69.23% | 53.49% |
| NP_460960 | erfK | putative periplasmic protein | - | NP_416494 | erfK | conserved protein with NAD(P)-binding Rossmann-fold domain | GCO | 79.67% | 52.47% |
| NP_460961 | cobT | nicotinate-nucleotide--dimethylbenzimidazole phosphoribosyltransferase | - | NP_416495 | cobT | nicotinate-nucleotide--dimethylbenzimidazole phosphoribosyltransferase | GCO | 78.37% | 59.85% |
| NP_460962 | cobS | cobalamin synthase | - | NP_416496 | cobS | cobalamin synthase | GCO | 70.25% | 56.45% |
| NP_460963 | cobU | adenosylcobinamide kinase | - | NP_416497 | cobU | adenosylcobinamide kinase/adenosylcobinamide-phosphate guanylyltransferase | GCO | 82.32% | 54.94% |
| NP_460964 | cbiP | cobyric acid synthase | - |  |  |  | NO HOMOLOG |  | 56.01% |
| NP_460965 | cbiO | vitamin B12 biosynthetic protein | + | NP_414796 | afuC | CP4-6 prophage; predicted ferric transporter subunit | nGCO | 31.33% | 52.32% |
| NP_460966 | cbiQ | vitamin B12 biosynthetic protein | - |  |  |  | NO HOMOLOG |  | 54.27% |
| NP_460967 | cbiN | cobalt transport protein CbiN | - |  |  |  | NO HOMOLOG |  | 49.64% |
| NP_460968 | cbiM | cobalt transport protein CbiM | + |  |  |  | NO HOMOLOG |  | 54.47% |
| NP_460969 | cbiL | precorrin-2 methyltransferase | + |  |  |  | NO HOMOLOG |  | 57.84% |
| NP_460970 | cbiK | vitamin B12 biosynthetic protein | + |  |  |  | NO HOMOLOG |  | 55.72% |
| NP_460971 | cbiJ | precorrin-6x reductase | - |  |  |  | NO HOMOLOG |  | 59.46% |
| NP_460972 | cbiH | precorrin-3B C17-methyltransferase | + |  |  |  | NO HOMOLOG |  | 56.88% |
| NP_460973 | cbiG | cobalamin biosynthesis protein CbiG | + |  |  |  | NO HOMOLOG |  | 55.68% |
| NP_460974 | cbiF | vitamin B12 biosynthetic protein | - | NP_417827 | cysG | fused siroheme synthase 1,3-dimethyluroporphyriongen III dehydrogenase and siroheme ferrochelatase/uroporphyrinogen methyltransferase | nGCO | 29.91% | 57.23% |
| NP_460975 | cbiT | precorrin-8w decarboxylase | - |  |  |  | NO HOMOLOG |  | 55.95% |
| NP_460976 | cbiE | precorrin-6B methylase | - |  |  |  | NO HOMOLOG |  | 57.75% |
| NP_460977 | cbiD | cobalt-precorrin-6A synthase | - |  |  |  | NO HOMOLOG |  | 55.70% |
| NP_460978 | cbiC | precorrin-8X methylmutase | - |  |  |  | NO HOMOLOG |  | 55.45% |
| NP_460979 | cobD | cobalamin biosynthesis protein | - |  |  |  | NO HOMOLOG |  | 59.06% |
| NP_460980 | cbiA | cobyrinic acid a,c-diamide synthase | - |  |  |  | NO HOMOLOG |  | 55.79% |
| NP_460981 | pocR | transcriptional regulator | - | NP_418341 | rhaS | DNA-binding transcriptional activator, L-rhamnose-binding | nGCO | 26.19% | 45.94% |
| NP_460982 | pduF | propanediol diffusion facilitator | - | NP_418362 | glpF | glycerol facilitator | nGCO | 62.54% | 50.94% |
| NP_460983 | pduA | polyhedral body protein | - | NP_416952 | cchA | predicted carboxysome structural protein, ethanolamine utilization protein | nGCO | 47.19% | 53.68% |
| NP_460984 | pudB | polyhedral body protein | - | NP_416934 | eutL | predicted carboxysome structural protein with predicted role in ethanolamine utilization | nGCO | 29.77% | 60.39% |
| NP_460985 | pduC | propanediol dehydratase large subunit | - |  |  |  | NO HOMOLOG |  | 56.81% |
| NP_460986 | pduD | propanediol dehydratase medium subunit | - |  |  |  | NO HOMOLOG |  | 57.03% |
| NP_460987 | pduE | propanediol dehydratase small subunit | - |  |  |  | NO HOMOLOG |  | 56.13% |
| NP_460988 | pduG | propanediol dehydratase reactivation protein | - |  |  |  | NO HOMOLOG |  | 59.68% |
| NP_460989 | pduH | propanediol dehydratase reactivation protein | - |  |  |  | NO HOMOLOG |  | 58.11% |
| NP_460990 | pduJ | polyhedral body protein | - | NP_416952 | cchA | predicted carboxysome structural protein, ethanolamine utilization protein | nGCO | 44.70% | 56.15% |
| NP_460991 | pduK | polyhedral body protein | - |  |  |  | NO HOMOLOG |  | 55.90% |
| NP_460992 | pduL | propanediol utilization protein | - |  |  |  | NO HOMOLOG |  | 60.18% |
| NP_460993 | pduM | propanediol utilization protein | - |  |  |  | NO HOMOLOG |  | 61.17% |
| NP_460994 | pduN | polyhedral body protein | - |  |  |  | NO HOMOLOG |  | 61.23% |
| NP_460995 | pduO | propanediol utilization protein | - |  |  |  | NO HOMOLOG |  | 61.42% |
| NP_460996 | pduP | CoA-dependent propionaldehyde dehydrogenase | - | NP_416950 | eutE | predicted aldehyde dehydrogenase, ethanolamine utilization protein | nGCO | 41.06% | 58.63% |
| NP_460997 | pduQ | propanol dehydrogenase | - | NP_416948 | eutG | predicted alcohol dehydrogenase in ethanolamine utilization | nGCO | 37.36% | 60.37% |
| NP_460998 | pduS | polyhedral body protein | - |  |  |  | NO HOMOLOG |  | 61.43% |
| NP_460999 | pduT | polyhedral body protein | - |  |  |  | NO HOMOLOG |  | 58.37% |
| NP_461000 | pduU | polyhedral body protein | - | NP_416957 | ypfE | predicted carboxysome structural protein with predicted role in ethanol utilization | GCO | 56.88% | 55.55% |
| NP_461001 | pduV | propanediol utilization protein | - | NP_416956 | eutP | conserved protein with nucleoside triphosphate hydrolase domain | GCO | 28.27% | 57.61% |
| NP_461002 | pduW | acetate/propionate kinase | - | NP_417585 | tdcD | propionate kinase/acetate kinase C, anaerobic | nGCO | 41.70% | 52.75% |
| NP_461003 | pduX | propanediol utilization protein | - |  |  |  | NO HOMOLOG |  | 55.81% |
| NP_461004 | yeeX | hypothetical protein | - | NP_416511 | yeeX | hypothetical protein | GCO | 93.57% | 46.72% |
| NP_461005 | yeeA | putative inner membrane protein | - | NP_416512 | yeeA | conserved inner membrane protein | GCO | 74.14% | 53.54% |
| NP_461006 | sbmC | DNA gyrase inhibitor | - | NP_416513 | sbmC | DNA gyrase inhibitor | GCO | 70.96% | 50.85% |
| NP_461007 | dacD | DD-carboxypeptidase | - | NP_416514 | dacD | D-alanyl-D-alanine carboxypeptidase (penicillin-binding protein 6b) | GCO | 87.37% | 52.85% |
| NP_461008 | phsC | thiosulfate reductase cytochrome B subunit | - | NP_416185 | ydhU | predicted cytochrome | GCO | 49.79% | 51.76% |
| NP_461009 | phsB | thiosulfate reductase electron transport protein | - | NP_416186 | ydhX | predicted 4Fe-4S ferridoxin-type protein | GCO | 54.69% | 54.23% |
| NP_461010 | phsA | thiosulfate reductase precursor | - | NP_416104 | ynfE | oxidoreductase subunit | nGCO | 25.29% | 55.46% |
| NP_461011 | sopA | secreted effector protein | + |  |  |  | NO HOMOLOG |  | 47.42% |
| NP_461012 | sbcB | exonuclease I | - | NP_416515 | sbcB | exonuclease I | nGCO | 93.16% | 54.43% |
| NP_461013 | yeeF | putative amino acid transport protein | - | NP_416518 | yeeF | predicted amino-acid transporter | GCO | 90.26% | 53.62% |
| NP_461014 | yeeY | putative transcriptional regulator | - | NP_416519 | yeeY | predicted DNA-binding transcriptional regulator | GCO | 87.08% | 52.56% |
| NP_461015 | yeeZ | putative dehydratase | - | NP_416520 | yeeZ | predicted epimerase, with NAD(P)-binding Rossmann-fold domain | GCO | 83.94% | 57.09% |
| NP_461016 | hisG | ATP phosphoribosyltransferase | - | NP_416523 | hisG | ATP phosphoribosyltransferase | GCO | 90.63% | 55.33% |
| NP_461017 | hisD | histidinol dehydrogenase | - | NP_416524 | hisD | histidinol dehydrogenase | GCO | 92.16% | 58.54% |
| NP_461018 | hisC | histidinol-phosphate aminotransferase | - | NP_416525 | hisC | histidinol-phosphate aminotransferase | GCO | 89.01% | 55.83% |
| NP_461019 | hisB | imidazole glycerol-phosphate dehydratase/histidinol phosphatase | - | NP_416526 | hisB | imidazole glycerol-phosphate dehydratase/histidinol phosphatase | GCO | 92.67% | 52.15% |
| NP_461020 | hisH | imidazole glycerol phosphate synthase subunit HisH | - | NP_416527 | hisH | imidazole glycerol phosphate synthase subunit HisH | GCO | 90.81% | 57.07% |
| NP_461021 | hisA | 1-(5-phosphoribosyl)-5-[(5-phosphoribosylamino)methylideneamino] imidazole-4-carboxamide isomerase | - | NP_416528 | hisA | 1-(5-phosphoribosyl)-5-[(5-phosphoribosylamino)methylideneamino] imidazole-4-carboxamide isomerase | GCO | 83.60% | 58.40% |
| NP_461022 | hisF | imidazole glycerol phosphate synthase subunit HisF | - | NP_416529 | hisF | imidazole glycerol phosphate synthase subunit HisF | GCO | 94.57% | 55.34% |
| NP_461023 | hisI | bifunctional phosphoribosyl-AMP cyclohydrolase/phosphoribosyl-ATP pyrophosphatase protein | - | NP_416530 | hisI | bifunctional phosphoribosyl-AMP cyclohydrolase/phosphoribosyl-ATP pyrophosphatase protein | GCO | 85.71% | 53.10% |
| NP_461024 | wzzB | lipopolysaccharide O-antigen chain length regulator | - | NP_416531 | cld | regulator of length of O-antigen component of lipopolysaccharide chains | GCO | 62.26% | 50.40% |
| NP_461025 | udg | UDP-glucose/GDP-mannose dehydrogenase | + | NP_416532 | ugd | UDP-glucose 6-dehydrogenase | GCO | 88.91% | 44.04% |
| NP_461026 | gnd | 6-phosphogluconate dehydrogenase | - | NP_416533 | gnd | 6-phosphogluconate dehydrogenase | GCO | 96.58% | 52.16% |
| NP_461027 | rfbP | undecaprenol-phosphate galactosephosphotransferase/O-antigen transferase | - | NP_416551 | wcaJ | predicted UDP-glucose lipid carrier transferase | nGCO | 26.56% | 37.10% |
| NP_461028 | rfbK | phosphomannomutase | - | NP_417643 | glmM | phosphoglucosamine mutase | nGCO | 31.51% | 40.86% |
| NP_461029 | rfbM | mannose-1-phosphate guanylyltransferase | - | NP_416553 | cpsB | mannose-1-phosphate guanyltransferase | nGCO | 57.35% | 40.27% |
| NP_461030 | rfbN | rhamnosyl transferase | - |  |  |  | NO HOMOLOG |  | 37.46% |
| NP_461031 | rfbU | mannosyl transferase | - |  |  |  | NO HOMOLOG |  | 33.52% |
| NP_461032 | rfbV | abequosyltransferase | - |  |  |  | NO HOMOLOG |  | 32.33% |
| NP_461033 | rfbX | putative O-antigen transferase | - |  |  |  | NO HOMOLOG |  | 31.01% |
| NP_461034 | rfbJ | CDP-abequose synthase | - |  |  |  | NO HOMOLOG |  | 32% |
| NP_461035 | rfbH | CDP-6-deoxy-D-xylo-4-hexulose-3-dehydrase | - | NP_416756 | yfbE | uridine 5'-(beta-1-threo-pentapyranosyl-4-ulose diphosphate) aminotransferase, PLP-dependent | nGCO | 27.62% | 44.52% |
| NP_461036 | rfbG | CDP glucose 4,6-dehydratase | - | NP_416557 | gmd | GDP-D-mannose dehydratase, NAD(P)-binding | nGCO | 23.80% | 43.61% |
| NP_461037 | rfbF | glucose-1-phosphate cytidylyltransferase | - |  |  |  | NO HOMOLOG |  | 43.54% |
| NP_461038 | rfbI | CDP-6-deoxy-delta-3,4-glucoseen reductase | - | NP_418286 | fre | NAD(P)H-flavin reductase | nGCO | 29.07% | 40.48% |
| NP_461039 | rfbC | dTDP-4,deoxyrhamnose 3,5 epimerase | - | NP_416542 | rfbC | dTDP-4-deoxyrhamnose-3,5-epimerase | GCO | 70.25% | 40.21% |
| NP_461040 | rfbA | dTDP-glucose pyrophosphorylase | - | NP_416543 | rfbA | glucose-1-phosphate thymidylyltransferase | GCO | 92.75% | 45.62% |
| NP_461041 | rfbD | TDP-rhamnose synthetase | - | NP_416544 | rfbD | dTDP-4-dehydrorhamnose reductase subunit, NAD(P)-binding, of dTDP-L-rhamnose synthase | GCO | 84.94% | 50.33% |
| NP_461042 | rfbB | dTDP-glucose 4,6-dehydratase | - | NP_416545 | rfbB | dTDP-glucose 4,6 dehydratase, NAD(P)-binding | GCO | 84.21% | 44.01% |
| NP_461043 | galF | putative glucose-1-phosphate uridylyltransferase subunit | - | NP_416546 | galF | predicted subunit with GalU | GCO | 92.25% | 51.78% |
| NP_461044 | wcaM | putative colanic acid biosynthetic protein | - | NP_416547 | wcaM | predicted colanic acid biosynthesis protein | GCO | 77.03% | 51.21% |
| NP_461045 | wcaL | putative glycosyl transferase | - | NP_416548 | wcaL | predicted glycosyl transferase | GCO | 85.46% | 56.18% |
| NP_461046 | wcaK | putative galactokinase | + | NP_416549 | wcaK | predicted pyruvyl transferase | GCO | 67.84% | 53.23% |
| NP_461047 | wzxC | putative export | - | NP_416550 | wzxC | colanic acid exporter | GCO | 83.33% | 51.72% |
| NP_461048 | wcaJ | UDP-glucose lipid carrier transferase/glucose-1-phosphate transferase | - | NP_416551 | wcaJ | predicted UDP-glucose lipid carrier transferase | GCO | 83.40% | 53.18% |
| NP_461049 | cpsG | phosphomannomutase | - | NP_416552 | cpsG | phosphomannomutase | GCO | 87.93% | 61.70% |
| NP_461050 | manC | mannose-1-phosphate guanylyltransferase | + | NP_416553 | cpsB | mannose-1-phosphate guanyltransferase | GCO | 85.56% | 60.61% |
| NP_461051 | wcaI | putative glycosyl transferase | + | NP_416554 | wcaI | predicted glycosyl transferase | GCO | 85.99% | 57.76% |
| NP_461052 | wcaH | GDP-mannose mannosyl hydrolase | + | NP_416555 | nudD | GDP-mannose mannosyl hydrolase | GCO | 78.51% | 59.92% |
| NP_461053 | wcaG | GDP-fucose synthetase | + | NP_416556 | fcl | bifunctional GDP-fucose synthetase: GDP-4-dehydro-6-deoxy-D-mannose epimerase/ GDP-4-dehydro-6-L-deoxygalactose reductase | GCO | 90.65% | 59.83% |
| NP_461054 | gmd | GDP-D-mannose dehydratase | + | NP_416557 | gmd | GDP-D-mannose dehydratase, NAD(P)-binding | GCO | 96.24% | 56.59% |
| NP_461055 | wcaF | putative acyltransferase | + | NP_416558 | wcaF | predicted acyl transferase | GCO | 87.22% | 50.81% |
| NP_461056 | wcaE | putative transferase | + | NP_416559 | wcaE | predicted glycosyl transferase | GCO | 71.77% | 48.72% |
| NP_461057 | wcaD | putative colanic acid polymerase | + | NP_416560 | wcaD | predicted colanic acid polymerase | GCO | 84.40% | 43.29% |
| NP_461058 | wcaC | putative glycosyl transferase | - | NP_416561 | wcaC | predicted glycosyl transferase | GCO | 79.01% | 55.50% |
| NP_461059 | wcaB | putative acyl transferase | - | NP_416562 | wcaB | predicted acyl transferase | GCO | 91.25% | 56.03% |
| NP_461060 | wcaA | putative glycosyl transferase | + | NP_416563 | wcaA | predicted glycosyl transferase | GCO | 91.33% | 50.53% |
| NP_461061 | wzc | putative tyrosine protein kinase | - | NP_416564 | wzc | protein-tyrosine kinase | GCO | 85.34% | 54.12% |
| NP_461062 | wzb | putative phosphotyrosine-protein phosphatase | - | NP_416565 | wzb | protein-tyrosine phosphatase | GCO | 78.76% | 57.11% |
| NP_461063 | wza | putative outer membrane polysaccharide export protein | - | NP_416566 | wza | lipoprotein required for capsular polysaccharide translocation through the outer membrane | GCO | 94.44% | 53.59% |
| NP_461064 | yegH | putative inner membrane protein | - | NP_416567 | yegH | fused predicted membrane protein/predicted membrane protein | GCO | 83.30% | 54.14% |
| NP_461065 | asmA | suppressor of OmpF assembly mutants | - | NP_416568 | asmA | predicted assembly protein | GCO | 71.84% | 55.46% |
| NP_461066 | dcd | deoxycytidine triphosphate deaminase | - | NP_416569 | dcd | deoxycytidine triphosphate deaminase | GCO | 95.85% | 59.79% |
| NP_461067 | udk | uridine kinase | - | NP_416570 | udk | uridine kinase | GCO | 95.77% | 46.88% |
| NP_461068 | yegE | putative diguanylate cyclase/phosphodiesterase | - | NP_416571 | yegE | predicted diguanylate cyclase, GGDEF domain signalling protein | GCO | 68.93% | 53.92% |
| NP_461069 | alkA | 3-methyl-adenine DNA glycosylase II | - | NP_416572 | alkA | 3-methyl-adenine DNA glycosylase II | GCO | 71.32% | 61.49% |
| NP_461070 | yegD | putative heat shock protein | - | NP_416573 | yegD | predicted chaperone | GCO | 88.44% | 59.71% |
| NP_461071 | - | putative secretion protein | - | NP_416578 | mdtA | multidrug efflux system, subunit A | GCO | 76.38% | 58.05% |
| NP_461072 | yegN | putative outer membrane receptor | - | NP_416579 | mdtB | multidrug efflux system, subunit B | GCO | 86.05% | 58.18% |
| NP_461073 | yegO | outer membrane putative efflux transporter | - | NP_416580 | mdtC | multidrug efflux system, subunit C | GCO | 84.50% | 56.50% |
| NP_461074 | yegB | putative transport protein | - | NP_416581 | mdtD | multidrug efflux system protein | GCO | 72.92% | 54.49% |
| NP_461075 | baeS | sensor kinase | - | NP_416582 | baeS | sensory histidine kinase in two-component regulatory system with BaeR | GCO | 84.58% | 56.62% |
| NP_461076 | baeR | response regulator | - | NP_416583 | baeR | DNA-binding response regulator in two-component regulatory system with BaeS | GCO | 90.41% | 53.80% |
| NP_461077 | - | putative cytoplasmic protein | - |  |  |  | NO HOMOLOG |  | 50.05% |
| NP_461078 | - | putative inner membrane protein | - |  |  |  | NO HOMOLOG |  | 57.32% |
| NP_461079 | - | putative inner membrane protein | - |  |  |  | NO HOMOLOG |  | 53.40% |
| NP_461080 | yegQ | putative protease | - | NP_416585 | yegQ | predicted peptidase | nGCO | 92.93% | 54.47% |
| NP_461081 | - | putative cytoplasmic protein | + |  |  |  | NO HOMOLOG |  | 37.15% |
| NP_461082 | - | putative cytoplasmic protein | + |  |  |  | NO HOMOLOG |  | 32.37% |
| NP_461083 | - | putative inner membrane protein | + |  |  |  | NO HOMOLOG |  | 50.59% |
| NP_945164 | - | hypothetical protein | + |  |  |  | NO HOMOLOG |  | 47.00% |
| NP_461084 | yegS | putative diacylglycerol kinase | - | NP_416590 | yegS | hypothetical protein | nGCO | 77.27% | 54.88% |
| NP_461085 | fbaB | fructose-bisphosphate aldolase | - | NP_416600 | fbaB | fructose-bisphosphate aldolase | GCO | 93.14% | 52.99% |
| NP_461086 | yegT | putative transport protein | - | NP_416601 | yegT | predicted nucleoside transporter | GCO | 89.41% | 51.65% |
| NP_461087 | yegU | putative glycohydrolase | - | NP_416602 | yegU | predicted hydrolase | GCO | 72.15% | 58.50% |
| NP_461088 | yegV | putative sugar kinase | - | NP_416603 | yegV | predicted kinase | GCO | 77.77% | 60.97% |
| NP_461089 | yegW | putative regulatory protein | - | NP_416604 | yegW | predicted DNA-binding transcriptional regulator | GCO | 92.33% | 53.01% |
| NP_461090 | thiD | phosphomethylpyrimidine kinase | - | NP_416606 | thiD | phosphomethylpyrimidine kinase | GCO | 86.84% | 60.54% |
| NP_461091 | thiM | hydroxyethylthiazole kinase | - | NP_416607 | thiM | hydroxyethylthiazole kinase | GCO | 63.70% | 61.02% |
| NP_461092 | - | putative periplasmic protein | - | NP_416610 | yohN | hypothetical protein | GCO | 80.90% | 47.61% |
| NP_461093 | stcD | putative outer membrane lipoprotein | + | NP_416611 | yehA | predicted fimbrial-like adhesin protein | GCO | 50.29% | 41.26% |
| NP_461094 | stcC | putative outer membrane protein | + | NP_416612 | yehB | predicted outer membrane protein | GCO | 72.13% | 45.78% |
| NP_461095 | stcB | putative periplasmic chaperone protein | + | NP_416613 | yehC | predicted periplasmic pilin chaperone | GCO | 66.19% | 40.20% |
| NP_461096 | stcA | putative fimbrial-like protein | + | NP_416614 | yehD | predicted fimbrial-like adhesin protein | GCO | 31.97% | 42.56% |
| NP_461097 | yehE | putative outer membrane protein | + | NP_416615 | yehE | hypothetical protein | GCO | 46.23% | 48.22% |
| NP_461098 | mrp | putative ATP-binding protein | - | NP_416616 | mrp | antiporter inner membrane protein | GCO | 92.95% | 56.12% |
| NP_461099 | metG | methionyl-tRNA synthetase | - | NP_416617 | metG | methionyl-tRNA synthetase | GCO | 93.50% | 54.76% |
| NP_461100 | yehR | putative lipoprotein | - | NP_416627 | yehR | hypothetical protein | nGCO | 73.48% | 43.57% |
| NP_461101 | - | hypothetical protein | - | NP_416627 | yehR | hypothetical protein | GCO | 30.21% | 40.67% |
| NP_461102 | yehS | putative cytoplasmic protein | - | NP_416628 | yehS | hypothetical protein | GCO | 90.32% | 50.64% |
| NP_461103 | yehT | putative regulatory protein | - | NP_416629 | yehT | predicted response regulator in two-component system withYehU | GCO | 87.86% | 50.69% |
| NP_461104 | yehU | putative sensor kinase | - | NP_416630 | yehU | predicted sensory kinase in two-component system with YehT | GCO | 87.70% | 52.90% |
| NP_461105 | yehV | putative transcriptional repressor | - | NP_416631 | mlrA | DNA-binding transcriptional regulator | GCO | 70.78% | 54.37% |
| NP_461106 | - | putative inner membrane protein | - | YP_588460 | yohO | hypothetical protein | GCO | 88.57% | 54.62% |
| NP_461107 | yehW | putative ABC-type proline/glycine betaine transport system permease component | - | NP_416632 | yehW | predicted transporter subunit: membrane component of ABC superfamily | GCO | 64.19% | 59.56% |
| NP_461108 | yehX | putative ABC-type proline/glycine betaine transport system ATPase component | - | NP_416633 | yehX | predicted transporter subunit: ATP-binding component of ABC superfamily | GCO | 87.62% | 58.01% |
| NP_461109 | yehY | putative ABC-type proline/glycine betaine transport system permease component | - | NP_416634 | yehY | predicted transporter subunit: membrane component of ABC superfamily | GCO | 64.08% | 59.65% |
| NP_461110 | yehZ | putative transport protein | - | NP_416635 | yehZ | predicted transporter subunit: periplasmic-binding component of ABC superfamily | GCO | 90.14% | 54.90% |
| NP_461111 | bglX | periplasmic beta-D-glucoside glucohydrolase | - | NP_416636 | bglX | beta-D-glucoside glucohydrolase, periplasmic | GCO | 91.24% | 54.52% |
| NP_461112 | dld | D-lactate dehydrogenase | - | NP_416637 | dld | D-lactate dehydrogenase, FAD-binding, NADH independent | GCO | 90.50% | 53.89% |
| NP_461113 | pbpG | D-alanyl-D-alanine endopeptidase | - | NP_416638 | pbpG | D-alanyl-D-alanine endopeptidase | GCO | 89.65% | 53.90% |
| NP_461114 | yohC | putative transport protein | - | NP_416639 | yohC | predicted inner membrane protein | GCO | 91.79% | 54.76% |
| NP_461115 | yohD | hypothetical protein | - | NP_416640 | yohD | conserved inner membrane protein | GCO | 76.21% | 57.95% |
| NP_461116 | yohF | oxidoreductase | - | NP_416641 | yohF | predicted oxidoreductase with NAD(P)-binding Rossmann-fold domain | GCO | 80.07% | 54.72% |
| NP_461117 | yohG | putative outer membrane efflux protein | - | NP_416643 | yohG | predicted outer membrane protein | GCO | 80.55% | 57.68% |
| NP_461119 | yohI | putative regulatory protein | - | NP_416645 | dusC | tRNA-dihydrouridine synthase C | nGCO | 89.74% | 57.29% |
| NP_461120 | - | salicylate hydroxylase | - |  |  |  | NO HOMOLOG |  | 58.87% |
| NP_461121 | - | putative glutathione S-transferase | - | NP_417696 | sspA | stringent starvation protein A | nGCO | 25.94% | 56.27% |
| NP_461122 | - | putative flutathione S-transferase | - | NP_415698 | ycgM | predicted isomerase/hydrolase | nGCO | 37.43% | 54.55% |
| NP_461123 | - | putative 1,2-dioxygenase | - |  |  |  | NO HOMOLOG |  | 56.74% |
| NP_461124 | - | putative sugar transporter | - | NP_414887 | mhpT | predicted 3-hydroxyphenylpropionic transporter | nGCO | 29.42% | 58.79% |
| NP_461125 | - | putative transcriptional regulator | - | NP_415939 | ydcI | predicted DNA-binding transcriptional regulator | nGCO | 26.31% | 54.89% |
| NP_461126 | yohJ | hypothetical protein | - | NP_416646 | yohJ | hypothetical protein | GCO | 82.57% | 46.36% |
| NP_461127 | yohK | hypothetical protein | - | NP_416647 | yohK | predicted inner membrane protein | GCO | 80% | 56.03% |
| NP_461128 | cdd | cytidine deaminase | - | NP_416648 | cdd | cytidine deaminase | GCO | 81.22% | 56.49% |
| NP_461129 | sanA | vancomycin sensitivity | - | NP_416649 | sanA | hypothetical protein | GCO | 84.51% | 51.94% |
| NP_461130 | b2145 | putative inner membrane protein | - | NP_416650 | yeiS | predicted inner membrane protein | GCO | 59.49% | 40.65% |
| NP_461131 | - | putative NADPH-dependent glutamate synthase beta chain | - | NP_416651 | yeiT | predicted oxidoreductase | GCO | 84.01% | 51.04% |
| NP_461132 | yeiA | dihydropyrimidine dehydrogenase | - | NP_416652 | yeiA | dihydropyrimidine dehydrogenase | GCO | 93.67% | 51.13% |
| NP_461133 | mglC | beta-methylgalactoside transporter inner membrane component | - | NP_416653 | mglC | beta-methylgalactoside transporter inner membrane component | GCO | 82.14% | 51.63% |
| NP_461134 | mglA | methyl-galactoside transport protein | - | NP_416654 | mglA | fused methyl-galactoside transporter subunits of ABC superfamily: ATP-binding components | GCO | 93.87% | 46.08% |
| NP_461135 | mglB | galactose transport protein | - | NP_416655 | mglB | methyl-galactoside transporter subunit | GCO | 89.45% | 51.45% |
| NP_461136 | galS | mgl operon transcriptional repressor | - | NP_416656 | galS | DNA-binding transcriptional repressor | GCO | 88.82% | 55.71% |
| NP_461137 | yeiB | putative inner membrane protein | - | NP_416657 | yeiB | conserved inner membrane protein | GCO | 72.94% | 55.11% |
| NP_461138 | folE | GTP cyclohydrolase I | - | NP_416658 | folE | GTP cyclohydrolase I | GCO | 80.54% | 52.61% |
| NP_461139 | yeiG | putative esterase | - | NP_416659 | yeiG | predicted esterase | GCO | 86.72% | 57.85% |
| NP_461140 | - | putative transcriptional regulator | - |  |  |  | NO HOMOLOG |  | 51.53% |
| NP_461141 | - | putative L-serine dehydratase | - | NP_416328 | sdaA | L-serine deaminase I | nGCO | 46.13% | 54.38% |
| NP_461142 | - | putative phosphoserine phosphatase | - | NP_418805 | serB | 3-phosphoserine phosphatase | nGCO | 39.48% | 44.59% |
| NP_461143 | - | putative regulatory protein | + | NP_418123 | uhpC | membrane protein regulates uhpT expression | nGCO | 38.74% | 51.02% |
| NP_461144 | cirA | colicin I receptor protein | - | NP_416660 | cirA | ferric iron-catecholate outer membrane transporter | GCO | 88.08% | 54.51% |
| NP_461145 | lysP | lysine-specific permease | - | NP_416661 | lysP | lysine transporter | GCO | 91.61% | 53.12% |
| NP_461146 | yeiE | putative transcriptional regulator | - | NP_416662 | yeiE | predicted DNA-binding transcriptional regulator | GCO | 85.71% | 54.62% |
| NP_461147 | yeiH | putative inner membrane protein | - | NP_416663 | yeiH | conserved inner membrane protein | GCO | 71.34% | 54.85% |
| NP_461148 | nfo | endonuclease IV | - | NP_416664 | nfo | endonuclease IV | GCO | 84.56% | 53.96% |
| NP_461149 | fruA | fructose-specific transport protein | - | NP_416672 | fruA | fused fructose-specific PTS enzymes: IIBcomponent/IIC components | GCO | 86.14% | 59.20% |
| NP_461150 | fruK | 1-phosphofructokinase | - | NP_416673 | fruK | 1-phosphofructokinase | GCO | 94.23% | 56.65% |
| NP_461151 | fruF | phosphoenolpyruvate-dependent sugar phosphotransferase system component | - | NP_416674 | fruB | fused fructose-specific PTS enzymes: IIA component/HPr component | GCO | 80.85% | 56.58% |
| NP_461152 | setB | proton efflux pump | - | NP_416675 | setB | lactose/glucose efflux system | GCO | 86.25% | 54.23% |
| NP_461153 | - | putative inner membrane protein | + | NP_414844 | ykgH | predicted inner membrane protein | nGCO | 20.18% | 38.43% |
| NP_461154 | - | putative inner membrane protein | - |  |  |  | NO HOMOLOG |  | 37.39% |
| NP_945165 | - | hypothetical protein | - | YP_588461 | yeiW | hypothetical protein | GCO | 63.09% | 50.19% |
| NP_461155 | yeiP | elongation factor P | - | NP_416676 | yeiP | elongation factor P | GCO | 97.89% | 50.95% |
| NP_461156 | yeiR | putative cobalamin biosynthetic protein | - | NP_416678 | yeiR | predicted enzyme | GCO | 81.40% | 55.72% |
| NP_461157 | yeiU | putative permease | - | NP_416679 | yeiU | undecaprenyl pyrophosphate phosphatase | GCO | 78.80% | 50.83% |
| NP_461158 | spr | putative lipoprotein | - | NP_416680 | spr | predicted peptidase, outer membrane lipoprotein | GCO | 92.63% | 49.91% |
| NP_461159 | rtn | hypothetical protein | - | NP_416681 | rtn | hypothetical protein | GCO | 68.72% | 51.89% |
| NP_461160 | yejA | putative ABC transporter periplasmic binding protein | - | NP_416682 | yejA | predicted oligopeptide transporter subunit | GCO | 86.35% | 51.99% |
| NP_461161 | yejB | putative ABC-type dipeptide/oligopeptide/nickel transport system permease | - | NP_416683 | yejB | predicted oligopeptide transporter subunit | GCO | 91.75% | 52.32% |
| NP_461162 | yejE | putative ABC-type dipeptide/oligopeptide/nickel transport system permease component | - | NP_416684 | yejE | predicted oligopeptide transporter subunit | GCO | 85.63% | 55.55% |
| NP_461163 | yejF | putative ABC-type transport system ATPase component | - | NP_416685 | yejF | fused predicted oligopeptide transporter subunits of ABC superfamilly: ATP-binding components | GCO | 86.57% | 56.79% |
| NP_461164 | yejG | putative cytoplasmic protein | - | NP_416686 | yejG | hypothetical protein | GCO | 91.22% | 52.46% |
| NP_461165 | bcr | bicyclomycin resistance protein | - | NP_416687 | bcr | bicyclomycin/multidrug efflux system | GCO | 83.83% | 53.90% |
| NP_461166 | rsuA | 16S rRNA pseudouridylate 516 synthase | - | NP_416688 | rsuA | 16S rRNA pseudouridylate 516 synthase | GCO | 94.78% | 54.31% |
| NP_461167 | yejH | putative ATP-dependent helicase | - | NP_416689 | yejH | predicted ATP-dependet helicase | GCO | 95.39% | 57.01% |
| NP_461168 | rplY | 50S ribosomal protein L25 | - | NP_416690 | rplY | 50S ribosomal protein L25 | GCO | 91.48% | 51.22% |
| NP_461169 | - | putative inner membrane protein | - |  |  |  | NO HOMOLOG |  | 43.63% |
| NP_461170 | yejK | nucleoid-associated protein NdpA | - | NP_416691 | yejK | nucleoid-associated protein NdpA | GCO | 91.01% | 53.57% |
| NP_461171 | yejL | putative cytoplasmic protein | - | NP_416692 | yejL | hypothetical protein | GCO | 70.66% | 52.19% |
| NP_461172 | yejM | putative hydrolase | - | NP_416693 | yejM | predicted hydrolase, inner membrane | GCO | 85.83% | 52.29% |
| NP_461173 | - | DNA polymerase V subunit | - | NP_415701 | umuD | DNA polymerase V, subunit D | nGCO | 39.34% | 51.59% |
| NP_461174 | - | virulence protein | - | NP_415579 | dinI | DNA damage-inducible protein I | nGCO | 39.70% | 47.76% |
| NP_461175 | oafA | O-antigen acetylase | + |  |  |  | NO HOMOLOG |  | 38.46% |
| NP_461176 | - | putative cytoplasmic protein | + | NP_416855 | yfdK | CPS-53 (KpLE1) prophage; conserved protein | nGCO | 46.85% | 45.04% |
| NP_461177 | - | putative phage tail fiber assembly protein | - |  |  |  | NO HOMOLOG |  | 43.93% |
| NP_461178 | - | hypothetical protein | - |  |  |  | NO HOMOLOG |  | 50.48% |
| NP_461179 | - | hypothetical protein | - |  |  |  | NO HOMOLOG |  | 55.10% |
| NP_461180 | - | putative inner membrane protein | - |  |  |  | NO HOMOLOG |  | 52.42% |
| NP_461181 | - | hypothetical protein | - |  |  |  | NO HOMOLOG |  | 33.48% |
| NP_461182 | - | putative phage antiterminator | - | NP_415083 | ybcQ | DLP12 prophage; predicted antitermination protein | nGCO | 47.93% | 44.17% |
| NP_461183 | - | putative cytoplasmic protein | - | NP_416078 | ydfU | Qin prophage; predicted protein | nGCO | 54.44% | 54.34% |
| NP_461184 | sspH2 | leucine-rich repeat protein | + |  |  |  | NO HOMOLOG |  | 55.34% |
| NP_461185 | - | putative phage tail fiber protein | - |  |  |  | NO HOMOLOG |  | 48.52% |
| NP_461186 | - | putative tail fiber protein of phage | - |  |  |  | NO HOMOLOG |  | 53.91% |
| NP_461187 | - | virulence protein | - |  |  |  | NO HOMOLOG |  | 38.72% |
| NP_461188 | - | putative outer membrane protein | + |  |  |  | NO HOMOLOG |  | 39.53% |
| NP_461189 | narP | response regulator | - | NP_416697 | narP | DNA-binding response regulator in two-component regulatory system with NarQ or NarX | GCO | 88.37% | 52.62% |
| NP_461190 | ccmH | putative heme lyase subunit | - | NP_416698 | ccmH | heme lyase, CcmH subunit | GCO | 64.65% | 66.28% |
| NP_461191 | ccmG | heme lyase/disulfide oxidoreductase | - | NP_416699 | ccmG | periplasmic thioredoxin of cytochrome c-type biogenesis | GCO | 79.37% | 62.18% |
| NP_461192 | ccmF | cytochrome c-type biogenesis protein | - | NP_416700 | ccmF | heme lyase, CcmF subunit | GCO | 76.62% | 68.16% |
| NP_461193 | ccmE | periplasmic heme-dependent peroxidase | - | NP_416701 | ccmE | periplasmic heme chaperone | GCO | 74.21% | 65.41% |
| NP_461194 | ccmD | heme exporter protein C | - | NP_416702 | ccmD | cytochrome c biogenesis protein | GCO | 74% | 69.48% |
| NP_461195 | ccmC | heme exporter protein | - | NP_416703 | ccmC | heme exporter subunit | GCO | 86.12% | 65.44% |
| NP_461196 | ccmB | heme exporter protein | - | NP_416704 | ccmB | heme exporter subunit | GCO | 48.40% | 70.15% |
| NP_461197 | ccmA | heme exporter protein | - | NP_416705 | ccmA | heme exporter subunit | GCO | 64.28% | 72.65% |
| NP_461198 | napC | periplasmic nitrate reductase | - | NP_416706 | napC | nitrate reductase, cytochrome c-type, periplasmic | GCO | 80% | 51.57% |
| NP_461199 | napB | periplasmic nitrate reductase | - | NP_416707 | napB | nitrate reductase, small, cytochrome C550 subunit, periplasmic | GCO | 91.94% | 57.55% |
| NP_461200 | napH | quinol dehydrogenase membrane component | - | NP_416708 | napH | quinol dehydrogenase membrane component | GCO | 78.21% | 53.79% |
| NP_461201 | napG | quinol dehydrogenase periplasmic component | - | NP_416709 | napG | quinol dehydrogenase periplasmic component | GCO | 87.44% | 60.77% |
| NP_461202 | napA | periplasmic nitrate reductase | - | NP_416710 | napA | nitrate reductase, periplasmic, large subunit | GCO | 91.06% | 57.13% |
| NP_461203 | napD | periplasmic nitrate reductase | - | NP_416711 | napD | assembly protein for periplasmic nitrate reductase | GCO | 81.60% | 54.16% |
| NP_461204 | napF | electron transfer protein | - | NP_416712 | napF | ferredoxin-type protein, predicted role in electron transfer to periplasmic nitrate reductase (NapA) | GCO | 76.54% | 57.11% |
| NP_461205 | eco | ecotin precursor | - | NP_416713 | eco | ecotin precursor | GCO | 82.97% | 54.94% |
| NP_461206 | yojI | putative ABC-type multidrug/protein/lipid transport system ATPase component | - | NP_416715 | yojI | fused predicted multidrug transport subunits of ABC superfamily: membrane component/ATP-binding component | GCO | 77.69% | 54.86% |
| NP_461207 | alkB | DNA repair system protein | - | NP_416716 | alkB | oxidative demethylase of N1-methyladenine or N3-methylcytosine DNA lesions | GCO | 76.38% | 58.83% |
| NP_461208 | ada | O6-methylguanine-DNA methyltransferase/transcription activator/repressor | - | NP_416717 | ada | fused DNA-binding transcriptional dual regulator/O6-methylguanine-DNA methyltransferase | GCO | 70.25% | 60.73% |
| NP_461209 | apbE | putative lipoprotein | - | NP_416718 | yojL | predicted thiamine biosynthesis lipoprotein | GCO | 79.48% | 56.41% |
| NP_461210 | ompC | outer membrane protein C precursor | - | NP_416719 | ompC | outer membrane porin protein C | GCO | 77.62% | 52.77% |
| NP_461211 | yojN | putative sensor kinase | - | NP_416720 | rcsD | phosphotransfer intermediate protein in two-component regulatory system with RcsBC | GCO | 78.08% | 51.38% |
| NP_461212 | rcsB | response regulator | - | NP_416721 | rcsB | DNA-binding response regulator in two-component regulatory system with RcsC and YojN | GCO | 99.07% | 49.00% |
| NP_461213 | rcsC | sensory histidine kinase | - | NP_416722 | rcsC | hybrid sensory kinase in two-component regulatory system with RcsB and YojN | GCO | 81.09% | 53.42% |
| NP_461214 | gyrA | DNA gyrase subunit A | - | NP_416734 | gyrA | DNA gyrase subunit A | nGCO | 87.84% | 56.04% |
| NP_461215 | - | putative dehydratase | + | YP_026237 | dgoD | galactonate dehydratase | nGCO | 28.23% | 43.22% |
| NP_461216 | - | putative permease | + | NP_416749 | yfaV | predicted transporter | nGCO | 27.92% | 42.00% |
| NP_461217 | - | putative regulatory protein | + | NP_416058 | ydfH | predicted DNA-binding transcriptional regulator | nGCO | 23.61% | 38.50% |
| NP_461218 | ubiG | 3-demethylubiquinone-9 3-methyltransferase | - | NP_416735 | ubiG | 3-demethylubiquinone-9 3-methyltransferase | nGCO | 92.50% | 55.00% |
| NP_461219 | nrdA | ribonucleotide-diphosphate reductase alpha subunit | - | NP_416737 | nrdA | ribonucleotide-diphosphate reductase alpha subunit | GCO | 96.58% | 53.45% |
| NP_461220 | nrdB | ribonucleotide-diphosphate reductase beta subunit | - | NP_416738 | nrdB | ribonucleotide-diphosphate reductase beta subunit | GCO | 98.40% | 51.37% |
| NP_461221 | yfaE | putative ferredoxin | - | NP_416739 | yfaE | predicted 2Fe-2S cluster-containing protein | GCO | 96.42% | 56.07% |
| NP_461222 | - | putative permease | - | NP_417930 | yhhS | predicted transporter | nGCO | 24.11% | 49.45% |
| NP_461223 | - | putative transcriptional regulator | - | NP_415791 | cysB | DNA-binding transcriptional dual regulator, O-acetyl-L-serine-binding | nGCO | 26% | 48.00% |
| NP_461224 | glpQ | periplasmic glycerophosphodiester phosphodiesterase | - | NP_416742 | glpQ | periplasmic glycerophosphodiester phosphodiesterase | GCO | 89.07% | 48.55% |
| NP_461225 | glpT | sn-glycerol-3-phosphate transport protein | - | NP_416743 | glpT | sn-glycerol-3-phosphate transporter | GCO | 86.72% | 54.74% |
| NP_461226 | glpA | sn-glycerol-3-phosphate dehydrogenase large subunit | - | NP_416744 | glpA | sn-glycerol-3-phosphate dehydrogenase (anaerobic), large subunit, FAD/NAD(P)-binding | GCO | 90.40% | 58.62% |
| NP_461227 | glpB | anaerobic glycerol-3-phosphate dehydrogenase subunit B | - | NP_416745 | glpB | anaerobic glycerol-3-phosphate dehydrogenase subunit B | GCO | 72.31% | 59.44% |
| NP_461228 | glpC | sn-glycerol-3-phosphate dehydrogenase K-small subunit | - | NP_416746 | glpC | sn-glycerol-3-phosphate dehydrogenase (anaerobic), small subunit | GCO | 94.68% | 53.90% |
| NP_461229 | - | putative cytoplasmic protein | + | NP_416772 | elaD | predicted enzyme | nGCO | 29.23% | 38.67% |
| NP_461230 | - | putative cytoplasmic protein | - |  |  |  | NO HOMOLOG |  | 52.54% |
| NP_461231 | - | putative 2,4-dihydroxyhept-2-ene-1,7-dioic acid aldolase | - | NP_416748 | yfaU | predicted 2,4-dihydroxyhept-2-ene-1,7-dioic acid aldolase | GCO | 81.27% | 54.60% |
| NP_461232 | yfaV | putative transport protein | - | NP_416749 | yfaV | predicted transporter | GCO | 85.31% | 54.10% |
| NP_461233 | yfaW | putative galactonate dehydratase | - | NP_416750 | yfaW | predicted enolase | GCO | 91.60% | 53.28% |
| NP_461234 | yfaX | putative transcriptional regulator | - | NP_416751 | yfaX | predicted DNA-binding transcriptional regulator | GCO | 87.30% | 51.46% |
| NP_461235 | - | competence damage-inducible protein A | - | NP_416752 | yfaY | competence damage-inducible protein A | GCO | 84.88% | 56.39% |
| NP_461236 | yfaZ | putative inner membrane protein | - | NP_416753 | yfaZ | predicted outer membrane porin protein | GCO | 73.33% | 52.00% |
| NP_461237 | yfaO | putative NTP pyrophosphohydrolase | - | NP_416754 | yfaO | predicted NUDIX hydrolase | GCO | 73.04% | 49.53% |
| NP_461238 | ais | aluminum-inducible protein | - | NP_416755 | ais | hypothetical protein | GCO | 65.32% | 47.68% |
| NP_461239 | yfbE | 4-amino-4-deoxy-L-arabinose lipopolysaccharide-modifying enzyme | - | NP_416756 | yfbE | uridine 5'-(beta-1-threo-pentapyranosyl-4-ulose diphosphate) aminotransferase, PLP-dependent | GCO | 79.48% | 56.21% |
| NP_461240 | pmrF | putative glycosyl transferase | - | NP_416757 | yfbF | undecaprenyl phosphate-L-Ara4FN transferase | GCO | 78.61% | 53.65% |
| NP_461241 | yfbG | hypothetical protein | - | NP_416758 | yfbG | hypothetical protein | GCO | 79.39% | 54.16% |
| NP_461242 | - | putative cytoplasmic protein | - | NP_416759 | yfbH | hypothetical protein | GCO | 69.79% | 57% |
| NP_461243 | pqaB | putative melittin resistance protein | - | NP_416760 | arnT | 4-amino-4-deoxy-L-arabinose transferase | GCO | 62.54% | 54.03% |
| NP_461244 | - | putative inner membrane protein | - | YP_588462 | yfbW | hypothetical protein | GCO | 75.64% | 60.11% |
| NP_461245 | - | hypothetical protein | - | NP_416761 | yfbJ | hypothetical protein | GCO | 47.54% | 54.23% |
| NP_461246 | pmrD | polymyxin resistance protein B | - | NP_416762 | pmrD | polymyxin resistance protein B | GCO | 59.49% | 48.06% |
| NP_461247 | menE | O-succinylbenzoic acid--CoA ligase | - | NP_416763 | menE | O-succinylbenzoic acid--CoA ligase | GCO | 79.06% | 59.94% |
| NP_461248 | menC | O-succinylbenzoate synthase | - | NP_416764 | menC | O-succinylbenzoate synthase | GCO | 84.68% | 60.12% |
| NP_461249 | menB | naphthoate synthase | - | NP_416765 | menB | naphthoate synthase | GCO | 96.84% | 56.29% |
| NP_461250 | yfbB | putative enzyme | - | NP_416766 | yfbB | predicted peptidase | GCO | 76.89% | 58.36% |
| NP_461251 | menD | 2-oxoglutarate decarboxylase | - | NP_416767 | menD | 2-succinyl-6-hydroxy-2,4-cyclohexadiene-1-carboxylate synthase | GCO | 91.00% | 59.48% |
| NP_461252 | menF | menaquinone-specific isochorismate synthase | - | NP_416768 | menF | menaquinone-specific isochorismate synthase | GCO | 78.65% | 56.86% |
| NP_461253 | elaB | putative inner membrane protein | - | NP_416769 | elaB | hypothetical protein | GCO | 65.47% | 51.60% |
| NP_461254 | elaA | putative acyltransferase | - | NP_416770 | elaA | predicted acyltransferase with acyl-CoA N-acyltransferase domain | GCO | 75.16% | 54.11% |
| NP_461255 | elaC | ribonuclease Z | - | NP_416771 | elaC | ribonuclease Z | GCO | 80.98% | 55.55% |
| NP_461256 | - | putative chemotaxis signal transduction protein | - |  |  |  | NO HOMOLOG |  | 52.09% |
| NP_461257 | yfbK | hypothetical protein | - | NP_416773 | yfbK | hypothetical protein | nGCO | 70.35% | 52.80% |
| NP_461258 | nuoN | NADH dehydrogenase subunit N | - | NP_416779 | nuoN | NADH dehydrogenase subunit N | GCO | 84% | 59.53% |
| NP_461259 | nuoM | NADH dehydrogenase subunit M | - | NP_416780 | nuoM | NADH dehydrogenase subunit M | GCO | 86.64% | 56.20% |
| NP_461260 | nuoL | NADH dehydrogenase subunit L | - | NP_416781 | nuoL | NADH dehydrogenase subunit L | GCO | 92.34% | 57.81% |
| NP_461261 | nuoK | NADH dehydrogenase kappa subunit | - | NP_416782 | nuoK | NADH dehydrogenase subunit K | GCO | 70% | 54.45% |
| NP_461262 | nuoJ | NADH dehydrogenase subunit J | - | NP_416783 | nuoJ | NADH dehydrogenase subunit J | GCO | 90.76% | 57.83% |
| NP_461263 | nuoI | NADH dehydrogenase subunit I | - | NP_416784 | nuoI | NADH dehydrogenase subunit I | GCO | 99.44% | 54.14% |
| NP_461264 | nuoH | NADH dehydrogenase subunit H | - | NP_416785 | nuoH | NADH dehydrogenase subunit H | GCO | 91.07% | 54.90% |
| NP_461265 | nuoG | NADH dehydrogenase gamma subunit | - | NP_416786 | nuoG | NADH dehydrogenase subunit G | GCO | 93.39% | 58.92% |
| NP_461266 | nuoF | NADH dehydrogenase I chain F | - | NP_416787 | nuoF | NADH:ubiquinone oxidoreductase, chain F | GCO | 98.87% | 58.74% |
| NP_461267 | nuoE | ATP synthase subunit E | - | NP_416788 | nuoE | NADH dehydrogenase subunit E | GCO | 96.98% | 56.28% |
| NP_461268 | nuoC | NADH dehydrogenase I chain C/D | - | NP_416789 | nuoC | NADH:ubiquinone oxidoreductase, chain C,D | GCO | 94.83% | 56.84% |
| NP_461269 | nuoB | NADH dehydrogenase beta subunit | - | NP_416790 | nuoB | NADH dehydrogenase subunit B | GCO | 99.09% | 54.44% |
| NP_461270 | nuoA | NADH dehydrogenase alpha subunit | - | NP_416791 | nuoA | NADH dehydrogenase subunit A | GCO | 97.95% | 53.15% |
| NP_461271 | - | putative cytoplasmic protein | - |  |  |  | NO HOMOLOG |  | 38.56% |
| NP_461272 | lrhA | NADH dehydrogenase transcriptional repressor | + | NP_416792 | lrhA | DNA-binding transcriptional repressor of flagellar, motility and chemotaxis genes | GCO | 75.96% | 52.92% |
| NP_461273 | yfbQ | aspartate aminotransferase | - | NP_416793 | yfbQ | aspartate aminotransferase | GCO | 96.53% | 51.44% |
| NP_461274 | - | hypothetical protein | - | NP_416794 | yfbR | hypothetical protein | GCO | 91.45% | 50% |
| NP_461275 | yfbS | putative response regulator | - | NP_416795 | yfbS | predicted transporter | GCO | 84.91% | 55.71% |
| NP_461276 | yfbT | putative phosphatase | - | NP_416796 | yfbT | predicted hydrolase or phosphatase | GCO | 80.46% | 57.96% |
| NP_461277 | yfbU | hypothetical protein | - | NP_416797 | yfbU | hypothetical protein | GCO | 95.73% | 52.52% |
| NP_461278 | - | hypothetical protein | - | NP_416798 | yfbV | hypothetical protein | GCO | 95.36% | 53.94% |
| NP_461279 | ackA | acetate/propionate kinase | - | NP_416799 | ackA | acetate kinase | GCO | 94.25% | 52.28% |
| NP_461280 | pta | phosphate acetyltransferase | - | NP_416800 | pta | phosphate acetyltransferase | GCO | 94.81% | 58.13% |
| NP_461281 | yfcC | putative integral membrane protein | - | NP_416801 | yfcC | predicted inner membrane protein | GCO | 89.92% | 55.02% |
| NP_461282 | - | putative transketolase | - |  |  |  | NO HOMOLOG |  | 54.40% |
| NP_461283 | - | putative transketolase | - |  |  |  | NO HOMOLOG |  | 56.07% |
| NP_461284 | ulaA | ascorbate-specific PTS system enzyme IIC | - | NP_418614 | ulaA | ascorbate-specific PTS system enzyme IIC | nGCO | 27.77% | 52.29% |
| NP_461285 | - | putative cytoplasmic protein | - |  |  |  | NO HOMOLOG |  | 47.25% |
| NP_461286 | - | putative phosphotransferase system enzyme II A component | - | NP_418616 | ulaC | L-ascorbate-specific enzyme IIA component of PTS | nGCO | 37.68% | 49.77% |
| NP_461287 | - | putative transcriptional regulator | - | YP_026222 | gntR | DNA-binding transcriptional repressor | nGCO | 34.42% | 55.68% |
| NP_461288 | - | putative NTP pyrophosphohydrolase | - | NP_416802 | yfcD | predicted NUDIX hydrolase | GCO | 84.44% | 54.41% |
| NP_461289 | yfcE | phosphodiesterase | - | NP_416803 | yfcE | phosphodiesterase | GCO | 85.16% | 54.71% |
| NP_461290 | yfcF | putative glutathione S-transferase | - | NP_416804 | yfcF | predicted enzyme | GCO | 80.84% | 56.27% |
| NP_461291 | yfcG | putative glutathione S-transferase | - | NP_416805 | yfcG | predicted glutathione S-transferase | GCO | 83.17% | 53.24% |
| NP_461292 | yfcH | putative sugar nucleotide epimerase | - | NP_416807 | yfcH | conserved protein with NAD(P)-binding Rossmann-fold domain | nGCO | 79.92% | 57.49% |
| NP_461293 | hisP | histidine/lysine/arginine/ornithine transport protein | - | NP_416809 | hisP | histidine/lysine/arginine/ornithine transporter subunit | GCO | 90.66% | 53.02% |
| NP_461294 | hisM | histidine/lysine/arginine/ornithine transport protein | - | NP_416810 | hisM | histidine/lysine/arginine/ornithine transporter subunit | GCO | 86.63% | 51.69% |
| NP_461295 | hisQ | histidine/lysine/arginine/ornithine transport protein | - | NP_416811 | hisQ | histidine/lysine/arginine/ornithine transporter subunit | GCO | 82.89% | 55.16% |
| NP_461296 | hisJ | histidine transport protein | - | NP_416812 | hisJ | histidine/lysine/arginine/ornithine transporter subunit | GCO | 98.31% | 52.10% |
| NP_461297 | argT | lysine/arginine/ornithine transport protein | - | NP_416813 | argT | lysine/arginine/ornithine transporter subunit | GCO | 91.15% | 50.57% |
| NP_461298 | ubiX | 3-octaprenyl-4-hydroxybenzoate carboxy-lyase | - | NP_416814 | ubiX | 3-octaprenyl-4-hydroxybenzoate carboxy-lyase | GCO | 91.00% | 55.08% |
| NP_461299 | - | putative amino acid transporter | - | NP_414794 | mmuP | CP4-6 prophage; predicted S-methylmethionine transporter | nGCO | 31.74% | 53.09% |
| NP_461300 | - | putative cytoplasmic protein | - |  |  |  | NO HOMOLOG |  | 50.63% |
| NP_461301 | - | putative amino acid transporter | - | NP_416009 | gadC | predicted glutamate:gamma-aminobutyric acid antiporter | nGCO | 24.88% | 50.56% |
| NP_461302 | - | putative diaminopimelate decarboxylase | - | NP_417315 | lysA | diaminopimelate decarboxylase, PLP-binding | nGCO | 26.81% | 49.78% |
| NP_461303 | - | putative regulatory protein | - | NP_416724 | atoC | fused response regulator of ato operon, in two-component system with AtoS: response regulator/sigma54 interaction protein | nGCO | 41.30% | 52.10% |
| NP_461304 | purF | amidophosphoribosyltransferase | - | NP_416815 | purF | amidophosphoribosyltransferase | GCO | 96.23% | 53.62% |
| NP_461305 | cvpA | colicin V production protein | - | NP_416816 | cvpA | membrane protein required for colicin V production | GCO | 96.27% | 51.12% |
| NP_461306 | dedD | putative lipoprotein | - | NP_416817 | dedD | hypothetical protein | GCO | 64.28% | 58.81% |
| NP_461307 | folC | multifunctional folylpolyglutamate synthase | - | NP_416818 | folC | bifunctional folylpolyglutamate synthase/ dihydrofolate synthase | GCO | 82.81% | 58.47% |
| NP_461308 | accD | acetyl-CoA carboxylase beta subunit | - | NP_416819 | accD | acetyl-CoA carboxylase subunit beta | GCO | 92.76% | 57.15% |
| NP_461309 | dedA | hypothetical protein | - | NP_416820 | dedA | conserved inner membrane protein | GCO | 88.58% | 50.90% |
| NP_461310 | truA | tRNA pseudouridine synthase A | - | NP_416821 | truA | tRNA pseudouridine synthase A | GCO | 88.14% | 55.59% |
| NP_461311 | usg | hypothetical protein | - | NP_416822 | usg | hypothetical protein | GCO | 78.04% | 57.79% |
| NP_461312 | pdxB | erythronate-4-phosphate dehydrogenase | - | NP_416823 | pdxB | erythronate-4-phosphate dehydrogenase | GCO | 89.12% | 56.64% |
| NP_461313 | flk | flagellar protein | - | NP_416824 | flk | predicted flagella assembly protein | GCO | 58.22% | 55.18% |
| NP_461314 | - | hypothetical protein | - | NP_416825 | yfcJ | predicted transporter | GCO | 78.06% | 60.89% |
| NP_461315 | - | putative cytoplasmic protein | - |  |  |  | NO HOMOLOG |  | 51.20% |
| NP_461316 | - | putative regulatory protein | - | NP_417655 | sfsB | DNA-binding transcriptional activator of maltose metabolism | nGCO | 50.68% | 46.98% |
| NP_461317 | - | putative cytoplasmic protein | - |  |  |  | NO HOMOLOG |  | 50.67% |
| NP_461318 | - | putative periplasmic protein | - |  |  |  | NO HOMOLOG |  | 51.25% |
| NP_461319 | - | putative inner membrane protein | - |  |  |  | NO HOMOLOG |  | 40.63% |
| NP_461320 | fabB | 3-oxoacyl-(acyl carrier protein) synthase | - | NP_416826 | fabB | 3-oxoacyl-(acyl carrier protein) synthase | GCO | 91.33% | 57.36% |
| NP_461321 | - | hypothetical protein | - | NP_416827 | trmC | hypothetical protein | GCO | 74.17% | 57.64% |
| NP_461322 | yfcL | putative cytoplasmic protein | - | NP_416828 | yfcL | hypothetical protein | GCO | 85.71% | 53.26% |
| NP_461323 | yfcM | putative cytoplasmic protein | - | NP_416829 | yfcM | hypothetical protein | GCO | 92.30% | 53.36% |
| NP_461324 | yfcA | putative permease | - | NP_416830 | yfcA | conserved inner membrane protein | GCO | 86.61% | 54.69% |
| NP_461325 | mepA | penicillin-insensitive murein endopeptidase | - | NP_416831 | mepA | penicillin-insensitive murein endopeptidase | GCO | 82.11% | 58.54% |
| NP_461326 | aroC | chorismate synthase | - | NP_416832 | aroC | chorismate synthase | GCO | 95.56% | 58.65% |
| NP_461327 | yfcB | putative methylase | - | NP_416833 | prmB | N5-glutamine methyltransferase | GCO | 90.64% | 53.16% |
| NP_461328 | yfcN | hypothetical protein | - | NP_416834 | yfcN | hypothetical protein | GCO | 93.98% | 54.16% |
| NP_461329 | sixA | phosphohistidine phosphatase | - | NP_416842 | sixA | phosphohistidine phosphatase | GCO | 89.44% | 54.93% |
| NP_461330 | yfcX | putative dehydrogenase | - | NP_416843 | yfcX | fused enoyl-CoA hydratase and epimerase and isomerase/3-hydroxyacyl-CoA dehydrogenase | GCO | 82.86% | 53.95% |
| NP_461331 | yfcY | acetyl-CoA acetyltransferase | - | NP_416844 | yfcY | acetyl-CoA acetyltransferase | GCO | 87.84% | 59.64% |
| NP_461332 | yfcZ | putative cytoplasmic protein | - | NP_416845 | yfcZ | hypothetical protein | GCO | 76.59% | 52.98% |
| NP_461333 | fadL | outer membrane-bound fatty acid transporter | - | NP_416846 | fadL | long-chain fatty acid outer membrane transporter | GCO | 91.51% | 53.04% |
| NP_461334 | vacJ | lipoprotein precursor | - | NP_416848 | vacJ | predicted lipoprotein | GCO | 94.02% | 54.23% |
| NP_461335 | yfdC | hypothetical protein | - | NP_416849 | yfdC | predicted inner membrane protein | GCO | 83.21% | 49.68% |
| NP_461336 | pgtE | outer membrane protein E precursor | + | NP_415097 | ompT | DLP12 prophage; outer membrane protease VII (outer membrane protein 3b) | nGCO | 46.56% | 46.32% |
| NP_461337 | pgtA | activator | + | NP_417049 | yfhA | predicted DNA-binding response regulator in two-component system | nGCO | 25.64% | 51.84% |
| NP_461338 | pgtB | phosphoglycerate transport system sensor protein | - |  |  |  | NO HOMOLOG |  | 53.86% |
| NP_461339 | pgtC | phosphoglycerate transport regulatory protein precursor | - |  |  |  | NO HOMOLOG |  | 53.93% |
| NP_461340 | pgtP | transporter | - | NP_416743 | glpT | sn-glycerol-3-phosphate transporter | nGCO | 35.46% | 49.78% |
| NP_461341 | - | putative inner membrane protein | - | NP_416878 | yfdY | predicted inner membrane protein | GCO | 67.50% | 43.62% |
| NP_461342 | ddg | lipid A biosynthesis lauroyl acyltransferase | - | NP_416879 | ddg | lipid A biosynthesis palmitoleoyl acyltransferase | GCO | 90.84% | 54.07% |
| NP_461343 | yfdZ | hypothetical protein | - | NP_416880 | yfdZ | hypothetical protein | GCO | 95.87% | 53.99% |
| NP_461344 | glk | glucokinase | - | NP_416889 | glk | glucokinase | GCO | 93.76% | 52.79% |
| NP_461345 | - | hypothetical protein | - | NP_416890 | yfeO | hypothetical protein | GCO | 62.50% | 59.22% |
| NP_461346 | - | indolepyruvate decarboxylase | - | YP_025294 | ilvI | acetolactate synthase III large subunit | nGCO | 22.85% | 59.10% |
| NP_461347 | - | putative oxidoreductase | - | NP_417474 | yghZ | aldo-keto reductase | nGCO | 60.48% | 53.85% |
| NP_461348 | ypeC | putative periplasmic protein | - | NP_416891 | ypeC | hypothetical protein | GCO | 76.92% | 55.35% |
| NP_461349 | mntH | putative manganese transport protein MntH | - | NP_416893 | mntH | manganese transport protein MntH | GCO | 91.50% | 54.83% |
| NP_461350 | nupC | nucleoside transport | - | NP_416894 | nupC | nucleoside (except guanosine) transporter | GCO | 88% | 50.78% |
| NP_461351 | yfeA | hypothetical protein | - | NP_416896 | yfeA | predicted diguanylate cyclase | GCO | 67.45% | 50.59% |
| NP_461352 | yfeC | putative negative regulator | - | NP_416897 | yfeC | predicted DNA-binding transcriptional regulator | GCO | 78.26% | 51.51% |
| NP_461353 | yfeD | putative negative regulator | - | NP_416898 | yfeD | predicted DNA-binding transcriptional regulator | GCO | 62.79% | 48.34% |
| NP_461354 | gltX | glutamyl-tRNA synthetase | - | NP_416899 | gltX | glutamyl-tRNA synthetase | GCO | 95.11% | 55.29% |
| NP_461355 | xapR | XapA transcriptional regulator | - | NP_416900 | xapR | DNA-binding transcriptional activator | GCO | 80.88% | 50.28% |
| NP_945166 | - | hypothetical protein | - |  |  |  | NO HOMOLOG |  | 33.96% |
| NP_461356 | xapB | xanthosine permease | - | NP_416901 | xapB | xanthosine transporter | nGCO | 78.70% | 49.00% |
| NP_461358 | yfeN | putative outer membrane protein | - | NP_416903 | yfeN | conserved outer membrane protein | GCO | 79.41% | 45.80% |
| NP_461359 | yfeR | putative transcriptional regulator | - | NP_416904 | yfeR | predicted DNA-binding transcriptional regulator | GCO | 79.22% | 56.63% |
| NP_461360 | yfeH | putative Na+-dependent transporter | - | NP_416905 | yfeH | predicted inner membrane protein | GCO | 81.62% | 55.35% |
| NP_461361 | - | putative cytoplasmic protein | - | YP_588464 | ypeB | hypothetical protein | GCO | 94.44% | 51.31% |
| NP_461362 | lig | DNA ligase | - | NP_416906 | ligA | NAD-dependent DNA ligase LigA | GCO | 92.84% | 56.89% |
| NP_461363 | zipA | cell division protein ZipA | - |  |  |  | NO HOMOLOG |  | 57.54% |
| NP_461364 | cysZ | putative sulfate transport protein CysZ | - | NP_416908 | cysZ | putative sulfate transport protein CysZ | GCO | 89.72% | 52.75% |
| NP_461365 | cysK | O-acetylserine sulfhydrolase A | - | NP_416909 | cysK | cysteine synthase A, O-acetylserine sulfhydrolase A subunit | GCO | 89.16% | 53.39% |
| NP_461366 | ptsH | phosphohistidinoprotein-hexose phosphotransferase | - | NP_416910 | ptsH | phosphohistidinoprotein-hexose phosphotransferase component of PTS system (Hpr) | GCO | 100% | 51.16% |
| NP_461367 | ptsI | PEP-protein phosphotransferase | - | NP_416911 | ptsI | PEP-protein phosphotransferase of PTS system (enzyme I) | GCO | 94.26% | 51.79% |
| NP_461368 | crr | glucose-specific PTS system enzyme IIA component | - | NP_416912 | crr | glucose-specific PTS system enzyme IIA component | GCO | 90.53% | 47.45% |
| NP_461369 | - | putative cytoplasmic protein | - |  |  |  | NO HOMOLOG |  | 58.33% |
| NP_461370 | pdxK | pyridoxine kinase | - | NP_416913 | pdxK | pyridoxine kinase | nGCO | 65.23% | 57.09% |
| NP_461371 | ptsJ | putative regulatory protein | - | NP_418760 | yjiR | fused predicted DNA-binding transcriptional regulator/predicted aminotransferase | nGCO | 30.21% | 58.93% |
| NP_461372 | yfeJ | glutamine amidotransferase | - |  |  |  | NO HOMOLOG |  | 55% |
| NP_461373 | yfeK | putative periplasmic protein | - | NP_416914 | yfeK | hypothetical protein | nGCO | 84.03% | 49.34% |
| NP_461374 | yfeL | putative membrane carboxypeptidase | - |  |  |  | NO HOMOLOG |  | 50% |
| NP_461375 | cysM | cysteine synthase B | - | NP_416916 | cysM | cysteine synthase B (O-acetylserine sulfhydrolase B) | GCO | 88.77% | 57.12% |
| NP_461376 | cysA | sulfate permease A protein | - | NP_416917 | cysA | sulfate/thiosulfate transporter subunit | GCO | 94.24% | 58.37% |
| NP_461377 | cysW | thiosulfate permease W protein | - | YP_026168 | cysW | sulfate/thiosulfate transporter subunit | GCO | 91.75% | 55.25% |
| NP_461378 | cysU | thiosulfate transport protein | - | NP_416919 | cysU | sulfate/thiosulfate transporter subunit | GCO | 84.11% | 55.87% |
| NP_461379 | cysP | thiosulfate transport protein | - | NP_416920 | cysP | thiosulfate transporter subunit | GCO | 87.83% | 53.78% |
| NP_461380 | ucpA | short chain dehydrogenase | - | NP_416921 | ucpA | short chain dehydrogenase | GCO | 92.77% | 53.91% |
| NP_461381 | - | putative iron-dependent peroxidase | - | NP_416926 | yfeX | hypothetical protein | GCO | 94.31% | 55.55% |
| NP_461382 | - | putative outer membrane lipoprotein | - | NP_416927 | yfeY | hypothetical protein | GCO | 76.43% | 56.07% |
| NP_461383 | yfeZ | putative inner membrane protein | - | NP_416928 | yfeZ | predicted inner membrane protein | GCO | 65.10% | 56% |
| NP_461384 | - | putative acetyltransferase | - | NP_416929 | ypeA | putative acetyltransferase | GCO | 93.61% | 53.05% |
| NP_461385 | amiA | N-acetylmuramoyl-l-alanine amidase I | - | NP_416930 | amiA | N-acetylmuramoyl-l-alanine amidase I | GCO | 88.23% | 50.57% |
| NP_461386 | hemF | coproporphyrinogen III oxidase | - | NP_416931 | hemF | coproporphyrinogen III oxidase | GCO | 90.63% | 57.33% |
| NP_461387 | - | putative inner membrane protein | - |  |  |  | NO HOMOLOG |  | 43.33% |
| NP_461388 | - | putative cytoplasmic protein | - |  |  |  | NO HOMOLOG |  | 52.97% |
| NP_461389 | eutR | putative ethanolamine operon regulator | - | NP_416932 | yfeG | predicted DNA-binding transcriptional regulator | GCO | 91.97% | 54.32% |
| NP_461390 | eutK | putative carboxysome structural protein | - | NP_416933 | yffI | predicted carboxysome structural protein with predicted role in ethanolamine utilization | GCO | 66.46% | 60.20% |
| NP_461391 | eutL | putative carboxysome structural protein | - | NP_416934 | eutL | predicted carboxysome structural protein with predicted role in ethanolamine utilization | GCO | 94.06% | 59.39% |
| NP_461392 | eutC | ethanolamine ammonia-lyase small subunit | - | NP_416935 | eutC | ethanolamine ammonia-lyase small subunit | GCO | 90.93% | 60.75% |
| NP_461393 | eutB | ethanolamine ammonia-lyase heavy chain | - | NP_416936 | eutB | ethanolamine ammonia-lyase, large subunit, heavy chain | GCO | 98.01% | 57.04% |
| NP_461394 | eutA | ethanolamine utilization protein | - | NP_416946 | eutA | reactivating factor for ethanolamine ammonia lyase | GCO | 87.79% | 61.46% |
| NP_461395 | eutH | putative transport protein | - | NP_416947 | eutH | predicted inner membrane protein | GCO | 91.91% | 57.13% |
| NP_461396 | eutG | putative transport protein | - | NP_416948 | eutG | predicted alcohol dehydrogenase in ethanolamine utilization | GCO | 81.47% | 62.87% |
| NP_461397 | eutJ | ethanolamine utilization protein | - | NP_416949 | eutJ | predicted chaperonin, ethanolamine utilization protein | GCO | 83.45% | 61.30% |
| NP_461398 | eutE | putative aldehyde oxidoreductase | - | NP_416950 | eutE | predicted aldehyde dehydrogenase, ethanolamine utilization protein | GCO | 89.93% | 59.90% |
| NP_461399 | eutN | putative detox protein | - | NP_416951 | cchB | predicted carboxysome structural protein, ethanolamine utilization protein | GCO | 91.57% | 57.33% |
| NP_461400 | eutM | putative detox protein | - | NP_416952 | cchA | predicted carboxysome structural protein, ethanolamine utilization protein | GCO | 82.10% | 60.48% |
| NP_461401 | eutD | phosphate acetyltransferase | - | NP_416953 | eutI | predicted phosphotransacetylase subunit | GCO | 85.20% | 62.14% |
| NP_461402 | eutT | putative cobalamin adenosyltransferase | - | NP_416954 | eutT | predicted cobalamin adenosyltransferase in ethanolamine utilization | GCO | 85.28% | 59.07% |
| NP_461403 | eutQ | putative ethanolamine utilization protein | - | NP_416955 | eutQ | hypothetical protein | GCO | 87.98% | 57.10% |
| NP_461404 | eutP | putative ethanolamine utilization protein | - | NP_416956 | eutP | conserved protein with nucleoside triphosphate hydrolase domain | GCO | 84.90% | 51.45% |
| NP_461405 | eutS | putative carboxysome structural protein | - | NP_416957 | ypfE | predicted carboxysome structural protein with predicted role in ethanol utilization | GCO | 94.59% | 50.89% |
| NP_461406 | tnpA_3 | transposase | - |  |  |  | NO HOMOLOG |  | 45.96% |
| NP_461407 | maeB | phosphate acetyltransferase | - | NP_416958 | maeB | malic enzyme | GCO | 91.04% | 56.05% |
| NP_461408 | talA | transaldolase | - | NP_416959 | talA | transaldolase A | GCO | 87.65% | 53.62% |
| NP_461409 | tktB | transketolase | - | NP_416960 | tktB | transketolase 2, thiamin-binding | GCO | 92.94% | 55.97% |
| NP_461410 | - | putative cytoplasmic protein | - |  |  |  | NO HOMOLOG |  | 46.75% |
| NP_461411 | ypfG | putative periplasmic protein | - | NP_416961 | ypfG | hypothetical protein | GCO | 77.23% | 56.70% |
| NP_461412 | yffH | putative pyrophosphohydrolase | - | NP_416962 | yffH | predicted NUDIX hydrolase | GCO | 80.62% | 48.78% |
| NP_461413 | - | hypothetical protein | - | NP_416925 | yfeW | hypothetical protein | nGCO | 72.91% | 46.03% |
| NP_461414 | aegA | putative oxidoreductase | - | NP_416963 | aegA | fused predicted oxidoreductase: FeS binding subunit/NAD/FAD-binding subunit | GCO | 83.56% | 58.30% |
| NP_461415 | narQ | sensory histidine kinase | - | NP_416964 | narQ | sensory histidine kinase in two-component regulatory system with NarP (NarL) | GCO | 82.47% | 54.08% |
| NP_461416 | acrD | aminoglycoside/multidrug efflux pump | - | NP_416965 | acrD | aminoglycoside/multidrug efflux system | GCO | 92.86% | 55.36% |
| NP_461417 | yffB | putative glutaredoxin | - | NP_416966 | yffB | hypothetical protein | GCO | 80.50% | 51.54% |
| NP_461418 | dapE | succinyl-diaminopimelate desuccinylase | - | NP_416967 | dapE | succinyl-diaminopimelate desuccinylase | GCO | 94.40% | 54.96% |
| NP_461419 | - | putative inner membrane protein | - | YP_588465 | ypfN | hypothetical protein | GCO | 71.42% | 45.77% |
| NP_461420 | ypfI | putative acetyltransferase | - | NP_416969 | ypfI | predicted hydrolase | GCO | 67.52% | 62.60% |
| NP_461421 | - | putative inner membrane protein | - | NP_416970 | ypfJ | hypothetical protein | GCO | 84.96% | 56.48% |
| NP_461422 | purC | phosphoribosylaminoimidazole-succinocarboxamide synthase | - | NP_416971 | purC | phosphoribosylaminoimidazole-succinocarboxamide synthase | GCO | 94.93% | 52.24% |
| NP_461423 | nlpB | lipoprotein | - | NP_416972 | nlpB | lipoprotein | GCO | 82.84% | 57.87% |
| NP_461424 | dapA | dihydrodipicolinate synthase | - | NP_416973 | dapA | dihydrodipicolinate synthase | GCO | 88.01% | 53.69% |
| NP_461425 | gcvR | gcv operon transcriptional repressor | - | NP_416974 | gcvR | DNA-binding transcriptional repressor, regulatory protein accessory to GcvA | GCO | 90% | 48.98% |
| NP_461426 | bcp | bacterioferritin comigratory protein | - | NP_416975 | bcp | thioredoxin-dependent thiol peroxidase | GCO | 98.07% | 48.19% |
| NP_461427 | - | putative glycerate kinase | - |  |  |  | NO HOMOLOG |  | 58.47% |
| NP_461428 | perM | putative permease | - | NP_416988 | yfgO | predicted inner membrane protein | GCO | 73.14% | 54.96% |
| NP_461429 | - | hypothetical protein | - | NP_416989 | yfgC | predicted peptidase | GCO | 85.83% | 57.37% |
| NP_461430 | yfgD | putative arsenate reductase | - | NP_416990 | yfgD | predicted oxidoreductase | GCO | 83.19% | 56.94% |
| NP_461431 | yfgE | DNA replication initiation factor | - | NP_416991 | hda | DNA replication initiation factor | GCO | 94.09% | 53.71% |
| NP_461432 | uraA | uracil transport protein | - | NP_416992 | uraA | uracil transporter | GCO | 91.29% | 56.04% |
| NP_461433 | upp | uracil phosphoribosyltransferase | - | NP_416993 | upp | uracil phosphoribosyltransferase | GCO | 99.03% | 53.26% |
| NP_461434 | purM | phosphoribosylaminoimidazole synthetase | - | NP_416994 | purM | phosphoribosylaminoimidazole synthetase | GCO | 88.40% | 55.46% |
| NP_461435 | purN | phosphoribosylglycinamide formyltransferase | - | NP_416995 | purN | phosphoribosylglycinamide formyltransferase | GCO | 83.96% | 56.02% |
| NP_461436 | ppk | polyphosphate kinase | - | NP_416996 | ppk | polyphosphate kinase | GCO | 94.33% | 49.00% |
| NP_461437 | ppx | exopolyphosphatase | - | NP_416997 | ppx | exopolyphosphatase | GCO | 89.27% | 54.02% |
| NP_461438 | - | putative diguanylate cyclase | - | NP_416998 | yfgF | predicted inner membrane protein | GCO | 70.80% | 47.69% |
| NP_461440 | - | putative inner membrane protein | - |  |  |  | NO HOMOLOG |  | 47.50% |
| NP_461441 | - | putative inner membrane protein | - | NP_416999 | yfgG | hypothetical protein | nGCO | 84.12% | 47.91% |
| NP_461443 | - | putative cytoplasmic protein | - |  |  |  | NO HOMOLOG |  | 38.48% |
| NP_461444 | - | putative transposase | - |  |  |  | NO HOMOLOG |  | 47.33% |
| NP_461445 | guaA | bifunctional GMP synthase/glutamine amidotransferase protein | - | NP_417002 | guaA | bifunctional GMP synthase/glutamine amidotransferase protein | GCO | 98.47% | 55.83% |
| NP_461446 | guaB | inositol-5-monophosphate dehydrogenase | - | NP_417003 | guaB | inositol-5-monophosphate dehydrogenase | GCO | 86.47% | 56.98% |
| NP_461447 | xseA | exodeoxyribonuclease VII large subunit | - | NP_417004 | xseA | exodeoxyribonuclease VII large subunit | GCO | 84.11% | 58.07% |
| NP_461448 | shdA | AIDA autotransporter-like protein | - |  |  |  | NO HOMOLOG |  | 58.07% |
| NP_461449 | ratB | putative outer membrane protein | - |  |  |  | NO HOMOLOG |  | 58.73% |
| NP_461450 | ratA | putative outer membrane protein | - |  |  |  | NO HOMOLOG |  | 58.98% |
| NP_461451 | sinI | putative outer membrane protein | - |  |  |  | NO HOMOLOG |  | 53.12% |
| NP_461452 | sinH | intimin-like protein | - | NP_415738 | ychP | predicted invasin | nGCO | 31.09% | 52.16% |
| NP_461453 | yfgJ | putative cytoplasmic protein | - | NP_417005 | yfgJ | hypothetical protein | GCO | 63.38% | 48.19% |
| NP_461454 | engA | GTP-binding protein EngA | - | NP_417006 | der | GTP-binding protein EngA | GCO | 94.28% | 54.71% |
| NP_461455 | yfgL | putative serine/threonine protein kinase | - | NP_417007 | yfgL | protein assembly complex, lipoprotein component | GCO | 84.69% | 54.96% |
| NP_461456 | yfgM | putative inner membrane protein | - | NP_417008 | yfgM | hypothetical protein | GCO | 82.52% | 53.78% |
| NP_461457 | hisS | histidyl-tRNA synthetase | - | NP_417009 | hisS | histidyl-tRNA synthetase | GCO | 95.28% | 56.23% |
| NP_461458 | gcpE | 4-hydroxy-3-methylbut-2-en-1-yl diphosphate synthase | - | NP_417010 | ispG | 4-hydroxy-3-methylbut-2-en-1-yl diphosphate synthase | GCO | 95.43% | 54.78% |
| NP_461459 | yfgA | hypothetical protein | - | NP_417011 | yfgA | hypothetical protein | GCO | 71.21% | 58.00% |
| NP_461460 | yfgB | putative FeS redox enzyme | - | NP_417012 | yfgB | predicted enzyme | GCO | 95.61% | 52.09% |
| NP_461461 | ndk | nucleoside diphosphate kinase | - | NP_417013 | ndk | nucleoside diphosphate kinase | GCO | 96.50% | 55.55% |
| NP_461462 | - | putative polyferredoxin | - |  |  |  | NO HOMOLOG |  | 59.83% |
| NP_461463 | - | putative dimethylsulfoxide reductase | - | NP_416107 | ynfH | oxidoreductase, membrane subunit | nGCO | 29.28% | 60.49% |
| NP_461464 | - | putative anaerobic dimethylsulfoxide reductase | - | NP_415415 | dmsB | dimethyl sulfoxide reductase, anaerobic, subunit B | GCO | 56.25% | 53.96% |
| NP_461465 | - | putative anaerobic dimethylsulfoxide reductase | - | NP_415414 | dmsA | dimethyl sulfoxide reductase, anaerobic, subunit A | GCO | 41.90% | 54.61% |
| NP_461466 | pbpC | penicillin-binding protein 1c | - | NP_417014 | pbpC | fused transglycosylase/transpeptidase | GCO | 81.20% | 61.26% |
| NP_461467 | - | putative inner membrane lipoprotein | - | NP_417015 | yfhM | hypothetical protein | GCO | 78.10% | 58.45% |
| NP_461468 | sseA | putative sulfurtransferase | - | NP_417016 | sseA | 3-mercaptopyruvate sulfurtransferase | GCO | 73.83% | 59.31% |
| NP_461469 | - | putative cytoplasmic protein | + |  |  |  | NO HOMOLOG |  | 44.69% |
| NP_461470 | sseB | enhanced serine sensitivity protein | - | NP_417017 | sseB | rhodanase-like enzyme, sulfur transfer from thiosulfate | GCO | 85.65% | 55.08% |
| NP_461471 | pepB | aminopeptidase B | - | NP_417018 | pepB | aminopeptidase B | GCO | 85.48% | 56.46% |
| NP_461472 | yfhJ | hypothetical protein | - | NP_417019 | yfhJ | hypothetical protein | GCO | 92.42% | 49.25% |
| NP_461473 | fdx | electron carrer protein | - | NP_417020 | fdx | [2Fe-2S] ferredoxin | GCO | 81.98% | 52.97% |
| NP_461474 | hscA | chaperone protein HscA | - | NP_417021 | hscA | chaperone protein HscA | GCO | 89.44% | 59.42% |
| NP_461475 | hscB | co-chaperone HscB | - | NP_417022 | hscB | co-chaperone HscB | GCO | 84.79% | 54.45% |
| NP_461476 | iscA | iron-sulfur cluster assembly protein | - | NP_417023 | iscA | iron-sulfur cluster assembly protein | GCO | 86.91% | 50.61% |
| NP_461477 | nifU | NifU-like protein | - | NP_417024 | iscU | scaffold protein | GCO | 98.43% | 51.16% |
| NP_461478 | nifS | cysteine desulfurase | - | YP_026169 | iscS | cysteine desulfurase | GCO | 94.30% | 54.89% |
| NP_461479 | yfhP | putative iron-sulfur cluster regulatory protein | - | NP_417026 | iscR | DNA-binding transcriptional repressor | GCO | 89.02% | 55.95% |
| NP_461480 | - | putative rRNA methylase | - | NP_417027 | yfhQ | predicted methyltransferase | GCO | 97.10% | 56.83% |
| NP_461481 | suhB | inositol monophosphatase | - | NP_417028 | suhB | inositol monophosphatase | GCO | 97.37% | 54.97% |
| NP_461482 | - | putative hydrolase | - | NP_417029 | yfhR | predicted peptidase | GCO | 66.89% | 51.99% |
| NP_461483 | asrA | anaerobic sulfide reductase | - |  |  |  | NO HOMOLOG |  | 52.68% |
| NP_461484 | asrB | anaerobic sulfite reductase subunit B | - |  |  |  | NO HOMOLOG |  | 55.55% |
| NP_461485 | asrC | anaerobic sulfide reductase | - |  |  |  | NO HOMOLOG |  | 55.91% |
| NP_461486 | - | putative inner membrane protein | - |  |  |  | NO HOMOLOG |  | 58.86% |
| NP_461487 | - | hypothetical protein | - |  |  |  | NO HOMOLOG |  | 52.11% |
| NP_461488 | csiE | stationary phase-inducible protein | - | NP_417030 | csiE | stationary phase inducible protein | GCO | 71.76% | 54.92% |
| NP_461489 | hcaT | putative transport protein | - | NP_417031 | hcaT | predicted 3-phenylpropionic transporter | GCO | 82.97% | 58.24% |
| NP_461490 | glyA | serine hydroxymethyltransferase | - | NP_417046 | glyA | serine hydroxymethyltransferase | GCO | 89.20% | 56.53% |
| NP_461491 | hmpA | dihydropteridine reductase 2/nitric oxide dioxygenase | - | NP_417047 | hmp | fused nitric oxide dioxygenase/dihydropteridine reductase 2 | GCO | 90.90% | 56.17% |
| NP_461492 | cadC | transcriptional activator | - | NP_418557 | cadC | DNA-binding transcriptional activator | GCO | 57.55% | 47.63% |
| NP_461493 | cadB | lysine/cadaverine transport protein | - | NP_418556 | cadB | predicted lysine/cadaverine transporter | GCO | 90.22% | 53.67% |
| NP_461494 | cadA | lysine decarboxylase 1 | - | NP_418555 | cadA | lysine decarboxylase 1 | GCO | 91.31% | 49.65% |
| NP_461495 | yjdL | putative di-/tripeptide transport protein | - | NP_418554 | yjdL | predicted transporter | GCO | 77.35% | 56.53% |
| NP_461496 | glnB | regulatory protein P-II | - | NP_417048 | glnB | regulatory protein P-II for glutamine synthetase | GCO | 85.71% | 49.85% |
| NP_461497 | yfhA | putative transcriptional regulator | - | NP_417049 | yfhA | predicted DNA-binding response regulator in two-component system | GCO | 95.04% | 55.45% |
| NP_461498 | yfhG | putative transcriptional regulator | - | NP_417050 | yfhG | hypothetical protein | GCO | 56.68% | 57.77% |
| NP_461499 | yfhK | putative sensor kinase | - | NP_417051 | yfhK | predicted sensory kinase in two-component system | GCO | 81.05% | 55.16% |
| NP_461500 | purG | phosphoribosylformylglycinamidine synthase | - | YP_026170 | purL | phosphoribosylformylglycinamidine synthase | GCO | 92.58% | 59.23% |
| NP_461501 | - | putative periplasmic protein | - |  |  |  | NO HOMOLOG |  | 46.15% |
| NP_461502 | yfhD | putative periplasmic amino acid-binding protein | - | NP_417053 | yfhD | predicted transglycosylase | GCO | 77.23% | 51.77% |
| NP_461503 | yfhC | putative cytosine/adenosine deaminase | - | NP_417054 | tadA | tRNA-specific adenosine deaminase | GCO | 89.59% | 56.70% |
| NP_461504 | yfhB | putative phosphoserine phosphatase | - | NP_417055 | yfhB | hypothetical protein | GCO | 85.30% | 54.55% |
| NP_461505 | - | putative phosphotransferase system IIB component | - | NP_416924 | murP | N-acetylmuramic acid phosphotransfer permease | GCO | 34.11% | 55.94% |
| NP_461506 | - | hypothetical protein | - | NP_416923 | yfeU | N-acetylmuramic acid-6-phosphate etherase | GCO | 51.68% | 57.04% |
| NP_461507 | yfhH | putative transport protein | - | NP_417056 | yfhH | predicted DNA-binding transcriptional regulator | nGCO | 79.07% | 57.24% |
| NP_461508 | - | 2-dehydropantoate 2-reductase | + | NP_414959 | panE | 2-dehydropantoate 2-reductase | nGCO | 25.40% | 48.91% |
| NP_461509 | - | putative permease | + | NP_418003 | yhjX | predicted transporter | nGCO | 26.61% | 46.44% |
| NP_461510 | - | putative transcriptional regulator | + | NP_414872 | cynR | DNA-binding transcriptional dual regulator | nGCO | 30.08% | 42.93% |
| NP_461511 | yfhL | putative ferredoxin | - | NP_417057 | yfhL | predicted 4Fe-4S cluster-containing protein | GCO | 94.18% | 47.12% |
| NP_461512 | acpS | 4'-phosphopantetheinyl transferase | - | NP_417058 | acpS | 4'-phosphopantetheinyl transferase | GCO | 94.44% | 56.69% |
| NP_461513 | pdxJ | pyridoxal phosphate biosynthetic protein | - | NP_417059 | pdxJ | pyridoxal phosphate biosynthetic protein | GCO | 88.47% | 57.51% |
| NP_461514 | recO | DNA repair protein RecO | - | NP_417060 | recO | DNA repair protein RecO | GCO | 94.56% | 54.73% |
| NP_461515 | era | GTP-binding protein Era | - | NP_417061 | era | GTP-binding protein Era | GCO | 97.00% | 51.76% |
| NP_461516 | rncS | ribonuclease III | - | NP_417062 | rnc | ribonuclease III | GCO | 97.12% | 52.86% |
| NP_461517 | lepB | signal peptidase I | - | NP_417063 | lepB | leader peptidase (signal peptidase I) | GCO | 90.74% | 53.12% |
| NP_461518 | lepA | GTP-binding protein LepA | - | NP_417064 | lepA | GTP-binding protein LepA | GCO | 90.98% | 52.88% |
| NP_461519 | gogB | leucine-rich repeat protein | - |  |  |  | NO HOMOLOG |  | 32.93% |
| NP_461520 | - | transposase-like protein | + |  |  |  | NO HOMOLOG |  | 39.92% |
| NP_461521 | - | PagK-like protein | - |  |  |  | NO HOMOLOG |  | 35.52% |
| NP_461522 | - | phage tail assembly-like protein | - | NP_415891 | tfaR | Rac prophage; predicted tail fiber assembly protein | nGCO | 59.16% | 47.84% |
| NP_461523 | - | phage tail assembly-like protein | - |  |  |  | NO HOMOLOG |  | 48.24% |
| NP_461524 | - | tail fiber-like protein | - |  |  |  | NO HOMOLOG |  | 57.90% |
| NP_461525 | - | host specificity protein-J-like | - |  |  |  | NO HOMOLOG |  | 57.02% |
| NP_461526 | - | tail assembly protein I-like | - |  |  |  | NO HOMOLOG |  | 60.64% |
| NP_461527 | - | tail assembly protein K-like | - |  |  |  | NO HOMOLOG |  | 58.33% |
| NP_461528 | - | phage tail component L-like protein | - |  |  |  | NO HOMOLOG |  | 52.93% |
| NP_461529 | - | phage tail component M-like protein | - |  |  |  | NO HOMOLOG |  | 48.48% |
| NP_461530 | - | phage tail component H-like protein | - |  |  |  | NO HOMOLOG |  | 56.42% |
| NP_461531 | - | minor tail-like protein | - |  |  |  | NO HOMOLOG |  | 55.16% |
| NP_461532 | - | minor tail-like protein | - |  |  |  | NO HOMOLOG |  | 53.28% |
| NP_461533 | - | major tail-like protein | - |  |  |  | NO HOMOLOG |  | 56.09% |
| NP_461534 | - | hypothetical protein | - |  |  |  | NO HOMOLOG |  | 50.49% |
| NP_461535 | - | putative virulence protein | - | NP_415949 | ydcM | predicted transposase | nGCO | 32.74% | 53.71% |
| NP_461536 | - | minor tail protein Z-like | - |  |  |  | NO HOMOLOG |  | 52.33% |
| NP_461537 | - | minor capsid protein FII | - |  |  |  | NO HOMOLOG |  | 56.51% |
| NP_461538 | - | DNA packaging-like protein | - |  |  |  | NO HOMOLOG |  | 54.72% |
| NP_461539 | - | phage head-like protein | - |  |  |  | NO HOMOLOG |  | 53.74% |
| NP_461540 | - | phage head-like protein | - |  |  |  | NO HOMOLOG |  | 58.90% |
| NP_461541 | - | head-tail preconnector-like protein | - |  |  |  | NO HOMOLOG |  | 56.57% |
| NP_461542 | - | head-tail preconnector-like protein | - |  |  |  | NO HOMOLOG |  | 56.60% |
| NP_461543 | - | head-to-tail joining-like protein | - |  |  |  | NO HOMOLOG |  | 57.84% |
| NP_461544 | - | terminase-like large protein | - |  |  |  | NO HOMOLOG |  | 51.34% |
| NP_461545 | - | DNA packaging-like protein | - | NP_416066 | nohA | Qin prophage; predicted packaging protein | nGCO | 58.02% | 52.12% |
| NP_461546 | - | hypothetical protein | - | NP_415696 | ycgK | hypothetical protein | nGCO | 80.45% | 41.79% |
| NP_461547 | - | endopeptidase-like protein | - | NP_415088 | rzpD | DLP12 prophage; predicted murein endopeptidase | GCO | 43.79% | 51.89% |
| NP_461548 | - | morphogenesis-like protein | - | NP_415087 | ybcS | DLP12 prophage; predicted lysozyme | GCO | 35.04% | 52.75% |
| NP_461549 | - | hypothetical protein | - |  |  |  | NO HOMOLOG |  | 51.81% |
| NP_461550 | - | hypothetical protein | - |  |  |  | NO HOMOLOG |  | 42.64% |
| NP_461551 | - | antirepressor-like protein | - |  |  |  | NO HOMOLOG |  | 45.92% |
| NP_461552 | - | antiterminator-like protein | - |  |  |  | NO HOMOLOG |  | 51.32% |
| NP_461553 | - | hypothetical protein | - | YP_588439 | ylcG | DLP12 prophage; predicted protein | nGCO | 63.63% | 51.06% |
| NP_461554 | - | hypothetical protein | - |  |  |  | NO HOMOLOG |  | 58.16% |
| NP_461555 | - | hypothetical protein | - |  |  |  | NO HOMOLOG |  | 53.23% |
| NP_461556 | - | hypothetical protein | - | NP_415579 | dinI | DNA damage-inducible protein I | nGCO | 47.43% | 46.15% |
| NP_461557 | - | hypothetical protein | - |  |  |  | NO HOMOLOG |  | 35.37% |
| NP_461558 | - | hypothetical protein | - |  |  |  | NO HOMOLOG |  | 54.48% |
| NP_461559 | - | hypothetical protein | - |  |  |  | NO HOMOLOG |  | 54.31% |
| NP_461560 | - | DNA replication protein DnaC | - | NP_415878 | ydaV | Rac prophage; predicted DNA replication protein | nGCO | 49.39% | 52.53% |
| NP_461561 | - | replication protein 15-like | - |  |  |  | NO HOMOLOG |  | 46.74% |
| NP_461562 | - | cI-like protein | - |  |  |  | NO HOMOLOG |  | 52.80% |
| NP_461563 | - | probable regulatory protein | - | NP_416088 | dicA | Qin prophage; predicted regulator for DicB | nGCO | 32.98% | 46.95% |
| NP_461564 | - | hypothetical protein | - |  |  |  | NO HOMOLOG |  | 48.65% |
| NP_461565 | - | hypothetical protein | + | YP_588451 | ydaE | Rac prophage; conserved protein | nGCO | 40% | 45.91% |
| NP_461566 | - | hypothetical protein | - |  |  |  | NO HOMOLOG |  | 39.03% |
| NP_461567 | - | exodeoxyribonuclease VIII-like protein | - |  |  |  | NO HOMOLOG |  | 53.08% |
| NP_461568 | - | enterohemolysin 1-like protein | - |  |  |  | NO HOMOLOG |  | 51.71% |
| NP_461569 | - | putative cytoplasmic protein | - |  |  |  | NO HOMOLOG |  | 52.08% |
| NP_461570 | - | excisionase-like protein | - |  |  |  | NO HOMOLOG |  | 50.52% |
| NP_461571 | - | integrase-like protein | - | NP_418691 | intB | KpLE2 phage-like element; predicted integrase | nGCO | 24.40% | 50.24% |
| NP_461572 | rseC | sigma E regulator | - | NP_417065 | rseC | RseC protein involved in reduction of the SoxR iron-sulfur cluster | GCO | 62.89% | 58.12% |
| NP_461573 | rseB | periplasmic negative regulator of sigmaE | - | NP_417066 | rseB | periplasmic negative regulator of sigmaE | GCO | 84.59% | 55.17% |
| NP_461574 | rseA | anti sigma-E factor | - | NP_417067 | rseA | anti-sigma factor | GCO | 79.62% | 54.22% |
| NP_461575 | rpoE | RNA polymerase sigma-70 factor | - | NP_417068 | rpoE | RNA polymerase, sigma 24 (sigma E) factor | GCO | 99.47% | 50.86% |
| NP_461576 | nadB | L-aspartate oxidase | - | NP_417069 | nadB | L-aspartate oxidase | GCO | 86.66% | 55.14% |
| NP_461577 | yfiC | putative transferase | - | NP_417070 | yfiC | predicted S-adenosyl-L-methionine-dependent methyltransferase | GCO | 84.48% | 53.65% |
| NP_461578 | srmB | ATP-dependent RNA helicase | - | NP_417071 | srmB | ATP-dependent RNA helicase | GCO | 80.40% | 54.30% |
| NP_461579 | yfiE | putative transcriptional regulator | - | NP_417072 | yfiE | predicted DNA-binding transcriptional regulator | GCO | 78.27% | 51.33% |
| NP_461580 | yfiK | putative transport protein | - | NP_417073 | yfiK | neutral amino-acid efflux system | GCO | 84.10% | 51.36% |
| NP_461581 | yfiD | putative formate acetyltransferase | - | NP_417074 | yfiD | pyruvate formate lyase subunit | GCO | 96.06% | 49.73% |
| NP_461582 | ung | uracil-DNA glycosylase | - | NP_417075 | ung | uracil-DNA glycosylase | GCO | 90.82% | 55.07% |
| NP_461583 | yfiF | putative tRNA/rRNA methyltransferase | - | NP_417076 | yfiF | predicted methyltransferase | GCO | 87.53% | 56.93% |
| NP_461584 | trxC | thioredoxin 2 | - | NP_417077 | trxC | thioredoxin 2 | GCO | 88.48% | 53.57% |
| NP_461585 | yfiP | putative cytoplasmic protein | - | NP_417078 | yfiP | hypothetical protein | GCO | 88.05% | 54.91% |
| NP_461586 | yfiQ | putative acetyl-CoA synthetase | - | NP_417079 | yfiQ | fused predicted acyl-CoA synthetase: NAD(P)-binding subunit/ATP-binding subunit | GCO | 90.40% | 55.84% |
| NP_461587 | pssA | phosphatidylserine synthase | - | NP_417080 | pssA | phosphatidylserine synthase | GCO | 93.50% | 49.85% |
| NP_461588 | yfiM | putative outer membrane lipoprotein | - | NP_417081 | yfiM | hypothetical protein | GCO | 80% | 55.86% |
| NP_461589 | kgtP | alpha-ketoglutarate permease | - | NP_417082 | kgtP | alpha-ketoglutarate transporter | GCO | 83.69% | 51.76% |
| NP_461590 | - | putative cytoplasmic protein | - |  |  |  | NO HOMOLOG |  | 49.56% |
| NP_461591 | clpB | ATP-dependent protease | - | NP_417083 | clpB | protein disaggregation chaperone | GCO | 92.41% | 53.30% |
| NP_461592 | yfiH | putative inner membrane protein | - | NP_417084 | yfiH | hypothetical protein | GCO | 87.65% | 54.23% |
| NP_461593 | rluD | ribosomal large subunit pseudouridine synthase D | - | NP_417085 | rluD | 23S rRNA pseudouridine synthase | GCO | 91.41% | 53.21% |
| NP_461594 | yfiO | putative lipoprotein | - | NP_417086 | yfiO | predicted lipoprotein | GCO | 96.32% | 52.16% |
| NP_461595 | yfiA | ribosome stabilization factor | - | NP_417088 | yfiA | cold shock protein associated with 30S ribosomal subunit | GCO | 91.96% | 47.19% |
| NP_461596 | - | hypothetical protein | - |  |  |  | NO HOMOLOG |  | 41.66% |
| NP_461597 | pheA | chorismate mutase P/prephenate dehydratase | - | NP_417090 | pheA | fused chorismate mutase P/prephenate dehydratase | nGCO | 87.27% | 53.74% |
| NP_461598 | - | putative cytoplasmic protein | - |  |  |  | NO HOMOLOG |  | 53.90% |
| NP_461599 | tyrA | chorismate mutase T/prephenate dehydrogenase | - | NP_417091 | tyrA | fused chorismate mutase T/prephenate dehydrogenase | GCO | 91.66% | 55.16% |
| NP_461600 | aroF | 3-deoxy-7-phosphoheptulonate synthase | + | NP_417092 | aroF | 3-deoxy-D-arabino-heptulosonate-7-phosphate synthase, tyrosine-repressible | GCO | 96.34% | 53.03% |
| NP_461601 | yfiR | putative periplasmic protein | - | NP_417094 | yfiR | hypothetical protein | GCO | 62.13% | 46.24% |
| NP_461602 | yfiN | putative diguanylate cyclase/phosphodiesterase | - | NP_417095 | yfiN | predicted diguanylate cyclase | GCO | 75.24% | 50.45% |
| NP_461603 | rplS | 50S ribosomal protein L19 | - | NP_417097 | rplS | 50S ribosomal protein L19 | GCO | 97.39% | 48.56% |
| NP_461604 | trmD | tRNA (guanine-N(1)-)-methyltransferase | - | NP_417098 | trmD | tRNA (guanine-N(1)-)-methyltransferase | GCO | 92.15% | 54.03% |
| NP_461605 | rimM | 16S rRNA-processing protein | - | NP_417099 | rimM | 16S rRNA-processing protein | GCO | 93.95% | 50.09% |
| NP_461606 | rpsP | 30S ribosomal protein S16 | - | NP_417100 | rpsP | 30S ribosomal protein S16 | GCO | 97.56% | 52.20% |
| NP_461607 | ffh | 4.5S-RNP protein | - | NP_417101 | ffh | Signal Recognition Particle (SRP) component with 4.5S RNA (ffs) | GCO | 93.59% | 54.99% |
| NP_461608 | corE | hypothetical protein | - | NP_417102 | ypjD | predicted inner membrane protein | GCO | 77.56% | 52.02% |
| NP_461609 | yfjD | hypothetical protein | - | YP_026171 | yfjD | predicted inner membrane protein | GCO | 92.21% | 52.81% |
| NP_461610 | - | putative cytoplasmic protein | - |  |  |  | NO HOMOLOG |  | 43.72% |
| NP_461611 | grpE | heat shock protein | - | NP_417104 | grpE | heat shock protein | GCO | 92.34% | 53.13% |
| NP_461613 | ppnK | inorganic polyphosphate/ATP-NAD kinase | - | NP_417105 | yfjB | inorganic polyphosphate/ATP-NAD kinase | GCO | 96.23% | 50.96% |
| NP_461614 | recN | recombination/DNA repair protein | - | YP_026172 | recN | recombination and repair protein | GCO | 83.36% | 54.81% |
| NP_461615 | smpA | small membrane protein A | - | NP_417107 | smpA | small membrane lipoprotein | GCO | 80.18% | 51.62% |
| NP_461616 | yfjF | hypothetical protein | - | NP_417108 | yfjF | hypothetical protein | GCO | 87.50% | 53.60% |
| NP_461617 | yfjG | putative oligoketide cyclase | - | NP_417109 | yfjG | hypothetical protein | GCO | 89.17% | 49.68% |
| NP_461618 | smpB | SsrA-binding protein | - | NP_417110 | smpB | SsrA-binding protein | GCO | 94.37% | 49.48% |
| NP_461619 | - | putative outer membrane efflux protein | - | NP_417507 | tolC | outer membrane channel precursor protein | nGCO | 20.69% | 56.87% |
| NP_461620 | - | putative ABC transporter transmembrane region | - | NP_415407 | cydD | fused cysteine transporter subunits of ABC superfamily: membrane component/ATP-binding component | nGCO | 24.15% | 56.85% |
| NP_461621 | - | putative HlyD family secretion protein | - |  |  |  | NO HOMOLOG |  | 53.86% |
| NP_461622 | - | late control-like protein | - | NP_416586 | ogrK | DNA-binding transcriptional regulator prophage P2 remnant | nGCO | 52.23% | 44.29% |
| NP_461623 | - | late control-like protein | - |  |  |  | NO HOMOLOG |  | 53.67% |
| NP_461624 | - | putative phage tail protein | - |  |  |  | NO HOMOLOG |  | 53.49% |
| NP_461625 | - | phage tail-like protein | - |  |  |  | NO HOMOLOG |  | 57.83% |
| NP_461626 | - | gpE-like protein | - |  |  |  | NO HOMOLOG |  | 58.33% |
| NP_461627 | - | putative phage tail-like protein | - |  |  |  | NO HOMOLOG |  | 55.77% |
| NP_461628 | - | phage tail fiber-like protein | - |  |  |  | NO HOMOLOG |  | 56.00% |
| NP_461629 | - | phage tail sheath-like protein | - |  |  |  | NO HOMOLOG |  | 57.97% |
| NP_461630 | - | DNA invertase-like protein | - | NP_415676 | pin | e14 prophage; site-specific DNA recombinase | nGCO | 80% | 50.35% |
| NP_461631 | - | hypothetical protein | - |  |  |  | NO HOMOLOG |  | 45.64% |
| NP_461632 | - | tail fibre assembly-like protein | - | NP_415093 | tfaD | DLP12 prophage; predicted tail fiber assembly protein (pseudogene) | nGCO | 38.21% | 48.77% |
| NP_461633 | - | hypothetical protein | - |  |  |  | NO HOMOLOG |  | 42.88% |
| NP_461634 | - | phage tail-like protein | - |  |  |  | NO HOMOLOG |  | 52.44% |
| NP_461635 | - | phage tail-like protein | - |  |  |  | NO HOMOLOG |  | 56.10% |
| NP_461636 | - | phage tail-like protein | - |  |  |  | NO HOMOLOG |  | 58.85% |
| NP_461637 | - | base plate tail-like protein | - |  |  |  | NO HOMOLOG |  | 56.94% |
| NP_461638 | - | phage baseplate assembly-like protein | - |  |  |  | NO HOMOLOG |  | 58.37% |
| NP_461639 | - | phage tail-like protein | - |  |  |  | NO HOMOLOG |  | 63.53% |
| NP_461640 | - | phage tail-like protein | - |  |  |  | NO HOMOLOG |  | 55.09% |
| NP_461641 | - | hypothetical protein | - |  |  |  | NO HOMOLOG |  | 60.70% |
| NP_461642 | - | lysis-like protein | - |  |  |  | NO HOMOLOG |  | 60.83% |
| NP_461643 | - | probable prophage lysozyme | - | NP_415087 | ybcS | DLP12 prophage; predicted lysozyme | nGCO | 34.19% | 60.37% |
| NP_461644 | - | phage-holin-like protein | - |  |  |  | NO HOMOLOG |  | 50.92% |
| NP_461645 | - | phage tail-like protein | - |  |  |  | NO HOMOLOG |  | 60.78% |
| NP_461646 | - | head completion-like protein | - |  |  |  | NO HOMOLOG |  | 59.56% |
| NP_461647 | - | terminase-like protein | - |  |  |  | NO HOMOLOG |  | 61.77% |
| NP_461648 | - | major capsid-like protein | - |  |  |  | NO HOMOLOG |  | 54.10% |
| NP_461649 | - | capsid scaffold-like protein | - |  |  |  | NO HOMOLOG |  | 58.27% |
| NP_461650 | - | terminase-like protein | - |  |  |  | NO HOMOLOG |  | 56.87% |
| NP_461651 | - | portal vertex-like protein | - |  |  |  | NO HOMOLOG |  | 52.92% |
| NP_461652 | - | hypothetical protein | - |  |  |  | NO HOMOLOG |  | 36.58% |
| NP_461653 | - | hypothetical protein | - |  |  |  | NO HOMOLOG |  | 39.89% |
| NP_461654 | - | hypothetical protein | - |  |  |  | NO HOMOLOG |  | 45.09% |
| NP_461655 | - | hypothetical protein | - |  |  |  | NO HOMOLOG |  | 44.44% |
| NP_461656 | - | hypothetical protein | - |  |  |  | NO HOMOLOG |  | 56.29% |
| NP_461657 | - | DNA adenine methylase-like protein | - | NP_417846 | dam | DNA adenine methylase | nGCO | 46.92% | 46.15% |
| NP_461658 | - | hypothetical protein | - |  |  |  | NO HOMOLOG |  | 57.89% |
| NP_461659 | - | hypothetical protein | - |  |  |  | NO HOMOLOG |  | 53.41% |
| NP_461660 | - | hypothetical protein | - |  |  |  | NO HOMOLOG |  | 44.44% |
| NP_461661 | - | hypothetical protein | - |  |  |  | NO HOMOLOG |  | 46.18% |
| NP_461662 | - | hypothetical protein | - |  |  |  | NO HOMOLOG |  | 47.43% |
| NP_461663 | - | hypothetical protein | - |  |  |  | NO HOMOLOG |  | 53.72% |
| NP_461664 | - | hypothetical protein | - |  |  |  | NO HOMOLOG |  | 45.41% |
| NP_461665 | - | hypothetical protein | - |  |  |  | NO HOMOLOG |  | 42.96% |
| NP_461666 | - | phage tail-like protein | - | NP_415069 | intD | DLP12 prophage; predicted integrase | nGCO | 24.93% | 49.70% |
| NP_461667 | - | integrase-like protein | - | NP_417111 | intA | CP4-57 prophage; integrase | nGCO | 46.62% | 56.52% |
| NP_945167 | - | hypothetical protein | - |  |  |  | NO HOMOLOG |  | 57.14% |
| NP_461668 | - | putative periplasmic protein | - |  |  |  | NO HOMOLOG |  | 55.55% |
| NP_461669 | - | putative cytoplasmic protein | + |  |  |  | NO HOMOLOG |  | 41.62% |
| NP_461670 | - | putative cytoplasmic protein | + |  |  |  | NO HOMOLOG |  | 33.69% |
| NP_461671 | - | putative cytoplasmic protein | - |  |  |  | NO HOMOLOG |  | 55.55% |
| NP_461672 | - | putative inner membrane protein | - |  |  |  | NO HOMOLOG |  | 63.23% |
| NP_461673 | - | putative ATPase | - |  |  |  | NO HOMOLOG |  | 27.88% |
| NP_461674 | - | putative cytoplasmic protein | - |  |  |  | NO HOMOLOG |  | 31.13% |
| NP_461675 | - | putative transcriptional regulator | - |  |  |  | NO HOMOLOG |  | 46.21% |
| NP_461676 | - | putative cytoplasmic protein | - | NP_417655 | sfsB | DNA-binding transcriptional activator of maltose metabolism | nGCO | 65.07% | 56.84% |
| NP_461677 | - | putative PTS system glucitol/sorbitol-specific enzyme II | - | YP_026180 | srlA | glucitol/sorbitol-specific enzyme IIC component of PTS | nGCO | 40.25% | 59.57% |
| NP_461678 | - | putative glucitol-specific PTS enzyme III | - | NP_417184 | srlB | glucitol/sorbitol-specific enzyme IIA component of PTS | GCO | 41.12% | 59.73% |
| NP_461679 | - | putative glucitol-specific PTS enzyme III | - | YP_026181 | srlE | glucitol/sorbitol-specific enzyme IIB component of PTS | GCO | 44.89% | 62.79% |
| NP_461680 | - | putative dehydrogenase | - | NP_418700 | yjhC | KpLE2 phage-like element; predicted oxidoreductase | nGCO | 23.85% | 59.66% |
| NP_461681 | - | putative hexulose 6 phosphate synthase | + |  |  |  | NO HOMOLOG |  | 36.74% |
| NP_461682 | - | putative hexulose 6 phosphate synthase | - | NP_418617 | ulaD | 3-keto-L-gulonate 6-phosphate decarboxylase | nGCO | 32.53% | 53.93% |
| NP_461683 | - | putative sugar phosphate aminotransferase | - |  |  |  | NO HOMOLOG |  | 57.80% |
| NP_461684 | - | putative cytoplasmic protein | - |  |  |  | NO HOMOLOG |  | 55.39% |
| NP_461685 | - | putative phosphotransferase system IIC component | - | NP_415619 | ptsG | fused glucose-specific PTS enzymes: IIB component/IIC component | nGCO | 42.56% | 57.52% |
| NP_461686 | - | putative dipeptide/oligopeptide/nickel ABC-type transport system periplasmic component | - | NP_414611 | sgrR | DNA-binding transcriptional regulator | nGCO | 26.58% | 57.02% |
| NP_461687 | - | putative integrase | - |  |  |  | NO HOMOLOG |  | 57.42% |
| NP_461688 | - | putative inner membrane protein | - |  |  |  | NO HOMOLOG |  | 32.90% |
| NP_461689 | - | putative inner membrane protein | - |  |  |  | NO HOMOLOG |  | 30.99% |
| NP_461692 | - | putative transposase | - |  |  |  | NO HOMOLOG |  | 47.19% |
| NP_461693 | - | putative cytoplasmic protein | - |  |  |  | NO HOMOLOG |  | 34.89% |
| NP_461694 | - | putative DNA/RNA helicase | - |  |  |  | NO HOMOLOG |  | 34.19% |
| NP_461695 | - | putative transposase | - | NP_416592 | insE-5 | IS3 element protein InsE | GCO | 85.85% | 53.33% |
| NP_461696 | - | putative transposase | - | NP_416593 | insF-5 | IS3 element protein InsF | GCO | 81.25% | 53.97% |
| NP_461697 | fljA | phase-1 flagellin repressor | - |  |  |  | NO HOMOLOG |  | 40.92% |
| NP_461698 | fljB | flagellar biosynthesis protein | - | NP_416433 | fliC | flagellin | nGCO | 52.90% | 50.69% |
| NP_461699 | hin | DNA-invertase Hin | - | NP_415676 | pin | e14 prophage; site-specific DNA recombinase | nGCO | 59.77% | 46.24% |
| NP_461700 | iroB | putative glycosyl transferase | - |  |  |  | NO HOMOLOG |  | 56.89% |
| NP_461701 | iroC | putative ABC transporter protein | - |  |  |  | NO HOMOLOG |  | 59.03% |
| NP_461702 | iroD | enterochelin esterase=-like protein | - | NP_415117 | fes | enterobactin/ferric enterobactin esterase | nGCO | 29.30% | 62.08% |
| NP_461703 | iroE | putative hydrolase | - |  |  |  | NO HOMOLOG |  | 53.31% |
| NP_461704 | iroN | TonB-dependent siderophore receptor protein | - | NP_415116 | fepA | iron-enterobactin outer membrane transporter | nGCO | 51.72% | 50.93% |
| NP_461706 | pipB2 | secreted effector protein | + |  |  |  | NO HOMOLOG |  | 44.44% |
| NP_461707 | virK | virulence protein | + | NP_415398 | ybjX | hypothetical protein | nGCO | 39.08% | 44.51% |
| NP_461708 | mig-14 | putative transcriptional activator | + |  |  |  | NO HOMOLOG |  | 41.02% |
| NP_461709 | nixA | putative nickel transporter | - |  |  |  | NO HOMOLOG |  | 50.29% |
| NP_461710 | tctE | regulatory protein | - | NP_417498 | qseC | sensory histidine kinase in two-component regulatory system with QseB | GCO | 28.78% | 56.14% |
| NP_461711 | tctD | regulatory protein | - | NP_417497 | qseB | DNA-binding response regulator in two-component regulatory system with QseC | GCO | 38.81% | 54.96% |
| NP_461712 | - | tricarboxylic transport | - |  |  |  | NO HOMOLOG |  | 56.33% |
| NP_461713 | - | tricarboxylic transport | - |  |  |  | NO HOMOLOG |  | 57.93% |
| NP_461714 | - | tricarboxylic transport | - |  |  |  | NO HOMOLOG |  | 57.16% |
| NP_461715 | - | hypothetical protein | - | NP_417145 | ygaT | hypothetical protein | GCO | 83.38% | 53.79% |
| NP_461716 | ygaF | putative sarcosine oxidase-like protein | - | NP_417146 | ygaF | predicted enzyme | GCO | 85.30% | 59.18% |
| NP_461717 | gabD | succinate-semialdehyde dehydrogenase I | - | NP_417147 | gabD | succinate-semialdehyde dehydrogenase I, NADP-dependent | GCO | 90.45% | 57.76% |
| NP_461718 | gabT | 4-aminobutyrate aminotransferase | - | NP_417148 | gabT | 4-aminobutyrate aminotransferase | GCO | 85.44% | 57.94% |
| NP_461719 | gabP | gamma-aminobutyrate transport protein | - | NP_417149 | gabP | gamma-aminobutyrate transporter | GCO | 90.77% | 51.39% |
| NP_461720 | ygaE | putative transcriptional repressor | - | NP_417150 | csiR | DNA-binding transcriptional dual regulator | GCO | 87.96% | 56.78% |
| NP_461721 | ygaU | hypothetical protein | - | NP_417151 | ygaU | hypothetical protein | GCO | 93.95% | 44.22% |
| NP_461722 | yqaE | putative transport protein | - | NP_417152 | yqaE | predicted membrane protein | GCO | 94.11% | 42.76% |
| NP_461723 | - | putative regulatory protein | - | NP_417153 | ygaV | predicted DNA-binding transcriptional regulator | GCO | 88.60% | 51.33% |
| NP_461724 | ygaP | putative rhodanese-like sulfurtransferase | - | NP_417154 | ygaP | predicted inner membrane protein with hydrolase activity | GCO | 70.52% | 56.43% |
| NP_461725 | stpA | DNA-binding protein | + | NP_417155 | stpA | DNA binding protein, nucleoid-associated | GCO | 66.41% | 46.51% |
| NP_461726 | - | putative inner membrane protein | - | NP_417156 | ygaW | predicted inner membrane protein | GCO | 79.86% | 50.22% |
| NP_461727 | ygaC | putative cytoplasmic protein | - | NP_417157 | ygaC | hypothetical protein | GCO | 88.39% | 49.85% |
| NP_461728 | ygaM | putative inner membrane protein | - | NP_417158 | ygaM | hypothetical protein | GCO | 76.78% | 54.57% |
| NP_461729 | - | putative regulatory protein | - | NP_415956 | ydcR | fused predicted DNA-binding transcriptional regulator/predicted amino transferase | nGCO | 29.38% | 56.62% |
| NP_461730 | - | putative cytoplasmic protein | - | NP_416267 | ynjA | hypothetical protein | nGCO | 33.04% | 52.08% |
| NP_951053 | - | hypothetical protein | - |  |  |  | NO HOMOLOG |  | 54.80% |
| NP_461731 | nrdH | glutaredoxin-like protein | - | NP_417159 | nrdH | glutaredoxin-like protein | GCO | 85.18% | 55.28% |
| NP_461732 | nrdI | hypothetical protein | - | NP_417160 | nrdI | hypothetical protein | GCO | 85.29% | 56.93% |
| NP_461733 | nrdE | ribonucleotide-diphosphate reductase alpha subunit | - | NP_417161 | nrdE | ribonucleotide-diphosphate reductase alpha subunit | GCO | 89.49% | 54.21% |
| NP_461734 | nrdF | ribonucleotide-diphosphate reductase beta subunit | - | NP_417162 | nrdF | ribonucleotide-diphosphate reductase beta subunit | GCO | 87.77% | 49.58% |
| NP_461735 | proV | glycine/betaine/proline transport protein | + | NP_417163 | proV | glycine betaine transporter subunit | GCO | 95% | 50.45% |
| NP_461736 | proW | glycine/betaine/proline transport protein | + | NP_417164 | proW | glycine betaine transporter subunit | GCO | 79.66% | 59.53% |
| NP_461737 | proX | glycine/betaine/proline transport protein | - | NP_417165 | proX | glycine betaine transporter subunit | GCO | 83.38% | 53.41% |
| NP_461738 | - | putative inner membrane protein | - | YP_026179 | ygaY | predicted transporter (pseudogene) | nGCO | 69.62% | 58.98% |
| NP_461739 | emrR | emrAB operon transcriptional repressor | - | NP_417169 | mprA | DNA-binding transcriptional repressor of microcin B17 synthesis and multidrug efflux | GCO | 93.14% | 48.77% |
| NP_461740 | emrA | multidrug resistance secretion protein | - | NP_417170 | emrA | multidrug efflux system | GCO | 86.41% | 54.47% |
| NP_461741 | emrB | putative multidrug transport protein | - | NP_417171 | emrB | multidrug efflux system protein | GCO | 93.42% | 56.85% |
| NP_461742 | - | putative glycoporin | - |  |  |  | NO HOMOLOG |  | 47.48% |
| NP_461743 | luxS | S-ribosylhomocysteinase | - | NP_417172 | luxS | S-ribosylhomocysteinase | GCO | 94.15% | 53.48% |
| NP_461744 | gshA | glutamate--cysteine ligase | - | NP_417173 | gshA | glutamate--cysteine ligase | GCO | 91.31% | 52.15% |
| NP_461745 | yqaA | putative inner membrane protein | - | NP_417174 | yqaA | conserved inner membrane protein | GCO | 92.50% | 53.75% |
| NP_461746 | yqaB | putative phosphoglucomutase | - | NP_417175 | yqaB | predicted hydrolase | GCO | 87.76% | 56.43% |
| NP_461747 | csrA | carbon storage regulator | - | NP_417176 | csrA | carbon storage regulator | GCO | 100% | 51.07% |
| NP_461748 | alaS | alanyl-tRNA synthetase | - | NP_417177 | alaS | alanyl-tRNA synthetase | GCO | 89.49% | 54.92% |
| NP_461749 | recX | RecA regulator RecX | - | NP_417178 | recX | RecA regulator RecX | GCO | 87.34% | 51.69% |
| NP_461750 | recA | recombinase A | - | NP_417179 | recA | recombinase A | GCO | 93.76% | 54.70% |
| NP_461751 | ygaD | competence damage-inducible protein A | - | NP_417180 | ygaD | competence damage-inducible protein A | GCO | 92.72% | 58.83% |
| NP_461752 | mltB | membrane-bound lytic murein transglycosylase B | - | NP_417181 | mltB | membrane-bound lytic murein transglycosylase B | GCO | 85.31% | 55.83% |
| NP_461753 | srlA | glucitol/sorbitol-specific enzyme IIC component | - | YP_026180 | srlA | glucitol/sorbitol-specific enzyme IIC component of PTS | GCO | 86.63% | 50.53% |
| NP_461754 | srlE | glucitol/sorbitol-specific enzyme IIB component | - | YP_026181 | srlE | glucitol/sorbitol-specific enzyme IIB component of PTS | GCO | 83.28% | 58.64% |
| NP_461755 | slrB | glucitol/sorbitol-specific enzyme IIA component | - | NP_417184 | srlB | glucitol/sorbitol-specific enzyme IIA component of PTS | GCO | 82.20% | 57.57% |
| NP_461756 | srlD | sorbitol-6-phosphate 2-dehydrogenase | - | NP_417185 | srlD | 3-ketoacyl-(acyl-carrier-protein) reductase | GCO | 93.05% | 54.74% |
| NP_461757 | gutM | putative glucitol operon regulatory protein | - | NP_417186 | gutM | DNA-binding transcriptional activator of glucitol operon | GCO | 73.10% | 55% |
| NP_461758 | srlR | transcriptional repressor for glucitol operon | - | NP_417187 | srlR | DNA-bindng transcriptional repressor | GCO | 94.55% | 49.87% |
| NP_461759 | gutQ | putative sugar phosphate isomerase | - | NP_417188 | gutQ | predicted phosphosugar-binding protein | GCO | 88.16% | 57.03% |
| NP_461760 | ygaA | anaerobic nitric oxide reductase transcription regulator | - | NP_417189 | norR | anaerobic nitric oxide reductase transcription regulator | GCO | 83.03% | 59.36% |
| NP_461761 | - | anaerobic nitric oxide reductase flavorubredoxin | - | NP_417190 | norV | anaerobic nitric oxide reductase flavorubredoxin | GCO | 92.90% | 53.68% |
| NP_461762 | ygbD | nitric oxide reductase | - | NP_417191 | norW | nitric oxide reductase | GCO | 82.66% | 56.61% |
| NP_461763 | hypF | hydrogenase maturation protein | - | NP_417192 | hypF | carbamoyl phosphate phosphatase and maturation protein for [NiFe] hydrogenases | GCO | 76.20% | 60.41% |
| NP_461764 | hydN | electron transport protein | - | NP_417193 | hydN | formate dehydrogenase-H, [4Fe-4S] ferredoxin subunit | GCO | 95.95% | 56.59% |
| NP_461765 | - | hypothetical protein | - |  |  |  | NO HOMOLOG |  | 52.71% |
| NP_461766 | hycI | hydrogenase 3 large subunit C-terminal protease | - | NP_417197 | hycI | protease involved in processing C-terminal end of HycE | GCO | 92.25% | 56.26% |
| NP_461767 | hycH | hydrogenase 3 large subunit processing protein | - | NP_417198 | hycH | protein required for maturation of hydrogenase 3 | GCO | 91.17% | 55.96% |
| NP_461768 | hycG | hydrogenase | - | NP_417199 | hycG | hydrogenase 3 and formate hydrogenase complex, HycG subunit | GCO | 92.15% | 59.50% |
| NP_461769 | hycF | hydrogenase 4 Fe-S subunit | - | NP_417200 | hycF | formate hydrogenlyase complex iron-sulfur protein | GCO | 96.62% | 55.98% |
| NP_461770 | hycE | hydrogenase 3 large subunit | - | NP_417201 | hycE | hydrogenase 3, large subunit | GCO | 95.07% | 57.95% |
| NP_461771 | hycD | hydrogenase 3 membrane subunit | - | NP_417202 | hycD | hydrogenase 3, membrane subunit | GCO | 82.13% | 58.54% |
| NP_461772 | hycC | NADH dehydrogenase subunit N | - | NP_417203 | hycC | NADH dehydrogenase subunit N | GCO | 76.55% | 60.97% |
| NP_461773 | hycB | hydrogenase-3 iron-sulfur subunit | - | NP_417204 | hycB | hydrogenase 3, Fe-S subunit | GCO | 87.62% | 57.79% |
| NP_461774 | hycA | transcriptional repressor | - | NP_417205 | hycA | regulator of the transcriptional regulator FhlA | GCO | 88.81% | 50.86% |
| NP_461775 | hypA | hydrogenase nickel incorporation protein | - | NP_417206 | hypA | protein involved in nickel insertion into hydrogenases 3 | GCO | 86.95% | 48.17% |
| NP_461776 | hypB | hydrogenase-3 accessory protein | - | NP_417207 | hypB | GTP hydrolase involved in nickel liganding into hydrogenases | GCO | 85.86% | 57.38% |
| NP_461777 | hypC | putative hydrogenase formation protein | - | NP_417208 | hypC | protein required for maturation of hydrogenases 1 and 3 | GCO | 96.66% | 55.67% |
| NP_461778 | hypD | putative hydrogenase formation protein | - | NP_417209 | hypD | protein required for maturation of hydrogenases | GCO | 94.35% | 57.99% |
| NP_461779 | hypE | putative hydrogenase formation protein | - | NP_417210 | hypE | carbamoyl phosphate phosphatase, hydrogenase 3 maturation protein | GCO | 89.75% | 59.34% |
| NP_461780 | fhlA | formate hydrogen-lyase transcriptional activator | - | NP_417211 | fhlA | DNA-binding transcriptional activator | GCO | 90.44% | 54.93% |
| NP_461781 | ygbA | putative cytoplasmic protein | - | NP_417212 | ygbA | hypothetical protein | GCO | 86.84% | 53.91% |
| NP_461782 | sitA | putative periplasmic binding protein | - | NP_416371 | znuA | high-affinity zinc transporter periplasmic component | nGCO | 20.31% | 53.70% |
| NP_461783 | sitB | putative ATP-binding protein | - | NP_418707 | fecE | KpLE2 phage-like element; iron-dicitrate transporter subunit | nGCO | 31.64% | 54.50% |
| NP_461784 | sitC | putative permease | - | NP_416373 | znuB | high-affinity zinc transporter membrane component | nGCO | 24.68% | 54.81% |
| NP_461785 | sitD | putative permease | - | NP_416373 | znuB | high-affinity zinc transporter membrane component | nGCO | 22.22% | 50.53% |
| NP_461786 | avrA | secreted effector protein | - |  |  |  | NO HOMOLOG |  | 42.24% |
| NP_461787 | sprB | transcriptional regulator | - |  |  |  | NO HOMOLOG |  | 45.37% |
| NP_461788 | hilC | invasion regulatory protein | - |  |  |  | NO HOMOLOG |  | 37.04% |
| NP_461789 | orgC | putative cytoplasmic protein | - |  |  |  | NO HOMOLOG |  | 45.69% |
| NP_461790 | orgB | needle complex export protein | - |  |  |  | NO HOMOLOG |  | 45.08% |
| NP_461791 | orgA | needle complex assembly protein | - |  |  |  | NO HOMOLOG |  | 47.83% |
| NP_461792 | prgK | needle complex inner membrane lipoprotein | - |  |  |  | NO HOMOLOG |  | 45.71% |
| NP_461793 | prgJ | needle complex minor subunit | - |  |  |  | NO HOMOLOG |  | 47.38% |
| NP_461794 | prgI | needle complex major subunit | - |  |  |  | NO HOMOLOG |  | 46.09% |
| NP_461795 | prgH | needle complex inner membrane protein | - |  |  |  | NO HOMOLOG |  | 48.93% |
| NP_461796 | hilD | invasion protein regulatory protein | - |  |  |  | NO HOMOLOG |  | 40.96% |
| NP_461797 | hilA | invasion protein transcriptional activator | - | NP_417329 | ygeH | predictedtranscriptional regulator | nGCO | 29.03% | 43.14% |
| NP_461798 | iagB | invasion protein precursor | - | NP_417331 | pbl | predicted peptidoglycan-binding enzyme (pseudogene) | nGCO | 42.10% | 41.40% |
| NP_461799 | sptP | protein tyrosine phosphatase/GTPase activating protein | + |  |  |  | NO HOMOLOG |  | 44.11% |
| NP_461800 | sicP | secretion chaparone | + |  |  |  | NO HOMOLOG |  | 39.18% |
| NP_461802 | iacP | acyl carrier protein | - | NP_415612 | acpP | acyl carrier protein | nGCO | 38.70% | 37.75% |
| NP_461803 | sipA | secreted effector protein | - |  |  |  | NO HOMOLOG |  | 48.00% |
| NP_461804 | sipD | translocation machinery component | - |  |  |  | NO HOMOLOG |  | 47.28% |
| NP_461805 | sipC | translocation machinery component | - |  |  |  | NO HOMOLOG |  | 47.72% |
| NP_461806 | sipB | translocation machinery component | - |  |  |  | NO HOMOLOG |  | 52.30% |
| NP_461807 | sicA | secretion chaperone | - | NP_417328 | ygeG | predicted chaperone | nGCO | 37.14% | 42.57% |
| NP_461808 | spaS | type III secretion protein | - | NP_416394 | flhB | flagellar biosynthesis protein B | nGCO | 21.47% | 43.32% |
| NP_461809 | spaR | needle complex export protein | - | NP_416460 | fliR | flagellar biosynthesis protein R | nGCO | 19.62% | 49.36% |
| NP_461810 | spaQ | needle complex export protein | - |  |  |  | NO HOMOLOG |  | 46.74% |
| NP_461811 | spaP | needle complex export protein | - | NP_416458 | fliP | flagellar biosynthesis protein P | nGCO | 29.71% | 41.48% |
| NP_461812 | spaO | type III secretion protein | - |  |  |  | NO HOMOLOG |  | 52.74% |
| NP_461813 | invJ | needle length control protein | - |  |  |  | NO HOMOLOG |  | 48.46% |
| NP_461814 | invI | needle complex assembly protein | - |  |  |  | NO HOMOLOG |  | 43.46% |
| NP_461815 | invC | type III secretion system ATPase | - | NP_416451 | fliI | flagellum-specific ATP synthase | nGCO | 37.44% | 54.24% |
| NP_461816 | invB | secretion chaperone | - |  |  |  | NO HOMOLOG |  | 43.87% |
| NP_461817 | invA | needle complex export protein | - | NP_416393 | flhA | flagellar biosynthesis protein A | nGCO | 28.65% | 45.57% |
| NP_461818 | invE | invasion protein | - |  |  |  | NO HOMOLOG |  | 45.39% |
| NP_461819 | invG | outer membrane secretin precursor | - |  |  |  | NO HOMOLOG |  | 46.41% |
| NP_461820 | invF | invasion regulatory protein | - |  |  |  | NO HOMOLOG |  | 45.92% |
| NP_461821 | invH | needle complex outer membrane lipoprotein precursor | - |  |  |  | NO HOMOLOG |  | 42.11% |
| NP_461822 | - | putative cytoplasmic protein | - |  |  |  | NO HOMOLOG |  | 42.88% |
| NP_461823 | - | putative cytoplasmic protein | - |  |  |  | NO HOMOLOG |  | 37.06% |
| NP_461824 | - | putative cytoplasmic protein | - |  |  |  | NO HOMOLOG |  | 30.58% |
| NP_461825 | - | putative ABC-type transporter | - |  |  |  | NO HOMOLOG |  | 47.76% |
| NP_461826 | - | putative acetyltransferase | - |  |  |  | NO HOMOLOG |  | 47.34% |
| NP_461828 | pphB | serine/threonine protein phosphatase 2 | + | NP_417214 | pphB | serine/threonine-specific protein phosphatase 2 | nGCO | 60.09% | 41.55% |
| NP_461829 | - | putative cytoplasmic protein | - |  |  |  | NO HOMOLOG |  | 43.48% |
| NP_461830 | mutS | DNA mismatch repair protein | - | NP_417213 | mutS | DNA mismatch repair protein | nGCO | 90.57% | 57.04% |
| NP_461831 | - | putative cytoplasmic protein | - |  |  |  | NO HOMOLOG |  | 55.64% |
| NP_461832 | - | putative permease | - |  |  |  | NO HOMOLOG |  | 54.42% |
| NP_461833 | - | putative transcriptional regulator | - | NP_414744 | yafC | predicted DNA-binding transcriptional regulator | nGCO | 26.73% | 55.09% |
| NP_461834 | - | putative permease | - | NP_418716 | yjhF | KpLE2 phage-like element; predicted transporter | nGCO | 22.36% | 58.58% |
| NP_461835 | - | putative nucleoside-diphosphate-sugar epimerase | - |  |  |  | NO HOMOLOG |  | 56.15% |
| NP_461836 | ygbM | putative endonuclease | - | NP_417219 | ygbM | hypothetical protein | GCO | 68.99% | 57.27% |
| NP_461837 | ygbL | hypothetical protein | - | NP_417218 | ygbL | hypothetical protein | GCO | 71.09% | 56.33% |
| NP_461838 | ygbK | putative tRNA synthase | - | NP_417217 | ygbK | hypothetical protein | GCO | 79.42% | 59.46% |
| NP_461839 | ygbJ | 3-hydroxyisobutyrate dehydrogenase | - | NP_417216 | ygbJ | predicted dehydrogenase, with NAD(P)-binding Rossmann-fold domain | GCO | 70.23% | 59.95% |
| NP_461840 | ygbI | putative regulatory protein | - | NP_417215 | ygbI | predicted DNA-binding transcriptional regulator | GCO | 86.56% | 55.68% |
| NP_461841 | - | putative transcriptional regulator | - | NP_416159 | slyA | transcriptional regulator SlyA | nGCO | 27.35% | 52.59% |
| NP_461842 | - | putative flavoprotein | - | NP_416814 | ubiX | 3-octaprenyl-4-hydroxybenzoate carboxy-lyase | nGCO | 55.91% | 57.74% |
| NP_461843 | - | putative 3-polyprenyl-4-hydroxybenzoate decarboxylase | - | NP_418285 | ubiD | 3-octaprenyl-4-hydroxybenzoate decarboxylase | nGCO | 27.30% | 55.53% |
| NP_461844 | - | putative cytoplasmic protein | - |  |  |  | NO HOMOLOG |  | 52.74% |
| NP_461845 | rpoS | RNA polymerase sigma factor | - | NP_417221 | rpoS | RNA polymerase sigma factor | GCO | 99.09% | 52.46% |
| NP_461846 | nlpD | lipoprotein | - | NP_417222 | nlpD | predicted outer membrane lipoprotein | GCO | 75.52% | 52.02% |
| NP_461847 | pcm | protein-L-isoaspartate O-methyltransferase | - | NP_417223 | pcm | protein-L-isoaspartate O-methyltransferase | GCO | 94.23% | 56.61% |
| NP_461848 | surE | acid phosphatase | - | NP_417224 | surE | acid phosphatase | GCO | 91.69% | 56.16% |
| NP_461849 | ygbO | tRNA pseudouridine synthase D | - | NP_417225 | truD | tRNA pseudouridine synthase D | GCO | 89.11% | 53.71% |
| NP_461850 | ispF | 2-C-methyl-D-erythritol 2,4-cyclodiphosphate synthase | - | NP_417226 | ispF | 2-C-methyl-D-erythritol 2,4-cyclodiphosphate synthase | GCO | 84.90% | 57.29% |
| NP_461851 | ispD | 2-C-methyl-D-erythritol 4-phosphate cytidylyltransferase | - | NP_417227 | ispD | 2-C-methyl-D-erythritol 4-phosphate cytidylyltransferase | GCO | 91.45% | 57.52% |
| NP_461852 | ftsB | cell divison protein FtsB | - | NP_417228 | ftsB | cell divison protein FtsB | GCO | 90.47% | 54.16% |
| NP_461853 | ygbE | putative inner membrane protein | - | NP_417229 | ygbE | conserved inner membrane protein | GCO | 58.87% | 49.01% |
| NP_461854 | cysC | adenylylsulfate kinase | - | NP_417230 | cysC | adenylylsulfate kinase | GCO | 85.63% | 54.78% |
| NP_461855 | cysN | sulfate adenylyltransferase subunit 1 | - | NP_417231 | cysN | sulfate adenylyltransferase subunit 1 | GCO | 87.78% | 54.51% |
| NP_461856 | cysD | sulfate adenylyltransferase subunit 2 | - | NP_417232 | cysD | sulfate adenylyltransferase subunit 2 | GCO | 97.01% | 53.02% |
| NP_461857 | iap | aminopeptidase | - | NP_417233 | iap | aminopeptidase in alkaline phosphatase isozyme conversion | GCO | 85.17% | 52.05% |
| NP_461858 | ygbF | putative inner membrane protein | - | NP_417234 | ygbF | hypothetical protein | GCO | 84.04% | 49.15% |
| NP_461859 | - | putative cytoplasmic protein | - | NP_417235 | ygbT | hypothetical protein | GCO | 73.92% | 55.70% |
| NP_461860 | ygcH | putative cytoplasmic protein | - | NP_417236 | ygcH | hypothetical protein | GCO | 28.50% | 57.14% |
| NP_461861 | - | putative cytoplasmic protein | + | NP_417237 | ygcI | hypothetical protein | GCO | 26.70% | 57.96% |
| NP_461862 | yghJ | putative cytoplasmic protein | + | NP_417238 | ygcJ | hypothetical protein | GCO | 26.51% | 52.78% |
| NP_461863 | - | putative transposase | + |  |  |  | NO HOMOLOG |  | 48.30% |
| NP_461864 | - | putative cytoplasmic protein | - |  |  |  | NO HOMOLOG |  | 54.39% |
| NP_461865 | ygcB | putative helicase | + | NP_417241 | ygcB | conserved protein, member of DEAD box family | nGCO | 31.04% | 50.30% |
| NP_461866 | sopD | secreted effector protein | + |  |  |  | NO HOMOLOG |  | 40.67% |
| NP_461867 | cysH | phosphoadenosine phosphosulfate reductase | - | NP_417242 | cysH | phosphoadenosine phosphosulfate reductase | GCO | 93.85% | 56.46% |
| NP_461868 | cysI | sulfite reductase alpha subunit | - | NP_417243 | cysI | sulfite reductase, beta subunit, NAD(P)-binding, heme-binding | GCO | 91.22% | 57.26% |
| NP_461869 | cysJ | sulfite reductase beta subunit | - | NP_417244 | cysJ | sulfite reductase, alpha subunit, flavoprotein | GCO | 83.90% | 57.50% |
| NP_461870 | ptpS | putative 6-pyruvoyl tetrahydrobiopterin synthase | - | NP_417245 | ygcM | 6-pyruvoyl tetrahydrobiopterin synthase (PTPS) | GCO | 94.16% | 52.89% |
| NP_461871 | - | putative metal-dependent hydrolase | - |  |  |  | NO HOMOLOG |  | 51.37% |
| NP_461872 | ygcF | hypothetical protein | - | NP_417257 | ygcF | hypothetical protein | nGCO | 95.96% | 50.89% |
| NP_461873 | eno | phosphopyruvate hydratase | - | NP_417259 | eno | phosphopyruvate hydratase | GCO | 91.66% | 52.27% |
| NP_461874 | pyrG | CTP synthetase | - | NP_417260 | pyrG | CTP synthetase | GCO | 91.37% | 52.31% |
| NP_461875 | mazG | putative pyrophosphatase | - | NP_417261 | mazG | nucleoside triphosphate pyrophosphohydrolase | GCO | 87.78% | 53.05% |
| NP_945168 | - | hypothetical protein | - |  |  |  | NO HOMOLOG |  | 38.83% |
| NP_461876 | - | putative transcriptional regulator | - |  |  |  | NO HOMOLOG |  | 48.18% |
| NP_461877 | relA | (p)ppGpp synthetase I | - | NP_417264 | relA | (p)ppGpp synthetase I/GTP pyrophosphokinase | GCO | 94.07% | 55.57% |
| NP_461878 | rumA | 23S rRNA (uracil-5-)-methyltransferase | - | NP_417265 | rumA | 23S rRNA (uracil-5-)-methyltransferase | GCO | 81.71% | 54.39% |
| NP_461879 | barA | sensor histidine kinase | - | NP_417266 | barA | hybrid sensory histidine kinase, in two-component regulatory system with UvrY | GCO | 89.03% | 52.66% |
| NP_461880 | - | putative glycerate kinase 2 | - | NP_417593 | garK | glycerate kinase I | nGCO | 65.87% | 58.09% |
| NP_461881 | gudD | d-glucarate dehydratase | - | NP_417267 | gudD | (D)-glucarate dehydratase 1 | GCO | 97.08% | 55.48% |
| NP_461882 | ygcY | putative D-glucarate dehydratase | - | NP_417268 | gudX | predicted glucarate dehydratase | GCO | 91.47% | 56.59% |
| NP_461883 | gudT | putative D-glucarate permease | - | NP_417269 | gudP | predicted D-glucarate transporter | GCO | 96.66% | 52.24% |
| NP_461884 | - | flavodoxin | - | NP_417270 | yqcA | flavodoxin | GCO | 84.56% | 54% |
| NP_461885 | yqcB | putative pseudouridylate synthase | - | NP_417271 | yqcB | tRNA pseudouridine synthase | GCO | 85.49% | 58.36% |
| NP_461886 | yqcC | putative cytoplasmic protein | - | NP_417272 | yqcC | hypothetical protein | GCO | 80.73% | 57.27% |
| NP_461887 | syd | SecY interacting protein Syd | - | NP_417273 | syd | SecY interacting protein Syd | GCO | 84.53% | 52.01% |
| NP_461888 | yqcD | putative GTP cyclohydrolase I | - | NP_417274 | yqcD | hypothetical protein | GCO | 87.23% | 53.47% |
| NP_461889 | ygdH | putative nucleotide binding | - | NP_417275 | ygdH | hypothetical protein | GCO | 94.48% | 55.38% |
| NP_461890 | sdaC | putative serine transport protein | - | NP_417276 | sdaC | predicted serine transporter | GCO | 92.30% | 53.25% |
| NP_461891 | sdaB | L-serine dehydratase/L-threonine deaminase 2 | - | NP_417277 | sdaB | L-serine deaminase II | GCO | 88.79% | 56.28% |
| NP_461892 | xni | exonuclease IX | - | NP_417278 | exo | exonuclease IX | GCO | 87.50% | 55.26% |
| NP_461893 | fucO | L-1,2-propanediol oxidoreductase | + | NP_417279 | fucO | L-1,2-propanediol oxidoreductase | GCO | 88.48% | 56.04% |
| NP_461894 | fucA | L-fuculose phosphate aldolase | + | NP_417280 | fucA | L-fuculose phosphate aldolase | GCO | 91.62% | 52.62% |
| NP_461895 | fucI | L-fucose isomerase | + | NP_417282 | fucI | L-fucose isomerase | GCO | 92.55% | 57.60% |
| NP_461896 | fucK | L-fuculokinase | - | NP_417283 | fucK | L-fuculokinase | GCO | 84.74% | 58.70% |
| NP_461897 | fucU | putative L-fucose-binding protein | + | NP_417284 | fucU | L-fucose mutarotase | GCO | 91.42% | 54.37% |
| NP_461898 | fucR | fuc operon positive regulator | - | NP_417285 | fucR | DNA-binding transcriptional activator | GCO | 88.93% | 49.78% |
| NP_461899 | ygdE | putative SAM-dependent methyltransferase | - | NP_417286 | ygdE | predicted methyltransferase | GCO | 94.26% | 53.04% |
| NP_461900 | ygdD | hypothetical protein | - | NP_417287 | ygdD | conserved inner membrane protein | GCO | 90.07% | 56.81% |
| NP_461901 | gcvA | gcv operon regulator | - | NP_417288 | gcvA | DNA-binding transcriptional dual regulator | GCO | 98.36% | 50.98% |
| NP_461902 | ygdI | putative lipoprotein | - | NP_417289 | ygdI | hypothetical protein | GCO | 93.24% | 45.61% |
| NP_461903 | csdA | putative selenocysteine lyase | - | NP_417290 | csdA | cysteine sulfinate desulfinase | GCO | 85.53% | 59.86% |
| NP_461904 | ygdK | putative FeS center assembly protein | - | NP_417291 | ygdK | predicted Fe-S metabolism protein | GCO | 88.19% | 57.20% |
| NP_461905 | - | putative integral membrane protein | - | YP_026262 | rarD | predicted chloramphenical resistance permease | nGCO | 32.75% | 42.40% |
| NP_461906 | ygdL | putative enzyme | - | NP_417292 | ygdL | hypothetical protein | GCO | 93.63% | 56.00% |
| NP_461907 | mltA | membrane-bound lytic murein transglycosylase A | - | NP_417293 | mltA | membrane-bound lytic murein transglycosylase A | GCO | 92.05% | 53.91% |
| NP_461908 | amiC | N-acetylmuramoyl-L-alanine amidase | - | NP_417294 | amiC | N-acetylmuramoyl-L-alanine amidase | GCO | 92.30% | 53.66% |
| NP_461909 | argA | N-acetylglutamate synthase | - | NP_417295 | argA | N-acetylglutamate synthase | GCO | 93.22% | 52.85% |
| NP_461910 | recD | exonuclease V alpha chain | - | NP_417296 | recD | exonuclease V (RecBCD complex), alpha chain | GCO | 84.43% | 58.22% |
| NP_461911 | recB | exonuclease V beta chain | - | NP_417297 | recB | exonuclease V (RecBCD complex), beta subunit | GCO | 79.91% | 55.80% |
| NP_461912 | ptr | protease III | - | NP_417298 | ptr | protease III | GCO | 88.98% | 52.09% |
| NP_461913 | recC | exonuclease V subunit | - | NP_417299 | recC | exonuclease V (RecBCD complex), gamma chain | GCO | 86.36% | 53.79% |
| NP_461914 | ppdC | prepilin peptidase dependent protein C | - | NP_417300 | ppdC | hypothetical protein | GCO | 57.54% | 53.58% |
| NP_461915 | ygdB | putative periplasmic protein | - | NP_417301 | ygdB | hypothetical protein | GCO | 45.18% | 56.37% |
| NP_461916 | ppdB | prepilin peptidase-dependent protein B | - | NP_417302 | ppdB | hypothetical protein | GCO | 67.37% | 54.25% |
| NP_461917 | ppdA | prepilin peptidase-dependent protein A | - | NP_417303 | ppdA | hypothetical protein | GCO | 59.35% | 56.05% |
| NP_461918 | thyA | thymidylate synthase | - | NP_417304 | thyA | thymidylate synthase | GCO | 96.21% | 51.94% |
| NP_461919 | lgt | prolipoprotein diacylglyceryl transferase | - | NP_417305 | lgt | prolipoprotein diacylglyceryl transferase | GCO | 89.00% | 53.65% |
| NP_461920 | ptsP | transcriptional regulator | - | NP_417306 | ptsP | fused PTS enzyme: PEP-protein phosphotransferase (enzyme I)/GAF domain containing protein | GCO | 94.83% | 57.14% |
| NP_461921 | ygdP | dinucleoside polyphosphate hydrolase | - | NP_417307 | nudH | dinucleoside polyphosphate hydrolase | GCO | 95.45% | 50.65% |
| NP_951054 | - | hypothetical protein | - |  |  |  | NO HOMOLOG |  | 41.49% |
| NP_461922 | mutH | DNA mismatch repair protein | - | NP_417308 | mutH | DNA mismatch repair protein | GCO | 88.64% | 57.75% |
| NP_461923 | ygdQ | putative transport protein | - | NP_417309 | ygdQ | predicted inner membrane protein | GCO | 70.46% | 50.42% |
| NP_461924 | ygdR | putative peptide transport protein | - | NP_417310 | ygdR | hypothetical protein | GCO | 90.27% | 47.03% |
| NP_461925 | tas | putative aldo/keto reductase | - | NP_417311 | tas | predicted oxidoreductase, NADP(H)-dependent aldo-keto reductase | GCO | 91.32% | 54.75% |
| NP_461926 | ygeD | putative efflux protein | - | NP_417312 | ygeD | predicted inner membrane protein | GCO | 72.47% | 58.60% |
| NP_461927 | aas | 2-acyl-glycerophospho-ethanolamine acyltransferase | - | NP_417313 | aas | 2-acyl-glycerophospho-ethanolamine acyltransferase | GCO | 91.65% | 55.04% |
| NP_461928 | galR | galETK operon transcriptional repressor | - | NP_417314 | galR | DNA-binding transcriptional repressor | GCO | 88.33% | 55.87% |
| NP_461929 | - | putative transcriptional regulator | - | NP_417194 | ascG | DNA-binding transcriptional repressor | nGCO | 41.01% | 57.86% |
| NP_461930 | lysA | diaminopimelate decarboxylase | - | NP_417315 | lysA | diaminopimelate decarboxylase, PLP-binding | GCO | 88.57% | 57.40% |
| NP_461931 | lysR | transcriptional regulator | - | NP_417316 | lysR | DNA-binding transcriptional dual regulator | GCO | 84.24% | 58.54% |
| NP_461932 | ygeA | putative aspartate racemase | - | NP_417317 | ygeA | predicted racemase | GCO | 70.86% | 53.38% |
| NP_461933 | araE | L-arabinose/proton symport protein | - | NP_417318 | araE | arabinose transporter | GCO | 93.00% | 52.78% |
| NP_461934 | kduD | 2-deoxy-D-gluconate 3-dehydrogenase | - | NP_417319 | kduD | 2-deoxy-D-gluconate 3-dehydrogenase | GCO | 93.28% | 51.83% |
| NP_461935 | kduI | 5-keto-4-deoxyuronate isomerase | - | NP_417320 | kduI | 5-keto-4-deoxyuronate isomerase | GCO | 81.65% | 51.49% |
| NP_461936 | yqeF | acetyl-CoA acetyltransferase | - | NP_417321 | yqeF | acetyl-CoA acetyltransferase | GCO | 91.32% | 58.43% |
| NP_461937 | - | putative transcriptional regulator | - | NP_415037 | ybbS | DNA-binding transcriptional activator of the allD operon | nGCO | 25.62% | 54.51% |
| NP_461938 | - | putative inner membrane protein | - |  |  |  | NO HOMOLOG |  | 50.51% |
| NP_461939 | - | putative transport protein | - | NP_417322 | yqeG | predicted transporter | nGCO | 87.00% | 46.99% |
| NP_461940 | yohL | putative cytoplasmic protein | - | NP_416608 | yohL | hypothetical protein | GCO | 93.33% | 46.15% |
| NP_461941 | yohM | putative inner membrane protein | - | NP_416609 | yohM | membrane protein conferring nickel and cobalt resistance | GCO | 67.70% | 53.86% |
| NP_461942 | - | putative cytoplasmic protein | + |  |  |  | NO HOMOLOG |  | 54.54% |
| NP_945169 | - | hypothetical protein | - |  |  |  | NO HOMOLOG |  | 52.63% |
| NP_461943 | - | putative outer membrane protein | - |  |  |  | NO HOMOLOG |  | 40.11% |
| NP_461944 | stdC | putative fimbrial chaparone | - | NP_415245 | ybgP | predicted assembly protein | nGCO | 40.18% | 58.87% |
| NP_461945 | stdB | putative outer membrane usher protein | - | YP_026198 | yqiG | predicted outer membrane usher protein | nGCO | 41.50% | 60.72% |
| NP_461946 | stdA | putative fimbrial-like protein | - | NP_415247 | ybgD | predicted fimbrial-like adhesin protein | nGCO | 32.63% | 52.76% |
| NP_461947 | - | putative periplasmic protein | + | NP_416876 | yfdX | hypothetical protein | nGCO | 38.75% | 45.02% |
| NP_461948 | - | Ail/OmpX-like protein | + | NP_415335 | ompX | outer membrane protein X | nGCO | 29.29% | 48.60% |
| NP_461949 | tnpA_4 | transposase | - |  |  |  | NO HOMOLOG |  | 45.96% |
| NP_461950 | - | putative nucleic acid-binding protein | - |  |  |  | NO HOMOLOG |  | 50.12% |
| NP_461951 | - | putative cytoplasmic protein | - |  |  |  | NO HOMOLOG |  | 47.36% |
| NP_461953 | - | putative inner membrane protein | - |  |  |  | NO HOMOLOG |  | 51.30% |
| NP_461954 | - | putative metalloendopeptidase | - | NP_417341 | ygeR | Tetratricopeptide repeat transcriptional regulator | nGCO | 66.26% | 53.75% |
| NP_461955 | idi | isopentenyl-diphosphate delta-isomerase | - | NP_417365 | idi | isopentenyl-diphosphate delta-isomerase | GCO | 70.55% | 52.01% |
| NP_461956 | lysS | lysyl-tRNA synthetase | - | NP_417366 | lysS | lysine tRNA synthetase, constitutive | GCO | 95.04% | 54.08% |
| NP_461957 | prfB | peptide chain release factor 2 | - | NP_417367 | prfB | peptide chain release factor 2 | GCO | 89.85% | 54.31% |
| NP_461958 | recJ | single-stranded-DNA-specific exonuclease | - | NP_417368 | recJ | ssDNA exonuclease, 5' --> 3'-specific | GCO | 90.29% | 57.32% |
| NP_461959 | dsbC | protein disulfide isomerase II | - | NP_417369 | dsbC | protein disulfide isomerase II | GCO | 83.54% | 50.28% |
| NP_461960 | xerD | tyrosine recombinase | - | NP_417370 | xerD | site-specific tyrosine recombinase XerD | GCO | 95.30% | 53.17% |
| NP_461961 | fldB | flavodoxin | - | NP_417371 | fldB | flavodoxin 2 | GCO | 94.21% | 52.68% |
| NP_461962 | ygfX | putative inner membrane protein | - | NP_417372 | ygfX | hypothetical protein | GCO | 83.96% | 55.07% |
| NP_461963 | ygfY | putative cytoplasmic protein | - | NP_417373 | ygfY | hypothetical protein | GCO | 93.18% | 44.19% |
| NP_461964 | ygfZ | putative aminomethyltransferase | - | NP_417374 | ygfZ | putative global regulator | GCO | 85.58% | 57.08% |
| NP_461965 | yqfA | putative hemolysin | - | NP_417375 | yqfA | predicted oxidoreductase, inner membrane subunit | GCO | 78.08% | 50.75% |
| NP_461966 | yqfB | hypothetical protein | - | NP_417376 | yqfB | hypothetical protein | GCO | 88.34% | 45.19% |
| NP_461967 | bglA | 6-phospho-beta-glucosidase A | - | NP_417377 | bglA | 6-phospho-beta-glucosidase A | GCO | 92.24% | 50.97% |
| NP_461968 | - | putative outer membrane protein | - |  |  |  | NO HOMOLOG |  | 43.40% |
| NP_461969 | gcvP | glycine dehydrogenase | - | NP_417379 | gcvP | glycine dehydrogenase | GCO | 90.38% | 56.71% |
| NP_461970 | gcvH | glycine cleavage system protein H | - | NP_417380 | gcvH | glycine cleavage system protein H | GCO | 99.22% | 54.87% |
| NP_461971 | gcvT | aminomethyltransferase | - | NP_417381 | gcvT | glycine cleavage system aminomethyltransferase T | GCO | 94.78% | 55.52% |
| NP_461972 | visC | hypothetical protein | - | NP_417382 | visC | hypothetical protein | GCO | 87.25% | 57.27% |
| NP_461973 | ubiH | 2-octaprenyl-6-methoxyphenyl hydroxylase | - | NP_417383 | ubiH | 2-octaprenyl-6-methoxyphenyl hydroxylase | GCO | 76.78% | 60.98% |
| NP_461974 | pepP | proline aminopeptidase P II | - | NP_417384 | pepP | proline aminopeptidase P II | GCO | 92.38% | 55.88% |
| NP_461975 | ygfB | hypothetical protein | - | NP_417385 | ygfB | hypothetical protein | GCO | 84.37% | 54.05% |
| NP_461976 | ygfE | putative cytoplasmic protein | - | NP_417386 | zapA | protein that localizes to the cytokinetic ring | GCO | 96.33% | 47.57% |
| NP_461977 | ygfA | putative ligase | - | NP_417387 | ygfA | predicted ligase | GCO | 82.41% | 57.55% |
| NP_461978 | serA | D-3-phosphoglycerate dehydrogenase | - | NP_417388 | serA | D-3-phosphoglycerate dehydrogenase | GCO | 94.87% | 55.39% |
| NP_461979 | rpiA | ribose-5-phosphate isomerase A | - | NP_417389 | rpiA | ribose-5-phosphate isomerase A | GCO | 98.17% | 54.54% |
| NP_461980 | iciA | chromosome replication initiation inhibitor protein | - | NP_417391 | argP | chromosome replication initiation inhibitor protein | nGCO | 91.91% | 57.94% |
| NP_461981 | yggE | hypothetical protein | + | NP_417397 | yggE | hypothetical protein | GCO | 77.02% | 55.55% |
| NP_461982 | yggA | arginine exporter protein | - | NP_417398 | argO | arginine exporter protein | GCO | 82.46% | 55.18% |
| NP_461983 | yggB | putative mechanosensitive channel | - | NP_417399 | mscS | mechanosensitive channel | GCO | 77.54% | 50.87% |
| NP_461984 | fba | fructose-bisphosphate aldolase | - | NP_417400 | fbaA | fructose-bisphosphate aldolase | GCO | 98.32% | 51.38% |
| NP_461985 | pgk | phosphoglycerate kinase | - | NP_417401 | pgk | phosphoglycerate kinase | GCO | 97.93% | 54.46% |
| NP_461986 | epd | D-erythrose 4-phosphate dehydrogenase | - | NP_417402 | epd | D-erythrose 4-phosphate dehydrogenase | GCO | 94.10% | 53.58% |
| NP_461987 | - | putative DNA-binding protein | - |  |  |  | NO HOMOLOG |  | 56.10% |
| NP_461988 | - | putative inner membrane protein | - |  |  |  | NO HOMOLOG |  | 56.64% |
| NP_461989 | - | putative ABC-type cobalt transport system permease component | - |  |  |  | NO HOMOLOG |  | 59.74% |
| NP_461990 | - | putative ABC-type cobalt transport system ATP-binding component | - | NP_417937 | nikE | nickel transporter subunit | nGCO | 33.33% | 55.89% |
| NP_461991 | - | putative ABC-type cobalt transport system ATP-binding component | - | NP_415635 | lolD | outer membrane-specific lipoprotein transporter subunit | nGCO | 29.71% | 58.29% |
| NP_461992 | tktA | transketolase | - | YP_026188 | tktA | transketolase 1, thiamin-binding | GCO | 95.92% | 57.27% |
| NP_461993 | yggG | putative Zn-dependent protease | - | NP_417411 | yggG | predicted peptidase | GCO | 82.14% | 54.94% |
| NP_461994 | speB | agmatinase | - | NP_417412 | speB | agmatinase | GCO | 94.77% | 55.48% |
| NP_461995 | - | putative hydrolase/acyltransferase | + |  |  |  | NO HOMOLOG |  | 50.49% |
| NP_461996 | - | putative mannitol dehydrogenase | + | NP_417688 | yhcH | hypothetical protein | nGCO | 34% | 40.92% |
| NP_461997 | - | putative malate/L-lactate dehydrogenase | - | NP_415050 | allD | ureidoglycolate dehydrogenase | nGCO | 40.60% | 51.09% |
| NP_461998 | - | putative zinc-binding dehydrogenase | - | NP_418778 | yjjN | predicted oxidoreductase, Zn-dependent and NAD(P)-binding | nGCO | 46.74% | 51.72% |
| NP_461999 | - | putative mannitol dehydrogenase | - | NP_416677 | yeiQ | predicted dehydrogenase, NAD-dependent | nGCO | 49.37% | 51.45% |
| NP_462000 | - | putative regulatory protein | - | NP_418744 | uxuR | DNA-binding transcriptional repressor | nGCO | 40.35% | 44.93% |
| NP_462001 | - | putative outer membrane lipoprotein | + |  |  |  | NO HOMOLOG |  | 48.22% |
| NP_462002 | speA | arginine decarboxylase | - | NP_417413 | speA | arginine decarboxylase | GCO | 95.20% | 55.43% |
| NP_462003 | yqgB | putative inner membrane protein | - | NP_417414 | yqgB | hypothetical protein | GCO | 74.35% | 44.69% |
| NP_462005 | yqgD | putative inner membrane protein | - | NP_417416 | yqgD | predicted inner membrane protein | GCO | 52.56% | 45.33% |
| NP_462006 | metK | S-adenosylmethionine synthetase | - | NP_417417 | metK | S-adenosylmethionine synthetase | GCO | 97.39% | 54.28% |
| NP_462007 | galP | galactose/proton symporter | - | NP_417418 | galP | D-galactose transporter | GCO | 94.82% | 55.19% |
| NP_462008 | sprT | hypothetical protein | - | NP_417419 | sprT | hypothetical protein | GCO | 90.24% | 57.63% |
| NP_462009 | endA | DNA-specific endonuclease I | - | NP_417420 | endA | DNA-specific endonuclease I | GCO | 83.09% | 55.64% |
| NP_462010 | yggJ | putative cytoplasmic protein | - | NP_417421 | yggJ | hypothetical protein | GCO | 87.65% | 55.12% |
| NP_462011 | gshB | glutathione synthetase | - | NP_417422 | gshB | glutathione synthetase | GCO | 90.09% | 54.53% |
| NP_462012 | yqgE | hypothetical protein | - | NP_417423 | yqgE | hypothetical protein | GCO | 92.51% | 52.48% |
| NP_462013 | yqgF | Holliday junction resolvase-like protein | - | NP_417424 | yqgF | Holliday junction resolvase-like protein | GCO | 95.65% | 55.63% |
| NP_462014 | - | putative transcriptional regulator | - |  |  |  | NO HOMOLOG |  | 57.20% |
| NP_462015 | yggR | twitching motility protein | - | NP_417425 | yggR | predicted transporter | GCO | 79.44% | 57.28% |
| NP_462016 | yggS | hypothetical protein | - | NP_417426 | yggS | predicted enzyme | GCO | 83.69% | 53.47% |
| NP_462017 | yggT | putative integral membrane protein | - | NP_417427 | yggT | predicted inner membrane protein | GCO | 74.46% | 52.02% |
| NP_462018 | yggU | hypothetical protein | - | NP_417428 | yggU | hypothetical protein | GCO | 90.42% | 51.89% |
| NP_462019 | yggV | putative deoxyribonucleotide triphosphate pyrophosphatase | - | NP_417429 | yggV | putative deoxyribonucleotide triphosphate pyrophosphatase | GCO | 91.87% | 55.38% |
| NP_462020 | yggW | coproporphyrinogen III oxidase | - | NP_417430 | yggW | coproporphyrinogen III oxidase | GCO | 92.32% | 54.61% |
| NP_462021 | yggM | putative periplasmic protein | - | NP_417431 | yggM | hypothetical protein | GCO | 64.47% | 46.13% |
| NP_462022 | ansB | periplasmic L-asparaginase II | - | NP_417432 | ansB | periplasmic L-asparaginase II | GCO | 88.79% | 52.62% |
| NP_462023 | yggN | putative periplasmic protein | - | NP_417433 | yggN | hypothetical protein | GCO | 87.44% | 54.86% |
| NP_462024 | yggL | putative cytoplasmic protein | - | NP_417434 | yggL | hypothetical protein | GCO | 95.37% | 47.70% |
| NP_462025 | yggH | tRNA (guanine-N(7)-)-methyltransferase | - | NP_417435 | yggH | tRNA(m7G46)-methyltransferase | GCO | 93.72% | 53.19% |
| NP_462026 | mutY | adenine DNA glycosylase | - | NP_417436 | mutY | adenine DNA glycosylase | GCO | 90.57% | 54.51% |
| NP_462027 | yggX | hypothetical protein | - | NP_417437 | yggX | hypothetical protein | GCO | 94.50% | 47.46% |
| NP_462028 | mltC | membrane-bound lytic murein transglycosylase C | - | NP_417438 | mltC | membrane-bound lytic murein transglycosylase C | GCO | 88.33% | 51.74% |
| NP_462029 | nupG | nucleoside transport | - | NP_417439 | nupG | nucleoside transporter | GCO | 88.51% | 51.47% |
| NP_462030 | speC | ornithine decarboxylase isozyme | - | NP_417440 | speC | ornithine decarboxylase, constitutive | GCO | 87.06% | 54.54% |
| NP_462031 | yqgA | putative inner membrane protein | - | NP_417441 | yqgA | predicted inner membrane protein | GCO | 79.57% | 51.55% |
| NP_462032 | - | putative lactoylglutathione lyase | + |  |  |  | NO HOMOLOG |  | 37.70% |
| NP_462033 | - | putative acetyl-CoA hydrolase | - | NP_417395 | ygfH | propionyl-CoA:succinate-CoA transferase | nGCO | 27.77% | 45.24% |
| NP_462034 | - | putative monoamine oxidase | - |  |  |  | NO HOMOLOG |  | 50% |
| NP_462035 | - | putative transcriptional regulator | - | NP_415149 | citE | citrate lyase, citryl-ACP lyase (beta) subunit | nGCO | 27.61% | 49.58% |
| NP_462036 | - | putative transcriptional regulator | - | NP_416112 | ynfL | predicted DNA-binding transcriptional regulator | nGCO | 38.67% | 52.44% |
| NP_462037 | - | putative arylsulfatase | - | NP_418134 | yidJ | predicted sulfatase/phosphatase | nGCO | 26.15% | 48.56% |
| NP_462038 | - | putative arylsulfatase regulator | - | YP_026259 | aslB | predicted regulator of arylsulfatase activity | nGCO | 36.43% | 49.87% |
| NP_462039 | - | putative response regulator | - |  |  |  | NO HOMOLOG |  | 44.88% |
| NP_462040 | - | putative cytoplasmic protein | - |  |  |  | NO HOMOLOG |  | 46.41% |
| NP_462041 | - | putative amino acid transporter | - | NP_414936 | proY | predicted cryptic proline transporter | nGCO | 23.98% | 51.53% |
| NP_462042 | - | putative cytoplasmic protein | - |  |  |  | NO HOMOLOG |  | 49.06% |
| NP_462043 | - | putative oxidoreductase | - | NP_415817 | puuB | gamma-Glu-putrescine oxidase, FAD/NAD(P)-binding | nGCO | 34.46% | 49.38% |
| NP_462044 | - | putative NAD-dependent aldehyde dehydrogenase | - | NP_415903 | feaB | phenylacetaldehyde dehydrogenase | nGCO | 43.11% | 51.17% |
| NP_462045 | - | putative cytoplasmic protein | - | NP_418746 | yjiD | DNA replication/recombination/repair protein | nGCO | 46.93% | 50.91% |
| NP_462046 | - | putative cytoplasmic protein | - |  |  |  | NO HOMOLOG |  | 50.61% |
| NP_462047 | - | putative xylanase/chitin deacetylase | + |  |  |  | NO HOMOLOG |  | 47.29% |
| NP_462048 | - | putative amidohydrolase | + |  |  |  | NO HOMOLOG |  | 43.79% |
| NP_462049 | - | putative permease | - | NP_417564 | exuT | hexuronate transporter | nGCO | 47.72% | 52.56% |
| NP_462050 | - | mannonate dehydratase | - | NP_418742 | uxuA | mannonate dehydratase | GCO | 91.87% | 52.65% |
| NP_462051 | - | putative D-mannonate oxidoreductase | - | NP_418743 | uxuB | D-mannonate oxidoreductase, NAD-binding | GCO | 75.61% | 56.48% |
| NP_462052 | - | uronate isomerase | - | NP_417563 | uxaC | glucuronate isomerase | nGCO | 67.02% | 53.92% |
| NP_462053 | - | putative methyl-accepting chemotaxis protein | - |  |  |  | NO HOMOLOG |  | 43.05% |
| NP_462054 | gsp | glutathionylspermidine synthetase/glutathionylspermidine amidase | - | NP_417462 | gss | fused glutathionylspermidine amidase/glutathionylspermidine synthetase | GCO | 90.12% | 53.47% |
| NP_462055 | yghU | putative glutathione S-transferase | - | NP_417463 | yghU | predicted S-transferase | GCO | 88.15% | 54.55% |
| NP_462056 | - | hypothetical protein | - |  |  |  | NO HOMOLOG |  | 59.21% |
| NP_462057 | - | putative periplasmic ferrichrome-binding protein | - |  |  |  | NO HOMOLOG |  | 53.56% |
| NP_462058 | hybG | chaperone-like protein | - | NP_417464 | hybG | hydrogenase 2 accessory protein | GCO | 93.90% | 52.61% |
| NP_462059 | hypA | hydrogenase nickel incorporation protein | - | NP_417465 | hybF | protein involved with the maturation of hydrogenases 1 and 2 | GCO | 84.95% | 55.55% |
| NP_462060 | hybE | putative hydrogenase | - | NP_417466 | hybE | hydrogenase 2-specific chaperone | GCO | 74.69% | 56.44% |
| NP_462061 | hybD | putative hydrogenase-2 processing element | - | NP_417467 | hybD | predicted maturation element for hydrogenase 2 | GCO | 94.47% | 54.54% |
| NP_462062 | hybC | hydrogenase-2 large subunit | - | NP_417468 | hybC | hydrogenase 2, large subunit | GCO | 94.00% | 55.04% |
| NP_462063 | hybB | putative chaperone | - | NP_417469 | hybB | predicted hydrogenase 2 cytochrome b type component | GCO | 93.11% | 52.84% |
| NP_462064 | hybA | putative hydrogenase-2 component | - | NP_417470 | hybA | hydrogenase 2 4Fe-4S ferredoxin-type component | GCO | 89.02% | 53.79% |
| NP_462065 | hypO | putative Ni/Fe hydrogenase small subunit | - | NP_417471 | hybO | hydrogenase 2, small subunit | GCO | 86.82% | 54.24% |
| NP_462066 | yghW | putative cytoplasmic protein | - | NP_417472 | yghW | hypothetical protein | GCO | 78.72% | 46.87% |
| NP_462067 | - | putative methyl-accepting chemotaxis protein | - | NP_418775 | tsr | methyl-accepting chemotaxis protein I, serine sensor receptor | nGCO | 34.17% | 57.17% |
| NP_462068 | yqhA | hypothetical protein | - | NP_417475 | yqhA | hypothetical protein | nGCO | 94.51% | 49.09% |
| NP_462069 | - | putative ATP-dependent RNA helicase-like protein | + |  |  |  | NO HOMOLOG |  | 43.21% |
| NP_462070 | - | putative cytoplasmic protein | + |  |  |  | NO HOMOLOG |  | 42.57% |
| NP_462071 | - | putative cytoplasmic protein | + |  |  |  | NO HOMOLOG |  | 47.40% |
| NP_462072 | yghA | oxidoreductase | - | NP_417476 | yghA | oxidoreductase | GCO | 88.09% | 56.61% |
| NP_462073 | exbD | energy transduction protein | - | NP_417478 | exbD | membrane spanning protein in TonB-ExbB-ExbD complex | GCO | 93.61% | 50.70% |
| NP_462074 | exbB | energy transduction protein | - | NP_417479 | exbB | membrane spanning protein in TonB-ExbB-ExbD complex | GCO | 89.75% | 55.64% |
| NP_462075 | - | putative inner membrane protein | - |  |  |  | NO HOMOLOG |  | 38% |
| NP_462076 | metC | cystathionine beta-lyase | - | NP_417481 | metC | cystathionine beta-lyase | GCO | 86.58% | 53.53% |
| NP_462077 | yghB | hypothetical protein | - | NP_417482 | yghB | conserved inner membrane protein | GCO | 83.10% | 53.78% |
| NP_462078 | yqhC | putative transcriptional regulator | - | NP_417483 | yqhC | predicted DNA-binding transcriptional regulator | GCO | 83.88% | 51.08% |
| NP_462079 | yqhD | putative alcohol dehydrogenase | - | NP_417484 | yqhD | alcohol dehydrogenase, NAD(P)-dependent | GCO | 88.63% | 55.24% |
| NP_462080 | yqhE | 2,5-diketo-D-gluconate reductase A | - | NP_417485 | dkgA | 2,5-diketo-D-gluconate reductase A | GCO | 88.36% | 52.89% |
| NP_462081 | - | putative cation transporter | - | NP_415291 | ybhI | predicted transporter | nGCO | 36.01% | 55.60% |
| NP_462082 | - | putative diadenosine tetraphosphatehydrolase | - |  |  |  | NO HOMOLOG |  | 48.02% |
| NP_462083 | ygiR | hypothetical protein | - | YP_026196 | ygiQ | hypothetical protein | nGCO | 95.02% | 57.22% |
| NP_462084 | - | putative periplasmic dicarboxylate-binding protein | - | NP_418036 | yiaO | predicted transporter | nGCO | 27.70% | 51.72% |
| NP_462085 | - | putative inner membrane protein | - | NP_418034 | yiaM | predicted transporter | GCO | 24.30% | 47.41% |
| NP_462086 | ygiK | putative transporter | - | YP_026232 | yiaN | predicted transporter | GCO | 27.38% | 51.14% |
| NP_462087 | sufI | suppressor of ftsI | - | NP_417489 | sufI | repressor protein for FtsI | GCO | 83.33% | 58.95% |
| NP_462088 | plsC | 1-acyl-sn-glycerol-3-phosphate acyltransferase | - | NP_417490 | plsC | 1-acyl-sn-glycerol-3-phosphate acyltransferase | GCO | 88.97% | 50% |
| NP_462089 | parC | DNA topoisomerase IV subunit A | - | NP_417491 | parC | DNA topoisomerase IV subunit A | GCO | 95.07% | 56.21% |
| NP_462090 | - | putative regulatory protein | - | YP_026197 | ygiV | predicted transcriptional regulator | GCO | 55.19% | 58.01% |
| NP_462091 | ygiW | putative outer membrane protein | - | NP_417496 | ygiW | hypothetical protein | GCO | 89.23% | 54.19% |
| NP_462092 | ygiX | putative transcriptional regulator | - | NP_417497 | qseB | DNA-binding response regulator in two-component regulatory system with QseC | GCO | 82.56% | 56.21% |
| NP_462093 | ygiY | putative sensor histidine kinase | - | NP_417498 | qseC | sensory histidine kinase in two-component regulatory system with QseB | GCO | 73.27% | 56.29% |
| NP_462094 | mdaB | NADPH-specific quinone oxidoreductase | - | NP_417500 | mdaB | NADPH quinone reductase | GCO | 92.70% | 51.37% |
| NP_462095 | ygiN | putative cytoplasmic protein | - | NP_417501 | ygiN | quinol monooxygenase | GCO | 90.38% | 53.01% |
| NP_462096 | parE | DNA topoisomerase IV subunit B | - | NP_417502 | parE | DNA topoisomerase IV subunit B | GCO | 94.60% | 55.62% |
| NP_462097 | yqiA | putative esterase | - | NP_417503 | yqiA | predicted esterase | GCO | 85.86% | 54.63% |
| NP_462098 | icc | cyclic 3',5'-adenosine monophosphate phosphodiesterase | - | NP_417504 | cpdA | cyclic 3',5'-adenosine monophosphate phosphodiesterase | GCO | 88.72% | 55.91% |
| NP_462099 | yqiB | putative cytoplasmic protein | - | NP_417505 | yqiB | predicted dehydrogenase | GCO | 95% | 50.35% |
| NP_462100 | yqiE | ADP-ribose pyrophosphatase | - | NP_417506 | nudF | ADP-ribose pyrophosphatase | GCO | 83.17% | 53.08% |
| NP_462101 | tolC | outer membrane channel precursor protein | - | NP_417507 | tolC | outer membrane channel precursor protein | GCO | 84.56% | 52.03% |
| NP_462102 | ygiB | putative inner membrane protein | - | NP_417509 | ygiB | conserved outer membrane protein | GCO | 89.00% | 57.88% |
| NP_462103 | ygiC | putative glutathionylspermidine synthase | - | NP_417510 | ygiC | predicted enzyme | GCO | 94.04% | 51.63% |
| NP_462104 | ygiD | putative cytoplasmic protein | - | NP_417511 | zupT | predicted dioxygenase | GCO | 83.08% | 55.47% |
| NP_462105 | ygiE | zinc transporter ZupT | - | NP_417512 | ygiE | zinc transporter ZupT | GCO | 86.77% | 56.58% |
| NP_462106 | - | putative arylsulfate sulfotransferase | + |  |  |  | NO HOMOLOG |  | 49.58% |
| NP_462107 | - | putative arylsulfate sulfotransferase | + |  |  |  | NO HOMOLOG |  | 48.55% |
| NP_462108 | - | putative disulfide bond formation protein | + | NP_418297 | dsbA | periplasmic protein disulfide isomerase I | nGCO | 29.27% | 46.13% |
| NP_462109 | - | putative disulfide oxidoreductase | + | NP_415703 | dsbB | disulfide bond formation protein B | nGCO | 30.23% | 51.03% |
| NP_462110 | ribB | 3,4-dihydroxy-2-butanone 4-phosphate synthase | - | NP_417513 | ribB | 3,4-dihydroxy-2-butanone 4-phosphate synthase | GCO | 96.31% | 56.11% |
| NP_462111 | yqiC | putative cytoplasmic protein | - | NP_417514 | yqiC | hypothetical protein | GCO | 84.04% | 52.66% |
| NP_462112 | glgS | glycogen synthesis protein GlgS | - | NP_417521 | glgS | glycogen synthesis protein GlgS | GCO | 83.92% | 48.09% |
| NP_462113 | - | putative inner membrane protein | - | NP_417522 | yqiJ | predicted inner membrane protein | GCO | 69.15% | 52.97% |
| NP_462114 | yqiK | hypothetical protein | - | NP_417523 | yqiK | hypothetical protein | GCO | 70.70% | 53.69% |
[truncated: 176,370 more chars]
