# Supplementary material for: Gene socialization: gene order, GC content and gene silencing in Salmonella
Source: BMC Genomics. 2009 Dec 11;10:597. doi: 10.1186/1471-2164-10-597 (PMC2801525; doi:10.1186/1471-2164-10-597)
Supplement: Additional file 2 — GC content analysis of the Salmonella gene set after removing predicted HTGs. Table displaying the content analysis of the Salmonella gene set after removing predicted HTGs. [file 1471-2164-10-597-S2.DOC]

***Salmonella* genes GC content Standard deviation**

GCO (2899 genes) 53.7 3.5

nGCO(463 genes) 52.0 4.6

No homolog in *E. coli* K12 (626 genes) 50.9 6.7

Kruskal-Wallis rank sum test

Kruskal-Wallis chi-squared = 121.3866, df = 2, P-value < 2.2e-16
