# Supplementary material for: Gene socialization: gene order, GC content and gene silencing in Salmonella
Source: BMC Genomics. 2009 Dec 11;10:597. doi: 10.1186/1471-2164-10-597 (PMC2801525; doi:10.1186/1471-2164-10-597)
Supplement: Additional file 3 — Duplicates in Salmonella that share the same best hit in E. coli K12. Table displaying in Salmonella that share the same best hit in E. coli K12. [file 1471-2164-10-597-S3.DOC]

| **SALMO** | **name** | **Salmonela gene description** | **hns** | **k12** | **name** | **K12 gene description** | **GCO** | **identity** | **%GC content** |
| --- | --- | --- | --- | --- | --- | --- | --- | --- | --- |
| NP_462044 | - | putative NAD-dependent aldehyde dehydrogenase | - | NP_415903 | feaB | phenylacetaldehyde dehydrogenase | nGCO | 43.11 | 51.17 |
| NP_463282 | - | putative NAD-dependent aldehyde dehydrogenase | - | NP_415903 | feaB | phenylacetaldehyde dehydrogenase | nGCO | 34.14 | 56.7 |
| --=-- |  |  |  |  |  |  |  |  |  |
| NP_462349 | tuf | elongation factor Tu | - | NP_417798 | tufA | protein chain elongation factor EF-Tu (duplicate of tufB) | GCO | 86.8 | 54 |
| NP_463015 | tuf | elongation factor Tu | - | NP_417798 | tufA | protein chain elongation factor EF-Tu (duplicate of tufB) | nGCO | 86.8 | 54 |
| --=-- |  |  |  |  |  |  |  |  |  |
| NP_460493 | - | putative hydrogenase | - | NP_415496 | hyaF | protein involved in nickel incorporation into hydrogenase-1 proteins | GCO | 30.87 | 50.37 |
| NP_460747 | - | putative hydrogenase-1 protein | - | NP_415496 | hyaF | protein involved in nickel incorporation into hydrogenase-1 proteins | GCO | 70.96 | 59.59 |
| --=-- |  |  |  |  |  |  |  |  |  |
| NP_459065 | citE2 | putative citrate lyase beta chain | - | NP_415149 | citE | citrate lyase, citryl-ACP lyase (beta) subunit | GCO | 60.34 | 55.63 |
| NP_459614 | citE | citrate lyase beta chain | - | NP_415149 | citE | citrate lyase, citryl-ACP lyase (beta) subunit | GCO | 88.41 | 59.51 |
| NP_462035 | - | putative transcriptional regulator | - | NP_415149 | citE | citrate lyase, citryl-ACP lyase (beta) subunit | nGCO | 27.61 | 49.58 |
| --=-- |  |  |  |  |  |  |  |  |  |
| NP_460579 | - | putative oxidase | - | NP_418062 | lldD | L-lactate dehydrogenase, FMN-linked | nGCO | 31.96 | 48.29 |
| NP_462594 | lldD | L-lactate dehydrogenase | - | NP_418062 | lldD | L-lactate dehydrogenase, FMN-linked | GCO | 92.67 | 59.78 |
| --=-- |  |  |  |  |  |  |  |  |  |
| NP_461036 | rfbG | CDP glucose 4,6-dehydratase | - | NP_416557 | gmd | GDP-D-mannose dehydratase, NAD(P)-binding | nGCO | 23.8 | 43.61 |
| NP_461054 | gmd | GDP-D-mannose dehydratase | + | NP_416557 | gmd | GDP-D-mannose dehydratase, NAD(P)-binding | GCO | 96.24 | 56.59 |
| --=-- |  |  |  |  |  |  |  |  |  |
| NP_459573 | - | putative regulatory protein | - | NP_416048 | marA | DNA-binding transcriptional dual activator of multiple antibiotic resistance | nGCO | 39.6 | 50 |
| NP_460479 | marA | transcriptional activator | - | NP_416048 | marA | DNA-binding transcriptional dual activator of multiple antibiotic resistance | GCO | 95.23 | 46.66 |
| --=-- |  |  |  |  |  |  |  |  |  |
| NP_460584 | ydcI | putative transcriptional regulator | - | NP_415939 | ydcI | predicted DNA-binding transcriptional regulator | GCO | 83.33 | 54.65 |
| NP_461125 | - | putative transcriptional regulator | - | NP_415939 | ydcI | predicted DNA-binding transcriptional regulator | nGCO | 26.31 | 54.89 |
| --=-- |  |  |  |  |  |  |  |  |  |
| NP_459743 | - | transcriptional regulator | + | NP_414872 | cynR | DNA-binding transcriptional dual regulator | nGCO | 25.61 | 34.9 |
| NP_461510 | - | putative transcriptional regulator | + | NP_414872 | cynR | DNA-binding transcriptional dual regulator | nGCO | 30.08 | 42.93 |
| --=-- |  |  |  |  |  |  |  |  |  |
| NP_462727 | dgoT | D-galactonate transport protein | - | NP_418146 | dgoT | D-galactonate transporter | GCO | 88.83 | 54.03 |
| NP_463273 | - | putative permease | - | NP_418146 | dgoT | D-galactonate transporter | nGCO | 20.58 | 47.55 |
| --=-- |  |  |  |  |  |  |  |  |  |
| NP_459299 | safC | putative fimbrial usher | + | NP_418737 | fimD | outer membrane usher protein, type 1 fimbrial synthesis | nGCO | 35.17 | 54.67 |
| NP_459333 | stbC | putative fimbrial usher | - | NP_418737 | fimD | outer membrane usher protein, type 1 fimbrial synthesis | nGCO | 30.8 | 53.94 |
| NP_463449 | sthB | putative fimbrial usher protein | - | NP_418737 | fimD | outer membrane usher protein, type 1 fimbrial synthesis | GCO | 39.53 | 47.2 |
| --=-- |  |  |  |  |  |  |  |  |  |
| NP_459114 | yabN | putative periplasmic binding protein | - | NP_414611 | sgrR | DNA-binding transcriptional regulator | GCO | 85.86 | 56.66 |
| NP_461686 | - | putative dipeptide/oligopeptide/nickel ABC-type transport system periplasmic component | - | NP_414611 | sgrR | DNA-binding transcriptional regulator | nGCO | 26.58 | 57.02 |
| NP_462759 | - | putative dipeptide/oligopeptide/nickel ABC-type transport system periplasmic component | - | NP_414611 | sgrR | DNA-binding transcriptional regulator | nGCO | 38.74 | 55.91 |
| --=-- |  |  |  |  |  |  |  |  |  |
| NP_459742 | - | fumarate hydratase | + | NP_417533 | ttdA | tartrate dehydratase | GCO | 27.58 | 40.89 |
| NP_462265 | - | tartrate dehydratase | - | NP_417533 | ttdA | tartrate dehydratase | GCO | 54.08 | 51.88 |
| --=-- |  |  |  |  |  |  |  |  |  |
| NP_460188 | ycfV | ATP-binding protein | - | NP_415635 | lolD | outer membrane-specific lipoprotein transporter subunit | GCO | 95.27 | 55.55 |
| NP_461991 | - | putative ABC-type cobalt transport system ATP-binding component | - | NP_415635 | lolD | outer membrane-specific lipoprotein transporter subunit | nGCO | 29.71 | 58.29 |
| --=-- |  |  |  |  |  |  |  |  |  |
| NP_460265 | gdhA | glutamate dehydrogenase | - | NP_416275 | gdhA | glutamate dehydrogenase | GCO | 92.84 | 57.96 |
| NP_460751 | - | putative glutamic dehyrogenase-like protein | - | NP_416275 | gdhA | glutamate dehydrogenase | nGCO | 32.32 | 55.5 |
| --=-- |  |  |  |  |  |  |  |  |  |
| NP_461002 | pduW | acetate/propionate kinase | - | NP_417585 | tdcD | propionate kinase/acetate kinase C, anaerobic | nGCO | 41.7 | 52.75 |
| NP_462156 | tdcD | acetate/propionate kinase | + | NP_417585 | tdcD | propionate kinase/acetate kinase C, anaerobic | GCO | 76.61 | 49.71 |
| --=-- |  |  |  |  |  |  |  |  |  |
| NP_459321 | - | putative truncated IS3 transposase | - | NP_416593 | insF-5 | IS3 element protein InsF | nGCO | 86.75 | 54.43 |
| NP_461696 | - | putative transposase | - | NP_416593 | insF-5 | IS3 element protein InsF | GCO | 81.25 | 53.97 |
| --=-- |  |  |  |  |  |  |  |  |  |
| NP_459027 | bcfB | fimbrial chaparone | - | NP_415064 | sfmC | pilin chaperone, periplasmic | nGCO | 51 | 50.94 |
| NP_459540 | fimC | periplasmic chaperone | + | NP_415064 | sfmC | pilin chaperone, periplasmic | GCO | 61.57 | 50.36 |
| --=-- |  |  |  |  |  |  |  |  |  |
| NP_462108 | - | putative disulfide bond formation protein | + | NP_418297 | dsbA | periplasmic protein disulfide isomerase I | nGCO | 29.27 | 46.13 |
| NP_462877 | dsbA | periplasmic protein disulfide isomerase I | - | NP_418297 | dsbA | periplasmic protein disulfide isomerase I | GCO | 86.05 | 50.32 |
| --=-- |  |  |  |  |  |  |  |  |  |
| NP_459422 | phnU | 2-aminoethylphosphonate transporter | - | NP_416919 | cysU | sulfate/thiosulfate transporter subunit | nGCO | 27.84 | 57.25 |
| NP_461378 | cysU | thiosulfate transport protein | - | NP_416919 | cysU | sulfate/thiosulfate transporter subunit | GCO | 84.11 | 55.87 |
| --=-- |  |  |  |  |  |  |  |  |  |
| NP_460380 | ssaN | type III secretion system ATPase | + | NP_416451 | fliI | flagellum-specific ATP synthase | nGCO | 42.01 | 51.22 |
| NP_460925 | fliI | flagellum-specific ATP synthase | - | NP_416451 | fliI | flagellum-specific ATP synthase | GCO | 91 | 60.24 |
| NP_461815 | invC | type III secretion system ATPase | - | NP_416451 | fliI | flagellum-specific ATP synthase | nGCO | 37.44 | 54.24 |
| --=-- |  |  |  |  |  |  |  |  |  |
| NP_462578 | - | putative regulatory protein | - | NP_418389 | yijO | predicted DNA-binding transcriptional regulator | nGCO | 23.97 | 54.41 |
| NP_462997 | yijO | putative regulatory protein | - | NP_418389 | yijO | predicted DNA-binding transcriptional regulator | GCO | 89 | 56.33 |
| --=-- |  |  |  |  |  |  |  |  |  |
| NP_461366 | ptsH | phosphohistidinoprotein-hexose phosphotransferase | - | NP_416910 | ptsH | phosphohistidinoprotein-hexose phosphotransferase component of PTS system (Hpr) | GCO | 100 | 51.16 |
| NP_462678 | - | putative phosphotransferase system HPr protein | + | NP_416910 | ptsH | phosphohistidinoprotein-hexose phosphotransferase component of PTS system (Hpr) | nGCO | 28.91 | 39.62 |
| --=-- |  |  |  |  |  |  |  |  |  |
| NP_460286 | ydjN | kinase/transporter-like protein | - | NP_416243 | ydjN | predicted transporter | GCO | 81.16 | 54.81 |
| NP_460333 | - | putative Na+-dicarboxylate symporter | - | NP_416243 | ydjN | predicted transporter | nGCO | 39.86 | 48.03 |
| --=-- |  |  |  |  |  |  |  |  |  |
| NP_460912 | fliC | flagellar biosynthesis protein | - | NP_416433 | fliC | flagellin | GCO | 50.39 | 49.19 |
| NP_461698 | fljB | flagellar biosynthesis protein | - | NP_416433 | fliC | flagellin | nGCO | 52.9 | 50.69 |
| --=-- |  |  |  |  |  |  |  |  |  |
| NP_463325 | - | ornithine carbamoyltransferase | - | NP_418675 | argI | ornithine carbamoyltransferase 1 | nGCO | 68.5 | 55.52 |
| NP_463329 | argI | ornithine carbamoyltransferase | - | NP_418675 | argI | ornithine carbamoyltransferase 1 | GCO | 85.62 | 52.13 |
| --=-- |  |  |  |  |  |  |  |  |  |
| NP_459057 | - | putative transcriptional regulator | - | NP_415153 | citB | DNA-binding response regulator in two-component regulatory system with citA | GCO | 47.29 | 48.61 |
| NP_459618 | dpiA | response regulator | + | NP_415153 | citB | DNA-binding response regulator in two-component regulatory system with citA | GCO | 84.07 | 53.74 |
| --=-- |  |  |  |  |  |  |  |  |  |
| NP_459323 | - | putative permease | + | NP_416113 | ynfM | predicted transporter | nGCO | 33.95 | 42.52 |
| NP_460446 | ynfM | putative transport protein | - | NP_416113 | ynfM | predicted transporter | GCO | 80.33 | 55.18 |
| --=-- |  |  |  |  |  |  |  |  |  |
| NP_460359 | ssaC | outer membrane secretin precursor | + | NP_417850 | hofQ | predicted fimbrial transporter | nGCO | 24.71 | 42.3 |
| NP_462391 | hofQ | putative type IV pilin assembly protein | - | NP_417850 | hofQ | predicted fimbrial transporter | GCO | 85.52 | 56.33 |
| --=-- |  |  |  |  |  |  |  |  |  |
| NP_461142 | - | putative phosphoserine phosphatase | - | NP_418805 | serB | 3-phosphoserine phosphatase | nGCO | 39.48 | 44.59 |
| NP_463434 | serB | 3-phosphoserine phosphatase | - | NP_418805 | serB | 3-phosphoserine phosphatase | GCO | 89.75 | 57.17 |
| --=-- |  |  |  |  |  |  |  |  |  |
| NP_459632 | mrdA | cell elongation-specific transpeptidase | - | NP_415168 | mrdA | transpeptidase involved in peptidoglycan synthesis (penicillin-binding protein 2) | GCO | 96.2 | 53.83 |
| NP_460867 | - | putative penicillin-binding protein | - | NP_415168 | mrdA | transpeptidase involved in peptidoglycan synthesis (penicillin-binding protein 2) | nGCO | 57.94 | 51.92 |
| --=-- |  |  |  |  |  |  |  |  |  |
| NP_461143 | - | putative regulatory protein | + | NP_418123 | uhpC | membrane protein regulates uhpT expression | nGCO | 38.74 | 51.02 |
| NP_462687 | uhpC | regulatory protein | - | NP_418123 | uhpC | membrane protein regulates uhpT expression | GCO | 82.64 | 59.66 |
| --=-- |  |  |  |  |  |  |  |  |  |
| NP_459154 | - | Na+/galactoside symporter | - | NP_418114 | yicJ | predicted transporter | nGCO | 43.73 | 50.95 |
| NP_462650 | yicJ | putative transport protein | - | NP_418114 | yicJ | predicted transporter | GCO | 84.13 | 54.44 |
| NP_462946 | - | putative Na+/galactoside symporter | - | NP_418114 | yicJ | predicted transporter | nGCO | 34.9 | 50.48 |
| --=-- |  |  |  |  |  |  |  |  |  |
| NP_460355 | orf242 | putative regulatory protein | + | NP_416631 | mlrA | DNA-binding transcriptional regulator | nGCO | 44.1 | 53.9 |
| NP_461105 | yehV | putative transcriptional repressor | - | NP_416631 | mlrA | DNA-binding transcriptional regulator | GCO | 70.78 | 54.37 |
| --=-- |  |  |  |  |  |  |  |  |  |
| NP_460452 | - | putative ABC transporter permease component | - | NP_416632 | yehW | predicted transporter subunit: membrane component of ABC superfamily | GCO | 28.64 | 54.93 |
| NP_460454 | - | putative transport system permease component | - | NP_416632 | yehW | predicted transporter subunit: membrane component of ABC superfamily | nGCO | 39 | 54.71 |
| NP_461107 | yehW | putative ABC-type proline/glycine betaine transport system permease component | - | NP_416632 | yehW | predicted transporter subunit: membrane component of ABC superfamily | GCO | 64.19 | 59.56 |
| --=-- |  |  |  |  |  |  |  |  |  |
| NP_459166 | kdgT | 2-keto-3-deoxygluconate permease | - | NP_418345 | kdgT | 2-keto-3-deoxygluconate permease | nGCO | 34.47 | 57.54 |
| NP_459643 | - | 2-keto-3-deoxygluconate permease | - | NP_418345 | kdgT | 2-keto-3-deoxygluconate permease | nGCO | 31.44 | 52.3 |
| --=-- |  |  |  |  |  |  |  |  |  |
| NP_459344 | - | putative outer membrane lipoprotein | + | NP_418568 | yjeI | hypothetical protein | nGCO | 34.45 | 49.44 |
| NP_463195 | yjeI | putative outer membrane lipoprotein | - | NP_418568 | yjeI | hypothetical protein | GCO | 88.88 | 54.56 |
| --=-- |  |  |  |  |  |  |  |  |  |
| NP_459429 | apbA | 2-dehydropantoate 2-reductase | - | NP_414959 | panE | 2-dehydropantoate 2-reductase | GCO | 87.45 | 52.63 |
| NP_461508 | - | 2-dehydropantoate 2-reductase | + | NP_414959 | panE | 2-dehydropantoate 2-reductase | nGCO | 25.4 | 48.91 |
| --=-- |  |  |  |  |  |  |  |  |  |
| NP_459869 | - | putative integrase | - | NP_416096 | intQ | Qin prophage; predicted defective integrase | nGCO | 55.78 | 47.36 |
| NP_459980 | - | integrase | - | NP_416096 | intQ | Qin prophage; predicted defective integrase | nGCO | 54.19 | 48.8 |
| --=-- |  |  |  |  |  |  |  |  |  |
| NP_461100 | yehR | putative lipoprotein | - | NP_416627 | yehR | hypothetical protein | nGCO | 73.48 | 43.57 |
| NP_461101 | - | hypothetical protein | - | NP_416627 | yehR | hypothetical protein | GCO | 30.21 | 40.67 |
| --=-- |  |  |  |  |  |  |  |  |  |
| NP_461190 | ccmH | putative heme lyase subunit | - | NP_416698 | ccmH | heme lyase, CcmH subunit | GCO | 64.65 | 66.28 |
| NP_462712 | ccmH | putative heme lyase subunit | - | NP_416698 | ccmH | heme lyase, CcmH subunit | GCO | 63.29 | 67.81 |
| --=-- |  |  |  |  |  |  |  |  |  |
| NP_459423 | phnT | 2-aminoethylphosphonate transporter | - | NP_415376 | potG | putrescine transporter subunit: ATP-binding component of ABC superfamily | nGCO | 35.18 | 58.73 |
| NP_459708 | - | putative ABC-type polysaccharide/polyol phosphate transport system ATPase component | + | NP_415376 | potG | putrescine transporter subunit: ATP-binding component of ABC superfamily | nGCO | 26.63 | 39.66 |
| NP_459855 | potG | putrescine transporter | + | NP_415376 | potG | putrescine transporter subunit: ATP-binding component of ABC superfamily | GCO | 93.63 | 54.76 |
| --=-- |  |  |  |  |  |  |  |  |  |
| NP_461302 | - | putative diaminopimelate decarboxylase | - | NP_417315 | lysA | diaminopimelate decarboxylase, PLP-binding | nGCO | 26.81 | 49.78 |
| NP_461930 | lysA | diaminopimelate decarboxylase | - | NP_417315 | lysA | diaminopimelate decarboxylase, PLP-binding | GCO | 88.57 | 57.4 |
| --=-- |  |  |  |  |  |  |  |  |  |
| NP_460625 | - | putative thiol peroxidase | + | NP_415840 | tpx | thiol peroxidase | nGCO | 30.25 | 44.44 |
| NP_460640 | tpx | thiol peroxidase | - | NP_415840 | tpx | thiol peroxidase | GCO | 88.09 | 50.09 |
| --=-- |  |  |  |  |  |  |  |  |  |
| NP_459046 | - | putative glycosyl hydrolase | + | NP_418113 | yicI | predicted alpha-glucosidase | nGCO | 25.33 | 54.6 |
| NP_462649 | yicI | putative alpha xylosidase | - | NP_418113 | yicI | predicted alpha-glucosidase | GCO | 84.84 | 56.4 |
| --=-- |  |  |  |  |  |  |  |  |  |
| NP_460724 | ychP | putative invasin | - | NP_415738 | ychP | predicted invasin | GCO | 77.69 | 53.47 |
| NP_461452 | sinH | intimin-like protein | - | NP_415738 | ychP | predicted invasin | nGCO | 31.09 | 52.16 |
| --=-- |  |  |  |  |  |  |  |  |  |
| NP_460996 | pduP | CoA-dependent propionaldehyde dehydrogenase | - | NP_416950 | eutE | predicted aldehyde dehydrogenase, ethanolamine utilization protein | nGCO | 41.06 | 58.63 |
| NP_461398 | eutE | putative aldehyde oxidoreductase | - | NP_416950 | eutE | predicted aldehyde dehydrogenase, ethanolamine utilization protein | GCO | 89.93 | 59.9 |
| --=-- |  |  |  |  |  |  |  |  |  |
| NP_462171 | - | galactitol-specific enzyme IIA | - | NP_416597 | gatA | galactitol-specific enzyme IIA component of PTS | GCO | 60.66 | 54.19 |
| NP_462683 | - | putative phosphotransferase system mannitol/fructose-specific IIA domain | - | NP_416597 | gatA | galactitol-specific enzyme IIA component of PTS | GCO | 23.88 | 44.09 |
| --=-- |  |  |  |  |  |  |  |  |  |
| NP_460321 | ydiP | putative transcriptional regulator | + | NP_416211 | ydiP | predicted DNA-binding transcriptional regulator | GCO | 80.82 | 42.64 |
| NP_463284 | - | putative DNA-binding protein | - | NP_416211 | ydiP | predicted DNA-binding transcriptional regulator | nGCO | 27.6 | 44.12 |
| --=-- |  |  |  |  |  |  |  |  |  |
| NP_462695 | ilvB | acetolactate synthase large subunit | - | NP_418127 | ilvB | acetolactate synthase large subunit | GCO | 91.99 | 56.06 |
| NP_462792 | ilvG | acetolactate synthase II large subunit | - | NP_418127 | ilvB | acetolactate synthase large subunit | nGCO | 46.89 | 56.34 |
| --=-- |  |  |  |  |  |  |  |  |  |
| NP_459841 | deoR | deoxyribose operon transcriptional repressor | - | NP_415361 | deoR | DNA-binding transcriptional repressor | GCO | 83.33 | 51.91 |
| NP_462693 | - | putative regulatory protein | - | NP_415361 | deoR | DNA-binding transcriptional repressor | nGCO | 36.86 | 52.03 |
| --=-- |  |  |  |  |  |  |  |  |  |
| NP_462270 | argR | arginine repressor | - | NP_417704 | argR | arginine repressor | GCO | 94.87 | 49.68 |
| NP_463323 | - | putative arginine repressor | + | NP_417704 | argR | arginine repressor | nGCO | 33.83 | 47.85 |
| --=-- |  |  |  |  |  |  |  |  |  |
| NP_461710 | tctE | regulatory protein | - | NP_417498 | qseC | sensory histidine kinase in two-component regulatory system with QseB | GCO | 28.78 | 56.14 |
| NP_462093 | ygiY | putative sensor histidine kinase | - | NP_417498 | qseC | sensory histidine kinase in two-component regulatory system with QseB | GCO | 73.27 | 56.29 |
| --=-- |  |  |  |  |  |  |  |  |  |
| NP_459011 | yaaJ | putative alanine/glycine transport protein | - | NP_414548 | yaaJ | predicted transporter | GCO | 70.16 | 55.83 |
| NP_459978 | - | putative transcriptional regulator | + | NP_414548 | yaaJ | predicted transporter | nGCO | 26.94 | 43.07 |
| --=-- |  |  |  |  |  |  |  |  |  |
| NP_460763 | dsbB | disulfide bond formation protein B | - | NP_415703 | dsbB | disulfide bond formation protein B | GCO | 78.97 | 54.8 |
| NP_462109 | - | putative disulfide oxidoreductase | + | NP_415703 | dsbB | disulfide bond formation protein B | nGCO | 30.23 | 51.03 |
| --=-- |  |  |  |  |  |  |  |  |  |
| NP_461314 | - | hypothetical protein | - | NP_416825 | yfcJ | predicted transporter | GCO | 78.06 | 60.89 |
| NP_463289 | - | hypothetical protein | - | NP_416825 | yfcJ | predicted transporter | nGCO | 41.14 | 60.58 |
| --=-- |  |  |  |  |  |  |  |  |  |
| NP_460616 | - | putative methyl-accepting chemotaxis protein | - | NP_418775 | tsr | methyl-accepting chemotaxis protein I, serine sensor receptor | nGCO | 36.3 | 48.91 |
| NP_462067 | - | putative methyl-accepting chemotaxis protein | - | NP_418775 | tsr | methyl-accepting chemotaxis protein I, serine sensor receptor | nGCO | 34.17 | 57.17 |
| NP_462130 | - | putative methyl-accepting chemotaxis protein | - | NP_418775 | tsr | methyl-accepting chemotaxis protein I, serine sensor receptor | nGCO | 41.85 | 50.51 |
| NP_462478 | tcp | methyl-accepting transmembrane citrate/phenol chemoreceptor | - | NP_418775 | tsr | methyl-accepting chemotaxis protein I, serine sensor receptor | nGCO | 46.75 | 55.65 |
| NP_463392 | tsr | methyl-accepting chemotaxis protein I | - | NP_418775 | tsr | methyl-accepting chemotaxis protein I, serine sensor receptor | GCO | 73.82 | 55.17 |
| --=-- |  |  |  |  |  |  |  |  |  |
| NP_459768 | hutC | histidine utilization repressor | - | NP_415258 | mngR | DNA-binding transcriptional dual regulator, fatty-acyl-binding | nGCO | 24.77 | 56.88 |
| NP_462684 | - | putative regulatory protein | - | NP_415258 | mngR | DNA-binding transcriptional dual regulator, fatty-acyl-binding | nGCO | 29.23 | 40.86 |
| NP_462949 | - | putative regulatory protein | - | NP_415258 | mngR | DNA-binding transcriptional dual regulator, fatty-acyl-binding | nGCO | 27.92 | 51.03 |
| --=-- |  |  |  |  |  |  |  |  |  |
| NP_459564 | - | putative phosphosugar isomerase | - | NP_417830 | frlB | fructoselysine-6-P-deglycase | nGCO | 26.25 | 52.58 |
| NP_462502 | - | putative phosphosugar isomerase | - | NP_417830 | frlB | fructoselysine-6-P-deglycase | nGCO | 26.59 | 50.3 |
| NP_463399 | - | putative glucosamine-fructose-6-phosphate aminotransferase | - | NP_417830 | frlB | fructoselysine-6-P-deglycase | nGCO | 26.86 | 50.64 |
| --=-- |  |  |  |  |  |  |  |  |  |
| NP_460128 | yceI | hypothetical protein | - | NP_415574 | yceI | hypothetical protein | GCO | 84.29 | 50.34 |
| NP_460580 | - | putative periplasmic protein | + | NP_415574 | yceI | hypothetical protein | nGCO | 31.76 | 42.36 |
| --=-- |  |  |  |  |  |  |  |  |  |
| NP_461630 | - | DNA invertase-like protein | - | NP_415676 | pin | e14 prophage; site-specific DNA recombinase | nGCO | 80 | 50.35 |
| NP_461699 | hin | DNA-invertase Hin | - | NP_415676 | pin | e14 prophage; site-specific DNA recombinase | nGCO | 59.77 | 46.24 |
| --=-- |  |  |  |  |  |  |  |  |  |
| NP_459552 | rfbI | putative glycosyl translocase | - | NP_416851 | yfdG | CPS-53 (KpLE1) prophage; bactoprenol-linked glucose translocase (flippase) | GCO | 81.66 | 46.55 |
| NP_463071 | - | putative phage glucose translocase | + | NP_416851 | yfdG | CPS-53 (KpLE1) prophage; bactoprenol-linked glucose translocase (flippase) | GCO | 55.83 | 43.52 |
| --=-- |  |  |  |  |  |  |  |  |  |
| NP_460227 | - | putative inner membrane protein | - | NP_415846 | ynaI | conserved inner membrane protein | nGCO | 23.94 | 48.32 |
| NP_460622 | ynaI | putative integral membrane protein | - | NP_415846 | ynaI | conserved inner membrane protein | nGCO | 71.34 | 44.86 |
| --=-- |  |  |  |  |  |  |  |  |  |
| NP_462164 | - | putative sugar kinase | - | NP_417983 | kdgK | ketodeoxygluconokinase | nGCO | 24.14 | 48.2 |
| NP_462513 | kdgK | ketodeoxygluconokinase | - | NP_417983 | kdgK | ketodeoxygluconokinase | GCO | 92.53 | 56.45 |
| --=-- |  |  |  |  |  |  |  |  |  |
| NP_459058 | - | putative transcriptional regulator | - | NP_415152 | citA | sensory histidine kinase in two-component regulatory system with citB | GCO | 39.39 | 48.02 |
| NP_459617 | dpiB | sensory histidine kinase | - | NP_415152 | citA | sensory histidine kinase in two-component regulatory system with citB | GCO | 83.88 | 51.86 |
| --=-- |  |  |  |  |  |  |  |  |  |
| NP_459926 | - | SlsA | - | NP_415417 | ycaC | predicted hydrolase | nGCO | 46.66 | 53.83 |
| NP_462660 | slsA | putative inner membrane protein | + | NP_415417 | ycaC | predicted hydrolase | nGCO | 47.17 | 52.27 |
| --=-- |  |  |  |  |  |  |  |  |  |
| NP_459250 | yaeE | putative transport protein | - | NP_414740 | metI | DL-methionine transporter subunit | GCO | 76.03 | 53.36 |
| NP_459507 | sfbC | putative ABC transporter permease component | - | NP_414740 | metI | DL-methionine transporter subunit | GCO | 44.11 | 54.39 |
| --=-- |  |  |  |  |  |  |  |  |  |
| NP_459603 | - | putative oxidoreductase protein | - | NP_416104 | ynfE | oxidoreductase subunit | nGCO | 28.78 | 54.73 |
| NP_460459 | - | putative dimethyl sulphoxide reductase chain A1 | - | NP_416104 | ynfE | oxidoreductase subunit | GCO | 84.97 | 54.21 |
| NP_461010 | phsA | thiosulfate reductase precursor | - | NP_416104 | ynfE | oxidoreductase subunit | nGCO | 25.29 | 55.46 |
| --=-- |  |  |  |  |  |  |  |  |  |
| NP_461195 | ccmC | heme exporter protein | - | NP_416703 | ccmC | heme exporter subunit | GCO | 86.12 | 65.44 |
| NP_462717 | ccmC | heme exporter protein | - | NP_416703 | ccmC | heme exporter subunit | GCO | 86.12 | 65.44 |
| --=-- |  |  |  |  |  |  |  |  |  |
| NP_459527 | arcC | carbamate kinase | - | NP_415054 | ybcF | predicted carbamate kinase | GCO | 75.08 | 58.16 |
| NP_463326 | - | carbamate kinase | + | NP_415054 | ybcF | predicted carbamate kinase | nGCO | 52.18 | 59.69 |
| --=-- |  |  |  |  |  |  |  |  |  |
| NP_459251 | abc | putative transport protein | - | NP_414741 | metN | DL-methionine transporter subunit | GCO | 95.33 | 52.42 |
| NP_459506 | sfbB | putative ABC-type transport system ATPase component | - | NP_414741 | metN | DL-methionine transporter subunit | GCO | 45.18 | 56.14 |
| --=-- |  |  |  |  |  |  |  |  |  |
| NP_459433 | - | putative periplasmic protein | + | NP_415177 | ybeQ | hypothetical protein | nGCO | 25.6 | 43.71 |
| NP_459646 | ybeQ | tetratricopeptide repeat protein | + | NP_415177 | ybeQ | hypothetical protein | GCO | 49.84 | 47.48 |
| NP_460716 | - | tetratricopeptide repeat protein | - | NP_415177 | ybeQ | hypothetical protein | nGCO | 31.8 | 49.47 |
| --=-- |  |  |  |  |  |  |  |  |  |
| NP_461027 | rfbP | undecaprenol-phosphate galactosephosphotransferase/O-antigen transferase | - | NP_416551 | wcaJ | predicted UDP-glucose lipid carrier transferase | nGCO | 26.56 | 37.1 |
| NP_461048 | wcaJ | UDP-glucose lipid carrier transferase/glucose-1-phosphate transferase | - | NP_416551 | wcaJ | predicted UDP-glucose lipid carrier transferase | GCO | 83.4 | 53.18 |
| --=-- |  |  |  |  |  |  |  |  |  |
| NP_459025 | - | putative cytoplasmic protein | - | NP_414909 | yaiV | predicted DNA-binding transcriptional regulator | nGCO | 33.33 | 46.95 |
| NP_459369 | yaiV | putative inner membrane protein | - | NP_414909 | yaiV | predicted DNA-binding transcriptional regulator | GCO | 71.01 | 42.46 |
| NP_463452 | - | putative inner membrane protein | + | NP_414909 | yaiV | predicted DNA-binding transcriptional regulator | nGCO | 31.6 | 42.7 |
| --=-- |  |  |  |  |  |  |  |  |  |
| NP_461337 | pgtA | activator | + | NP_417049 | yfhA | predicted DNA-binding response regulator in two-component system | nGCO | 25.64 | 51.84 |
| NP_461497 | yfhA | putative transcriptional regulator | - | NP_417049 | yfhA | predicted DNA-binding response regulator in two-component system | GCO | 95.04 | 55.45 |
| --=-- |  |  |  |  |  |  |  |  |  |
| NP_460455 | ynfI | putative anaerobic dehydrogenase component | - | NP_416108 | dmsD | twin-argninine leader-binding protein for DmsA and TorA | GCO | 76.96 | 54.47 |
| NP_463173 | - | putative anaerobic dehydrogenase component | - | NP_416108 | dmsD | twin-argninine leader-binding protein for DmsA and TorA | GCO | 32.55 | 53.36 |
| --=-- |  |  |  |  |  |  |  |  |  |
| NP_460357 | ssrA | sensor kinase | + | NP_415513 | torS | hybrid sensory histidine kinase in two-component regulatory system with TorR | nGCO | 25.67 | 42.49 |
| NP_462726 | torS | sensor kinase | - | NP_415513 | torS | hybrid sensory histidine kinase in two-component regulatory system with TorR | GCO | 56.41 | 54.09 |
| --=-- |  |  |  |  |  |  |  |  |  |
| NP_460982 | pduF | propanediol diffusion facilitator | - | NP_418362 | glpF | glycerol facilitator | nGCO | 62.54 | 50.94 |
| NP_462968 | glpF | glycerol diffusion | - | NP_418362 | glpF | glycerol facilitator | GCO | 86.12 | 52.83 |
| --=-- |  |  |  |  |  |  |  |  |  |
| NP_459454 | - | putative cysteine synthase/cystathionine beta-synthase | - | NP_416916 | cysM | cysteine synthase B (O-acetylserine sulfhydrolase B) | nGCO | 25.96 | 54.64 |
| NP_461375 | cysM | cysteine synthase B | - | NP_416916 | cysM | cysteine synthase B (O-acetylserine sulfhydrolase B) | GCO | 88.77 | 57.12 |
| --=-- |  |  |  |  |  |  |  |  |  |
| NP_460635 | - | putative transcriptional regulator | - | NP_417978 | yhjC | predicted DNA-binding transcriptional regulator | nGCO | 35.51 | 54.74 |
| NP_462508 | yhjC | putative transcriptional regulator | - | NP_417978 | yhjC | predicted DNA-binding transcriptional regulator | GCO | 66.21 | 53.55 |
| --=-- |  |  |  |  |  |  |  |  |  |
| NP_459917 | ybjX | VirK-like protein | - | NP_415398 | ybjX | hypothetical protein | GCO | 57.14 | 46.85 |
| NP_461707 | virK | virulence protein | + | NP_415398 | ybjX | hypothetical protein | nGCO | 39.08 | 44.51 |
| --=-- |  |  |  |  |  |  |  |  |  |
| NP_459047 | - | putative sodium galactoside symporter | + | NP_418313 | yihP | predicted transporter | nGCO | 26.74 | 49.05 |
| NP_462898 | yihP | putative GPH family transport protein | - | NP_418313 | yihP | predicted transporter | GCO | 88.59 | 52.63 |
| --=-- |  |  |  |  |  |  |  |  |  |
| NP_461711 | tctD | regulatory protein | - | NP_417497 | qseB | DNA-binding response regulator in two-component regulatory system with QseC | GCO | 38.81 | 54.96 |
| NP_462092 | ygiX | putative transcriptional regulator | - | NP_417497 | qseB | DNA-binding response regulator in two-component regulatory system with QseC | GCO | 82.56 | 56.21 |
| --=-- |  |  |  |  |  |  |  |  |  |
| NP_462904 | yihV | putative sugar kinase | - | NP_418319 | yihV | predicted sugar kinase | GCO | 79.86 | 56.69 |
| NP_462947 | - | aminoimidazole riboside kinase | - | NP_418319 | yihV | predicted sugar kinase | nGCO | 27.36 | 58.02 |
| --=-- |  |  |  |  |  |  |  |  |  |
| NP_459817 | ybiV(2) | putative hydrolase | - | NP_415343 | ybiV | predicted hydrolase | GCO | 93.67 | 46.8 |
| NP_459819 | ybiV(1) | putative hydrolase | - | NP_415343 | ybiV | predicted hydrolase | GCO | 88.84 | 49.38 |
| --=-- |  |  |  |  |  |  |  |  |  |
| NP_460466 | rspB | putative dehydrogenase | - | NP_416097 | rspB | predicted oxidoreductase, Zn-dependent and NAD(P)-binding | GCO | 75.22 | 51.56 |
| NP_460502 | - | putative zinc-binding dehydrogenase | + | NP_416097 | rspB | predicted oxidoreductase, Zn-dependent and NAD(P)-binding | nGCO | 31.17 | 47.66 |
| --=-- |  |  |  |  |  |  |  |  |  |
| NP_460850 | znuB | high-affinity Zn transport protein | - | NP_416373 | znuB | high-affinity zinc transporter membrane component | GCO | 76.24 | 55.47 |
| NP_461784 | sitC | putative permease | - | NP_416373 | znuB | high-affinity zinc transporter membrane component | nGCO | 24.68 | 54.81 |
| NP_461785 | sitD | putative permease | - | NP_416373 | znuB | high-affinity zinc transporter membrane component | nGCO | 22.22 | 50.53 |
| --=-- |  |  |  |  |  |  |  |  |  |
| NP_459269 | - | putative chaperone ATPase | - | NP_417083 | clpB | protein disaggregation chaperone | nGCO | 37.11 | 59.54 |
| NP_461591 | clpB | ATP-dependent protease | - | NP_417083 | clpB | protein disaggregation chaperone | GCO | 92.41 | 53.3 |
| --=-- |  |  |  |  |  |  |  |  |  |
| NP_461843 | - | putative 3-polyprenyl-4-hydroxybenzoate decarboxylase | - | NP_418285 | ubiD | 3-octaprenyl-4-hydroxybenzoate decarboxylase | nGCO | 27.3 | 55.53 |
| NP_462863 | yigC | putative oxidoreductase | - | NP_418285 | ubiD | 3-octaprenyl-4-hydroxybenzoate decarboxylase | GCO | 95.92 | 53.82 |
| --=-- |  |  |  |  |  |  |  |  |  |
| NP_461035 | rfbH | CDP-6-deoxy-D-xylo-4-hexulose-3-dehydrase | - | NP_416756 | yfbE | uridine 5'-(beta-1-threo-pentapyranosyl-4-ulose diphosphate) aminotransferase, PLP-dependent | nGCO | 27.62 | 44.52 |
| NP_461239 | yfbE | 4-amino-4-deoxy-L-arabinose lipopolysaccharide-modifying enzyme | - | NP_416756 | yfbE | uridine 5'-(beta-1-threo-pentapyranosyl-4-ulose diphosphate) aminotransferase, PLP-dependent | GCO | 79.48 | 56.21 |
| --=-- |  |  |  |  |  |  |  |  |  |
| NP_459355 | - | cytochrome BD2 subunit I | - | NP_415497 | appC | cytochrome bd-II oxidase, subunit I | nGCO | 32.81 | 55.41 |
| NP_460748 | - | putative cytochrome oxidase subunit I | - | NP_415497 | appC | cytochrome bd-II oxidase, subunit I | GCO | 86.9 | 55.53 |
| --=-- |  |  |  |  |  |  |  |  |  |
| NP_459763 | ybhA | putative hydrolase | - | NP_415287 | ybhA | predicted hydrolase | GCO | 84.19 | 53.23 |
| NP_462890 | - | putative hydrolase | - | NP_415287 | ybhA | predicted hydrolase | nGCO | 34.45 | 47.34 |
| --=-- |  |  |  |  |  |  |  |  |  |
| NP_461234 | yfaX | putative transcriptional regulator | - | NP_416751 | yfaX | predicted DNA-binding transcriptional regulator | GCO | 87.3 | 51.46 |
| NP_462434 | - | putative transcriptional regulator | - | NP_416751 | yfaX | predicted DNA-binding transcriptional regulator | nGCO | 38.8 | 50.52 |
| --=-- |  |  |  |  |  |  |  |  |  |
| NP_460491 | - | putative hydrogenase | - | NP_417465 | hybF | protein involved with the maturation of hydrogenases 1 and 2 | nGCO | 38.05 | 50.58 |
| NP_462059 | hypA | hydrogenase nickel incorporation protein | - | NP_417465 | hybF | protein involved with the maturation of hydrogenases 1 and 2 | GCO | 84.95 | 55.55 |
| --=-- |  |  |  |  |  |  |  |  |  |
| NP_462583 | selA | selenocysteine synthase | - | NP_418048 | selA | selenocysteine synthase | GCO | 78.4 | 59.33 |
| NP_462667 | - | putative selenocysteine synthase | - | NP_418048 | selA | selenocysteine synthase | nGCO | 27.56 | 57.92 |
| NP_463307 | - | putative selenocysteine synthase | - | NP_418048 | selA | selenocysteine synthase | nGCO | 21.47 | 56.47 |
| --=-- |  |  |  |  |  |  |  |  |  |
| NP_460517 | - | putative glycosyl hydrolase | - | NP_417889 | glgX | glycogen debranching enzyme | nGCO | 46.39 | 52.11 |
| NP_460519 | - | putative alpha amylase | - | NP_417889 | glgX | glycogen debranching enzyme | nGCO | 27.25 | 54.34 |
| NP_462438 | glgX | glycogen debranching enzyme | - | NP_417889 | glgX | glycogen debranching enzyme | GCO | 85.38 | 57.2 |
| --=-- |  |  |  |  |  |  |  |  |  |
| NP_459745 | - | putative cation transporter | - | NP_417959 | arsB | arsenite/antimonite transporter | nGCO | 20.96 | 38.06 |
| NP_462266 | - | putative cation transporter | - | NP_417959 | arsB | arsenite/antimonite transporter | nGCO | 21.82 | 53.11 |
| --=-- |  |  |  |  |  |  |  |  |  |
| NP_461000 | pduU | polyhedral body protein | - | NP_416957 | ypfE | predicted carboxysome structural protein with predicted role in ethanol utilization | GCO | 56.88 | 55.55 |
| NP_461405 | eutS | putative carboxysome structural protein | - | NP_416957 | ypfE | predicted carboxysome structural protein with predicted role in ethanol utilization | GCO | 94.59 | 50.89 |
| --=-- |  |  |  |  |  |  |  |  |  |
| NP_459388 | yajF | putative sugar kinase/transcriptional regulator | - | NP_414928 | mak | fructokinase | GCO | 88.66 | 58.08 |
| NP_462581 | - | putative transcriptional regulator | - | NP_414928 | mak | fructokinase | nGCO | 22.4 | 56.8 |
| --=-- |  |  |  |  |  |  |  |  |  |
| NP_461192 | ccmF | cytochrome c-type biogenesis protein | - | NP_416700 | ccmF | heme lyase, CcmF subunit | GCO | 76.62 | 68.16 |
| NP_462714 | ccmF | cytochrome c-type biogenesis protein | - | NP_416700 | ccmF | heme lyase, CcmF subunit | GCO | 76.62 | 68.16 |
| --=-- |  |  |  |  |  |  |  |  |  |
| NP_459990 | - | DNA replication protein DnaC | - | NP_415878 | ydaV | Rac prophage; predicted DNA replication protein | nGCO | 49.39 | 52.53 |
| NP_461560 | - | DNA replication protein DnaC | - | NP_415878 | ydaV | Rac prophage; predicted DNA replication protein | nGCO | 49.39 | 52.53 |
| --=-- |  |  |  |  |  |  |  |  |  |
| NP_461197 | ccmA | heme exporter protein | - | NP_416705 | ccmA | heme exporter subunit | GCO | 64.28 | 72.65 |
| NP_462719 | ccmA | heme exporter protein | - | NP_416705 | ccmA | heme exporter subunit | GCO | 64.28 | 72.65 |
| --=-- |  |  |  |  |  |  |  |  |  |
| NP_460451 | - | proline/glycine betaine transport systems | - | NP_416633 | yehX | predicted transporter subunit: ATP-binding component of ABC superfamily | GCO | 41.91 | 53.08 |
| NP_461108 | yehX | putative ABC-type proline/glycine betaine transport system ATPase component | - | NP_416633 | yehX | predicted transporter subunit: ATP-binding component of ABC superfamily | GCO | 87.62 | 58.01 |
| --=-- |  |  |  |  |  |  |  |  |  |
| NP_463004 | oxyR | oxidative stress regulatory protein | - | NP_418396 | oxyR | DNA-binding transcriptional dual regulator | GCO | 95.73 | 56.53 |
| NP_463135 | - | putative transcriptional regulator | - | NP_418396 | oxyR | DNA-binding transcriptional dual regulator | nGCO | 28.26 | 56.08 |
| --=-- |  |  |  |  |  |  |  |  |  |
| NP_459644 | - | putative sigma-54 dependent transcriptional regulator | - | NP_415839 | tyrR | DNA-binding transcriptional dual regulator, tyrosine-binding | nGCO | 32.35 | 50.07 |
| NP_460641 | tyrR | transcriptional regulator | - | NP_415839 | tyrR | DNA-binding transcriptional dual regulator, tyrosine-binding | GCO | 85.18 | 53.56 |
| --=-- |  |  |  |  |  |  |  |  |  |
| NP_459551 | yfdH | putative glycosyltransferase | - | NP_416852 | yfdH | CPS-53 (KpLE1) prophage; bactoprenol glucosyl transferase | GCO | 80.71 | 42.28 |
| NP_463070 | - | putative phage glycosyltransferase | + | NP_416852 | yfdH | CPS-53 (KpLE1) prophage; bactoprenol glucosyl transferase | GCO | 75.73 | 43.76 |
| --=-- |  |  |  |  |  |  |  |  |  |
| NP_459326 | - | putative fumarylacetoacetate hydrolase | - | NP_415698 | ycgM | predicted isomerase/hydrolase | nGCO | 33.81 | 49.82 |
| NP_460768 | ycgM | putative fumarylacetoacetate hydrolase | - | NP_415698 | ycgM | predicted isomerase/hydrolase | GCO | 84.93 | 53.18 |
| NP_461122 | - | putative flutathione S-transferase | - | NP_415698 | ycgM | predicted isomerase/hydrolase | nGCO | 37.43 | 54.55 |
| --=-- |  |  |  |  |  |  |  |  |  |
| NP_459377 | - | putative permease | - | NP_415571 | mdtG | predicted drug efflux system | nGCO | 56.81 | 52.39 |
| NP_460125 | yceE | putative transport protein | - | NP_415571 | mdtG | predicted drug efflux system | GCO | 74.93 | 54.89 |
| --=-- |  |  |  |  |  |  |  |  |  |
| NP_459348 | - | putative cation transport ATPase | - | NP_415017 | copA | copper transporter | nGCO | 42.04 | 60.02 |
| NP_459493 | copA | putative copper-transporting ATPase | - | NP_415017 | copA | copper transporter | nGCO | 91.47 | 59.15 |
| --=-- |  |  |  |  |  |  |  |  |  |
| NP_459338 | - | hypothetical protein | + | NP_416681 | rtn | hypothetical protein | nGCO | 38.72 | 48.44 |
| NP_461159 | rtn | hypothetical protein | - | NP_416681 | rtn | hypothetical protein | GCO | 68.72 | 51.89 |
| --=-- |  |  |  |  |  |  |  |  |  |
| NP_462596 | - | putative transcriptional regulator | - | NP_418685 | idnR | DNA-binding transcriptional repressor, 5-gluconate-binding | nGCO | 28.75 | 55.22 |
| NP_463341 | idnR | L-idonate regulator | - | NP_418685 | idnR | DNA-binding transcriptional repressor, 5-gluconate-binding | GCO | 89.15 | 50.35 |
| --=-- |  |  |  |  |  |  |  |  |  |
| NP_460984 | pudB | polyhedral body protein | - | NP_416934 | eutL | predicted carboxysome structural protein with predicted role in ethanolamine utilization | nGCO | 29.77 | 60.39 |
| NP_461391 | eutL | putative carboxysome structural protein | - | NP_416934 | eutL | predicted carboxysome structural protein with predicted role in ethanolamine utilization | GCO | 94.06 | 59.39 |
| --=-- |  |  |  |  |  |  |  |  |  |
| NP_459741 | - | fumarate hydratase | + | NP_417534 | ttdB | L(+)-tartrate dehydratase | GCO | 30.27 | 42.49 |
| NP_462264 | - | L(+)-tartrate dehydratase | - | NP_417534 | ttdB | L(+)-tartrate dehydratase | GCO | 57.63 | 46.6 |
| --=-- |  |  |  |  |  |  |  |  |  |
| NP_459249 | yaeC | putative outer membrane lipoprotein | - | NP_414739 | metQ | DL-methionine transporter subunit | GCO | 91.88 | 48.77 |
| NP_459505 | sfbA | putative ABC-type transport system ATPase component | - | NP_414739 | metQ | DL-methionine transporter subunit | nGCO | 37.34 | 52.58 |
| --=-- |  |  |  |  |  |  |  |  |  |
| NP_461028 | rfbK | phosphomannomutase | - | NP_417643 | glmM | phosphoglucosamine mutase | nGCO | 31.51 | 40.86 |
| NP_462205 | mrsA | phosphoglucosamine mutase | - | NP_417643 | glmM | phosphoglucosamine mutase | GCO | 84.94 | 57.24 |
| --=-- |  |  |  |  |  |  |  |  |  |
| NP_460514 | - | putative transcriptional regulator | - | NP_417314 | galR | DNA-binding transcriptional repressor | nGCO | 42.12 | 53.96 |
| NP_461928 | galR | galETK operon transcriptional repressor | - | NP_417314 | galR | DNA-binding transcriptional repressor | GCO | 88.33 | 55.87 |
| --=-- |  |  |  |  |  |  |  |  |  |
| NP_459080 | fixA | putative electron transfer flavoprotein subunit beta | - | NP_414583 | fixA | predicted electron transfer flavoprotein subunit, ETFP adenine nucleotide-binding domain | GCO | 82.42 | 52.01 |
| NP_459832 | - | putative electron transfer protein beta subunit | + | NP_414583 | fixA | predicted electron transfer flavoprotein subunit, ETFP adenine nucleotide-binding domain | nGCO | 27.42 | 37.77 |
| --=-- |  |  |  |  |  |  |  |  |  |
| NP_459089 | - | putative sulfatase | + | NP_418134 | yidJ | predicted sulfatase/phosphatase | nGCO | 22.55 | 46.19 |
| NP_459862 | - | putative sulfatase | + | NP_418134 | yidJ | predicted sulfatase/phosphatase | nGCO | 24.4 | 51.88 |
| NP_462037 | - | putative arylsulfatase | - | NP_418134 | yidJ | predicted sulfatase/phosphatase | nGCO | 26.15 | 48.56 |
| --=-- |  |  |  |  |  |  |  |  |  |
| NP_462165 | agaR | Aga operon transcriptional repressor | - | NP_417600 | agaR | DNA-binding transcriptional dual regulator | nGCO | 26.93 | 51.79 |
| NP_462175 | - | galactitol utilization operon transcriptional repressor | - | NP_417600 | agaR | DNA-binding transcriptional dual regulator | nGCO | 35.82 | 50.38 |
| --=-- |  |  |  |  |  |  |  |  |  |
| NP_461657 | - | DNA adenine methylase-like protein | - | NP_417846 | dam | DNA adenine methylase | nGCO | 46.92 | 46.15 |
| NP_462387 | dam | DNA adenine methylase | - | NP_417846 | dam | DNA adenine methylase | GCO | 92.08 | 49.1 |
| --=-- |  |  |  |  |  |  |  |  |  |
| NP_459063 | citC2 | putative citrate lyase synthetase | - | NP_415151 | citC | citrate lyase synthetase | GCO | 46.38 | 53.73 |
| NP_459616 | citC | citrate lyase synthetase | - | NP_415151 | citC | citrate lyase synthetase | GCO | 86.89 | 51.62 |
| --=-- |  |  |  |  |  |  |  |  |  |
| NP_461029 | rfbM | mannose-1-phosphate guanylyltransferase | - | NP_416553 | cpsB | mannose-1-phosphate guanyltransferase | nGCO | 57.35 | 40.27 |
| NP_461050 | manC | mannose-1-phosphate guanylyltransferase | + | NP_416553 | cpsB | mannose-1-phosphate guanyltransferase | GCO | 85.56 | 60.61 |
| --=-- |  |  |  |  |  |  |  |  |  |
| NP_459121 | ilvI | acetolactate synthase III large subunit | - | YP_025294 | ilvI | acetolactate synthase III large subunit | GCO | 89.51 | 54.69 |
| NP_461346 | - | indolepyruvate decarboxylase | - | YP_025294 | ilvI | acetolactate synthase III large subunit | nGCO | 22.85 | 59.1 |
| --=-- |  |  |  |  |  |  |  |  |  |
| NP_459605 | - | putative hydrogenase protein | - | NP_416107 | ynfH | oxidoreductase, membrane subunit | nGCO | 28.5 | 55.07 |
| NP_460456 | - | putative dimethylsulfoxide reductase | - | NP_416107 | ynfH | oxidoreductase, membrane subunit | GCO | 74.73 | 56.87 |
| NP_461463 | - | putative dimethylsulfoxide reductase | - | NP_416107 | ynfH | oxidoreductase, membrane subunit | nGCO | 29.28 | 60.49 |
| NP_463172 | - | putative anaerobic dimethylsulfoxide reductase subunit C | - | NP_416107 | ynfH | oxidoreductase, membrane subunit | GCO | 36.84 | 61.49 |
| --=-- |  |  |  |  |  |  |  |  |  |
| NP_459127 | ftsI | division specific transpeptidase | - | NP_414626 | ftsI | transpeptidase involved in septal peptidoglycan synthesis (penicillin-binding protein 3) | GCO | 96.25 | 54.83 |
| NP_460792 | - | putative penicillin-binding protein 3 | - | NP_414626 | ftsI | transpeptidase involved in septal peptidoglycan synthesis (penicillin-binding protein 3) | nGCO | 59.33 | 52.74 |
| --=-- |  |  |  |  |  |  |  |  |  |
| NP_460544 | - | putative outer membrane lipoprotein | - | NP_417310 | ygdR | hypothetical protein | nGCO | 45.07 | 43.69 |
| NP_461924 | ygdR | putative peptide transport protein | - | NP_417310 | ygdR | hypothetical protein | GCO | 90.27 | 47.03 |
| --=-- |  |  |  |  |  |  |  |  |  |
| NP_461215 | - | putative dehydratase | + | YP_026237 | dgoD | galactonate dehydratase | nGCO | 28.23 | 43.22 |
| NP_462728 | dgoA | 2-oxo-3-deoxygalactonate 6-phosphate aldolase/galactonate dehydratase | - | YP_026237 | dgoD | galactonate dehydratase | GCO | 92.4 | 55.17 |
| NP_462733 | - | putative mandelate racemase | - | YP_026237 | dgoD | galactonate dehydratase | nGCO | 29.72 | 54.35 |
| --=-- |  |  |  |  |  |  |  |  |  |
| NP_461287 | - | putative transcriptional regulator | - | YP_026222 | gntR | DNA-binding transcriptional repressor | nGCO | 34.42 | 55.68 |
| NP_462444 | gntR | gluconate operon transcriptional repressor | - | YP_026222 | gntR | DNA-binding transcriptional repressor | GCO | 92.14 | 57.32 |
| --=-- |  |  |  |  |  |  |  |  |  |
| NP_461880 | - | putative glycerate kinase 2 | - | NP_417593 | garK | glycerate kinase I | nGCO | 65.87 | 58.09 |
| NP_462160 | garK | glycerate kinase | - | NP_417593 | garK | glycerate kinase I | GCO | 78.21 | 55.06 |
| --=-- |  |  |  |  |  |  |  |  |  |
| NP_459935 | lrp | leucine-responsive regulatory protein | - | NP_415409 | lrp | DNA-binding transcriptional dual regulator, leucine-binding | GCO | 99.39 | 49.09 |
| NP_459976 | - | putative leucine response regulator | - | NP_415409 | lrp | DNA-binding transcriptional dual regulator, leucine-binding | nGCO | 31.12 | 48.91 |
| --=-- |  |  |  |  |  |  |  |  |  |
| NP_462919 | - | putative inner membrane lipoprotein | + | NP_418332 | yiiG | hypothetical protein | GCO | 30.87 | 44.76 |
| NP_462920 | yiiG | putative cytoplasmic protein | - | NP_418332 | yiiG | hypothetical protein | nGCO | 63.86 | 45.89 |
| --=-- |  |  |  |  |  |  |  |  |  |
| NP_462884 | hemN | coproporphyrinogen III oxidase | - | NP_418303 | hemN | coproporphyrinogen III oxidase | GCO | 92.56 | 51.74 |
| NP_462892 | - | coproporphyrinogen III oxidase | - | NP_418303 | hemN | coproporphyrinogen III oxidase | nGCO | 28.08 | 47.98 |
| --=-- |  |  |  |  |  |  |  |  |  |
| NP_461286 | - | putative phosphotransferase system enzyme II A component | - | NP_418616 | ulaC | L-ascorbate-specific enzyme IIA component of PTS | nGCO | 37.68 | 49.77 |
| NP_463246 | ptxA | putative PTS enzyme II | - | NP_418616 | ulaC | L-ascorbate-specific enzyme IIA component of PTS | GCO | 96.75 | 54.83 |
| --=-- |  |  |  |  |  |  |  |  |  |
| NP_459196 | fhuA | outer membrane ferrichrome receptor protein precursor | - | NP_414692 | fhuA | ferrichrome outer membrane transporter | GCO | 71.35 | 51.05 |
| NP_459359 | foxA | ferrioxamine receptor | - | NP_414692 | fhuA | ferrichrome outer membrane transporter | nGCO | 35.8 | 52.72 |
| --=-- |  |  |  |  |  |  |  |  |  |
| NP_459167 | - | putative inner membrane protein | - | NP_417217 | ygbK | hypothetical protein | nGCO | 25.62 | 57.78 |
| NP_461838 | ygbK | putative tRNA synthase | - | NP_417217 | ygbK | hypothetical protein | GCO | 79.42 | 59.46 |
| --=-- |  |  |  |  |  |  |  |  |  |
| NP_461996 | - | putative mannitol dehydrogenase | + | NP_417688 | yhcH | hypothetical protein | nGCO | 34 | 40.92 |
| NP_462245 | yhcH | putative cytoplasmic protein | - | NP_417688 | yhcH | hypothetical protein | GCO | 74.02 | 53.41 |
| --=-- |  |  |  |  |  |  |  |  |  |
| NP_461720 | ygaE | putative transcriptional repressor | - | NP_417150 | csiR | DNA-binding transcriptional dual regulator | GCO | 87.96 | 56.78 |
| NP_462268 | - | putative regulatory protein | - | NP_417150 | csiR | DNA-binding transcriptional dual regulator | nGCO | 22.5 | 49.04 |
| --=-- |  |  |  |  |  |  |  |  |  |
| NP_461298 | ubiX | 3-octaprenyl-4-hydroxybenzoate carboxy-lyase | - | NP_416814 | ubiX | 3-octaprenyl-4-hydroxybenzoate carboxy-lyase | GCO | 91 | 55.08 |
| NP_461842 | - | putative flavoprotein | - | NP_416814 | ubiX | 3-octaprenyl-4-hydroxybenzoate carboxy-lyase | nGCO | 55.91 | 57.74 |
| --=-- |  |  |  |  |  |  |  |  |  |
| NP_459940 | dmsA | anaerobic dimethyl sulfoxide reductase subunit A | - | NP_415414 | dmsA | dimethyl sulfoxide reductase, anaerobic, subunit A | GCO | 92.25 | 53.53 |
| NP_461465 | - | putative anaerobic dimethylsulfoxide reductase | - | NP_415414 | dmsA | dimethyl sulfoxide reductase, anaerobic, subunit A | GCO | 41.9 | 54.61 |
| NP_463170 | - | putative anaerobic dimethylsulfoxide reductase subunit A | - | NP_415414 | dmsA | dimethyl sulfoxide reductase, anaerobic, subunit A | GCO | 50.62 | 55.59 |
| --=-- |  |  |  |  |  |  |  |  |  |
| NP_460105 | - | putative dehydrogenase | + | NP_418700 | yjhC | KpLE2 phage-like element; predicted oxidoreductase | GCO | 59.56 | 46.52 |
| NP_461680 | - | putative dehydrogenase | - | NP_418700 | yjhC | KpLE2 phage-like element; predicted oxidoreductase | nGCO | 23.85 | 59.66 |
| NP_463286 | - | putative dehydrogenase | + | NP_418700 | yjhC | KpLE2 phage-like element; predicted oxidoreductase | nGCO | 24.74 | 49.06 |
| NP_463294 | - | myo-inositol 2-dehydrogenase | - | NP_418700 | yjhC | KpLE2 phage-like element; predicted oxidoreductase | GCO | 24.03 | 46.52 |
| --=-- |  |  |  |  |  |  |  |  |  |
| NP_459039 | - | putative outer membrane/exported protein | - | NP_416903 | yfeN | conserved outer membrane protein | nGCO | 34.71 | 44.22 |
| NP_461358 | yfeN | putative outer membrane protein | - | NP_416903 | yfeN | conserved outer membrane protein | GCO | 79.41 | 45.8 |
| --=-- |  |  |  |  |  |  |  |  |  |
| NP_459485 | aes | acetyl esterase | - | NP_415009 | aes | acetyl esterase | GCO | 69.9 | 56.27 |
| NP_462912 | - | putative acetyl esterase | - | NP_415009 | aes | acetyl esterase | nGCO | 31.41 | 55.8 |
| --=-- |  |  |  |  |  |  |  |  |  |
| NP_460292 | pfkB | 6-phosphofructokinase II | - | NP_416237 | pfkB | 6-phosphofructokinase II | GCO | 90.25 | 57.12 |
| NP_462167 | - | putative fructose-1-phosphate kinase | - | NP_416237 | pfkB | 6-phosphofructokinase II | nGCO | 28.31 | 43.27 |
| --=-- |  |  |  |  |  |  |  |  |  |
| NP_462384 | trpS | tryptophanyl-tRNA synthetase | - | NP_417843 | trpS | tryptophanyl-tRNA synthetase | GCO | 96.7 | 51.84 |
| NP_463367 | trpS2 | tryptophanyl-tRNA synthetase | - | NP_417843 | trpS | tryptophanyl-tRNA synthetase | nGCO | 26.42 | 52.95 |
| --=-- |  |  |  |  |  |  |  |  |  |
| NP_459863 | artJ | arginine transport system component | - | NP_415381 | artJ | arginine transporter subunit | GCO | 84.36 | 48.63 |
| NP_463215 | - | putative arginine-binding periplasmic protein | - | NP_415381 | artJ | arginine transporter subunit | nGCO | 59.45 | 49.12 |
| --=-- |  |  |  |  |  |  |  |  |  |
| NP_462172 | - | PTS family galactitol-specific enzyme IIB | - | NP_416596 | gatB | galactitol-specific enzyme IIB component of PTS | GCO | 77.65 | 49.12 |
| NP_462682 | - | putative periplasmic protein | + | NP_416596 | gatB | galactitol-specific enzyme IIB component of PTS | GCO | 37.23 | 38.88 |
| --=-- |  |  |  |  |  |  |  |  |  |
| NP_459038 | - | putative 5'-nucleotidase | + | NP_415013 | ushA | UDP-sugar hydrolase | nGCO | 24.48 | 46.81 |
| NP_459489 | ushA | UDP-sugar hydrolase/5'-nucleotidase | - | NP_415013 | ushA | UDP-sugar hydrolase | GCO | 85.97 | 51.72 |
| NP_462985 | - | putative 5'-nucleotidase/2',3'-cyclic phosphodiesterase | - | NP_415013 | ushA | UDP-sugar hydrolase | nGCO | 23.54 | 51.63 |
| --=-- |  |  |  |  |  |  |  |  |  |
| NP_461038 | rfbI | CDP-6-deoxy-delta-3,4-glucoseen reductase | - | NP_418286 | fre | NAD(P)H-flavin reductase | nGCO | 29.07 | 40.48 |
| NP_462864 | ubiB | NAD(P)H-flavin reductase | - | NP_418286 | fre | NAD(P)H-flavin reductase | GCO | 90.55 | 52.99 |
| --=-- |  |  |  |  |  |  |  |  |  |
| NP_460498 | - | putative hydrogenase-1 large subunit | - | NP_415492 | hyaB | hydrogenase 1, large subunit | GCO | 66.33 | 52.63 |
| NP_460743 | - | hydrogenase-1 large subunit | - | NP_415492 | hyaB | hydrogenase 1, large subunit | GCO | 91.12 | 55.18 |
| --=-- |  |  |  |  |  |  |  |  |  |
| NP_459068 | citG2 | triphosphoribosyl-dephospho-CoA synthase | - | NP_415146 | citG | triphosphoribosyl-dephospho-CoA transferase | GCO | 44.75 | 54.78 |
| NP_459611 | citG | triphosphoribosyl-dephospho-CoA synthase | - | NP_415146 | citG | triphosphoribosyl-dephospho-CoA transferase | GCO | 71.23 | 60.64 |
| NP_459748 | - | putative cytoplasmic protein | - | NP_415146 | citG | triphosphoribosyl-dephospho-CoA transferase | nGCO | 30.23 | 59.65 |
| --=-- |  |  |  |  |  |  |  |  |  |
| NP_462085 | - | putative inner membrane protein | - | NP_418034 | yiaM | predicted transporter | GCO | 24.3 | 47.41 |
| NP_462571 | yiaM | putative transporter | - | NP_418034 | yiaM | predicted transporter | GCO | 70.06 | 46.62 |
| NP_462934 | - | putative C4-dicarboxylate transport system | - | NP_418034 | yiaM | predicted transporter | GCO | 25.97 | 50.58 |
| --=-- |  |  |  |  |  |  |  |  |  |
| NP_459834 | - | putative acyl-CoA dehydrogenase | + | NP_416210 | ydiO | predicted acyl-CoA dehydrogenase | nGCO | 28.06 | 43.12 |
| NP_460322 | ydiO | crotonobetainyl-CoA dehydrogenase | + | NP_416210 | ydiO | predicted acyl-CoA dehydrogenase | GCO | 95.3 | 49.91 |
| --=-- |  |  |  |  |  |  |  |  |  |
| NP_460325 | aroE | shikimate 5-dehydrogenase | + | NP_416207 | ydiB | quinate/shikimate 5-dehydrogenase, NAD(P)-binding | GCO | 85.76 | 48.21 |
| NP_462758 | aroE | shikimate 5-dehydrogenase | - | NP_416207 | ydiB | quinate/shikimate 5-dehydrogenase, NAD(P)-binding | nGCO | 27.71 | 52.74 |
| --=-- |  |  |  |  |  |  |  |  |  |
| NP_461196 | ccmB | heme exporter protein | - | NP_416704 | ccmB | heme exporter subunit | GCO | 48.4 | 70.15 |
| NP_462718 | ccmB | heme exporter protein | - | NP_416704 | ccmB | heme exporter subunit | GCO | 48.4 | 70.15 |
| --=-- |  |  |  |  |  |  |  |  |  |
| NP_460387 | ssaU | type III secretion system apparatus protein | + | NP_416394 | flhB | flagellar biosynthesis protein B | nGCO | 29.71 | 39.66 |
| NP_460871 | flhB | flagellar biosynthesis protein | - | NP_416394 | flhB | flagellar biosynthesis protein B | GCO | 80.05 | 55.9 |
| NP_461808 | spaS | type III secretion protein | - | NP_416394 | flhB | flagellar biosynthesis protein B | nGCO | 21.47 | 43.32 |
| --=-- |  |  |  |  |  |  |  |  |  |
| NP_460386 | ssaT | type III secretion system apparatus protein | + | NP_416460 | fliR | flagellar biosynthesis protein R | nGCO | 25.13 | 38.97 |
| NP_460934 | fliR | flagellar biosynthesis protein | - | NP_416460 | fliR | flagellar biosynthesis protein R | GCO | 81.15 | 51.57 |
| NP_461809 | spaR | needle complex export protein | - | NP_416460 | fliR | flagellar biosynthesis protein R | nGCO | 19.62 | 49.36 |
| --=-- |  |  |  |  |  |  |  |  |  |
| NP_459860 | ulaA | ascorbate-specific PTS system enzyme IIC | - | NP_418614 | ulaA | ascorbate-specific PTS system enzyme IIC | GCO | 37.81 | 45.61 |
| NP_461284 | ulaA | ascorbate-specific PTS system enzyme IIC | - | NP_418614 | ulaA | ascorbate-specific PTS system enzyme IIC | nGCO | 27.77 | 52.29 |
| NP_463244 | ulaA | ascorbate-specific PTS system enzyme IIC | - | NP_418614 | ulaA | ascorbate-specific PTS system enzyme IIC | GCO | 95.91 | 52.64 |
| --=-- |  |  |  |  |  |  |  |  |  |
| NP_459085 | - | putative outer membrane lipoprotein | - | NP_417289 | ygdI | hypothetical protein | nGCO | 60.71 | 48.48 |
| NP_461902 | ygdI | putative lipoprotein | - | NP_417289 | ygdI | hypothetical protein | GCO | 93.24 | 45.61 |
| --=-- |  |  |  |  |  |  |  |  |  |
| NP_459626 | ybeF | putative transcriptional regulator | + | NP_415162 | ybeF | predicted DNA-binding transcriptional regulator | GCO | 68.76 | 50.94 |
| NP_462636 | - | putative transcriptional regulator | - | NP_415162 | ybeF | predicted DNA-binding transcriptional regulator | nGCO | 25.28 | 52.71 |
| --=-- |  |  |  |  |  |  |  |  |  |
| NP_460384 | ssaR | needle complex export protein | + | NP_416458 | fliP | flagellar biosynthesis protein P | nGCO | 40.2 | 42.9 |
| NP_460932 | fliP | flagellar biosynthesis protein | - | NP_416458 | fliP | flagellar biosynthesis protein P | GCO | 89.38 | 53.65 |
| NP_461811 | spaP | needle complex export protein | - | NP_416458 | fliP | flagellar biosynthesis protein P | nGCO | 29.71 | 41.48 |
| --=-- |  |  |  |  |  |  |  |  |  |
| NP_459604 | - | putative hydrogenase protein | - | NP_415415 | dmsB | dimethyl sulfoxide reductase, anaerobic, subunit B | nGCO | 41.33 | 53.76 |
| NP_459941 | dmsB | anaerobic dimethyl sulfoxide reductase subunit B | - | NP_415415 | dmsB | dimethyl sulfoxide reductase, anaerobic, subunit B | GCO | 96.58 | 57.11 |
| NP_460457 | - | putative dimethyl sulphoxide reductase | - | NP_415415 | dmsB | dimethyl sulfoxide reductase, anaerobic, subunit B | nGCO | 96.09 | 56.79 |
| NP_461464 | - | putative anaerobic dimethylsulfoxide reductase | - | NP_415415 | dmsB | dimethyl sulfoxide reductase, anaerobic, subunit B | GCO | 56.25 | 53.96 |
| NP_463171 | - | putative anaerobic dimethylsulfoxide reductase subunit B | - | NP_415415 | dmsB | dimethyl sulfoxide reductase, anaerobic, subunit B | GCO | 60.09 | 53.9 |
| --=-- |  |  |  |  |  |  |  |  |  |
| NP_459029 | bcfD | fimbrial subunit | - | NP_415066 | sfmH | predicted fimbrial-like adhesin protein | nGCO | 43.96 | 50.09 |
| NP_459542 | fimH | minor fimbrial subunit | + | NP_415066 | sfmH | predicted fimbrial-like adhesin protein | GCO | 72.2 | 51.68 |
| --=-- |  |  |  |  |  |  |  |  |  |
| NP_461682 | - | putative hexulose 6 phosphate synthase | - | NP_418617 | ulaD | 3-keto-L-gulonate 6-phosphate decarboxylase | nGCO | 32.53 | 53.93 |
| NP_463247 | sgaH | putative hexulose phosphate synthase | - | NP_418617 | ulaD | 3-keto-L-gulonate 6-phosphate decarboxylase | GCO | 98.61 | 57.91 |
| --=-- |  |  |  |  |  |  |  |  |  |
| NP_460351 | ttrS | sensory histidine kinase | - | NP_418536 | basS | sensory histidine kinase in two-component regulatory system with BasR | nGCO | 25.07 | 56.77 |
| NP_463156 | basS | sensor kinase | - | NP_418536 | basS | sensory histidine kinase in two-component regulatory system with BasR | GCO | 85.91 | 54.62 |
| --=-- |  |  |  |  |  |  |  |  |  |
| NP_459179 | stiH | putative fimbrial protein precurosr | - | NP_415461 | ycbT | predicted fimbrial-like adhesin protein | GCO | 27.05 | 52.68 |
| NP_462538 | lpfD | long polar fimbrial protein | - | NP_415461 | ycbT | predicted fimbrial-like adhesin protein | GCO | 26.92 | 46.66 |
| --=-- |  |  |  |  |  |  |  |  |  |
| NP_460465 | rspA | putative dehydratase | - | NP_416098 | rspA | predicted dehydratase | GCO | 94.8 | 54.65 |
| NP_462597 | - | putative mandelate racemase | - | NP_416098 | rspA | predicted dehydratase | nGCO | 24.79 | 54.55 |
| --=-- |  |  |  |  |  |  |  |  |  |
| NP_461677 | - | putative PTS system glucitol/sorbitol-specific enzyme II | - | YP_026180 | srlA | glucitol/sorbitol-specific enzyme IIC component of PTS | nGCO | 40.25 | 59.57 |
| NP_461753 | srlA | glucitol/sorbitol-specific enzyme IIC component | - | YP_026180 | srlA | glucitol/sorbitol-specific enzyme IIC component of PTS | GCO | 86.63 | 50.53 |
| --=-- |  |  |  |  |  |  |  |  |  |
| NP_462500 | - | anaerobic C4-dicarboxylate transporter | - | NP_418561 | dcuA | C4-dicarboxylate antiporter | nGCO | 35.51 | 50.68 |
| NP_463189 | dcuA | anaerobic C4-dicarboxylate transporter | - | NP_418561 | dcuA | C4-dicarboxylate antiporter | GCO | 86.29 | 53.68 |
| --=-- |  |  |  |  |  |  |  |  |  |
| NP_459648 | ybeS | putative molecular chaperone | - | NP_415182 | djlC | Hsc56 co-chaperone of HscC | GCO | 47.29 | 54.88 |
| NP_459650 | ybeV | putative molecular chaperone | - | NP_415182 | djlC | Hsc56 co-chaperone of HscC | GCO | 57.14 | 54.1 |
| --=-- |  |  |  |  |  |  |  |  |  |
| NP_459356 | - | cytochrome BD2 subunit II | - | NP_415262 | cydB | cytochrome d terminal oxidase, subunit II | nGCO | 28.49 | 55.19 |
| NP_459726 | cydB | cytochrome d terminal oxidase polypeptide subunit II | - | NP_415262 | cydB | cytochrome d terminal oxidase, subunit II | GCO | 86.01 | 54.91 |
| --=-- |  |  |  |  |  |  |  |  |  |
| NP_460341 | lppB | putative methyl-accepting chemotaxis protein | - | NP_416192 | lpp | murein lipoprotein | GCO | 88.57 | 47.08 |
| NP_460342 | lpp | murein lipoprotein | - | NP_416192 | lpp | murein lipoprotein | GCO | 96.15 | 51.47 |
| --=-- |  |  |  |  |  |  |  |  |  |
| NP_460557 | ydcR | putative regulatory protein | - | NP_415956 | ydcR | fused predicted DNA-binding transcriptional regulator/predicted amino transferase | nGCO | 86.11 | 53.19 |
| NP_461729 | - | putative regulatory protein | - | NP_415956 | ydcR | fused predicted DNA-binding transcriptional regulator/predicted amino transferase | nGCO | 29.38 | 56.62 |
| --=-- |  |  |  |  |  |  |  |  |  |
| NP_459399 | queA | S-adenosylmethionine:tRNA ribosyltransferase-isomerase | - | NP_414939 | queA | S-adenosylmethionine:tRNA ribosyltransferase-isomerase | GCO | 94.33 | 55.86 |
| NP_460508 | - | putative S-adenosylmethionine/tRNA-ribosyltransferase-isomerase | + | NP_414939 | queA | S-adenosylmethionine:tRNA ribosyltransferase-isomerase | nGCO | 28.52 | 38.51 |
| --=-- |  |  |  |  |  |  |  |  |  |
| NP_460352 | ttrR | response regulator | - | NP_418125 | uhpA | DNA-binding response regulator in two-component regulatory system wtih UhpB | nGCO | 32.65 | 55.08 |
| NP_462689 | uhpA | response regulator | - | NP_418125 | uhpA | DNA-binding response regulator in two-component regulatory system wtih UhpB | GCO | 97.44 | 58.71 |
| --=-- |  |  |  |  |  |  |  |  |  |
| NP_461149 | fruA | fructose-specific transport protein | - | NP_416672 | fruA | fused fructose-specific PTS enzymes: IIBcomponent/IIC components | GCO | 86.14 | 59.2 |
| NP_462168 | - | putative phosphotransferase system fructose-specific component IIB | - | NP_416672 | fruA | fused fructose-specific PTS enzymes: IIBcomponent/IIC components | nGCO | 37.97 | 47.33 |
| NP_462757 | - | putative phosphotransferase system fructose-specific component IIB | - | NP_416672 | fruA | fused fructose-specific PTS enzymes: IIBcomponent/IIC components | nGCO | 63.69 | 56.04 |
| --=-- |  |  |  |  |  |  |  |  |  |
| NP_460434 | ompN | outer membrane protein N precursor | + | NP_415895 | ompN | outer membrane pore protein N, non-specific | nGCO | 76.96 | 47.61 |
| NP_460490 | - | putative outer membrane protein | - | NP_415895 | ompN | outer membrane pore protein N, non-specific | nGCO | 62.15 | 46.38 |
| NP_460946 | ompS | putative porin | - | NP_415895 | ompN | outer membrane pore protein N, non-specific | nGCO | 66.84 | 50.37 |
| --=-- |  |  |  |  |  |  |  |  |  |
| NP_461193 | ccmE | periplasmic heme-dependent peroxidase | - | NP_416701 | ccmE | periplasmic heme chaperone | GCO | 74.21 | 65.41 |
| NP_462715 | ccmE | periplasmic heme-dependent peroxidase | - | NP_416701 | ccmE | periplasmic heme chaperone | GCO | 74.21 | 65.41 |
| --=-- |  |  |  |  |  |  |  |  |  |
| NP_459744 | - | transcriptional regulator | + | NP_418747 | yjiE | predicted DNA-binding transcriptional regulator | nGCO | 29.6 | 39.53 |
| NP_463370 | yjiE | putative transcriptional regulator | - | NP_418747 | yjiE | predicted DNA-binding transcriptional regulator | GCO | 81.78 | 52.36 |
| --=-- |  |  |  |  |  |  |  |  |  |
| NP_459298 | safB | putative fimbrial assembly chaparone | + | NP_417612 | yraI | predicted periplasmic pilin chaperone | nGCO | 35.64 | 48.87 |
| NP_462540 | lpfB | long polar fimbrial chaperone precursor | - | NP_417612 | yraI | predicted periplasmic pilin chaperone | nGCO | 41.28 | 44.77 |
| --=-- |  |  |  |  |  |  |  |  |  |
| NP_459349 | - | putative transcriptional regulator | - | NP_415020 | cueR | DNA-binding transcriptional activator of copper-responsive regulon genes | nGCO | 40.47 | 54.4 |
| NP_459494 | cueR | putative heavy metal transcriptional repressor | - | NP_415020 | cueR | DNA-binding transcriptional activator of copper-responsive regulon genes | GCO | 91.79 | 52.27 |
| NP_463185 | - | putative regulatory protein | - | NP_415020 | cueR | DNA-binding transcriptional activator of copper-responsive regulon genes | nGCO | 26.36 | 47.74 |
| --=-- |  |  |  |  |  |  |  |  |  |
| NP_460499 | - | putative hydrogenase-1 small subunit | + | NP_415491 | hyaA | hydrogenase 1, small subunit | GCO | 72.92 | 51.9 |
| NP_460742 | - | hydrogenase-1 small subunit | - | NP_415491 | hyaA | hydrogenase 1, small subunit | GCO | 84.67 | 56.92 |
| --=-- |  |  |  |  |  |  |  |  |  |
| NP_459567 | - | putative inner membrane protein | - | NP_416332 | manY | mannose-specific enzyme IIC component of PTS | GCO | 34.71 | 57.25 |
| NP_460787 | manY | mannose-specific enzyme IIC | - | NP_416332 | manY | mannose-specific enzyme IIC component of PTS | GCO | 81.95 | 56.3 |
| NP_463396 | - | putative PTS permease | - | NP_416332 | manY | mannose-specific enzyme IIC component of PTS | GCO | 25.31 | 50.51 |
| --=-- |  |  |  |  |  |  |  |  |  |
| NP_461678 | - | putative glucitol-specific PTS enzyme III | - | NP_417184 | srlB | glucitol/sorbitol-specific enzyme IIA component of PTS | GCO | 41.12 | 59.73 |
| NP_461755 | slrB | glucitol/sorbitol-specific enzyme IIA component | - | NP_417184 | srlB | glucitol/sorbitol-specific enzyme IIA component of PTS | GCO | 82.2 | 57.57 |
| --=-- |  |  |  |  |  |  |  |  |  |
| NP_460672 | cysB | transcriptional regulator for cysteine regulon | - | NP_415791 | cysB | DNA-binding transcriptional dual regulator, O-acetyl-L-serine-binding | GCO | 95.06 | 51.89 |
| NP_461223 | - | putative transcriptional regulator | - | NP_415791 | cysB | DNA-binding transcriptional dual regulator, O-acetyl-L-serine-binding | nGCO | 26 | 48 |
| --=-- |  |  |  |  |  |  |  |  |  |
| NP_461127 | yohK | hypothetical protein | - | NP_416647 | yohK | predicted inner membrane protein | GCO | 80 | 56.03 |
| NP_463137 | - | putative inner membrane protein | - | NP_416647 | yohK | predicted inner membrane protein | GCO | 27.97 | 58.84 |
| --=-- |  |  |  |  |  |  |  |  |  |
| NP_462431 | gldA | glycerol dehydrogenase | - | NP_418380 | gldA | glycerol dehydrogenase | nGCO | 45.58 | 54.86 |
| NP_462989 | gldA | glycerol dehydrogenase | - | NP_418380 | gldA | glycerol dehydrogenase | GCO | 91.55 | 58.15 |
| --=-- |  |  |  |  |  |  |  |  |  |
| NP_459067 | citX2 | putative cytoplasmic protein | - | NP_415147 | citX | 2'-(5'-triphosphoribosyl)-3'-dephospho-CoA:apo-citrate lyase | GCO | 40.88 | 55.97 |
| NP_459612 | citX | 2'-(5'-triphosphoribosyl)-3'-dephospho-CoA:apo-citrate lyase | - | NP_415147 | citX | 2'-(5'-triphosphoribosyl)-3'-dephospho-CoA:apo-citrate lyase | GCO | 75.41 | 61.95 |
| --=-- |  |  |  |  |  |  |  |  |  |
| NP_459578 | fes | enterochelin esterase | - | NP_415117 | fes | enterobactin/ferric enterobactin esterase | GCO | 73.92 | 60.08 |
| NP_461702 | iroD | enterochelin esterase=-like protein | - | NP_415117 | fes | enterobactin/ferric enterobactin esterase | nGCO | 29.3 | 62.08 |
| --=-- |  |  |  |  |  |  |  |  |  |
| NP_460079 | hpaI | 4-hydroxyphenylacetate catabolism | - | NP_416748 | yfaU | predicted 2,4-dihydroxyhept-2-ene-1,7-dioic acid aldolase | GCO | 58.89 | 60.73 |
| NP_461231 | - | putative 2,4-dihydroxyhept-2-ene-1,7-dioic acid aldolase | - | NP_416748 | yfaU | predicted 2,4-dihydroxyhept-2-ene-1,7-dioic acid aldolase | GCO | 81.27 | 54.6 |
| --=-- |  |  |  |  |  |  |  |  |  |
| NP_459064 | citD2 | putative citrate lyase acyl carrier protein gamma chain | - | NP_415150 | citD | citrate lyase, acyl carrier (gamma) subunit | GCO | 51.35 | 57.14 |
| NP_459615 | citD | citrate lyase acyl carrier protein gamma chain | - | NP_415150 | citD | citrate lyase, acyl carrier (gamma) subunit | GCO | 79.59 | 56.9 |
| --=-- |  |  |  |  |  |  |  |  |  |
| NP_460003 | - | lysozyme | - | NP_415087 | ybcS | DLP12 prophage; predicted lysozyme | GCO | 36.75 | 53.2 |
| NP_461548 | - | morphogenesis-like protein | - | NP_415087 | ybcS | DLP12 prophage; predicted lysozyme | GCO | 35.04 | 52.75 |
| NP_461643 | - | probable prophage lysozyme | - | NP_415087 | ybcS | DLP12 prophage; predicted lysozyme | nGCO | 34.19 | 60.37 |
| --=-- |  |  |  |  |  |  |  |  |  |
| NP_460473 | - | putative cytoplasmic protein | + | NP_415775 | yciG | hypothetical protein | nGCO | 89.83 | 49.72 |
| NP_460687 | yciG | putative cytoplasmic protein | + | NP_415775 | yciG | hypothetical protein | GCO | 88.33 | 50.81 |
| --=-- |  |  |  |  |  |  |  |  |  |
| NP_460593 | - | putative ABC transporter permease component | + | NP_416428 | yecS | predicted transporter subunit: membrane component of ABC superfamily | nGCO | 31.34 | 41.97 |
| NP_460595 | - | putative ABC-type transport system membrane component | + | NP_416428 | yecS | predicted transporter subunit: membrane component of ABC superfamily | nGCO | 32.85 | 43.46 |
| NP_460905 | yecS | putative ABC-type amino acid transporter permease component | - | NP_416428 | yecS | predicted transporter subunit: membrane component of ABC superfamily | GCO | 86.03 | 54.55 |
| --=-- |  |  |  |  |  |  |  |  |  |
| NP_459515 | - | putative permease | - | NP_418776 | yjiZ | predicted transporter | nGCO | 20.68 | 46.07 |
| NP_460503 | - | putative transport protein | - | NP_418776 | yjiZ | predicted transporter | nGCO | 27.1 | 46.62 |
| --=-- |  |  |  |  |  |  |  |  |  |
| NP_459877 | - | putative helicase | - | NP_416689 | yejH | predicted ATP-dependet helicase | nGCO | 28.03 | 52.9 |
| NP_461167 | yejH | putative ATP-dependent helicase | - | NP_416689 | yejH | predicted ATP-dependet helicase | GCO | 95.39 | 57.01 |
| --=-- |  |  |  |  |  |  |  |  |  |
| NP_459885 | - | hypothetical protein | - | NP_415088 | rzpD | DLP12 prophage; predicted murein endopeptidase | nGCO | 66.43 | 53.9 |
| NP_460004 | - | hypothetical protein | - | NP_415088 | rzpD | DLP12 prophage; predicted murein endopeptidase | GCO | 65.3 | 54.37 |
| NP_461547 | - | endopeptidase-like protein | - | NP_415088 | rzpD | DLP12 prophage; predicted murein endopeptidase | GCO | 43.79 | 51.89 |
| --=-- |  |  |  |  |  |  |  |  |  |
| NP_459642 | - | putative hydrolas | - | NP_417597 | garD | (D)-galactarate dehydrogenase | nGCO | 33.91 | 52.6 |
| NP_462163 | garD | galactarate dehydrogenase | + | NP_417597 | garD | (D)-galactarate dehydrogenase | nGCO | 91.39 | 56.8 |
| --=-- |  |  |  |  |  |  |  |  |  |
| NP_459019 | - | putative transcriptional regulator | + | NP_415136 | ybdO | predicted DNA-binding transcriptional regulator | nGCO | 24.52 | 40.61 |
| NP_459035 | - | putative transcriptional regulator | - | NP_415136 | ybdO | predicted DNA-binding transcriptional regulator | nGCO | 20.77 | 38.8 |
| NP_459598 | ybdO | putative transcriptional regulator | + | NP_415136 | ybdO | predicted DNA-binding transcriptional regulator | GCO | 54.36 | 38.98 |
| --=-- |  |  |  |  |  |  |  |  |  |
| NP_460218 | - | putative molecular chaperone | - | NP_418141 | ibpB | heat shock chaperone | nGCO | 31.45 | 45.51 |
| NP_462708 | ibpB | small heat shock protein | - | NP_418141 | ibpB | heat shock chaperone | GCO | 88.73 | 51.26 |
| --=-- |  |  |  |  |  |  |  |  |  |
| NP_462173 | - | PTS family galactitol-specific enzyme IIC | - | NP_416595 | gatC | galactitol-specific enzyme IIC component of PTS | GCO | 78.92 | 55.38 |
| NP_462681 | - | putative PTS system galactitol-specific enzyme IIC component | + | NP_416595 | gatC | galactitol-specific enzyme IIC component of PTS | GCO | 39.75 | 46.79 |
| --=-- |  |  |  |  |  |  |  |  |  |
| NP_459116 | leuC | isopropylmalate isomerase large subunit | - | NP_414614 | leuC | isopropylmalate isomerase large subunit | GCO | 93.13 | 58.95 |
| NP_459324 | - | isopropylmalate isomerase large subunit | + | NP_414614 | leuC | isopropylmalate isomerase large subunit | GCO | 50.64 | 51.4 |
| --=-- |  |  |  |  |  |  |  |  |  |
| NP_461619 | - | putative outer membrane efflux protein | - | NP_417507 | tolC | outer membrane channel precursor protein | nGCO | 20.69 | 56.87 |
| NP_462101 | tolC | outer membrane channel precursor protein | - | NP_417507 | tolC | outer membrane channel precursor protein | GCO | 84.56 | 52.03 |
| NP_463124 | - | putative ABC exporter outer membrane component | - | NP_417507 | tolC | outer membrane channel precursor protein | nGCO | 17.2 | 36.74 |
| --=-- |  |  |  |  |  |  |  |  |  |
| NP_459115 | leuD | isopropylmalate isomerase small subunit | - | NP_414613 | leuD | isopropylmalate isomerase small subunit | GCO | 92 | 52.97 |
| NP_459325 | - | putative 3-isopropylmalate isomerase | - | NP_414613 | leuD | isopropylmalate isomerase small subunit | GCO | 38.21 | 52.31 |
| --=-- |  |  |  |  |  |  |  |  |  |
| NP_459833 | - | putative electron transfer protein alpha subunit | + | NP_416213 | ydiR | predicted electron transfer flavoprotein, FAD-binding | nGCO | 29.43 | 41.45 |
| NP_460319 | ydiR | putative electron transfer flavoprotein subunit alpha | - | NP_416213 | ydiR | predicted electron transfer flavoprotein, FAD-binding | GCO | 72.75 | 53.84 |
| --=-- |  |  |  |  |  |  |  |  |  |
| NP_460634 | - | putative aldo/keto reductase | + | NP_417485 | dkgA | 2,5-diketo-D-gluconate reductase A | nGCO | 40.07 | 46.66 |
| NP_462080 | yqhE | 2,5-diketo-D-gluconate reductase A | - | NP_417485 | dkgA | 2,5-diketo-D-gluconate reductase A | GCO | 88.36 | 52.89 |
| --=-- |  |  |  |  |  |  |  |  |  |
| NP_459904 | - | putative phage tail assembly protein | - | NP_415891 | tfaR | Rac prophage; predicted tail fiber assembly protein | nGCO | 61.13 | 47.22 |
| NP_460025 | - | tail fiber assembly like-protein | - | NP_415891 | tfaR | Rac prophage; predicted tail fiber assembly protein | nGCO | 62.69 | 48.62 |
| NP_460823 | mig-3 | phage-tail assembly-like protein | - | NP_415891 | tfaR | Rac prophage; predicted tail fiber assembly protein | nGCO | 50.88 | 50.68 |
| NP_461522 | - | phage tail assembly-like protein | - | NP_415891 | tfaR | Rac prophage; predicted tail fiber assembly protein | nGCO | 59.16 | 47.84 |
| --=-- |  |  |  |  |  |  |  |  |  |
| NP_459028 | bcfC | fimbrial usher | - | NP_415460 | ycbS | predicted outer membrane usher protein | nGCO | 52.01 | 54.46 |
| NP_459180 | stiC | putativie fimbrial usher | - | NP_415460 | ycbS | predicted outer membrane usher protein | GCO | 38.67 | 55.35 |
| NP_462539 | lpfC | long polar fimbrial outer membrane usher protein | - | NP_415460 | ycbS | predicted outer membrane usher protein | GCO | 44.28 | 55.35 |
| --=-- |  |  |  |  |  |  |  |  |  |
| NP_460997 | pduQ | propanol dehydrogenase | - | NP_416948 | eutG | predicted alcohol dehydrogenase in ethanolamine utilization | nGCO | 37.36 | 60.37 |
| NP_461396 | eutG | putative transport protein | - | NP_416948 | eutG | predicted alcohol dehydrogenase in ethanolamine utilization | GCO | 81.47 | 62.87 |
| --=-- |  |  |  |  |  |  |  |  |  |
| NP_459306 | yafV | putative amidohydrolase | - | NP_414754 | yafV | predicted C-N hydrolase family amidase, NAD(P)-binding | nGCO | 82.35 | 54.16 |
| NP_459623 | ybeM | putative hydrolase | - | NP_414754 | yafV | predicted C-N hydrolase family amidase, NAD(P)-binding | nGCO | 26.87 | 56.4 |
| --=-- |  |  |  |  |  |  |  |  |  |
| NP_459509 | ybbS | putative transcriptional regulator | - | NP_415037 | ybbS | DNA-binding transcriptional activator of the allD operon | GCO | 86.64 | 53.5 |
| NP_461937 | - | putative transcriptional regulator | - | NP_415037 | ybbS | DNA-binding transcriptional activator of the allD operon | nGCO | 25.62 | 54.51 |
| --=-- |  |  |  |  |  |  |  |  |  |
| NP_460592 | - | putative periplasmic binding protein | + | NP_416430 | fliY | cystine transporter subunit | nGCO | 23.66 | 39.63 |
| NP_460907 | fliY | putative periplasmic binding transport protein | - | NP_416430 | fliY | cystine transporter subunit | GCO | 86.84 | 51.68 |
| --=-- |  |  |  |  |  |  |  |  |  |
| NP_460173 | ptsG | glucose-specific IIBC component | - | NP_415619 | ptsG | fused glucose-specific PTS enzymes: IIB component/IIC component | GCO | 93.06 | 54.74 |
| NP_461685 | - | putative phosphotransferase system IIC component | - | NP_415619 | ptsG | fused glucose-specific PTS enzymes: IIB component/IIC component | nGCO | 42.56 | 57.52 |
| --=-- |  |  |  |  |  |  |  |  |  |
| NP_460564 | ydcN | putative repressor | - | NP_415951 | ydcN | predicted DNA-binding transcriptional regulator | GCO | 72.88 | 52.88 |
| NP_460623 | - | putative transcriptional regulator | - | NP_415951 | ydcN | predicted DNA-binding transcriptional regulator | nGCO | 28.65 | 51.74 |
| NP_462923 | - | hypothetical protein | - | NP_415951 | ydcN | predicted DNA-binding transcriptional regulator | nGCO | 24.37 | 58.73 |
| --=-- |  |  |  |  |  |  |  |  |  |
| NP_460101 | - | putative inner membrane protein | - | NP_417690 | nanE | predicted N-acetylmannosamine-6-P epimerase | nGCO | 65.48 | 51.54 |
| NP_462247 | nanE | putative ManNAc-6P epimerase | - | NP_417690 | nanE | predicted N-acetylmannosamine-6-P epimerase | GCO | 56.82 | 58.98 |
| --=-- |  |  |  |  |  |  |  |  |  |
| NP_460082 | - | putative periplasmic protein | - | NP_416771 | elaC | ribonuclease Z | nGCO | 23.79 | 52.6 |
| NP_461255 | elaC | ribonuclease Z | - | NP_416771 | elaC | ribonuclease Z | GCO | 80.98 | 55.55 |
| --=-- |  |  |  |  |  |  |  |  |  |
| NP_460662 | yciR | putative diguanylate cyclase/phosphodiesterase | - | NP_415801 | gmr | modulator of Rnase II stability | GCO | 76.71 | 50.52 |
| NP_462298 | - | putative signal transduction protein | - | NP_415801 | gmr | modulator of Rnase II stability | nGCO | 36.21 | 54.19 |
| --=-- |  |  |  |  |  |  |  |  |  |
| NP_461001 | pduV | propanediol utilization protein | - | NP_416956 | eutP | conserved protein with nucleoside triphosphate hydrolase domain | GCO | 28.27 | 57.61 |
| NP_461404 | eutP | putative ethanolamine utilization protein | - | NP_416956 | eutP | conserved protein with nucleoside triphosphate hydrolase domain | GCO | 84.9 | 51.45 |
| --=-- |  |  |  |  |  |  |  |  |  |
| NP_459565 | - | putative inner membrane protein | - | NP_418185 | glmS | D-fructose-6-phosphate amidotransferase | nGCO | 20.7 | 58.14 |
| NP_462760 | glmS | D-fructose-6-phosphate amidotransferase | - | NP_418185 | glmS | D-fructose-6-phosphate amidotransferase | GCO | 96.88 | 54.48 |
| NP_463398 | - | putative glucosamine-fructose-6-phosphate aminotransferase | - | NP_418185 | glmS | D-fructose-6-phosphate amidotransferase | nGCO | 23.31 | 52.91 |
| --=-- |  |  |  |  |  |  |  |  |  |
| NP_461222 | - | putative permease | - | NP_417930 | yhhS | predicted transporter | nGCO | 24.11 | 49.45 |
| NP_462482 | yhhS | hypothetical protein | - | NP_417930 | yhhS | predicted transporter | GCO | 78.37 | 58.86 |
| --=-- |  |  |  |  |  |  |  |  |  |
| NP_459341 | - | putative outer membrane protein | + | NP_415335 | ompX | outer membrane protein X | nGCO | 39.49 | 41.52 |
| NP_459810 | ompX | outer membrane protein X | - | NP_415335 | ompX | outer membrane protein X | GCO | 77.77 | 51.74 |
| NP_459897 | - | Ail/OmpX-like protein | - | NP_415335 | ompX | outer membrane protein X | nGCO | 34.91 | 45.66 |
| NP_460018 | - | attachment/invasion protein | - | NP_415335 | ompX | outer membrane protein X | nGCO | 33.33 | 44.37 |
| NP_460215 | pagC | virulence membrane protein PAGC precursor | - | NP_415335 | ompX | outer membrane protein X | nGCO | 38.78 | 43.18 |
| NP_461948 | - | Ail/OmpX-like protein | + | NP_415335 | ompX | outer membrane protein X | nGCO | 29.29 | 48.6 |
| --=-- |  |  |  |  |  |  |  |  |  |
| NP_462022 | ansB | periplasmic L-asparaginase II | - | NP_417432 | ansB | periplasmic L-asparaginase II | GCO | 88.79 | 52.62 |
| NP_462499 | - | putative L-asparaginase | - | NP_417432 | ansB | periplasmic L-asparaginase II | nGCO | 44.07 | 51.34 |
| --=-- |  |  |  |  |  |  |  |  |  |
| NP_460496 | - | putative hydrogenase maturation protease | - | NP_415494 | hyaD | protein involved in processing of HyaA and HyaB proteins | GCO | 53.07 | 53.2 |
| NP_460745 | - | putative hydrogenase maturation protease | - | NP_415494 | hyaD | protein involved in processing of HyaA and HyaB proteins | GCO | 79.16 | 57.95 |
| --=-- |  |  |  |  |  |  |  |  |  |
| NP_462386 | rpe | ribulose-phosphate 3-epimerase | - | NP_417845 | rpe | ribulose-phosphate 3-epimerase | GCO | 97.32 | 53.68 |
| NP_462961 | - | ribulose-phosphate 3-epimerase | - | NP_417845 | rpe | ribulose-phosphate 3-epimerase | nGCO | 25.47 | 51.63 |
| --=-- |  |  |  |  |  |  |  |  |  |
| NP_461316 | - | putative regulatory protein | - | NP_417655 | sfsB | DNA-binding transcriptional activator of maltose metabolism | nGCO | 50.68 | 46.98 |
| NP_461676 | - | putative cytoplasmic protein | - | NP_417655 | sfsB | DNA-binding transcriptional activator of maltose metabolism | nGCO | 65.07 | 56.84 |
| NP_462216 | nlp | transcriptional regulator | - | NP_417655 | sfsB | DNA-binding transcriptional activator of maltose metabolism | GCO | 86.95 | 49.3 |
| --=-- |  |  |  |  |  |  |  |  |  |
| NP_460501 | - | putative regulatory protein | - | NP_418744 | uxuR | DNA-binding transcriptional repressor | nGCO | 31.83 | 47.09 |
| NP_462000 | - | putative regulatory protein | - | NP_418744 | uxuR | DNA-binding transcriptional repressor | nGCO | 40.35 | 44.93 |
| NP_463366 | uxuR | uxu operon transcriptional repressor | - | NP_418744 | uxuR | DNA-binding transcriptional repressor | nGCO | 89.49 | 54.65 |
| --=-- |  |  |  |  |  |  |  |  |  |
| NP_460453 | - | putative ABC transporter periplasmic component | - | NP_416635 | yehZ | predicted transporter subunit: periplasmic-binding component of ABC superfamily | nGCO | 27.71 | 52.38 |
| NP_461110 | yehZ | putative transport protein | - | NP_416635 | yehZ | predicted transporter subunit: periplasmic-binding component of ABC superfamily | GCO | 90.14 | 54.9 |
| --=-- |  |  |  |  |  |  |  |  |  |
| NP_461507 | yfhH | putative transport protein | - | NP_417056 | yfhH | predicted DNA-binding transcriptional regulator | nGCO | 79.07 | 57.24 |
| NP_463278 | - | putative transcriptional regulator | - | NP_417056 | yfhH | predicted DNA-binding transcriptional regulator | nGCO | 25.09 | 48.2 |
| --=-- |  |  |  |  |  |  |  |  |  |
| NP_460949 | umuD | SOS-response transcriptional repressors | - | NP_415701 | umuD | DNA polymerase V, subunit D | GCO | 73.18 | 50.23 |
| NP_461173 | - | DNA polymerase V subunit | - | NP_415701 | umuD | DNA polymerase V, subunit D | nGCO | 39.34 | 51.59 |
| --=-- |  |  |  |  |  |  |  |  |  |
| NP_459181 | stiB | putative fimbrial chaparone | - | NP_418736 | fimC | chaperone, periplasmic | nGCO | 39.1 | 53.07 |
| NP_459331 | stbE | putative fimbrial chaparone | + | NP_418736 | fimC | chaperone, periplasmic | nGCO | 25.6 | 47.69 |
| NP_463450 | sthA | putative fimbrial chaparone | + | NP_418736 | fimC | chaperone, periplasmic | GCO | 33.99 | 46.19 |
| --=-- |  |  |  |  |  |  |  |  |  |
| NP_461225 | glpT | sn-glycerol-3-phosphate transport protein | - | NP_416743 | glpT | sn-glycerol-3-phosphate transporter | GCO | 86.72 | 54.74 |
| NP_461340 | pgtP | transporter | - | NP_416743 | glpT | sn-glycerol-3-phosphate transporter | nGCO | 35.46 | 49.78 |
| --=-- |  |  |  |  |  |  |  |  |  |
| NP_459474 | - | putative transposase | - | NP_416808 | yfcI | hypothetical protein | nGCO | 49.67 | 53.09 |
| NP_462411 | - | putative cytoplasmic protein | - | NP_416808 | yfcI | hypothetical protein | nGCO | 67.76 | 52.24 |
| NP_462665 | - | putative cytoplasmic protein | - | NP_416808 | yfcI | hypothetical protein | nGCO | 62.93 | 50.21 |
| --=-- |  |  |  |  |  |  |  |  |  |
| NP_459959 | msbA | transport protein | - | NP_415434 | msbA | fused lipid transporter subunits of ABC superfamily: membrane component/ATP-binding component | GCO | 91.75 | 51.68 |
| NP_463127 | - | putative ABC-type bacteriocin/lantibiotic exporter | - | NP_415434 | msbA | fused lipid transporter subunits of ABC superfamily: membrane component/ATP-binding component | nGCO | 20.77 | 35.31 |
| --=-- |  |  |  |  |  |  |  |  |  |
| NP_459310 | yafK | putative periplasmic protein | - | NP_414759 | yafK | hypothetical protein | GCO | 86.99 | 48.58 |
| NP_462190 | - | putative inner membrane protein | - | NP_414759 | yafK | hypothetical protein | nGCO | 51.53 | 46.52 |
| --=-- |  |  |  |  |  |  |  |  |  |
| NP_462086 | ygiK | putative transporter | - | YP_026232 | yiaN | predicted transporter | GCO | 27.38 | 51.14 |
| NP_462572 | yiaN | hypothetical protein | - | YP_026232 | yiaN | predicted transporter | GCO | 66.11 | 52.58 |
| NP_462933 | - | putative C4-dicarboxylate transport system | - | YP_026232 | yiaN | predicted transporter | GCO | 27.76 | 52.37 |
| --=-- |  |  |  |  |  |  |  |  |  |
| NP_459927 | - | putative cytoplasmic protein | - | NP_417896 | yhhW | hypothetical protein | nGCO | 28.64 | 56.21 |
| NP_462445 | yhhW | putative cytoplasmic protein | - | NP_417896 | yhhW | hypothetical protein | GCO | 93.93 | 55.45 |
| --=-- |  |  |  |  |  |  |  |  |  |
| NP_459347 | - | putative cation efflux pump | - | NP_414996 | acrA | multidrug efflux system | nGCO | 29.64 | 60.77 |
| NP_459471 | acrA | acridine efflux pump | - | NP_414996 | acrA | multidrug efflux system | GCO | 80.35 | 55.19 |
| --=-- |  |  |  |  |  |  |  |  |  |
| NP_461291 | yfcG | putative glutathione S-transferase | - | NP_416805 | yfcG | predicted glutathione S-transferase | GCO | 83.17 | 53.24 |
| NP_463132 | - | putative glutathione S-transferase | - | NP_416805 | yfcG | predicted glutathione S-transferase | nGCO | 23.87 | 54.7 |
| --=-- |  |  |  |  |  |  |  |  |  |
| NP_460497 | - | putative Ni/Fe hydrogenase 1 b-type cytochrome subunit | - | NP_415493 | hyaC | hydrogenase 1, b-type cytochrome subunit | GCO | 54.42 | 51.07 |
| NP_460744 | - | putative Ni/Fe hydrogenase 1 b-type cytochrome subunit | - | NP_415493 | hyaC | hydrogenase 1, b-type cytochrome subunit | GCO | 83.04 | 55.32 |
| --=-- |  |  |  |  |  |  |  |  |  |
| NP_462662 | mgtB | Mg2+ transporter | - | NP_418663 | mgtA | magnesium transporter | nGCO | 52.34 | 52.36 |
| NP_463316 | mgtA | Mg2+ ATPase transporter | - | NP_418663 | mgtA | magnesium transporter | GCO | 86.83 | 55.07 |
| --=-- |  |  |  |  |  |  |  |  |  |
| NP_460166 | acpP | acyl carrier protein | - | NP_415612 | acpP | acyl carrier protein | GCO | 82.05 | 47.67 |
| NP_461802 | iacP | acyl carrier protein | - | NP_415612 | acpP | acyl carrier protein | nGCO | 38.7 | 37.75 |
| --=-- |  |  |  |  |  |  |  |  |  |
| NP_461191 | ccmG | heme lyase/disulfide oxidoreductase | - | NP_416699 | ccmG | periplasmic thioredoxin of cytochrome c-type biogenesis | GCO | 79.37 | 62.18 |
| NP_462713 | ccmG | heme lyase disulfide oxidoreductase | - | NP_416699 | ccmG | periplasmic thioredoxin of cytochrome c-type biogenesis | GCO | 79.37 | 62.18 |
| --=-- |  |  |  |  |  |  |  |  |  |
| NP_461194 | ccmD | heme exporter protein C | - | NP_416702 | ccmD | cytochrome c biogenesis protein | GCO | 74 | 69.48 |
| NP_462716 | ccmD | heme exporter protein C | - | NP_416702 | ccmD | cytochrome c biogenesis protein | GCO | 74 | 69.48 |
| --=-- |  |  |  |  |  |  |  |  |  |
| NP_460484 | yneI | putative succinate-semialdehyde dehydrogenase | - | NP_416042 | yneI | predicted aldehyde dehydrogenase | GCO | 75.75 | 56.94 |
| NP_463378 | - | putative NAD-dependent aldehyde dehydrogenase | - | NP_416042 | yneI | predicted aldehyde dehydrogenase | nGCO | 41.07 | 57.03 |
| --=-- |  |  |  |  |  |  |  |  |  |
| NP_460447 | ynfL | putative transcriptional regulator | - | NP_416112 | ynfL | predicted DNA-binding transcriptional regulator | GCO | 78.45 | 57.11 |
| NP_462036 | - | putative transcriptional regulator | - | NP_416112 | ynfL | predicted DNA-binding transcriptional regulator | nGCO | 38.67 | 52.44 |
| --=-- |  |  |  |  |  |  |  |  |  |
| NP_460848 | znuA | high-affinity Zn transport protein | - | NP_416371 | znuA | high-affinity zinc transporter periplasmic component | GCO | 83.43 | 49.9 |
| NP_461782 | sitA | putative periplasmic binding protein | - | NP_416371 | znuA | high-affinity zinc transporter periplasmic component | nGCO | 20.31 | 53.7 |
| --=-- |  |  |  |  |  |  |  |  |  |
| NP_459096 | pdxA | 4-hydroxythreonine-4-phosphate dehydrogenase | - | NP_414594 | pdxA | 4-hydroxythreonine-4-phosphate dehydrogenase | GCO | 89.66 | 58.68 |
| NP_459168 | - | 4-hydroxythreonine-4-phosphate dehydrogenase | - | NP_414594 | pdxA | 4-hydroxythreonine-4-phosphate dehydrogenase | nGCO | 37.08 | 55.69 |
| --=-- |  |  |  |  |  |  |  |  |  |
| NP_460510 | - | putative cytoplasmic protein | - | NP_416081 | relE | Qin prophage; toxin of the RelE-RelB toxin-antitoxin system | nGCO | 62.06 | 42.1 |
| NP_463311 | - | putative inner membrane protein | - | NP_416081 | relE | Qin prophage; toxin of the RelE-RelB toxin-antitoxin system | GCO | 56.38 | 47.01 |
| --=-- |  |  |  |  |  |  |  |  |  |
| NP_461126 | yohJ | hypothetical protein | - | NP_416646 | yohJ | hypothetical protein | GCO | 82.57 | 46.36 |
| NP_463136 | - | putative inner membrane protein | - | NP_416646 | yohJ | hypothetical protein | GCO | 28.12 | 54.74 |
| --=-- |  |  |  |  |  |  |  |  |  |
| NP_460347 | orf408 | putative regulatory protein | + | NP_418208 | rbsK | ribokinase | nGCO | 27.83 | 45.8 |
| NP_462448 | - | putative transcriptional regulator | + | NP_418208 | rbsK | ribokinase | nGCO | 35.88 | 42.4 |
| NP_462692 | - | putative sugar kinase | - | NP_418208 | rbsK | ribokinase | nGCO | 34.33 | 48.96 |
| NP_462784 | rbsK | ribokinase | - | NP_418208 | rbsK | ribokinase | GCO | 85.06 | 55.16 |
| --=-- |  |  |  |  |  |  |  |  |  |
| NP_461679 | - | putative glucitol-specific PTS enzyme III | - | YP_026181 | srlE | glucitol/sorbitol-specific enzyme IIB component of PTS | GCO | 44.89 | 62.79 |
| NP_461754 | srlE | glucitol/sorbitol-specific enzyme IIB component | - | YP_026181 | srlE | glucitol/sorbitol-specific enzyme IIB component of PTS | GCO | 83.28 | 58.64 |
| --=-- |  |  |  |  |  |  |  |  |  |
| NP_460237 | yeaR | putative cytoplasmic protein | - | NP_416311 | yeaR | hypothetical protein | GCO | 83.19 | 49.86 |
| NP_460764 | - | putative cytoplasmic protein | - | NP_416311 | yeaR | hypothetical protein | nGCO | 42.85 | 51.75 |
| --=-- |  |  |  |  |  |  |  |  |  |
| NP_461983 | yggB | putative mechanosensitive channel | - | NP_417399 | mscS | mechanosensitive channel | GCO | 77.54 | 50.87 |
| NP_462983 | - | putative inner membrane protein | - | NP_417399 | mscS | mechanosensitive channel | nGCO | 46.26 | 46.96 |
| --=-- |  |  |  |  |  |  |  |  |  |
| NP_459880 | - | putative chaparone | - | NP_416077 | ydfT | Qin prophage; predicted antitermination protein Q | nGCO | 33.33 | 54.65 |
| NP_459997 | - | putative molecular chaperone | - | NP_416077 | ydfT | Qin prophage; predicted antitermination protein Q | nGCO | 34.31 | 46.36 |
| --=-- |  |  |  |  |  |  |  |  |  |
| NP_460356 | ssrB | transcriptional activator | + | NP_416424 | uvrY | response regulator | nGCO | 30.54 | 39.59 |
| NP_460900 | uvrY | response regulator | - | NP_416424 | uvrY | response regulator | GCO | 96.78 | 51.44 |
| --=-- |  |  |  |  |  |  |  |  |  |
| NP_459577 | fepA | outer membrane ferric enterobactin receptor precursor | - | NP_415116 | fepA | iron-enterobactin outer membrane transporter | GCO | 78.54 | 53.98 |
| NP_461704 | iroN | TonB-dependent siderophore receptor protein | - | NP_415116 | fepA | iron-enterobactin outer membrane transporter | nGCO | 51.72 | 50.93 |
| --=-- |  |  |  |  |  |  |  |  |  |
| NP_460158 | - | putative inner membrane lipoprotein | - | NP_418010 | yiaF | hypothetical protein | nGCO | 28.12 | 46.23 |
| NP_462548 | yiaF | putative outer membrane lipoprotein | - | NP_418010 | yiaF | hypothetical protein | GCO | 82.62 | 55.13 |
| --=-- |  |  |  |  |  |  |  |  |  |
| NP_459428 | thiJ | 4-methyl-5(beta-hydroxyethyl)-thiazole synthesis | - | NP_414958 | yajL | hypothetical protein | GCO | 91.32 | 56.85 |
| NP_460887 | araH | putative intracellular protease/amidase | - | NP_414958 | yajL | hypothetical protein | nGCO | 30.55 | 54.65 |
| --=-- |  |  |  |  |  |  |  |  |  |
| NP_459066 | citF2 | putative citrate lyase alpha chain/citrate-ACP transferase | - | NP_415148 | citF | citrate lyase, citrate-ACP transferase (alpha) subunit | GCO | 72.29 | 55.22 |
| NP_459613 | citF | citrate lyase alpha chain/citrate-ACP transferase | - | NP_415148 | citF | citrate lyase, citrate-ACP transferase (alpha) subunit | GCO | 88.62 | 58.36 |
| --=-- |  |  |  |  |  |  |  |  |  |
| NP_459836 | - | putative transcriptional regulator | + | NP_415289 | ybhD | predicted DNA-binding transcriptional regulator | nGCO | 23.86 | 40.17 |
| NP_462734 | - | putative transcriptional regulator | - | NP_415289 | ybhD | predicted DNA-binding transcriptional regulator | nGCO | 26.75 | 53.4 |
| --=-- |  |  |  |  |  |  |  |  |  |
| NP_459523 | allD | ureidoglycolate dehydrogenase | + | NP_415050 | allD | ureidoglycolate dehydrogenase | GCO | 86.81 | 50 |
| NP_461997 | - | putative malate/L-lactate dehydrogenase | - | NP_415050 | allD | ureidoglycolate dehydrogenase | nGCO | 40.6 | 51.09 |
| --=-- |  |  |  |  |  |  |  |  |  |
| NP_460983 | pduA | polyhedral body protein | - | NP_416952 | cchA | predicted carboxysome structural protein, ethanolamine utilization protein | nGCO | 47.19 | 53.68 |
| NP_460990 | pduJ | polyhedral body protein | - | NP_416952 | cchA | predicted carboxysome structural protein, ethanolamine utilization protein | nGCO | 44.7 | 56.15 |
| NP_461400 | eutM | putative detox protein | - | NP_416952 | cchA | predicted carboxysome structural protein, ethanolamine utilization protein | GCO | 82.1 | 60.48 |
| --=-- |  |  |  |  |  |  |  |  |  |
| NP_459901 | - | putative CuZn superoxide dismutase | - | NP_416163 | sodC | superoxide dismutase, Cu, Zn | nGCO | 61.74 | 48.57 |
| NP_460019 | sodC | superoxide dismutase precursor | - | NP_416163 | sodC | superoxide dismutase, Cu, Zn | nGCO | 55.88 | 48.68 |
| NP_460403 | sodC | copper/zinc superoxide dismutase | - | NP_416163 | sodC | superoxide dismutase, Cu, Zn | GCO | 82.08 | 56.32 |
| --=-- |  |  |  |  |  |  |  |  |  |
| NP_459582 | fepC | enterobactin transporter | - | NP_415120 | fepC | iron-enterobactin transporter subunit | GCO | 88.21 | 56.85 |
| NP_459750 | - | putative ABC-type cobalamin/Fe3+-siderophore transport component | - | NP_415120 | fepC | iron-enterobactin transporter subunit | nGCO | 31.3 | 56.37 |
| --=-- |  |  |  |  |  |  |  |  |  |
| NP_459346 | - | putative cation efflux system protein | - | NP_417732 | acrF | multidrug efflux system protein | nGCO | 38.04 | 56.69 |
| NP_462301 | acrF | multidrug transport protein | - | NP_417732 | acrF | multidrug efflux system protein | GCO | 83.88 | 50.86 |
| --=-- |  |  |  |  |  |  |  |  |  |
| NP_461281 | yfcC | putative integral membrane protein | - | NP_416801 | yfcC | predicted inner membrane protein | GCO | 89.92 | 55.02 |
| NP_463324 | - | putative arginine repressor | - | NP_416801 | yfcC | predicted inner membrane protein | nGCO | 29.37 | 56.05 |
| --=-- |  |  |  |  |  |  |  |  |  |
| NP_459254 | yafC | putative transcriptional regulator | - | NP_414744 | yafC | predicted DNA-binding transcriptional regulator | GCO | 90.42 | 51.69 |
| NP_459928 | - | putative transcriptional regulator | - | NP_414744 | yafC | predicted DNA-binding transcriptional regulator | nGCO | 28.47 | 57.89 |
| NP_461833 | - | putative transcriptional regulator | - | NP_414744 | yafC | predicted DNA-binding transcriptional regulator | nGCO | 26.73 | 55.09 |
| --=-- |  |  |  |  |  |  |  |  |  |
| NP_463279 | - | sugar transporter | - | NP_418455 | xylE | D-xylose transporter | nGCO | 40.86 | 46.79 |
| NP_463280 | - | sugar transporter | - | NP_418455 | xylE | D-xylose transporter | nGCO | 31.37 | 49.68 |
| --=-- |  |  |  |  |  |  |  |  |  |
| NP_459987 | - | probable regulatory protein | - | NP_416088 | dicA | Qin prophage; predicted regulator for DicB | nGCO | 32.98 | 46.95 |
| NP_461563 | - | probable regulatory protein | - | NP_416088 | dicA | Qin prophage; predicted regulator for DicB | nGCO | 32.98 | 46.95 |
| --=-- |  |  |  |  |  |  |  |  |  |
| NP_460782 | sdaA | L-serine deaminase I/L-threonine deaminase I | - | NP_416328 | sdaA | L-serine deaminase I | GCO | 92.29 | 55.53 |
| NP_461141 | - | putative L-serine dehydratase | - | NP_416328 | sdaA | L-serine deaminase I | nGCO | 46.13 | 54.38 |
| --=-- |  |  |  |  |  |  |  |  |  |
| NP_460080 | hpaX | 4-hydroxyphenylacetate catabolism | - | NP_416749 | yfaV | predicted transporter | GCO | 30.42 | 53.08 |
| NP_461216 | - | putative permease | + | NP_416749 | yfaV | predicted transporter | nGCO | 27.92 | 42 |
| NP_461232 | yfaV | putative transport protein | - | NP_416749 | yfaV | predicted transporter | GCO | 85.31 | 54.1 |
| --=-- |  |  |  |  |  |  |  |  |  |
| NP_460818 | pagO | integral membrane protein | + | NP_416468 | yedA | predicted inner membrane protein | nGCO | 23.92 | 41.2 |
| NP_460941 | yedA | putative permease | - | NP_416468 | yedA | predicted inner membrane protein | GCO | 73.15 | 56.02 |
| --=-- |  |  |  |  |  |  |  |  |  |
| NP_459184 | yadE | putative xylanase/chitin deacetylase | - | NP_414672 | yadE | predicted polysaccharide deacetylase lipoprotein | GCO | 81.9 | 50.32 |
| NP_459301 | ybeJ | putative xylanase/chitin deacetylase | + | NP_414672 | yadE | predicted polysaccharide deacetylase lipoprotein | nGCO | 26.18 | 51.58 |
| --=-- |  |  |  |  |  |  |  |  |  |
| NP_462670 | - | putative phosphotransferase system enzyme IIB | - | NP_417602 | agaV | N-acetylgalactosamine-specific enzyme IIB component of PTS | nGCO | 33.12 | 51.85 |
| NP_463395 | - | putative PTS permease | - | NP_417602 | agaV | N-acetylgalactosamine-specific enzyme IIB component of PTS | nGCO | 28.85 | 45.67 |
| --=-- |  |  |  |  |  |  |  |  |  |
| NP_459395 | proY | putative proline transporter | - | NP_414936 | proY | predicted cryptic proline transporter | GCO | 86.59 | 54.12 |
| NP_462041 | - | putative amino acid transporter | - | NP_414936 | proY | predicted cryptic proline transporter | nGCO | 23.98 | 51.53 |
| --=-- |  |  |  |  |  |  |  |  |  |
| NP_459636 | cobD | threonine-phosphate decarboxylase | - | NP_416525 | hisC | histidinol-phosphate aminotransferase | nGCO | 25.66 | 56.62 |
| NP_461018 | hisC | histidinol-phosphate aminotransferase | - | NP_416525 | hisC | histidinol-phosphate aminotransferase | GCO | 89.01 | 55.83 |
| --=-- |  |  |  |  |  |  |  |  |  |
| NP_459397 | - | putative thiol-alkyl hydroperoxide reductase | - | NP_415138 | ahpC | alkyl hydroperoxide reductase, C22 subunit | nGCO | 39.02 | 53.23 |
| NP_459600 | ahpC | alkyl hydroperoxide reductase C22 subunit | - | NP_415138 | ahpC | alkyl hydroperoxide reductase, C22 subunit | GCO | 98.39 | 50.88 |
| --=-- |  |  |  |  |  |  |  |  |  |
| NP_461905 | - | putative integral membrane protein | - | YP_026262 | rarD | predicted chloramphenical resistance permease | nGCO | 32.75 | 42.4 |
| NP_462840 | rarD | chloramphenicol resistance | - | YP_026262 | rarD | predicted chloramphenical resistance permease | GCO | 87.37 | 53.22 |
| --=-- |  |  |  |  |  |  |  |  |  |
| NP_460226 | - | putative ABC-type transport system ATPase component | - | NP_415806 | sapF | predicted antimicrobial peptide transporter subunit | nGCO | 27.46 | 57.36 |
| NP_460654 | sapF | peptide transport protein | - | NP_415806 | sapF | predicted antimicrobial peptide transporter subunit | GCO | 97.76 | 54.27 |
| --=-- |  |  |  |  |  |  |  |  |  |
| NP_460310 | ydiV | hypothetical protein | - | NP_416222 | ydiV | hypothetical protein | GCO | 51.5 | 46.07 |
| NP_460655 | - | hypothetical protein | - | NP_416222 | ydiV | hypothetical protein | nGCO | 29.88 | 48.24 |
| --=-- |  |  |  |  |  |  |  |  |  |
| NP_459994 | - | hypothetical protein | - | NP_415579 | dinI | DNA damage-inducible protein I | nGCO | 47.43 | 46.15 |
| NP_460133 | dinI | DNA damage-inducible protein I | - | NP_415579 | dinI | DNA damage-inducible protein I | GCO | 85.18 | 51.62 |
| NP_460211 | msgA | macrophage survival protein | - | NP_415579 | dinI | DNA damage-inducible protein I | nGCO | 37.87 | 46.25 |
| NP_461174 | - | virulence protein | - | NP_415579 | dinI | DNA damage-inducible protein I | nGCO | 39.7 | 47.76 |
| NP_461556 | - | hypothetical protein | - | NP_415579 | dinI | DNA damage-inducible protein I | nGCO | 47.43 | 46.15 |
| --=-- |  |  |  |  |  |  |  |  |  |
| NP_459861 | - | putative inner membrane protein | + | NP_418615 | ulaB | L-ascorbate-specific enzyme IIB component of PTS | GCO | 25.51 | 38.62 |
| NP_463245 | sgaB | putative PTS enzyme II | - | NP_418615 | ulaB | L-ascorbate-specific enzyme IIB component of PTS | GCO | 99 | 50 |
| --=-- |  |  |  |  |  |  |  |  |  |
| NP_459674 | citA | citrate-proton symporter | - | NP_417082 | kgtP | alpha-ketoglutarate transporter | nGCO | 30.58 | 54.02 |
| NP_461589 | kgtP | alpha-ketoglutarate permease | - | NP_417082 | kgtP | alpha-ketoglutarate transporter | GCO | 83.69 | 51.76 |
| --=-- |  |  |  |  |  |  |  |  |  |
| NP_461896 | fucK | L-fuculokinase | - | NP_417283 | fucK | L-fuculokinase | GCO | 84.74 | 58.7 |
| NP_462680 | - | putative sugar kinase | + | NP_417283 | fucK | L-fuculokinase | nGCO | 23.17 | 49.02 |
| --=-- |  |  |  |  |  |  |  |  |  |
| NP_461883 | gudT | putative D-glucarate permease | - | NP_417269 | gudP | predicted D-glucarate transporter | GCO | 96.66 | 52.24 |
| NP_462598 | - | putative permease | - | NP_417269 | gudP | predicted D-glucarate transporter | nGCO | 46.55 | 47.82 |
| NP_462732 | - | putative permease | - | NP_417269 | gudP | predicted D-glucarate transporter | nGCO | 24.88 | 47.5 |
| --=-- |  |  |  |  |  |  |  |  |  |
| NP_461087 | yegU | putative glycohydrolase | - | NP_416602 | yegU | predicted hydrolase | GCO | 72.15 | 58.5 |
| NP_462948 | - | putative ADP-ribosylglycohydrolase | - | NP_416602 | yegU | predicted hydrolase | nGCO | 25.93 | 57.06 |
| --=-- |  |  |  |  |  |  |  |  |  |
| NP_459351 | - | putative inner membrane protein | - | NP_418699 | yjhB | KpLE2 phage-like element; predicted transporter | nGCO | 29.01 | 53.9 |
| NP_460104 | - | putative sialic acid transporter | + | NP_418699 | yjhB | KpLE2 phage-like element; predicted transporter | GCO | 56.4 | 45.27 |
| NP_463295 | - | putative permease | - | NP_418699 | yjhB | KpLE2 phage-like element; predicted transporter | GCO | 22.3 | 51.5 |
| --=-- |  |  |  |  |  |  |  |  |  |
| NP_460974 | cbiF | vitamin B12 biosynthetic protein | - | NP_417827 | cysG | fused siroheme synthase 1,3-dimethyluroporphyriongen III dehydrogenase and siroheme ferrochelatase/uroporphyrinogen methyltransferase | nGCO | 29.91 | 57.23 |
| NP_462380 | cysG | siroheme synthase | - | NP_417827 | cysG | fused siroheme synthase 1,3-dimethyluroporphyriongen III dehydrogenase and siroheme ferrochelatase/uroporphyrinogen methyltransferase | GCO | 90.37 | 58.07 |
| --=-- |  |  |  |  |  |  |  |  |  |
| NP_459087 | - | putative secreted protein | + | NP_417705 | yhcN | hypothetical protein | nGCO | 35.95 | 46.39 |
| NP_462271 | yhcN | putative outer membrane protein | - | NP_417705 | yhcN | hypothetical protein | GCO | 68.96 | 50.75 |
| NP_462272 | - | putative periplasmic protein | - | NP_417705 | yhcN | hypothetical protein | GCO | 53.4 | 50.18 |
| --=-- |  |  |  |  |  |  |  |  |  |
| NP_459041 | - | putative arylsulfatase regulator | - | YP_026259 | aslB | predicted regulator of arylsulfatase activity | nGCO | 46.32 | 49.7 |
| NP_460253 | - | arylsulfatase regulator | - | YP_026259 | aslB | predicted regulator of arylsulfatase activity | nGCO | 46.05 | 49.95 |
| NP_462038 | - | putative arylsulfatase regulator | - | YP_026259 | aslB | predicted regulator of arylsulfatase activity | nGCO | 36.43 | 49.87 |
| NP_462851 | - | putative arylsulfatase regulator | - | YP_026259 | aslB | predicted regulator of arylsulfatase activity | nGCO | 47.83 | 53.72 |
| --=-- |  |  |  |  |  |  |  |  |  |
| NP_459538 | fimA | fimbrin | + | NP_415063 | sfmA | predicted fimbrial-like adhesin protein | GCO | 65 | 55.24 |
| NP_462541 | lpfA | long polar fimbrial protein A precursor | - | NP_415063 | sfmA | predicted fimbrial-like adhesin protein | nGCO | 38.56 | 46.36 |
| --=-- |  |  |  |  |  |  |  |  |  |
| NP_459169 | - | putative transcriptional regulator | - | NP_417215 | ygbI | predicted DNA-binding transcriptional regulator | nGCO | 34.66 | 55.07 |
| NP_461840 | ygbI | putative regulatory protein | - | NP_417215 | ygbI | predicted DNA-binding transcriptional regulator | GCO | 86.56 | 55.68 |
| --=-- |  |  |  |  |  |  |  |  |  |
| NP_460470 | ydfH | putative regulatory protein | - | NP_416058 | ydfH | predicted DNA-binding transcriptional regulator | GCO | 83.33 | 50.65 |
| NP_461217 | - | putative regulatory protein | + | NP_416058 | ydfH | predicted DNA-binding transcriptional regulator | nGCO | 23.61 | 38.5 |
| NP_462267 | - | putative regulatory protein | - | NP_416058 | ydfH | predicted DNA-binding transcriptional regulator | nGCO | 23.41 | 49.69 |
| --=-- |  |  |  |  |  |  |  |  |  |
| NP_459654 | gltL | glutamate/aspartate transporter | - | NP_415185 | gltL | glutamate and aspartate transporter subunit | GCO | 90.45 | 51.92 |
| NP_460594 | - | putative ABC-type polar amino acid transport system ATPase component | + | NP_415185 | gltL | glutamate and aspartate transporter subunit | nGCO | 39.71 | 43.44 |
| --=-- |  |  |  |  |  |  |  |  |  |
| NP_460494 | - | putative hydrogenase | - | NP_415495 | hyaE | protein involved in processing of HyaA and HyaB proteins | GCO | 36.36 | 53.04 |
| NP_460746 | - | putative chaperone | - | NP_415495 | hyaE | protein involved in processing of HyaA and HyaB proteins | GCO | 73.6 | 57.53 |
| --=-- |  |  |  |  |  |  |  |  |  |
| NP_460223 | - | putative ABC transporter protein | - | NP_418000 | dppB | dipeptide transporter | nGCO | 27.24 | 52.92 |
| NP_462531 | dppB | dipeptide transport protein 1 | - | NP_418000 | dppB | dipeptide transporter | GCO | 97.64 | 56.76 |
| --=-- |  |  |  |  |  |  |  |  |  |
| NP_945159 | - | hypothetical protein | - | YP_588451 | ydaE | Rac prophage; conserved protein | nGCO | 40 | 44.65 |
| NP_461565 | - | hypothetical protein | + | YP_588451 | ydaE | Rac prophage; conserved protein | nGCO | 40 | 45.91 |
| --=-- |  |  |  |  |  |  |  |  |  |
| NP_459182 | stiA | putative fimbrial subunit | - | NP_418734 | fimA | major type 1 subunit fimbrin (pilin) | nGCO | 34.75 | 52.4 |
| NP_459335 | stbA | putative fimbrial major subunit | + | NP_418734 | fimA | major type 1 subunit fimbrin (pilin) | nGCO | 28.93 | 47.11 |
| NP_462537 | lpfE | long polar fimbrial minor protein | - | NP_418734 | fimA | major type 1 subunit fimbrin (pilin) | nGCO | 28.8 | 51.13 |
| --=-- |  |  |  |  |  |  |  |  |  |
| NP_462084 | - | putative periplasmic dicarboxylate-binding protein | - | NP_418036 | yiaO | predicted transporter | nGCO | 27.7 | 51.72 |
| NP_462573 | yiaO | putative periplasmic dicarboxylate-binding protein | - | NP_418036 | yiaO | predicted transporter | GCO | 87.5 | 50.05 |
| NP_462935 | - | putative periplasmic dicarboxylate-binding protein | - | NP_418036 | yiaO | predicted transporter | nGCO | 28.11 | 53.15 |
| --=-- |  |  |  |  |  |  |  |  |  |
| NP_460379 | ssaV | type III secretion system apparatus protein | + | NP_416393 | flhA | flagellar biosynthesis protein A | nGCO | 26.51 | 46.52 |
| NP_460870 | flhA | flagellar biosynthesis protein | - | NP_416393 | flhA | flagellar biosynthesis protein A | GCO | 75.28 | 55.98 |
| NP_461817 | invA | needle complex export protein | - | NP_416393 | flhA | flagellar biosynthesis protein A | nGCO | 28.65 | 45.57 |
| --=-- |  |  |  |  |  |  |  |  |  |
| NP_459566 | - | putative PTS system mannose-specific enzyme IID | - | NP_416333 | manZ | mannose-specific enzyme IID component of PTS | GCO | 34.7 | 56.95 |
| NP_460788 | manZ | mannose-specific enzyme IID | - | NP_416333 | manZ | mannose-specific enzyme IID component of PTS | GCO | 89.47 | 54.58 |
| NP_462668 | - | putative phosphotransferase system enzyme II | - | NP_416333 | manZ | mannose-specific enzyme IID component of PTS | nGCO | 39.19 | 54.07 |
| NP_463397 | - | putative PTS permease | - | NP_416333 | manZ | mannose-specific enzyme IID component of PTS | GCO | 40.5 | 49.58 |
| --=-- |  |  |  |  |  |  |  |  |  |
| NP_460350 | ttrB | tetrathionate reductase complex subunit B | - | NP_418496 | nrfC | formate-dependent nitrite reductase, 4Fe4S subunit | nGCO | 38.91 | 56.3 |
| NP_463144 | nrfC | putative formate-dependent nitrite reductase | - | NP_418496 | nrfC | formate-dependent nitrite reductase, 4Fe4S subunit | GCO | 91.03 | 57.58 |
| --=-- |  |  |  |  |  |  |  |  |  |
| NP_459933 | cydD | cytochrome-related transporter | - | NP_415407 | cydD | fused cysteine transporter subunits of ABC superfamily: membrane component/ATP-binding component | GCO | 86.9 | 56.59 |
| NP_461620 | - | putative ABC transporter transmembrane region | - | NP_415407 | cydD | fused cysteine transporter subunits of ABC superfamily: membrane component/ATP-binding component | nGCO | 24.15 | 56.85 |
| --=-- |  |  |  |  |  |  |  |  |  |
| NP_460087 | scsB | suppression of copper sensitivity protein | - | NP_418559 | dipZ | thiol:disulfide interchange protein precursor | nGCO | 24.52 | 58.08 |
| NP_463187 | dsbD | thiol:disulfide interchange protein precursor | - | NP_418559 | dipZ | thiol:disulfide interchange protein precursor | GCO | 79.4 | 56.57 |
| --=-- |  |  |  |  |  |  |  |  |  |
| NP_461121 | - | putative glutathione S-transferase | - | NP_417696 | sspA | stringent starvation protein A | nGCO | 25.94 | 56.27 |
| NP_462252 | sspA | stringent starvation protein A | - | NP_417696 | sspA | stringent starvation protein A | GCO | 98.1 | 51.64 |
