# Supplementary material for: Gene socialization: gene order, GC content and gene silencing in Salmonella
Source: BMC Genomics. 2009 Dec 11;10:597. doi: 10.1186/1471-2164-10-597 (PMC2801525; doi:10.1186/1471-2164-10-597)
Supplement: Additional file 4 — List of Salmonella nGCO duplicated genes. Table displaying of Salmonella nGCO duplicated genes. [file 1471-2164-10-597-S4.DOC]

| **Salmonela** | **name** | **Salmonela gene description** | **hns** | **K12** | **name** | **K12 gene description** | **identity** | **GC content** |
| --- | --- | --- | --- | --- | --- | --- | --- | --- |
| NP_462044 | - | putative NAD-dependent aldehyde dehydrogenase | - | NP_415903 | feaB | phenylacetaldehyde dehydrogenase | 43.11% | 51.17% |
| NP_463282 | - | putative NAD-dependent aldehyde dehydrogenase | - | NP_415903 | feaB | phenylacetaldehyde dehydrogenase | 34.14% | 56.70% |
| --=-- |  |  |  |  |  |  |  |  |
| NP_459743 | - | transcriptional regulator | + | NP_414872 | cynR | DNA-binding transcriptional dual regulator | 25.61% | 34.90% |
| NP_461510 | - | putative transcriptional regulator | + | NP_414872 | cynR | DNA-binding transcriptional dual regulator | 30.08% | 42.93% |
| --=-- |  |  |  |  |  |  |  |  |
| NP_459166 | kdgT | 2-keto-3-deoxygluconate permease | - | NP_418345 | kdgT | 2-keto-3-deoxygluconate permease | 34.47% | 57.54% |
| NP_459643 | - | 2-keto-3-deoxygluconate permease | - | NP_418345 | kdgT | 2-keto-3-deoxygluconate permease | 31.44% | 52.30% |
| --=-- |  |  |  |  |  |  |  |  |
| NP_459869 | - | putative integrase | - | NP_416096 | intQ | Qin prophage; predicted defective integrase | 55.78% | 47.36% |
| NP_459980 | - | integrase | - | NP_416096 | intQ | Qin prophage; predicted defective integrase | 54.19% | 48.80% |
| --=-- |  |  |  |  |  |  |  |  |
| NP_459768 | hutC | histidine utilization repressor | - | NP_415258 | mngR | DNA-binding transcriptional dual regulator, fatty-acyl-binding | 24.77% | 56.88% |
| NP_462684 | - | putative regulatory protein | - | NP_415258 | mngR | DNA-binding transcriptional dual regulator, fatty-acyl-binding | 29.23% | 40.86% |
| NP_462949 | - | putative regulatory protein | - | NP_415258 | mngR | DNA-binding transcriptional dual regulator, fatty-acyl-binding | 27.92% | 51.03% |
| --=-- |  |  |  |  |  |  |  |  |
| NP_459564 | - | putative phosphosugar isomerase | - | NP_417830 | frlB | fructoselysine-6-P-deglycase | 26.25% | 52.58% |
| NP_462502 | - | putative phosphosugar isomerase | - | NP_417830 | frlB | fructoselysine-6-P-deglycase | 26.59% | 50.30% |
| NP_463399 | - | putative glucosamine-fructose-6-phosphate aminotransferase | - | NP_417830 | frlB | fructoselysine-6-P-deglycase | 26.86% | 50.64% |
| --=-- |  |  |  |  |  |  |  |  |
| NP_461630 | - | DNA invertase-like protein | - | NP_415676 | pin | e14 prophage; site-specific DNA recombinase | 80% | 50.35% |
| NP_461699 | hin | DNA-invertase Hin | - | NP_415676 | pin | e14 prophage; site-specific DNA recombinase | 59.77% | 46.24% |
| --=-- |  |  |  |  |  |  |  |  |
| NP_460227 | - | putative inner membrane protein | - | NP_415846 | ynaI | conserved inner membrane protein | 23.94% | 48.32% |
| NP_460622 | ynaI | putative integral membrane protein | - | NP_415846 | ynaI | conserved inner membrane protein | 71.34% | 44.86% |
| --=-- |  |  |  |  |  |  |  |  |
| NP_459926 | - | SlsA | - | NP_415417 | ycaC | predicted hydrolase | 46.66% | 53.83% |
| NP_462660 | slsA | putative inner membrane protein | + | NP_415417 | ycaC | predicted hydrolase | 47.17% | 52.27% |
| --=-- |  |  |  |  |  |  |  |  |
| NP_459745 | - | putative cation transporter | - | NP_417959 | arsB | arsenite/antimonite transporter | 20.96% | 38.06% |
| NP_462266 | - | putative cation transporter | - | NP_417959 | arsB | arsenite/antimonite transporter | 21.82% | 53.11% |
| --=-- |  |  |  |  |  |  |  |  |
| NP_459990 | - | DNA replication protein DnaC | - | NP_415878 | ydaV | Rac prophage; predicted DNA replication protein | 49.39% | 52.53% |
| NP_461560 | - | DNA replication protein DnaC | - | NP_415878 | ydaV | Rac prophage; predicted DNA replication protein | 49.39% | 52.53% |
| --=-- |  |  |  |  |  |  |  |  |
| NP_459348 | - | putative cation transport ATPase | - | NP_415017 | copA | copper transporter | 42.04% | 60.02% |
| NP_459493 | copA | putative copper-transporting ATPase | - | NP_415017 | copA | copper transporter | 91.47% | 59.15% |
| --=-- |  |  |  |  |  |  |  |  |
| NP_459089 | - | putative sulfatase | + | NP_418134 | yidJ | predicted sulfatase/phosphatase | 22.55% | 46.19% |
| NP_459862 | - | putative sulfatase | + | NP_418134 | yidJ | predicted sulfatase/phosphatase | 24.40% | 51.88% |
| NP_462037 | - | putative arylsulfatase | - | NP_418134 | yidJ | predicted sulfatase/phosphatase | 26.15% | 48.56% |
| --=-- |  |  |  |  |  |  |  |  |
| NP_462165 | agaR | Aga operon transcriptional repressor | - | NP_417600 | agaR | DNA-binding transcriptional dual regulator | 26.93% | 51.79% |
| NP_462175 | - | galactitol utilization operon transcriptional repressor | - | NP_417600 | agaR | DNA-binding transcriptional dual regulator | 35.82% | 50.38% |
| --=-- |  |  |  |  |  |  |  |  |
| NP_460557 | ydcR | putative regulatory protein | - | NP_415956 | ydcR | fused predicted DNA-binding transcriptional regulator/predicted amino transferase | 86.11% | 53.19% |
| NP_461729 | - | putative regulatory protein | - | NP_415956 | ydcR | fused predicted DNA-binding transcriptional regulator/predicted amino transferase | 29.38% | 56.62% |
| --=-- |  |  |  |  |  |  |  |  |
| NP_460434 | ompN | outer membrane protein N precursor | + | NP_415895 | ompN | outer membrane pore protein N, non-specific | 76.96% | 47.61% |
| NP_460490 | - | putative outer membrane protein | - | NP_415895 | ompN | outer membrane pore protein N, non-specific | 62.15% | 46.38% |
| NP_460946 | ompS | putative porin | - | NP_415895 | ompN | outer membrane pore protein N, non-specific | 66.84% | 50.37% |
| --=-- |  |  |  |  |  |  |  |  |
| NP_459298 | safB | putative fimbrial assembly chaparone | + | NP_417612 | yraI | predicted periplasmic pilin chaperone | 35.64% | 48.87% |
| NP_462540 | lpfB | long polar fimbrial chaperone precursor | - | NP_417612 | yraI | predicted periplasmic pilin chaperone | 41.28% | 44.77% |
| --=-- |  |  |  |  |  |  |  |  |
| NP_459515 | - | putative permease | - | NP_418776 | yjiZ | predicted transporter | 20.68% | 46.07% |
| NP_460503 | - | putative transport protein | - | NP_418776 | yjiZ | predicted transporter | 27.10% | 46.62% |
| --=-- |  |  |  |  |  |  |  |  |
| NP_459642 | - | putative hydrolas | - | NP_417597 | garD | (D)-galactarate dehydrogenase | 33.91% | 52.60% |
| NP_462163 | garD | galactarate dehydrogenase | + | NP_417597 | garD | (D)-galactarate dehydrogenase | 91.39% | 56.80% |
| --=-- |  |  |  |  |  |  |  |  |
| NP_459904 | - | putative phage tail assembly protein | - | NP_415891 | tfaR | Rac prophage; predicted tail fiber assembly protein | 61.13% | 47.22% |
| NP_460025 | - | tail fiber assembly like-protein | - | NP_415891 | tfaR | Rac prophage; predicted tail fiber assembly protein | 62.69% | 48.62% |
| NP_460823 | mig-3 | phage-tail assembly-like protein | - | NP_415891 | tfaR | Rac prophage; predicted tail fiber assembly protein | 50.88% | 50.68% |
| NP_461522 | - | phage tail assembly-like protein | - | NP_415891 | tfaR | Rac prophage; predicted tail fiber assembly protein | 59.16% | 47.84% |
| --=-- |  |  |  |  |  |  |  |  |
| NP_459306 | yafV | putative amidohydrolase | - | NP_414754 | yafV | predicted C-N hydrolase family amidase, NAD(P)-binding | 82.35% | 54.16% |
| NP_459623 | ybeM | putative hydrolase | - | NP_414754 | yafV | predicted C-N hydrolase family amidase, NAD(P)-binding | 26.87% | 56.40% |
| --=-- |  |  |  |  |  |  |  |  |
| NP_460501 | - | putative regulatory protein | - | NP_418744 | uxuR | DNA-binding transcriptional repressor | 31.83% | 47.09% |
| NP_462000 | - | putative regulatory protein | - | NP_418744 | uxuR | DNA-binding transcriptional repressor | 40.35% | 44.93% |
| NP_463366 | uxuR | uxu operon transcriptional repressor | - | NP_418744 | uxuR | DNA-binding transcriptional repressor | 89.49% | 54.65% |
| --=-- |  |  |  |  |  |  |  |  |
| NP_461507 | yfhH | putative transport protein | - | NP_417056 | yfhH | predicted DNA-binding transcriptional regulator | 79.07% | 57.24% |
| NP_463278 | - | putative transcriptional regulator | - | NP_417056 | yfhH | predicted DNA-binding transcriptional regulator | 25.09% | 48.20% |
| --=-- |  |  |  |  |  |  |  |  |
| NP_459474 | - | putative transposase | - | NP_416808 | yfcI | hypothetical protein | 49.67% | 53.09% |
| NP_462411 | - | putative cytoplasmic protein | - | NP_416808 | yfcI | hypothetical protein | 67.76% | 52.24% |
| NP_462665 | - | putative cytoplasmic protein | - | NP_416808 | yfcI | hypothetical protein | 62.93% | 50.21% |
| --=-- |  |  |  |  |  |  |  |  |
| NP_459880 | - | putative chaparone | - | NP_416077 | ydfT | Qin prophage; predicted antitermination protein Q | 33.33% | 54.65% |
| NP_459997 | - | putative molecular chaperone | - | NP_416077 | ydfT | Qin prophage; predicted antitermination protein Q | 34.31% | 46.36% |
| --=-- |  |  |  |  |  |  |  |  |
| NP_459836 | - | putative transcriptional regulator | + | NP_415289 | ybhD | predicted DNA-binding transcriptional regulator | 23.86% | 40.17% |
| NP_462734 | - | putative transcriptional regulator | - | NP_415289 | ybhD | predicted DNA-binding transcriptional regulator | 26.75% | 53.40% |
| --=-- |  |  |  |  |  |  |  |  |
| NP_463279 | - | sugar transporter | - | NP_418455 | xylE | D-xylose transporter | 40.86% | 46.79% |
| NP_463280 | - | sugar transporter | - | NP_418455 | xylE | D-xylose transporter | 31.37% | 49.68% |
| --=-- |  |  |  |  |  |  |  |  |
| NP_459987 | - | probable regulatory protein | - | NP_416088 | dicA | Qin prophage; predicted regulator for DicB | 32.98% | 46.95% |
| NP_461563 | - | probable regulatory protein | - | NP_416088 | dicA | Qin prophage; predicted regulator for DicB | 32.98% | 46.95% |
| --=-- |  |  |  |  |  |  |  |  |
| NP_462670 | - | putative phosphotransferase system enzyme IIB | - | NP_417602 | agaV | N-acetylgalactosamine-specific enzyme IIB component of PTS | 33.12% | 51.85% |
| NP_463395 | - | putative PTS permease | - | NP_417602 | agaV | N-acetylgalactosamine-specific enzyme IIB component of PTS | 28.85% | 45.67% |
| --=-- |  |  |  |  |  |  |  |  |
| NP_459041 | - | putative arylsulfatase regulator | - | YP_026259 | aslB | predicted regulator of arylsulfatase activity | 46.32% | 49.70% |
| NP_460253 | - | arylsulfatase regulator | - | YP_026259 | aslB | predicted regulator of arylsulfatase activity | 46.05% | 49.95% |
| NP_462038 | - | putative arylsulfatase regulator | - | YP_026259 | aslB | predicted regulator of arylsulfatase activity | 36.43% | 49.87% |
| NP_462851 | - | putative arylsulfatase regulator | - | YP_026259 | aslB | predicted regulator of arylsulfatase activity | 47.83% | 53.72% |
| --=-- |  |  |  |  |  |  |  |  |
| NP_945159 | - | hypothetical protein | - | YP_588451 | ydaE | Rac prophage; conserved protein | 40% | 44.65% |
| NP_461565 | - | hypothetical protein | + | YP_588451 | ydaE | Rac prophage; conserved protein | 40% | 45.91% |
| --=-- |  |  |  |  |  |  |  |  |
| NP_459182 | stiA | putative fimbrial subunit | - | NP_418734 | fimA | major type 1 subunit fimbrin (pilin) | 34.75% | 52.40% |
| NP_459335 | stbA | putative fimbrial major subunit | + | NP_418734 | fimA | major type 1 subunit fimbrin (pilin) | 28.93% | 47.11% |
| NP_462537 | lpfE | long polar fimbrial minor protein | - | NP_418734 | fimA | major type 1 subunit fimbrin (pilin) | 28.80% | 51.13% |
| --=-- |  |  |  |  |  |  |  |  |
