# Supplementary material for: Gene socialization: gene order, GC content and gene silencing in Salmonella
Source: BMC Genomics. 2009 Dec 11;10:597. doi: 10.1186/1471-2164-10-597 (PMC2801525; doi:10.1186/1471-2164-10-597)
Supplement: Additional file 5 — Genes with no homolog between Salmonella and E. coli k12 that share a homolog between Salmonella and EPEC. Table displaying Genes with no homolog between Salmonella and E. coli k12 that share a homolog between Salmonella and EPEC. [file 1471-2164-10-597-S5.DOC]

| **Salmonela** | **name** | **Salmonela gene description** | **hns** | **GCO** | **EPEC** | **name** | **EPEC gene description** | **%**  **Identity** | **%GC**  **content** |
| --- | --- | --- | --- | --- | --- | --- | --- | --- | --- |
| NP_459021 | - | hypothetical protein | hns- | nGCO | YP_002330153 | - | hypothetical protein | 58.38 | 51.12 |
| NP_459037 | - | putative arylsulfatase | hns- | nGCO | YP_002330803 | - | arylsulfate sulfotransferase | 26.56 | 46.3 |
| NP_459042 | - | putative cytoplasmic protein | hns- | nGCO | YP_002327612 | - | hypothetical protein | 67.15 | 49.92 |
| NP_459043 | - | putative arylsulfatase | hns- | nGCO | YP_002330803 | - | arylsulfate sulfotransferase | 23.91 | 49.7 |
| NP_945154 | - | hypothetical protein | hns- | nGCO | YP_002332092 | yjiW | endoribonuclease SymE | 49.12 | 52.89 |
| NP_459368 | yaiU | flagellar protein | hns+ | nGCO | YP_002330403 | espC | extracellular serine protease EspC | 20.79 | 46.23 |
| NP_951048 | - | hypothetical protein | hns- | nGCO | YP_002328573 | - | hypothetical protein | 84.09 | 51.7 |
| NP_459884 | - | putative chitinase | hns- | nGCO | YP_002330232 | - | predicted endolysin | 61.76 | 53.49 |
| NP_459886 | - | hypothetical protein | hns- | GCO | YP_002328582 | - | hypothetical protein | 40.49 | 51.42 |
| NP_459887 | - | hypothetical protein | hns- | GCO | YP_002328583 | - | predicted terminase large subunit | 63 | 56.7 |
| NP_459888 | - | hypothetical protein | hns- | GCO | YP_002328585 | - | predicted portal protein | 62.23 | 60.48 |
| NP_459889 | - | ATP-dependent protease | hns- | GCO | YP_002328586 | - | predicted protease/scaffold protein | 67.86 | 57.65 |
| NP_945156 | - | hypothetical protein | hns- | GCO | YP_002328587 | - | hypothetical protein | 47.17 | 60.91 |
| NP_945157 | - | hypothetical protein | hns- | GCO | YP_002328588 | - | hypothetical protein | 41.76 | 60.5 |
| NP_459891 | - | putative phage tail component | hns- | GCO | YP_002328589 | - | predicted minor tail protein | 62.5 | 58.63 |
| NP_459892 | - | hypothetical protein | hns- | GCO | YP_002328590 | - | predicted minor tail protein | 65.41 | 54.97 |
| NP_459893 | - | putative major tail protein | hns- | GCO | YP_002328591 | - | predicted major tail protein | 70.09 | 56.85 |
| NP_945158 | - | hypothetical protein | hns- | GCO | YP_002328592 | - | predicted minor tail protein | 56.88 | 56.29 |
| NP_459894 | - | putative minor tail protein | hns- | GCO | YP_002328593 | - | predicted minor tail protein | 65.09 | 58.17 |
| NP_459895 | - | putative minor tail protein | hns- | nGCO | YP_002328278 | - | predicted tail length tape measure protein | 25 | 57.17 |
| NP_459896 | - | putative minor tail protein | hns- | nGCO | YP_002328594 | - | predicted minor tail protein | 68.52 | 55.75 |
| NP_459898 | - | putative minor tail protein | hns- | GCO | YP_002328595 | - | predicted minor tail protein | 71.43 | 58.04 |
| NP_459899 | - | putative phage tail assembly protein | hns- | GCO | YP_002328596 | - | predicted tail assembly protein | 77.82 | 55.78 |
| NP_459900 | - | putative phage tail assembly protein | hns- | nGCO | YP_002328282 | - | predicted tail assembly protein | 64.89 | 58.99 |
| NP_459902 | - | putative host-specificity protein | hns- | nGCO | YP_002328806 | - | predicted host specificity protein | 68.97 | 57.21 |
| NP_459903 | - | putative minor tail protein | hns- | nGCO | YP_002329348 | - | predicted tail fiber protein | 31.84 | 58.23 |
| NP_459923 | tnpA_1 | transposase | hns- | nGCO | YP_002331247 | - | transposase of IS200C | 94.74 | 45.96 |
| NP_459983 | - | hypothetical protein | hns- | nGCO | YP_002330274 | - | RecT family protein | 47.6 | 51.89 |
| NP_459989 | - | probable regulatory protein | hns- | nGCO | YP_002328562 | - | predicted replication protein | 33.18 | 46.74 |
| NP_460005 | - | hypothetical protein | hns- | GCO | YP_002328582 | - | hypothetical protein | 48.1 | 51.49 |
| NP_460006 | - | hypothetical protein | hns- | GCO | YP_002328583 | - | predicted terminase large subunit | 68.27 | 54.93 |
| NP_460007 | - | hypothetical protein | hns- | GCO | YP_002328585 | - | predicted portal protein | 64.29 | 56.84 |
| NP_460008 | - | Clp protease-like protein | hns- | GCO | YP_002328586 | - | predicted protease/scaffold protein | 69.16 | 52.49 |
| NP_460009 | - | putative RecA/RadA recombinase | hns- | GCO | YP_002328587 | - | hypothetical protein | 61.32 | 52.77 |
| NP_460010 | - | ATP-binding sugar transporter-like protein | hns- | GCO | YP_002328588 | - | hypothetical protein | 45.05 | 52.33 |
| NP_460011 | - | probable minor tail protein | hns- | GCO | YP_002328589 | - | predicted minor tail protein | 34.22 | 51.32 |
| NP_460012 | - | probable minor tail protein | hns- | nGCO | YP_002328274 | - | predicted minor tail protein | 56.49 | 55.47 |
| NP_460013 | - | probable major tail protein | hns- | GCO | YP_002328591 | - | predicted major tail protein | 63.27 | 50.8 |
| NP_460014 | - | probable minor tail protein | hns- | GCO | YP_002328592 | - | predicted minor tail protein | 56.06 | 50.12 |
| NP_460015 | - | probable minor tail protein | hns- | GCO | YP_002328593 | - | predicted minor tail protein | 50.94 | 53.63 |
| NP_460016 | - | probable minor tail protein | hns- | nGCO | YP_002328278 | - | predicted tail length tape measure protein | 23.49 | 56.29 |
| NP_460017 | - | probable minor tail protein | hns- | nGCO | YP_002328594 | - | predicted minor tail protein | 61.47 | 51.35 |
| NP_460020 | - | probable minor tail protein | hns- | nGCO | YP_002328280 | - | predicted minor tail protein | 67.1 | 53.73 |
| NP_460021 | - | probable tail assembly protein | hns- | nGCO | YP_002328974 | - | predicted tail assembly protein | 75.63 | 54.6 |
| NP_460022 | - | probable tail assembly protein | hns- | nGCO | YP_002328282 | - | predicted tail assembly protein | 64.62 | 56.87 |
| NP_460023 | - | host specificity protein J | hns- | nGCO | YP_002328976 | - | predicted host specificity protein | 67.56 | 56.61 |
| NP_460024 | - | probable tail fiber protein | hns- | nGCO | YP_002329348 | - | predicted tail fiber protein | 30.67 | 57.6 |
| NP_460219 | - | putative cytoplasmic protein | hns- | nGCO | YP_002328135 | - | hypothetical protein | 26.1 | 50.67 |
| NP_460294 | - | putative outer membrane protein | hns- | nGCO | YP_002328253 | - | SfpA (systemic factor protein A)-like protein | 71.7 | 47.6 |
| NP_460354 | orf319 | putative inner membrane protein | hns- | nGCO | YP_002328161 | ybgA | hypothetical protein | 48.19 | 55.72 |
| NP_460360 | ssaD | virulence protein | hns+ | nGCO | YP_002331400 | escD | T3SS structure protein EscD | 23.06 | 43.64 |
| NP_460363 | sseB | translocation machinery component | hns+ | nGCO | YP_002331398 | espA | translocon EspA | 33.33 | 43.31 |
| NP_460364 | sscA | secretion system chaparone | hns+ | nGCO | YP_002331418 | cesD | chaperone CesD | 29.53 | 50.63 |
| NP_460371 | ssaG | type III secretion system apparatus protein | hns+ | nGCO | YP_002331394 | escF | T3SS structure protein EscF | 35.29 | 38.42 |
| NP_460374 | ssaJ | needle complex inner membrane lipoprotein | hns+ | nGCO | YP_002331415 | escJ | T3SS structure protein EscJ | 37.97 | 42 |
| NP_460377 | ssaL | type III secretion system apparatus protein | hns+ | nGCO | YP_002331399 | speL | secretion switching protein SpeL | 26.11 | 45.62 |
| NP_460385 | ssaS | type III secretion system apparatus protein | hns+ | GCO | YP_002331424 | escS | T3SS structure protein EscS | 31.17 | 41.94 |
| NP_460589 | - | putative inner membrane protein | hns+ | nGCO | YP_002328575 | - | predicted membrane protein | 41.6 | 36.54 |
| NP_460785 | - | putative cytoplasmic protein | hns- | GCO | YP_002329459 | - | hypothetical protein | 56.34 | 41.96 |
| NP_945163 | - | hypothetical protein | hns- | nGCO | YP_002328582 | - | hypothetical protein | 43.68 | 50.37 |
| NP_460910 | tnpA_2 | transposase for IS200 | hns- | nGCO | YP_002331247 | - | transposase of IS200C | 94.74 | 46.07 |
| NP_460985 | pduC | propanediol dehydratase large subunit | hns- | GCO | YP_002329644 | pduC | propanediol dehydratase, large subunit, AdoCbl-dependent | 94.93 | 56.81 |
| NP_460986 | pduD | propanediol dehydratase medium subunit | hns- | GCO | YP_002329645 | pduD | propanediol dehydratase, medium subunit, AdoCbl-dependent | 81.98 | 57.03 |
| NP_460987 | pduE | propanediol dehydratase small subunit | hns- | GCO | YP_002329646 | pduE | propanediol dehydratase, small subunit, AdoCbl-dependent | 84.39 | 56.13 |
| NP_460988 | pduG | propanediol dehydratase reactivation protein | hns- | GCO | YP_002329647 | pduG | propanediol dehydratase reactivation protein PduG | 85.08 | 59.68 |
| NP_460989 | pduH | propanediol dehydratase reactivation protein | hns- | GCO | YP_002329648 | pduH | propanediol dehydratase reactivation protein PduH | 69.83 | 58.11 |
| NP_460991 | pduK | polyhedral body protein | hns- | GCO | YP_002329650 | pduK | propanediol utilization protein PduK | 63.82 | 55.9 |
| NP_460992 | pduL | propanediol utilization protein | hns- | GCO | YP_002329651 | pduL | predicted propanediol utilization protein | 79.71 | 60.18 |
| NP_460993 | pduM | propanediol utilization protein | hns- | GCO | YP_002329652 | pduM | predicted propanediol utilization protein | 49.69 | 61.17 |
| NP_460994 | pduN | polyhedral body protein | hns- | GCO | YP_002329653 | pduN | propanediol utilization protein PduN | 75.56 | 61.23 |
| NP_460995 | pduO | propanediol utilization protein | hns- | GCO | YP_002329654 | pduO | propanediol utilization protein PduO, AdoCbl-dependent | 66.07 | 61.42 |
| NP_460998 | pduS | polyhedral body protein | hns- | GCO | YP_002329657 | pduS | predicted propanediol utilization protein | 78.23 | 61.43 |
| NP_460999 | pduT | polyhedral body protein | hns- | GCO | YP_002329658 | pduT | propanediol utilization protein PduT | 83.15 | 58.37 |
| NP_461081 | - | putative cytoplasmic protein | hns+ | nGCO | YP_002330703 | - | T3SS secreted effector NleB homolog | 53.09 | 37.15 |
| NP_461082 | - | putative cytoplasmic protein | hns+ | nGCO | YP_002331402 | cesT | chaperone CesT | 50.72 | 32.37 |
| NP_461175 | oafA | O-antigen acetylase | hns+ | nGCO | YP_002330236 | - | predicted acyltransferase | 21.07 | 38.46 |
| NP_461387 | - | putative inner membrane protein | hns- | GCO | YP_002330124 | - | predicted inner membrane protein | 54.08 | 43.33 |
| NP_461388 | - | putative cytoplasmic protein | hns- | GCO | YP_002330123 | - | hypothetical protein | 52.94 | 52.97 |
| NP_461406 | tnpA_3 | transposase | hns- | nGCO | YP_002331247 | - | transposase of IS200C | 94.74 | 45.96 |
| NP_461451 | sinI | putative outer membrane protein | hns- | GCO | YP_002330284 | - | predicted outer membrane protein | 40.18 | 53.12 |
| NP_461524 | - | tail fiber-like protein | hns- | nGCO | YP_002329348 | - | predicted tail fiber protein | 34.01 | 57.9 |
| NP_461525 | - | host specificity protein-J-like | hns- | nGCO | YP_002328283 | - | predicted host specificity protein | 76.61 | 57.02 |
| NP_461526 | - | tail assembly protein I-like | hns- | nGCO | YP_002328805 | - | predicted tail assembly protein | 77.67 | 60.64 |
| NP_461527 | - | tail assembly protein K-like | hns- | GCO | YP_002328596 | - | predicted tail assembly protein | 84.18 | 58.33 |
| NP_461528 | - | phage tail component L-like protein | hns- | GCO | YP_002328595 | - | predicted minor tail protein | 75 | 52.93 |
| NP_461529 | - | phage tail component M-like protein | hns- | GCO | YP_002328594 | - | predicted minor tail protein | 70.37 | 48.48 |
| NP_461530 | - | phage tail component H-like protein | hns- | nGCO | YP_002328278 | - | predicted tail length tape measure protein | 23.41 | 56.42 |
| NP_461531 | - | minor tail-like protein | hns- | GCO | YP_002328593 | - | predicted minor tail protein | 62.39 | 55.16 |
| NP_461532 | - | minor tail-like protein | hns- | GCO | YP_002328592 | - | predicted minor tail protein | 56.49 | 53.28 |
| NP_461533 | - | major tail-like protein | hns- | GCO | YP_002328591 | - | predicted major tail protein | 64.53 | 56.09 |
| NP_461534 | - | hypothetical protein | hns- | GCO | YP_002328590 | - | predicted minor tail protein | 67.67 | 50.49 |
| NP_461536 | - | minor tail protein Z-like | hns- | nGCO | YP_002328589 | - | predicted minor tail protein | 80.21 | 52.33 |
| NP_461537 | - | minor capsid protein FII | hns- | GCO | YP_002328272 | - | predicted head-tail adapotor | 50.43 | 56.51 |
| NP_461538 | - | DNA packaging-like protein | hns- | GCO | YP_002328271 | - | predicted DNA packaging protein | 30.23 | 54.72 |
| NP_461539 | - | phage head-like protein | hns- | GCO | YP_002328270 | - | predicted major capsid protein | 86.22 | 53.74 |
| NP_461540 | - | phage head-like protein | hns- | GCO | YP_002328269 | - | predicted head-DNA stabilization protein | 73.87 | 58.9 |
| NP_461541 | - | head-tail preconnector-like protein | hns- | GCO | YP_002328268 | - | predicted head protein/prohead protease | 60.88 | 56.57 |
| NP_461542 | - | head-tail preconnector-like protein | hns- | GCO | YP_002328267 | - | predicted portal protein | 79.01 | 56.6 |
| NP_461543 | - | head-to-tail joining-like protein | hns- | GCO | YP_002328803 | - | predicted head-tail joining protein | 52.94 | 57.84 |
| NP_461544 | - | terminase-like large protein | hns- | GCO | YP_002328802 | - | predicted terminase large subunit | 82.34 | 51.34 |
| NP_461552 | - | antiterminator-like protein | hns- | nGCO | YP_002328946 | - | predicted antitermination protein | 66.97 | 51.32 |
| NP_461561 | - | replication protein 15-like | hns- | nGCO | YP_002328562 | - | predicted replication protein | 33.18 | 46.74 |
| NP_461568 | - | enterohemolysin 1-like protein | hns- | nGCO | YP_002330274 | - | RecT family protein | 47.6 | 51.71 |
| NP_461623 | - | late control-like protein | hns- | GCO | YP_002328407 | - | predicted late gene regulator | 83.06 | 53.67 |
| NP_461624 | - | putative phage tail protein | hns- | GCO | YP_002328406 | - | predicted tail protein | 80.62 | 53.49 |
| NP_461625 | - | phage tail-like protein | hns- | GCO | YP_002328405 | - | predicted tail tape measure protein | 60.58 | 57.83 |
| NP_461626 | - | gpE-like protein | hns- | GCO | YP_002328404 | - | predicted tail protein | 89.74 | 58.33 |
| NP_461627 | - | putative phage tail-like protein | hns- | GCO | YP_002328403 | - | predicted tail protein | 90 | 55.77 |
| NP_461628 | - | phage tail fiber-like protein | hns- | GCO | YP_002328402 | - | predicted major tail tube protein | 88.3 | 56 |
| NP_461629 | - | phage tail sheath-like protein | hns- | GCO | YP_002328401 | - | predicted major tail sheath protein | 89.74 | 57.97 |
| NP_461634 | - | phage tail-like protein | hns- | nGCO | YP_002329348 | - | predicted tail fiber protein | 40.25 | 52.44 |
| NP_461635 | - | phage tail-like protein | hns- | GCO | YP_002328395 | - | predicted tail protein | 92.04 | 56.1 |
| NP_461636 | - | phage tail-like protein | hns- | GCO | YP_002328396 | - | predicted baseplate assembly protein | 85.43 | 58.85 |
| NP_461637 | - | base plate tail-like protein | hns- | GCO | YP_002328397 | - | predicted baseplate assembly protein | 84.75 | 56.94 |
| NP_461638 | - | phage baseplate assembly-like protein | hns- | GCO | YP_002328398 | - | predicted baseplate assembly protein | 85.94 | 58.37 |
| NP_461640 | - | phage tail-like protein | hns- | nGCO | YP_002328393 | - | predicted tail protein | 83.22 | 55.09 |
| NP_461642 | - | lysis-like protein | hns- | nGCO | YP_002328392 | - | predicted regulatory protein | 73.05 | 60.83 |
| NP_461644 | - | phage-holin-like protein | hns- | GCO | YP_002328389 | - | predicted secretory protein | 80.28 | 50.92 |
| NP_461645 | - | phage tail-like protein | hns- | GCO | YP_002328388 | - | predicted tail protein | 92.54 | 60.78 |
| NP_461646 | - | head completion-like protein | hns- | GCO | YP_002328387 | - | predicted capsid completion protein | 88.96 | 59.56 |
| NP_461647 | - | terminase-like protein | hns- | GCO | YP_002328386 | - | predicted terminase, endonuclease subunit | 82.95 | 61.77 |
| NP_461648 | - | major capsid-like protein | hns- | GCO | YP_002328385 | - | predicted major capsid protein | 83.38 | 54.1 |
| NP_461649 | - | capsid scaffold-like protein | hns- | GCO | YP_002328384 | - | predicted capsid scaffolding protein | 81.95 | 58.27 |
| NP_461650 | - | terminase-like protein | hns- | GCO | YP_002328383 | - | predicted terminase, ATPase subunit | 97.28 | 56.87 |
| NP_461651 | - | portal vertex-like protein | hns- | GCO | YP_002328382 | - | predicted capsid portal protein | 89.94 | 52.92 |
| NP_461654 | - | hypothetical protein | hns- | GCO | YP_002328377 | - | predicted DNA-damage-inducible protein | 100 | 45.09 |
| NP_461655 | - | hypothetical protein | hns- | GCO | YP_002328376 | - | hypothetical protein | 96.77 | 44.44 |
| NP_461656 | - | hypothetical protein | hns- | GCO | YP_002328375 | - | predicted replication protein | 87.31 | 56.29 |
| NP_461658 | - | hypothetical protein | hns- | GCO | YP_002328373 | - | predicted transcriptional regulator, TraR/DksA family | 74.67 | 57.89 |
| NP_461659 | - | hypothetical protein | hns- | GCO | YP_002328372 | - | hypothetical protein | 80.52 | 53.41 |
| NP_461660 | - | hypothetical protein | hns- | GCO | YP_002328371 | - | hypothetical protein | 85.84 | 44.44 |
| NP_461663 | - | hypothetical protein | hns- | nGCO | YP_002328369 | - | predicted regulatory protein | 91.72 | 53.72 |
| NP_461665 | - | hypothetical protein | hns- | nGCO | YP_002328367 | - | predicted repressor protein | 39.7 | 42.96 |
| NP_461671 | - | putative cytoplasmic protein | hns- | nGCO | YP_002331363 | - | hypothetical protein | 46.55 | 55.55 |
| NP_461683 | - | putative sugar phosphate aminotransferase | hns- | nGCO | YP_002331590 | - | predicted phosphosugar isomerase | 41.25 | 57.8 |
| NP_461714 | - | tricarboxylic transport | hns- | nGCO | YP_002327662 | - | hypothetical protein | 32.75 | 57.16 |
| NP_461742 | - | putative glycoporin | hns- | nGCO | YP_002331663 | - | predicted glycoprotein | 60.04 | 47.48 |
| NP_461792 | prgK | needle complex inner membrane lipoprotein | hns- | nGCO | YP_002331415 | escJ | T3SS structure protein EscJ | 26.34 | 45.71 |
| NP_461810 | spaQ | needle complex export protein | hns- | GCO | YP_002331424 | escS | T3SS structure protein EscS | 31.58 | 46.74 |
| NP_461819 | invG | outer membrane secretin precursor | hns- | nGCO | YP_002331417 | escC | T3SS structure protein EscC | 29.2 | 46.41 |
| NP_461826 | - | putative acetyltransferase | hns- | nGCO | YP_002328724 | - | predicted acetyltransferase | 24.5 | 47.34 |
| NP_461844 | - | putative cytoplasmic protein | hns- | GCO | YP_002330489 | - | hypothetical protein | 91.03 | 52.74 |
| NP_951054 | - | hypothetical protein | hns- | GCO | YP_002330580 | ygdT | hypothetical protein | 70.83 | 41.49 |
| NP_945169 | - | hypothetical protein | hns- | nGCO | YP_002331419 | grlA | positive regulator GrlA | 39.52 | 52.63 |
| NP_461949 | tnpA_4 | transposase | hns- | nGCO | YP_002331247 | - | transposase of IS200C | 94.74 | 45.96 |
| NP_461987 | - | putative DNA-binding protein | hns- | GCO | YP_002330652 | - | predicted DNA-binding protein | 79.14 | 56.1 |
| NP_461988 | - | putative inner membrane protein | hns- | GCO | YP_002330653 | - | predicted transporter | 76.56 | 56.64 |
| NP_461989 | - | putative ABC-type cobalt transport system permease component | hns- | GCO | YP_002330654 | - | predicted lipoprotein | 76.17 | 59.74 |
| NP_462001 | - | putative outer membrane lipoprotein | hns+ | GCO | YP_002330666 | - | hypothetical protein | 72.04 | 48.22 |
| NP_462169 | - | putative phosphotransferase system mannitol/fructose-specific IIA domain | hns- | nGCO | YP_002330661 | cmtB | putative mannitol phosphotransferase subunit EIIA | 25.9 | 53.4 |
| NP_462382 | tnpA_5 | transposase for IS200 | hns- | nGCO | YP_002331247 | - | transposase of IS200C | 94.74 | 45.96 |
| NP_462552 | - | putative acetyltransferase | hns- | GCO | YP_002328724 | - | predicted acetyltransferase | 49.04 | 48.97 |
| NP_462553 | - | putative cytoplasmic protein | hns- | GCO | YP_002328725 | - | hypothetical protein | 39.51 | 47.56 |
| NP_462570 | - | putative chemotaxis protein | hns- | GCO | YP_002331293 | - | hypothetical protein | 75.08 | 51.87 |
| NP_462613 | rfaL | O-antigen ligase | hns- | GCO | YP_002331332 | rfaL | lipid A-core: surface polymer ligase | 59.75 | 39.25 |
| NP_462614 | rfaK | putative hexose transferase | hns- | GCO | YP_002331333 | - | lipopolysaccharide 1,2-N-acetylglucosamine transferase | 72.82 | 41.18 |
| NP_462745 | - | putative reverse transcriptase | hns- | nGCO | YP_002331352 | - | predicted RNA-directed DNA polymerase | 30.45 | 30.55 |
| NP_462866 | - | putative cytoplasmic protein | hns- | nGCO | YP_002330803 | - | arylsulfate sulfotransferase | 47.32 | 50.23 |
| NP_945170 | - | hypothetical protein | hns- | GCO | YP_002331649 | - | hypothetical protein | 70.87 | 44.23 |
| NP_462913 | - | putative regulatory protein | hns- | GCO | YP_002331650 | - | hypothetical protein | 87.23 | 45.01 |
| NP_462979 | - | putative arylsulfate sulfotransferase | hns- | nGCO | YP_002330803 | - | arylsulfate sulfotransferase | 46.07 | 47.96 |
| NP_462984 | - | putative cytoplasmic protein | hns- | GCO | YP_002331706 | - | hypothetical protein | 58.42 | 51.46 |
| NP_463026 | - | putative cytoplasmic protein | hns+ | nGCO | YP_002330703 | - | T3SS secreted effector NleB homolog | 61.76 | 33.82 |
| NP_463066 | - | putative phage tail protein | hns- | nGCO | YP_002329347 | - | predicted tail protein | 28.02 | 46.6 |
| NP_463067 | - | putative phage baseplate protein | hns- | nGCO | YP_002328396 | - | predicted baseplate assembly protein | 32.09 | 58.24 |
| NP_463072 | - | putative phage baseplate component | hns- | nGCO | YP_002328398 | - | predicted baseplate assembly protein | 29.44 | 58.09 |
| NP_463073 | - | putative cytoplasmic protein | hns- | nGCO | YP_002330384 | - | predicted late gene regulator | 29.87 | 55.36 |
| NP_463077 | - | putative phage tail core protein | hns- | nGCO | YP_002330380 | - | predicted tail tube protein | 29.01 | 44.57 |
| NP_463078 | - | putative phage tail sheath protein | hns- | nGCO | YP_002328401 | - | predicted major tail sheath protein | 31.05 | 54.97 |
| NP_463176 | tnpA_6 | transposase for IS200 | hns- | nGCO | YP_002331247 | - | transposase of IS200C | 94.74 | 45.96 |
| NP_463182 | - | hypothetical protein | hns- | GCO | YP_002328725 | - | hypothetical protein | 71.13 | 54.76 |
| NP_463183 | - | putative acetyltransferase | hns- | GCO | YP_002328724 | - | predicted acetyltransferase | 73.91 | 57.11 |
| NP_463288 | - | putative endonuclease | hns- | nGCO | YP_002331984 | - | hypothetical protein | 24.84 | 54.43 |
| NP_463309 | - | putative phosphotransferase system mannitol/fructose-specific IIA domain | hns- | nGCO | YP_002331389 | - | predicted transcriptional antiterminator | 21.06 | 54.12 |
