# Supplementary material for: Gene socialization: gene order, GC content and gene silencing in Salmonella
Source: BMC Genomics. 2009 Dec 11;10:597. doi: 10.1186/1471-2164-10-597 (PMC2801525; doi:10.1186/1471-2164-10-597)
Supplement: Additional file 6 — Salmonella genes whose order switches from nGCO to GCO when the reference genome is K12 and EPEC, respectively. Table displaying Salmonella genes whose order switches from nGCO to GCO when the reference genome is K12 and EPEC, respectively. [file 1471-2164-10-597-S6.DOC]

| **Salmonela** | **name** | **Salmonela gene description** | **hns** | **K12** | **name** | **K12 gene description** | **K12_GCO** | **identity** | **EPEC** | **name** | **Epec gene description** | **EPEC**  **GCO** | **identity** | **gc** |
| --- | --- | --- | --- | --- | --- | --- | --- | --- | --- | --- | --- | --- | --- | --- |
| NP_459039 | - | putative outer membrane/exported protein | hns- | NP_416903 | yfeN | conserved outer membrane protein | nGCO | 34.71 | YP_002327610 | - | hypothetical protein | GCO | 55.24 | 44.22 |
| NP_459040 | - | putative arylsulfatase | hns+ | NP_416015 | ydeN | hypothetical protein | nGCO | 25.13 | YP_002327611 | - | predicted arylsulfatase | GCO | 87.93 | 52.4 |
| NP_459046 | - | putative glycosyl hydrolase | hns+ | NP_418113 | yicI | predicted alpha-glucosidase | nGCO | 25.33 | YP_002331390 | yicI | alpha-xylosidase YicI | GCO | 25.8 | 54.6 |
| NP_459167 | - | putative inner membrane protein | hns- | NP_417217 | ygbK | hypothetical protein | nGCO | 25.62 | YP_002328139 | - | predicted inner membrane protein | GCO | 37.56 | 57.78 |
| NP_459168 | - | 4-hydroxythreonine-4-phosphate dehydrogenase | hns- | NP_414594 | pdxA | 4-hydroxythreonine-4-phosphate dehydrogenase | nGCO | 37.08 | YP_002328140 | pdxA | 4-hydroxythreonine-4-phosphate dehydrogenase 2 | GCO | 75.78 | 55.69 |
| NP_459169 | - | putative transcriptional regulator | hns- | NP_417215 | ygbI | predicted DNA-binding transcriptional regulator | nGCO | 34.66 | YP_002328141 | - | predicted transcriptional regulator, DeoR family | GCO | 70.09 | 55.07 |
| NP_459299 | safC | putative fimbrial usher | hns+ | NP_418737 | fimD | outer membrane usher protein, type 1 fimbrial synthesis | nGCO | 35.17 | YP_002332072 | fimD | outer membrane usher protein, type 1 fimbrial synthesis | GCO | 35.07 | 54.67 |
| NP_459333 | stbC | putative fimbrial usher | hns- | NP_418737 | fimD | outer membrane usher protein, type 1 fimbrial synthesis | nGCO | 30.8 | YP_002332072 | fimD | outer membrane usher protein, type 1 fimbrial synthesis | GCO | 30.88 | 53.94 |
| NP_459525 | ylbE | putative cytoplasmic protein | hns- | NP_414855 | yahG | hypothetical protein | nGCO | 43.47 | YP_002328027 | ylbE | hypothetical protein | GCO | 90.91 | 57.14 |
| NP_459539 | fimI | fimbrial protein | hns+ | NP_418735 | fimI | fimbrial protein involved in type 1 pilus biosynthesis | nGCO | 35.09 | YP_002332070 | fimI | fimbrial protein involved in type 1 pilus biosynthesis | GCO | 34.44 | 54.11 |
| NP_459623 | ybeM | putative hydrolase | hns- | NP_414754 | yafV | predicted C-N hydrolase family amidase, NAD(P)-binding | nGCO | 26.87 | YP_002328098 | ybeM | predicted amidase | GCO | 72.9 | 56.4 |
| NP_459715 | gltA | citrate synthase | hns- | NP_415248 | gltA | citrate synthase | nGCO | 96.25 | YP_002328170 | gltA | type II citrate synthase | GCO | 96.25 | 52.72 |
| NP_460379 | ssaV | type III secretion system apparatus protein | hns+ | NP_416393 | flhA | flagellar biosynthesis protein A | nGCO | 26.51 | YP_002331411 | escV | translocator EscV | GCO | 39.36 | 46.52 |
| NP_460380 | ssaN | type III secretion system ATPase | hns+ | NP_416451 | fliI | flagellum-specific ATP synthase | nGCO | 42.01 | YP_002331410 | escN | translocator EscN | GCO | 48.66 | 51.22 |
| NP_460384 | ssaR | needle complex export protein | hns+ | NP_416458 | fliP | flagellar biosynthesis protein P | nGCO | 40.2 | YP_002331425 | escR | type III secretion system protein | GCO | 54.4 | 42.9 |
| NP_460386 | ssaT | type III secretion system apparatus protein | hns+ | NP_416460 | fliR | flagellar biosynthesis protein R | nGCO | 25.13 | YP_002331423 | escT | T3SS structure protein EscT | GCO | 39.23 | 38.97 |
| NP_460387 | ssaU | type III secretion system apparatus protein | hns+ | NP_416394 | flhB | flagellar biosynthesis protein B | nGCO | 29.71 | YP_002331422 | escU | secretion system apparatus protein SsaU | GCO | 38.26 | 39.66 |
| NP_460622 | ynaI | putative integral membrane protein | hns- | NP_415846 | ynaI | conserved inner membrane protein | nGCO | 71.34 | YP_002329059 | ynaI | conserved inner membrane protein | GCO | 71.34 | 44.86 |
| NP_460982 | pduF | propanediol diffusion facilitator | hns- | NP_418362 | glpF | glycerol facilitator | nGCO | 62.54 | YP_002329641 | pduF | propanediol diffusion facilitator | GCO | 78.46 | 50.94 |
| NP_460983 | pduA | polyhedral body protein | hns- | NP_416952 | cchA | predicted carboxysome structural protein, ethanolamine utilization protein | nGCO | 47.19 | YP_002329642 | pduA | propanediol utilization protein PduA | GCO | 73.12 | 53.68 |
| NP_460984 | pudB | polyhedral body protein | hns- | NP_416934 | eutL | predicted carboxysome structural protein with predicted role in ethanolamine utilization | nGCO | 29.77 | YP_002329643 | pduB | propanediol utilization protein PduB | GCO | 91.42 | 60.39 |
| NP_460990 | pduJ | polyhedral body protein | hns- | NP_416952 | cchA | predicted carboxysome structural protein, ethanolamine utilization protein | nGCO | 44.7 | YP_002329649 | pduJ | propanediol utilization protein PduJ | GCO | 70.33 | 56.15 |
| NP_460996 | pduP | CoA-dependent propionaldehyde dehydrogenase | hns- | NP_416950 | eutE | predicted aldehyde dehydrogenase, ethanolamine utilization protein | nGCO | 41.06 | YP_002329655 | pduP | CoA-dependent proprionaldehyde dehydrogenase | GCO | 77.37 | 58.63 |
| NP_460997 | pduQ | propanol dehydrogenase | hns- | NP_416948 | eutG | predicted alcohol dehydrogenase in ethanolamine utilization | nGCO | 37.36 | YP_002329656 | pduQ | propanol dehydrogenase | GCO | 78.45 | 60.37 |
| NP_461012 | sbcB | exonuclease I | hns- | NP_416515 | sbcB | exonuclease I | nGCO | 93.16 | YP_002329665 | sbcB | exonuclease I | GCO | 93.16 | 54.43 |
| NP_461119 | yohI | putative regulatory protein | hns- | NP_416645 | dusC | tRNA-dihydrouridine synthase C | nGCO | 89.74 | YP_002329792 | dusC | tRNA-dihydrouridine synthase C | GCO | 90.06 | 57.29 |
| NP_461452 | sinH | intimin-like protein | hns- | NP_415738 | ychP | predicted invasin | nGCO | 31.09 | YP_002330285 | - | intimin-like protein | GCO | 69.35 | 52.16 |
| NP_461545 | - | DNA packaging-like protein | hns- | NP_416066 | nohA | Qin prophage; predicted packaging protein | nGCO | 58.02 | YP_002328801 | - | predicted terminase small subunit | GCO | 81.13 | 52.12 |
| NP_461622 | - | late control-like protein | hns- | NP_416586 | ogrK | DNA-binding transcriptional regulator prophage P2 remnant | nGCO | 52.23 | YP_002328408 | - | predicted transcriptional activator | GCO | 69.01 | 44.29 |
| NP_461630 | - | DNA invertase-like protein | hns- | NP_415676 | pin | e14 prophage; site-specific DNA recombinase | nGCO | 80 | YP_002328400 | - | predicted DNA invertase | GCO | 76.09 | 50.35 |
| NP_461643 | - | probable prophage lysozyme | hns- | NP_415087 | ybcS | DLP12 prophage; predicted lysozyme | nGCO | 34.19 | YP_002328390 | - | predicted lysozyme | GCO | 91.67 | 60.37 |
| NP_461657 | - | DNA adenine methylase-like protein | hns- | NP_417846 | dam | DNA adenine methylase | nGCO | 46.92 | YP_002328374 | - | DNA adenine methylase | GCO | 73.59 | 46.15 |
| NP_461738 | - | putative inner membrane protein | hns- | YP_026179 | ygaY | predicted transporter (pseudogene) | nGCO | 69.62 | YP_002330432 | ygaY | predicted transporter | GCO | 74.87 | 58.98 |
| NP_461808 | spaS | type III secretion protein | hns- | NP_416394 | flhB | flagellar biosynthesis protein B | nGCO | 21.47 | YP_002331422 | escU | secretion system apparatus protein SsaU | GCO | 27.85 | 43.32 |
| NP_461809 | spaR | needle complex export protein | hns- | NP_416460 | fliR | flagellar biosynthesis protein R | nGCO | 19.62 | YP_002331423 | escT | T3SS structure protein EscT | GCO | 29.11 | 49.36 |
| NP_461811 | spaP | needle complex export protein | hns- | NP_416458 | fliP | flagellar biosynthesis protein P | nGCO | 29.71 | YP_002331425 | escR | type III secretion system protein | GCO | 34.74 | 41.48 |
| NP_461842 | - | putative flavoprotein | hns- | NP_416814 | ubiX | 3-octaprenyl-4-hydroxybenzoate carboxy-lyase | nGCO | 55.91 | YP_002330491 | - | predicted phenylacrylic acid decarboxylase | GCO | 85.2 | 57.74 |
| NP_461843 | - | putative 3-polyprenyl-4-hydroxybenzoate decarboxylase | hns- | NP_418285 | ubiD | 3-octaprenyl-4-hydroxybenzoate decarboxylase | nGCO | 27.3 | YP_002330490 | - | predicted 4-hydroxybenzoate decarboxylase | GCO | 96.42 | 55.53 |
| NP_461980 | iciA | chromosome replication initiation inhibitor protein | hns- | NP_417391 | argP | chromosome replication initiation inhibitor protein | nGCO | 91.91 | YP_002330644 | argP | chromosome replication initiation inhibitor protein | GCO | 91.92 | 57.94 |
| NP_461997 | - | putative malate/L-lactate dehydrogenase | hns- | NP_415050 | allD | ureidoglycolate dehydrogenase | nGCO | 40.6 | YP_002330774 | - | predicted malate/L-lactate dehydrogenase-family protein | GCO | 77.78 | 51.09 |
| NP_461998 | - | putative zinc-binding dehydrogenase | hns- | NP_418778 | yjjN | predicted oxidoreductase, Zn-dependent and NAD(P)-binding | nGCO | 46.74 | YP_002330773 | - | predicted zinc-binding dehydrogenase | GCO | 64.5 | 51.72 |
| NP_462000 | - | putative regulatory protein | hns- | NP_418744 | uxuR | DNA-binding transcriptional repressor | nGCO | 40.35 | YP_002330771 | - | predicted regulator | GCO | 78.88 | 44.93 |
| NP_462084 | - | putative periplasmic dicarboxylate-binding protein | hns- | NP_418036 | yiaO | predicted transporter | nGCO | 27.7 | YP_002330775 | - | predicted TRAP-type C4-dicarboxylate transport system, periplasmic component | GCO | 83.79 | 51.72 |
| NP_462108 | - | putative disulfide bond formation protein | hns+ | NP_418297 | dsbA | periplasmic protein disulfide isomerase I | nGCO | 29.27 | YP_002330804 | - | predicted disulfide isomerase, DsbA family | GCO | 84.23 | 46.13 |
| NP_462109 | - | putative disulfide oxidoreductase | hns+ | NP_415703 | dsbB | disulfide bond formation protein B | nGCO | 30.23 | YP_002330805 | - | putative disulfide oxidoreductase | GCO | 87.33 | 51.03 |
| NP_462168 | - | putative phosphotransferase system fructose-specific component IIB | hns- | NP_416672 | fruA | fused fructose-specific PTS enzymes: IIBcomponent/IIC components | nGCO | 37.97 | YP_002329819 | fruA | fructose-specific PTS system IIBC component | GCO | 37.98 | 47.33 |
| NP_462175 | - | galactitol utilization operon transcriptional repressor | hns- | NP_417600 | agaR | DNA-binding transcriptional dual regulator | nGCO | 35.82 | YP_002329743 | gatR | DNA-binding transcriptional repressor GatR of galactitol utilization | GCO | 72.76 | 50.38 |
| NP_462536 | yhjW | putative membrane-associated metal-dependent hydrolase | hns- | NP_418002 | eptB | predicted metal dependent hydrolase | nGCO | 85.76 | YP_002331259 | eptB | phosphoethanolamine transferase | GCO | 85.77 | 52.12 |
| NP_462540 | lpfB | long polar fimbrial chaperone precursor | hns- | NP_417612 | yraI | predicted periplasmic pilin chaperone | nGCO | 41.28 | YP_002331263 | lpfB | predicted fimbrial chaperone | GCO | 66.82 | 44.77 |
| NP_462541 | lpfA | long polar fimbrial protein A precursor | hns- | NP_415063 | sfmA | predicted fimbrial-like adhesin protein | nGCO | 38.56 | YP_002331264 | lpfA | predicted fimbrial major protein precursor | GCO | 74.51 | 46.36 |
| NP_462792 | ilvG | acetolactate synthase II large subunit | hns- | NP_418127 | ilvB | acetolactate synthase large subunit | nGCO | 46.89 | YP_002331529 | ilvG | acetolactate synthase 2 catalytic subunit | GCO | 92.34 | 56.34 |
| NP_462912 | - | putative acetyl esterase | hns- | NP_415009 | aes | acetyl esterase | nGCO | 31.41 | YP_002331648 | - | predicted lipase | GCO | 74.92 | 55.8 |
| NP_462935 | - | putative periplasmic dicarboxylate-binding protein | hns- | NP_418036 | yiaO | predicted transporter | nGCO | 28.11 | YP_002330775 | - | predicted TRAP-type C4-dicarboxylate transport system, periplasmic component | GCO | 38.97 | 53.15 |
| NP_462985 | - | putative 5'-nucleotidase/2',3'-cyclic phosphodiesterase | hns- | NP_415013 | ushA | UDP-sugar hydrolase | nGCO | 23.54 | YP_002331707 | - | predicted 5'-nucleotidase/2', 3'-cyclic phosphodiesterase | GCO | 92.07 | 51.63 |
| NP_463328 | yjgK | putative cytoplasmic protein | hns- | NP_418673 | yjgK | hypothetical protein | nGCO | 80 | YP_002332026 | yjgK | hypothetical protein | GCO | 80.67 | 53.64 |
