# Supplementary material for: Gene socialization: gene order, GC content and gene silencing in Salmonella
Source: BMC Genomics. 2009 Dec 11;10:597. doi: 10.1186/1471-2164-10-597 (PMC2801525; doi:10.1186/1471-2164-10-597)
Supplement: Additional file 7 — Salmonella genes that were excluded from Ka/Ks ratio calculations. Table displaying Salmonella genes that were excluded from Ka/Ks ratio calculations. [file 1471-2164-10-597-S7.DOC]

| **Salmonela** | **name** | ***Salmonella* gene description** |
| --- | --- | --- |
| NP_462295 | fis | DNA-binding protein Fis |
| NP_462343 | rplD | 50S ribosomal protein L4 |
| NP_462255 | rplM | 50S ribosomal protein L13 |
| NP_462330 | rpsH | 30S ribosomal protein S8 |
| NP_462352 | rpsL | 30S ribosomal protein S12 |
| NP_462741 | - | hypothetical protein |
| NP_459480 | ybaB | hypothetical protein |
| NP_462641 | rpoZ | DNA-directed RNA polymerase omega subunit |
| NP_460302 | rplT | 50S ribosomal protein L20 |
| NP_462739 | rpmH | 50S ribosomal protein L34 |
| NP_462327 | rpsE | 30S ribosomal protein S5 |
| NP_460919 | yedF | putative transcriptional regulator |
| NP_462321 | rpsK | 30S ribosomal protein S11 |
| NP_460793 | cspC | cold shock protein |
| NP_462284 | mreB | rod shape-determining protein |
| NP_462333 | rplX | 50S ribosomal protein L24 |
| NP_462339 | rplV | 50S ribosomal protein L22 |
| NP_459929 | infA | translation initiation factor IF-1 |
| NP_462806 | trxA | thioredoxin |
| NP_462550 | cspA | major cold shock protein |
| NP_461747 | csrA | carbon storage regulator |
| NP_461366 | ptsH | phosphohistidinoprotein-hexose phosphotransferase |
| NP_460166 | acpP | acyl carrier protein |
| NP_462338 | rpsC | 30S ribosomal protein S3 |
| NP_461496 | glnB | regulatory protein P-II |
| NP_460161 | rpmF | 50S ribosomal protein L32 |
| NP_462124 | rpsU | 30S ribosomal protein S21 |
| NP_462405 | ompR | osmolarity response regulator |
| NP_463018 | rplK | 50S ribosomal protein L11 |
| NP_462208 | ftsJ | 23S rRNA methyltransferase |
| NP_462627 | rpmG | 50S ribosomal protein L33 |
| NP_462769 | atpE | ATP synthase subunit C |
| NP_460301 | rpmI | 50S ribosomal protein L35 |
| NP_461212 | rcsB | response regulator |
| NP_463454 | arcA | response regulator |
| NP_459678 | fur | ferric uptake regulator |
