# Supplementary material for: Gene socialization: gene order, GC content and gene silencing in Salmonella
Source: BMC Genomics. 2009 Dec 11;10:597. doi: 10.1186/1471-2164-10-597 (PMC2801525; doi:10.1186/1471-2164-10-597)
Supplement: Additional file 8 — Gene order conservation of H-NS repressed genes predicted to be HTGs is not associated to their GC content. We illustrate features of the subset of 130 H-NS repressed genes predicted to be HTGs (HNS-HTGs). More specifically, the distribution of HNS-HTGs genes in the three categories depending on gene order conservation largely deviates from the overall genome representation, with a striking under-representation of GCO genes. Additionally, the average GC content between these gene sets does not exhibit significant differences among the three gene classes (Kruskal-Wallis chi-squared = 1.1224, df = 2, P-value < 0.7717). This finding was expected since the prediction of the HTGs is mainly based on GC content (Garcia-Vallve S, Guzman E, Montero MA, Romeu A: HGT-DB: a database of putative horizontally transferred genes in prokaryotic complete genomes. Nucleic Acids Res 2003, 31(1):187-189.) [file 1471-2164-10-597-S8.DOC]

Correlation of gene order conservation with sequence identity and GC content (Salmonella vs E. coli K12). **(A)** Frequency distributions for pairwise sequence identities among proteins encoded by GCO genes and nGCO genes for various datasets used in this study. Box-and-whisker plots are used in order to graphically illustrate, in addition to the differences between the medians, the dispersion of the respective datasets. Orange and light blue have been chosen to represent GCO versus nGCO datasets for easy comparison. Average values are displayed on top of the box plots.The two leftmost box plots (GCO, nGCO) depict differences between those two gene classes within the overall protein sequence data set. Standard deviations were 12.8 and 21.7 respectively. A Wilcoxon rank-sum test provides statistical support for the difference between the sequence identities observed in the genes belonging to the two groups (W=1522118, P-value = 0). Similarly, for the next two data sets (D-GCO: Duplicated GCO genes, D-nGCO: Duplicated nGCO genes) standard deviations were 20.2 and 14.6 respectively. (Wilcoxon rank-sum test: W = 106315.5, P-value = 0). Regarding the set of H-NS repressed genes (HNS-GCO: GCO genes that are H-NS repressed, HNS-nGCO: nGCO genes that are H-NS repressed), standard deviations were 19.1 and 12.9 respectively. (Wilcoxon rank-sum test: W = 10908, P-value < 2.2e-16). We have alternatively calculated the poisson and gamma corrected distances for multiple substitutions as a measure of sequence divergence and we observed the same trends (Table S9). **(B)** GC content of the three categories of genes (GCO, nGCO and genes with no homolog in E. coli K12 - NH) for the various datasets used in this study. We have used the same coloring scheme with Fig. 1A, with the addition of light grey for sequences that had no homolog in E. coli K12. Within the overall dataset (GCO genes, nGCO genes, NH), standard deviations were 3.9, 5.5 and 7.5 respectively (Kruskal-Wallis chi-squared = 348.0419, df = 2, P-value < 2.2e-16). A similar trend was observed for the subset of duplicated genes (D-GCO: Duplicated GCO genes, D-nGCO: Duplicated nGCO genes) were standard deviations were 5.1 and 5.6 respectively (Wilcoxon rank-sum test: W = 79328.5, P-value = 5.329e-15). Significant differences were observed within the subset of H-NS repressed genes (HNS-GCO: GCO genes that are H-NS repressed, HNS-nGCO: nGCO genes that are H-NS repressed, HNS-NH: Genes with no homolog in E. coli K12). Standard deviations were 5.7, 5.2 and 6.0 respectively (Kruskal-Wallis chi-squared = 51.8533, df = 2, P-value < 5.498e-12). The dashed horizontal line corresponds to the overall GC content of *S.* Typhimurium genome (52.2%).
