# Supplementary material for: Gene socialization: gene order, GC content and gene silencing in Salmonella
Source: BMC Genomics. 2009 Dec 11;10:597. doi: 10.1186/1471-2164-10-597 (PMC2801525; doi:10.1186/1471-2164-10-597)
Supplement: Additional file 9 — Correlation of gene order conservation with poisson and gamma corrected distances for multiple substitutions. Poisson and gamma corrected distances for multiple substitutions were calculated as described in Yang Z., "Computational Molecular Evolution", Oxford University Press, 2006 (pp. 45 and 46 respectively). [file 1471-2164-10-597-S9.DOC]

|  | Mean/Median Poisson Corrected distance | Stdev Poisson Corrected distance | Mean/Median Gamma Corrected distance (alpha=2; Ota and Nei, 1994) | Stdev Gamma Corrected distance |
| --- | --- | --- | --- | --- |
| GCO | 0.1979 / 0.1445 | 0.2039032 | 0.2223 / 0.1501 | 0.2742560 |
| nGCO | 0.9467 / 1.0540 | 0.4744066 | 1.3220 / 1.4100 | 0.7568567 |
|  |  |  |  |  |
| Duplicates GCO | 0.3572 / 0.1990 | 0.3729465 | 0.4424 / 0.2097 | 0.5402101 |
| Duplicates nGCO | 1.1110 / 1.1710 | 0.3707512 | 1.5720 / 1.6190 | 0.6352742 |
|  |  |  |  |  |
| HNS GCO | 0.3463 / 0.2227 | 0.3509079 | 0.4233 / 0.2362 | 0.50689 |
| HNS nGCO | 1.1860 / 1.2600 | 0.3281556 | 1.6960 / 1.7870 | 0.567365 |

Asymptotic Wilcoxon rank sum test

data: Poisson corrected distances for GCO versus nGCO genes

W = 1522518, p-value < 2.2e-16

alternative hypothesis: true mu is not equal to 0

Asymptotic Wilcoxon rank sum test

data: Gamma corrected distances (alpha=2) for GCO versus nGCO genes

W = 1522518, p-value < 2.2e-16

alternative hypothesis: true mu is not equal to 0

Asymptotic Wilcoxon rank sum test

data: Poisson corrected distances for GCO versus nGCO duplicate genes

W = 106316, p-value < 2.2e-16

alternative hypothesis: true mu is not equal to 0

Asymptotic Wilcoxon rank sum test

data: Gamma corrected distances for GCO versus nGCO duplicate genes

W = 106316, p-value < 2.2e-16

alternative hypothesis: true mu is not equal to 0

Asymptotic Wilcoxon rank sum test

data: Poisson corrected distances for GCO versus nGCO H-NS regulated genes

W = 10907.5, p-value < 2.2e-16

alternative hypothesis: true mu is not equal to 0

Asymptotic Wilcoxon rank sum test

data: Gamma corrected distances for GCO versus nGCO H-NS regulated genes

W = 10907.5, p-value < 2.2e-16

alternative hypothesis: true mu is not equal to 0

Poisson and gamma corrected distances for multiple substitutions were calculated as described in Yang Z., "Computational Molecular Evolution", Oxford University Press, 2006 (pp. 45 and 46 respectively).
