# Supplementary material for: Gene socialization: gene order, GC content and gene silencing in Salmonella
Source: BMC Genomics. 2009 Dec 11;10:597. doi: 10.1186/1471-2164-10-597 (PMC2801525; doi:10.1186/1471-2164-10-597)
Supplement: Additional file 10 — A detailed legend of Fig. 1including statistical analysis. A Word DOC containing a full legend for figure 1. [file 1471-2164-10-597-S10.DOC]

|  | HNS-HTGs | |
| --- | --- | --- |
| ***Gene order status*** | ***Number of Genes (%)*** | ***Average %GC content (sd)*** |
| GCO | 26 (20.0) | 43.1 (3.6) |
| nGCO | 45 (34.6) | 42.9 (3.7) |
| No *E. coli* K12 homolog | 59 (45.4) | 42.0 (4.2) |
